# Functional Diversity of Carbohydrate-Active Enzymes Enabling a Bacterium to Ferment Plant Biomass

**DOI:** 10.1371/journal.pgen.1004773

**Published:** 2014-11-13

**Authors:** Magali Boutard, Tristan Cerisy, Pierre-Yves Nogue, Adriana Alberti, Jean Weissenbach, Marcel Salanoubat, Andrew C. Tolonen

**Affiliations:** 1 Genoscope, CEA, DSV, IG, Évry, France; 2 CNRS-UMR8030, Évry, France; 3 Department of Biology, Université d'Évry Val d'Essonne, Évry, France; MicroTrek Incorporated, United States of America

## Abstract

Microbial metabolism of plant polysaccharides is an important part of environmental carbon cycling, human nutrition, and industrial processes based on cellulosic bioconversion. Here we demonstrate a broadly applicable method to analyze how microbes catabolize plant polysaccharides that integrates carbohydrate-active enzyme (CAZyme) assays, RNA sequencing (RNA-seq), and anaerobic growth screening. We apply this method to study how the bacterium *Clostridium phytofermentans* ferments plant biomass components including glucans, mannans, xylans, galactans, pectins, and arabinans. These polysaccharides are fermented with variable efficiencies, and diauxies prioritize metabolism of preferred substrates. Strand-specific RNA-seq reveals how this bacterium responds to polysaccharides by up-regulating specific groups of CAZymes, transporters, and enzymes to metabolize the constituent sugars. Fifty-six up-regulated CAZymes were purified, and their activities show most polysaccharides are degraded by multiple enzymes, often from the same family, but with divergent rates, specificities, and cellular localizations. CAZymes were then tested in combination to identify synergies between enzymes acting on the same substrate with different catalytic mechanisms. We discuss how these results advance our understanding of how microbes degrade and metabolize plant biomass.

## Author Summary

Plant-fermenting bacteria are important for the global carbon cycle, human nutrition, and industrial production of renewable fuels and commodities from cellulosic biomass. Plants are primarily composed of heterogeneous polysaccharides, requiring plant-degrading microbes to encode many carbohydrate-active enzymes (CAZymes) to cleave different sugar linkages. Here we develop a broadly applicable method to study how microbes catabolize plant biomass by determining the combination of CAZymes that depolymerize each polysaccharide into sugars, how the cell alters global mRNA expression, and the efficiency with which each polysaccharide is metabolized. We apply this method to investigate how *Clostridium phytofermentans*, a bacterium encoding 171 CAZymes, ferments polysaccharides. We assimilate our results into a genetic model of how this bacterium metabolizes plant biomass and discuss how these results further our understanding of microbial plant fermentation.

## Introduction

Plants annually produce 200 billion tons of lignocellulosic biomass [1], which is metabolized by specialized microbes in diverse environments. For instance, recycling of plant biomass by soil [2] and marine [3] microbes is a key part of the global carbon cycle and intestinal bacteria ferment indigestible plant fiber to short chain fatty acids that constitute 60–85% of calories in ruminants and 5–10% in humans [4]. Further, as only 2% of cellulosic biomass is currently used by humans [5], it is a vast potential feedstock that industrial microbes could convert into energy and commodities. Elucidating how microbes depolymerize and metabolize plant biomass is thus important to understand carbon flow in the environment, to promote healthy human nutrition and prevent disease [6], and to develop industrial processes based on cellulosic bioconversion.

Most of plant biomass is in the cell wall, a macromolecular network of phenolic lignin and three types of polysaccharides (cellulose, hemicelluloses, and pectins) whose relative abundances vary widely among species and tissues (Table S1). The load bearing structure of the cell wall consists of cellulose fibrils tethered by various types of hemicellulose. Hemicellulose is enriched in xylan [7] and xyloglucan [8] in dicots, arabinoxylan in monocots [9], and galacto- and glucomannans in gymnosperms [10]. Outside the cell wall, mannans also act as storage polysaccharides in seeds [11], similar to starch. Pectins are cross-linked galacturonic acid-based polysaccharides that act in cellular adhesion and primary wall extension. More than 60% of pectin is often homogalacturonan (HG) [12], which is esterified with methanol to various degrees. Rhamnogalacturonan I (RGI) [13], the second most abundant pectin, can have galactan and arabinan side chains on the rhamnose residues [14]. Because plant tissues are composed of such heterogeneous polysaccharides, plant-degrading microbes express a myriad of carbohydrate-active enzymes (CAZymes) [15], each of which modifies or cleaves a specific type of sugar linkage.

Here we demonstrate a strategy for systematic analysis of the enzymatic machinery used by microbes to degrade and metabolize plant polysaccharides. Among these microbes, the plant-fermenting clostridia are of particular interest for being a dominant group in the human gut microbiome [6] and top candidates to transform cellulosic biomass into fuels and commodities [16], [17]. We studied *Clostridium phytofermentans* [18], a soil bacterium with 171 CAZyme-encoding genes (Table S2) including 116 glycoside hydrolases in 44 different families. We first quantified growth on comprehensive panel of plant polysaccharides and sugars (Table S3). Strand-specific RNA sequencing revealed all genes whose expression changed on the various substrates. In particular, we focused on up-regulated CAZyme genes and determined how they are organized into regulons that respond to specific polysaccharides. A set of 56 up-regulated CAZymes were cloned, purified, and an “each enzyme versus each substrate” screen quantified their abilities to bind and cleave plant polysaccharides. These enzymes were then tested in combination to identify synergies for polysaccharide degradation. We discuss how the results can be integrated to further our knowledge of how microbes metabolize plant biomass.

## Results/Discussion

### Growth on polysaccharides and sugars

**Figure 1:**
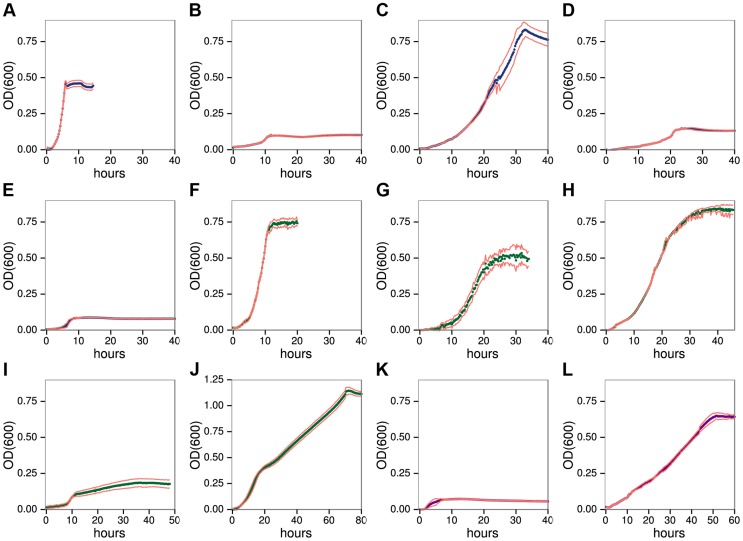
*C. phytofermentans* growth on pectic A–E, hemicellulosic F–J, and glucan K–L. Polysaccharides: homogalacturonan **A**, rhamnogalacturonan I **B**, galactan **C**, arabinan **D**, arabinogalactan II **E**, xylan **F**, arabinoxylan **G**, glucomannan **H**, galactomannan **I**, xyloglucan **J**, carboxymethylcellulose **K**, starch **L**. Growth was measured as OD_600_ every 15 minutes. Each point is the mean of six cultures; red lines show one standard deviation.

We developed a high resolution, microtiter anaerobic growth assay that shows *C. phytofermentans* ferments diverse plant polysaccharides (Fig. 1) and their constituent monosaccharides (Fig. S1), but with widely varying cell yields and growth rates (Table S4). It also forms colonies on solid medium containing each polysaccharide except arabinogalactan II (AGII) (Fig. S2). Growth was fastest on HG (Fig. 1A, generation time 0.70h), similar to rumen microbes that digest pectin more rapidly than cellulose and hemicellulose [19]. Although *C. phytofermentans* ferments both galacturonic acid (Fig. S1F) and rhamnose (Fig. S1H), cell yield was low on RGI (Fig. 1B). *C. phytofermentans* grows well on galactan (Fig. 1C), xylans (Fig. 1F–G), mannans (Fig. 1H–I), xyloglucan (Fig. 1J), and starch (Fig. 1L). Limited growth on AGII (Fig. 1E) relative to galactan supports that *C. phytofermentans* cleaves β-1,4 galactan, but not the β-1,3 and β-1,6-galactose bonds in AGII. Poor growth on arabinan (Fig. 1D) is similar to arabinose (Fig. S1G), suggesting this sugar is transported or metabolized inefficiently. *C. phytofermentans* grows well on cellulose plates (Fig. S2) and solubilizes cellulosic substrates such as filter paper and raw corn stover (Fig. S3), but weak growth on carboxymethylcellulose (CMC) might result from either lack of a suitable endoglucanase or carboxymethyl side groups inhibiting its metabolism.

*C. phytofermentans* shows diauxic growth on the mixed sugar polysaccharides galactomannan (Fig. 1I) and xyloglucan (Fig. 1J). For each of these substrates, one of the component sugars (galactose or glucose) supports faster growth than the other (mannose or xylose) (Fig. S1, Table S4). Growth on various mixtures of galactose/mannose (Fig. S4) and of glucose/xylose (Fig. S5) shows rapid metabolism of the preferred sugar followed by slower growth on the other one. However, in both cases when the favored sugar reached 75% of the total, the other sugar does not appear to be metabolized.

Similar to some ruminal [19] and human gut microbes [20], *C. phytofermentans* often grows faster on polysaccharides than the constituent sugars (Table S4). When presented with mixtures of xylan and xylose, this bacterium shows diauxic growth with preferential metabolism of xylan (Fig. S6), which is surprising because xylan must be cleaved to xylose to be metabolized. Growth on polysaccharides could be energetically favorable if significant ATP is saved by simultaneous transport of multiple sugar units in a single oligosaccharide [21] or by intracellular phosphorolysis of oligosaccharides [22]. *C. phytofermentans* encodes at least a dozen phosphorylases [23] [24], which cleave oligosaccharides without using ATP. Although the mechanisms regulating sugar metabolism in *C. phytofermentans* are unknown, diauxic growth supports carbon catabolite repression prioritizes growth on preferred sugars and polysaccharides.

### Gene expression

**Figure 2:**
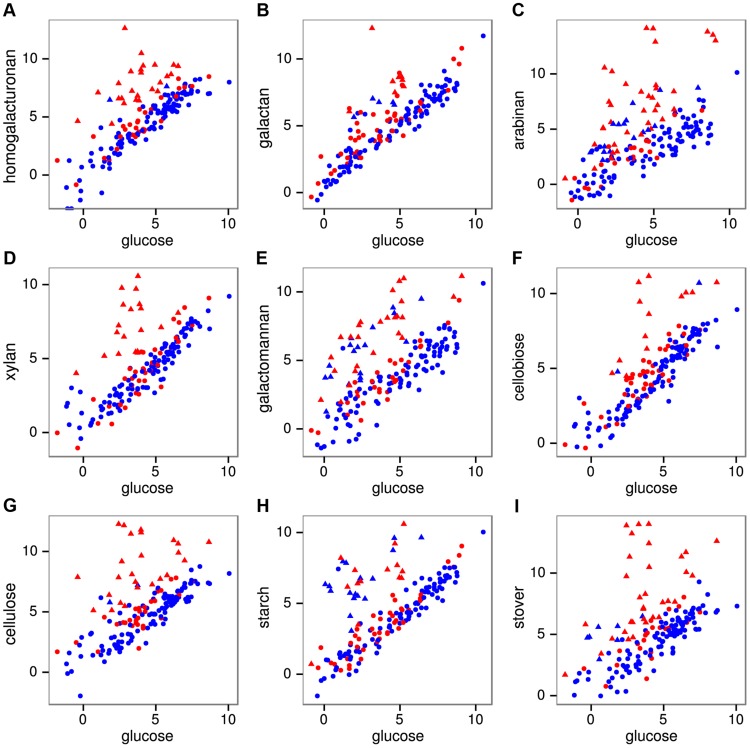
mRNA expression of all 171 CAZymes during growth on pectins A–C, hemicelluloses D–E, glucans F–H, and raw corn stover I relative to expression on glucose. Expression was quantified as log_2_(RPKM) with significantly differentially expressed genes on a given polysaccharide shown as triangles and unchanged genes as circles. The 56 purified CAZymes are red and others are blue.

We quantified mRNA expression by strand-specific RNA sequencing during log-phase growth on 8 polysaccharides, 3 monosaccharides, and raw corn stover as a complex biomass substrate. An average of 17.3 million mRNA reads were mapped per sample (Table S5), yielding expression (RPKM) values (Table S6) that were highly correlated (r^2^ = 0.96–0.99) between duplicate cultures for all conditions (Fig. S7). The reads were also highly strand-specific (Fig. S8), which will facilitate their future use for *de novo* transcriptome assembly, gene annotation and detection of antisense transcription. The fraction of reads mapping to CAZymes during growth on glucose was 2.0%, but this increased greatly on polysaccharides, especially cellulose (11.9%) and stover (31.0%). We assessed which genes were significantly differentially expressed on each polysaccharide relative to glucose using DESeq [25] (Table S7). Expression of CAZyme genes on polysaccharides relative to glucose (Fig. 2) shows that between 15 (cellobiose) and 40 (stover) CAZymes were significantly up-regulated per treatment (Table S8) with a total of 92 CAZymes up-regulated on at least one polysaccharide.

**Figure 3:**
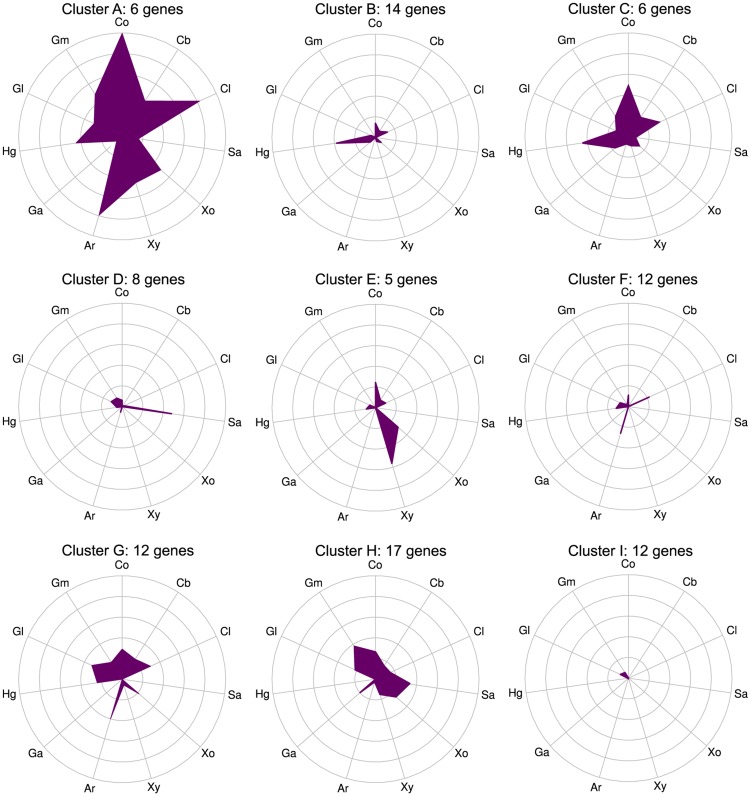
CAZymes clustered based on gene expression patterns (clusters A–I) show that some genes respond to multiple carbon sources while others are substrate-specific. mRNA expression changes (log_2_ expression ratios relative to glucose) for all 92 CAZyme genes differentially expressed on at least 1 polysaccharide relative to glucose were separated into nine clusters using K-means. Plot centers are expression on glucose and concentric rings show log_2_ up-regulation on the following carbon sources: cellobiose (Cb), filter paper cellulose (Cl), starch (Sa), xylose (Xo), xylan (Xy), arabinan (Ar), galacturonic acid (Ga), homogalacturonan (Hg), galactan (Gl), galactomannan (Gm), raw corn stover (Co). Gene membership of clusters is shown in Table S9.

The differentially expressed CAZymes are putatively classified by the CAZy database as 67 glycoside hydrolases, 6 carbohydrate esterases, 4 polysaccharide lyases, 14 glycosyl transferases, and 2 CBM proteins. We analyzed the specificity of the CAZyme transcriptional response by K-means clustering the expression profiles of these genes (Fig. 3, Table S9). Cluster A consists six genes that were highly up-regulated on multiple substrates: the GH26 *cphy1071*, the GH11 *cphy2105*, two GH18 chitinases *cphy1799* and *cphy1800* [26], the GH9 cellulase *cphy3367* [27] [28] and the GH48 cellulase *cphy3368* [29]. Clusters B–F respond to specific polysaccharides such as homogalacturonan (clusters B,C), starch (cluster D), xylan (cluster E), and cellulose/arabinan (cluster F). *C. phytofermentans* thus perceives signals from individual polysaccharides and responds by up-regulating specific transcriptional regulons that enable it to tailor its complement of CAZymes to the polysaccharide substrate.

### CAZyme activities

**Figure 4:**
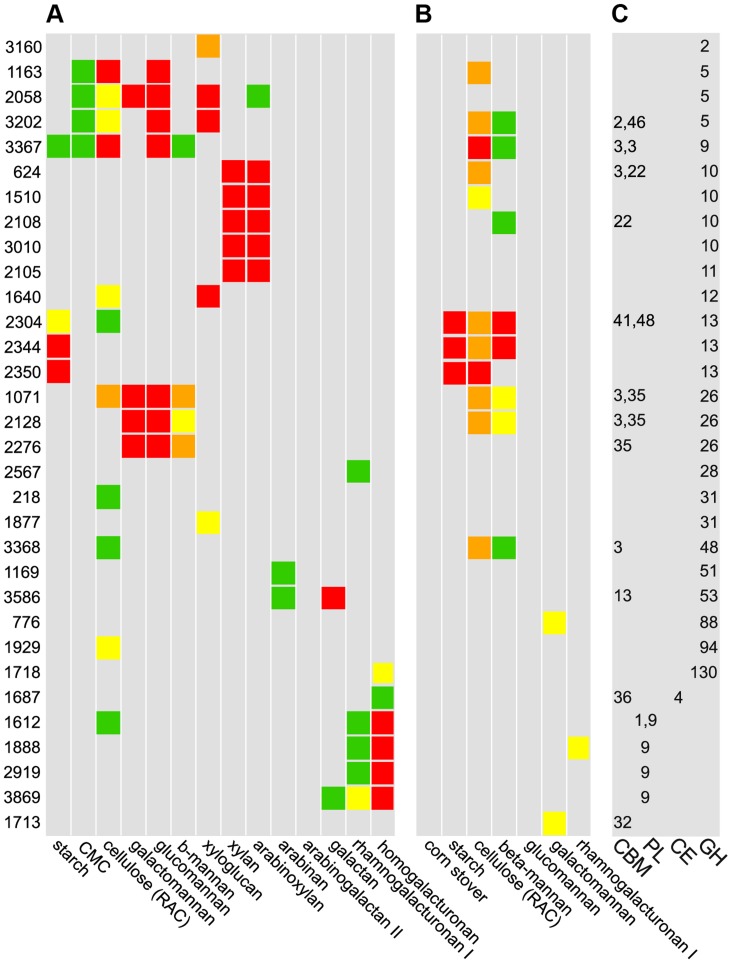
Cleavage A, binding B, and CAZy database classification C of purified enzymes. **A** Polysaccharide cleavage was quantified as nmol reducing sugar released per milligram enzyme per minute: >160 red, 80–160 orange, 40–80 yellow, 20–40 green, <20 gray. **B** Binding to insoluble polysaccharides was quantified as percentage enzyme bound to substrate: >30% red, 20–30% orange, 15–20% yellow, 10–15% green, <10% gray. **C** CAZy database classifications: glycoside hydrolases (GH), carbohydrate esterases (CE), polysaccharide lyases (PL), and carbohydrate binding domains (CBM). Among 56 purified CAZymes, only the 32 enzymes for which activities were found are shown.

A set of 56 CAZymes up-regulated on polysaccharides were His-tagged, overexpressed, purified, and their abilities to bind and cleave polysaccharides were quantified. The CAZy database classifies these enzymes putatively as 47 glycoside hydrolases, 4 polysaccharide lyases, and 4 carbohydrate esterases (Table S2); putative glycosyltransferases were not examined as they are not involved in polysaccharide catabolism [6]. Thirty-two enzymes have significant cleavage or binding activities (Fig. 4, Table S11). Some substrates such as β-1,4-galactan appear to be cut by a single, highly active enzyme, while multiple CAZymes from the same family degrade other substrates such as xylan (GH10), mannan (GH26), starch (GH13), and HG (PL9). CAZymes from multiple families together depolymerize substrates such as xyloglucan (GH2,5,12,31), glucomannan (GH5,GH9,GH26) and galactomannan (GH5,GH26).

We found 15 CAZymes that bind insoluble polysaccharides, most commonly cellulose and mannan (Fig. 4B). Unexpectedly, no CAZyme bound corn stover, suggesting that partial digestion of raw biomass is needed to facilitate enzyme binding. Nine enzymes that bound substrates have carbohydrate binding modules (CBM), but some enzymes such as the cellulase Cphy1163 can bind their substrate without one. While CBM are known to discriminate between polysaccharides such as cellulose and mannan [30], we observed overlap with cellulase CBMs binding mannan and vice versa. Further, CBM from xylanases can bind cellulose and mannose, but with lower affinity, showing that CBM often bind a range of polysaccharides. Consistent with their cleavage activities, GH13 were the only enzymes to bind starch. Enzymes with CBM usually also have catalytic modules, but Cphy1713, a CAZyme with a CBM32 and no catalytic module, binds galactomannan. CBM32 are known to bind galactose and this protein may function similar to one in Yersinia that is proposed to bind oligosaccharides to prevent them from leaking out of the cell [31].

**Figure 5:**
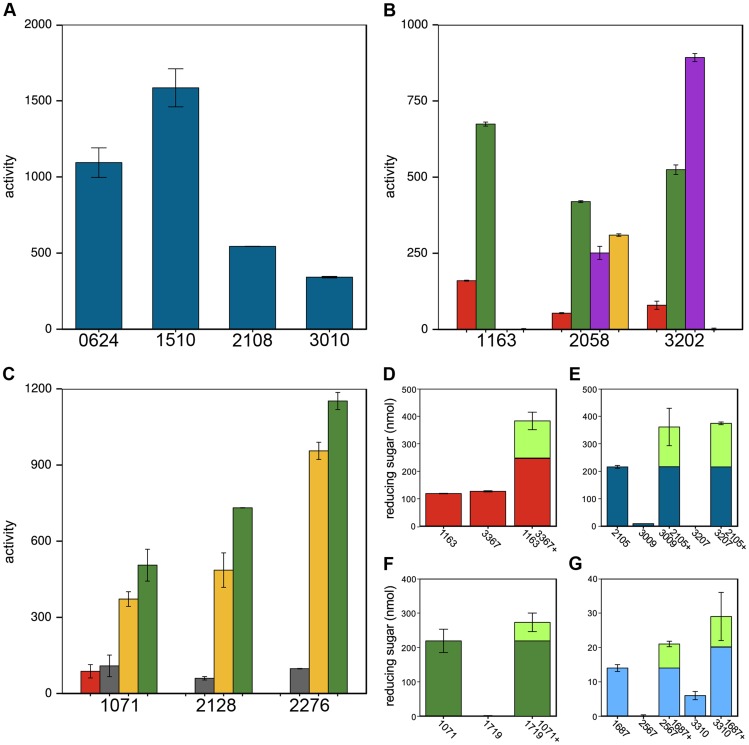
Members of the same CAZy family vary in polysaccharide cleavage activities and CAZymes can by potentiated by other enzymes. **A** Variation in cleavage activities of GH10 enzymes on xylan. **B** GH5 and **C** GH26 family members differ in their activities and substrate specificities on amorphous cellulose (red), glucomannan (green), xyloglucan (violet), galactomannan (yellow), mannan (gray). Enzyme activities in **A**–**C** are nmol reducing sugar released per milligram enzyme per minute. **D**–**G** CAZyme mixtures have higher activities than the individual enzymes. **D** Cphy1163 and Cphy3367 alone and together on amorphous cellulose. **E** Cphy2105, Cphy3009, and Cphy3207 alone and the latter two enzymes plus Cphy2105 on xylan. **F** Cphy1719 and Cphy1071 alone and together on glucomannan. **G** Cphy1687, Cphy2567, and Cphy3310 alone and the latter two enzymes plus Cphy1687 on homogalacturonan. In **D**–**G**, enzyme activities are shown as reducing sugar (nmol) produced by individual and combined enzymes. The fraction of the reducing sugar produced by the mixed enzymes that exceeds the sum of the individual enzymes is shown in green.

Thirty-two CAZy families have multiple members, which often have divergent cleavage activities and cellular localizations. Cphy1510 has the highest activity among the four GH10 active on xylan (Fig. 5A). Cphy3010, the GH10 with lowest activity, is the only one lacking a secretion signal, supporting it acts intracellularly on xylo-oligosaccharides while the other GH10 are extracellular. Members of the GH5 family act on a wide range of polysaccharides [32]. *C. phytofermentans* encodes 3 GH5 enzymes, among which one is active on galactomannan and two on xyloglucan (Fig. 5B). The GH5 Cphy1163 has no activity on either of these substrates, but is the most active on cellulose and glucomannan. The 3 GH26 also vary in substrate specificities (Fig. 5C); all the GH26 are similarly active on β-mannan, but only Cphy1071 has cellulase activity and it has lower activity on gluco- and galactomannan. Sequenced-based families are thus useful to make general substrate predictions for CAZymes, but experiments are needed to determine substrate range and catalytic efficiency.

CAZymes mixtures can degrade polysaccharides more efficiently than individual enzymes. We assessed pairwise interactions between each CAZyme and a second enzyme on cellulose (Cphy3367), xylan (Cphy2105), glucomannan (Cphy1071), and homogalacturonan (Cphy1687) (Fig. 5D–G). Similar to results showing synergy between the GH9 Cphy3367 and a *B. subtilis* GH5 [33], we found that a mix of Cphy3367 and the GH5 Cphy1163 has higher activity on cellulose than either enzyme alone (Fig. 5D), supporting they have complementary roles in cellulolysis. CAZymes can also potentiate other enzymes that have no activity by themselves. For example, the xylanase Cphy2105 activates the putative xylosidases Cphy3009 and Cphy3207 on xylan (Fig. 5E). Similarly, Cphy1071 activates the putative mannosidase Cphy1719 on glucomannan (Fig. 5F). Activities of the GH28 Cphy2567 and Cphy3310 are enhanced by the carbohydrate esterase Cphy1687 (Fig. 5G), supporting this enzyme demethylesterifies homogalacturonan to facilitate its degradation. This carbohydrate esterase did not, however, increase cleavage by the PL9 enzymes that were the most active on homogalacturonan.

Global correlations between CAZyme mRNA expression and cleavage activities were weak for all polysaccharides (Fig. S12), mostly because many CAZyme genes are up-regulated on substrates upon which they have no activity. CAZymes up-regulated on multiple substrates (Fig. 3, cluster A) may act as ‘carbon scouts’ [34] that degrade complex substrates into inducing molecules used to fine-tune the expression of hydrolytic enzymes. As described above, some CAZymes such as xylosidases (Fig. 5D) are inactive on intact xylan, but are potentiated by other xylanases. The GH18 Cphy1799 and Cphy1800 are the most highly-upregulated CAZymes on cellulose (Fig. S12), but are chitinases with no activity on cellulose or other plant substrates [26]. As such, the set of up-regulated CAZymes is useful to identify active enzymes, but strong up-regulation does not necessarily indicate activity on a given substrate.

### Conclusions

**Figure 6:**
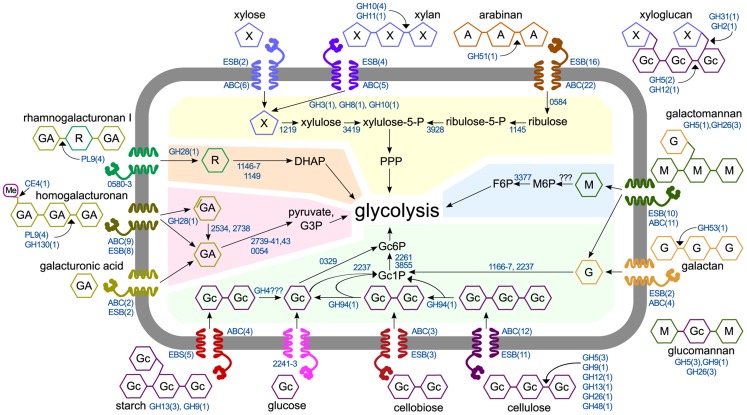
Model of polysaccharide degradation and metabolism by *C. phytofermentans*. CAZymes (shown as the number of enzymes in CAZy families) are based on purified activities and are intra- or extracellular based on putative secretion signals. Metabolic enzymes are shown as NCBI numbers and are proposed based on mRNA expression. Rhamnose transport and assimilation is based on pathway from [55]. Abbreviations are D-galacturonic acid (GA), L-rhamnose (R), D-mannose (M), D-glucose (Gc), D-galactose (G), D-xylose (X), L-arabinose (A), fructose (F), phosphate (P), pentose phosphate pathway (PPP), dihydroxyacetone-phosphate (DHAP), glyceraldehyde-3-phosphate (G3P). For each substrate, the number of significantly up-regulated extracellular solute binding proteins (ESB) and ABC transporters (ABC) are shown. Shaded regions show metabolism of glucose (green), mannose (blue), xylose and arabinose (yellow), rhamnose (orange), and galacturonic acid (red).

We assimilated our results into a model of *C. phytofermentans* polysaccharide catabolism that shows degradation by active CAZymes and uses mRNA expression profiles to predict how these substrates are transported and metabolized (Fig. 6). Unlike other clostridia that transport sugars with numerous phosphotransferase systems (PTS) [35] [36], *C. phytofermentans* encodes a single, lowly expressed PTS and also lacks the symporters to transport xylose and arabinose [37]. Instead, *C. phytofermentans* responds to carbon sources by up-regulating between two (galacturonic acid) and twenty-two (arabinan) ABC transporters (Fig. 6). Expression changes support that oligosaccharides and monosaccharides are uptaken by distinct transporters. For example, different ABC transporters are up-regulated on xylose and xylan. Similarly, different transporters respond to glucose, cellobiose, and cellulose. Intracellular cellodextrins are cleaved by at least one cellodextin phosphorylase (GH94); hexoses are phosphorylated, likely by a ROK hexokinase (Cphy0329) and a putative galactokinase (Cphy2237), and fed into glycolysis. While hexokinases may have wide substrate activity [38], poor growth on mannose could be due to inefficient mannose phosphorylation. The pentoses xylose and arabinose are isomerized and metabolized by the pentose phosphate pathway (PPP). Weak growth on arabinose could be due to inefficient transport or the lack of the phosphoketolase in the PPP enabling rapid L-arabinose metabolism by *C. acetobutylicum* [39].

Plant degrading microbes differ widely in their abilities to depolymerize and metabolize polysaccharides, likely reflecting niche differentiation to alleviate resource competition. Among soil clostridia, *C. thermocellum* ferments cellulose, but not xylan [40]. *C. cellulolyticum* grows faster on xylose than xylan and faster on cellobiose than glucose [41], both of which differ from *C. phytofermentans*. Similar specialization exists in the human gut microbiome where microbes catabolize different glycans in dietary fiber [20]. The strategy presented here of high-resolution anaerobic growth measurements, RNA sequencing, and CAZyme assays complements other methods such as proteomics [42] and metagenomics. Elucidating how microbes metabolize polysaccharides is key to understanding the function of plant-degrading microbial communities and to develop improved enzyme mixtures and recombinant microbes for industrial processing of plant biomass.

## Materials and Methods

### Growth measurements

*C. phytofermentans* was cultured anaerobically at 30°C in GS2 medium [43]. Growth was quantified in medium containing 3 g l^−1^ mono-, di-, or polysaccharides (Table S3 for product numbers) in 100-well microtiter plates (Bioscreen 9502550) that were sealed by press-fitting adhesive sheets (Qiagen 1018104) under the lids. Growth was measured every 15 minutes as OD_600_ at 30°C using a Thermo Scientific Bioscreen C with 30 seconds shaking before each reading. Growth was not measured for ß-mannan and amorphous cellulose cultures because these low solubility substrates occluded optical density measurements. Growth on insoluble substrates (15 g l^−1^ of 0.5×5 cm strips of filter paper cellulose or raw corn stover) was measured in 10 ml cultures as substrate consumption by collection on 11 µm filters by vacuum filtration, drying overnight at 65°C, and weighing. Growth on solid GS2 medium with 3 g l^−1^ polysaccharide and 15 g l^−1^ agar was tested by incubating plates anaerobically for 10 days at 30°C (Fig. S2). Regenerated amorphous cellulose (RAC) for GS2 agar plates and enzyme assays was prepared from Avicel PH-101 by phosphoric acid treatment [44].

### RNA-seq

Cultures containing soluble substrates were sampled for RNA-seq in mid-log phase. Filter paper cellulose cultures were sampled under the same conditions as in [45]; corn stover cultures were sampled after 3 days. Cells were arrested with RNA stabilization buffer, collected by centrifugation (5 kg, 5 min, 4°C), and RNA was isolated with an Ambion Ribopure Bacteria Kit (AM1925). Twenty µg total RNA was treated with Turbo DNase (Invitrogen AM1907), phenol-chloroform extracted, ethanol precipitated, and resuspended in 15 µl 10 mM Tris-Cl, pH 8.5. Ribosomal RNA was depleted from 10 µg RNA using a MICROBExpress kit (Invitrogen AM1905), giving a typical yield of 1–2 µg RNA. 500 ng of rRNA-depleted RNA was fragmented with magnesium buffer (NEB E6101) for 2.5 minutes at 94°C, ethanol precipitated, and resuspended in 10 µl water. RNA was resolved on an Agilent Bioanalyzer 2100 to confirm it was 200–800 bp.

Single stranded cDNA was made from 500 ng fragmented RNA by Superscript III first strand synthesis (Invitrogen 18080-051) including 200 ng/µl actinomycin D (Invitrogen 11805017) and 120 ng/µl random hexamers (Invitrogen 48190-011). Single stranded DNA was twice phenol-chloroform extracted, ethanol precipitated, and resuspended in 52 µl water. The second cDNA strand was synthesized by the Invitrogen double stranded cDNA synthesis kit (Invitrogen 11917-010) using 250 µM dNTP (dATP, dCTP, dGTP, dUTP). Double stranded cDNA was purified using 1.8 volumes of Solid Phase Reversible Immobilisation (SPRI) beads [46] prepared from carboxylate-modified microparticles [47] (ThermoScientific 6515-2105-050250) and resuspended in 50 µl 10 mM Tris-Cl, pH 8.5. DNA was resolved on an Agilent Bioanalyzer to confirm cDNA was 100–500 bp.

Sequencing libraries were prepared from ∼250 ng cDNA using the Illumina Truseq DNA Kit (Illumina FC-121-2001). DNA was purified with 1.8 volumes of SPRI beads after end repair. Following adapter ligation, 200–500 bp DNA was selected by Pippin Prep gel extraction (Sage Science). The second cDNA strand (contains dUTP) was then degraded by incubating with 1 unit USER enzyme (NEB NEB M5505S) at 37°C for 15 minutes, followed by 5 minutes at 95°C. Single stranded DNA was PCR amplified and size selected with 1.8 volumes of SPRI beads. Multiplexed libraries were normalized to 2 ng µl^−1^, pooled, and resolved on an Agilent Bioanalyzer to confirm the peak was at 200–500 bp. Libraries were sequenced on a Illumina HiSeq2000 sequencer. RNA-seq reads were mapped to the *C. phytofermentans* genome (NCBI NC_010001.1) using Bowtie [48] to report the single, best alignment (see Table S5 for parameters). Gene expression was measured as RPKM (sum of nucleotides in gene per million mapped nucleotides per gene kb) using RSEQtools [49] (Table S6), excluding rRNA reads from RPKM calculations. Differentially expressed genes (Tables S7, S8) were identified by DESeq [25] using the statistical thresholds defined in [50] of >4-fold expression and a p-value<10^−5^, which corresponds to a p-value<0.05 adjusted for multiple testing of the 3902 genes in *C. phytofermentans* genome by Bonferroni correction.

### Enzyme purification and activities

A set of 56 CAZymes (Table S2) that were up-regulated on plant polysaccharides were His-tagged, overexpressed, and purified. Primers (Table S10) were designed to clone CAZyme genes by Ligation-Independent Cloning [51] into pET-22B(+). Genes were cloned with C-terminal His-tags except for *cphy1687* and *cphy2105*, which were cloned with N-terminal tags to improve yields. The 26 enzymes predicted to be secreted by SignalP [52] were cloned as their mature forms, omitting the N-terminal secretion signals. Forward and reverse sequences of the 56 genes cloned in pET-22B(+) were confirmed by sequencing (Fig. S9). Plasmids were transformed into *E. coli* BL21(DE3) (Novagen 70235), grown in 50 ml TB medium to OD_600_ = 1.0, and induced by adding 500 µM IPTG and incubating overnight at 20°C. Cells were pelleted, resuspended in lysis buffer: 50 mM phosphate buffer pH = 8, 0.5M NaCl, 10 mM imidazole, 15% glycerol, 1 mM pefabloc (Sigma 76307). Cells were lysed by sonication (Cole-Parmer Vibracell CV33) in the presence of lysozyme (Novagen 71230). His-tagged proteins were purified from 50 ml culture on Ni-NTA spin columns (Qiagen 31014) and quantified by Bradford assay, giving an average yield of 3 mg protein. Purified proteins were visualized on 12% SDS-PAGE gels (Novex 12% Bis-Tris Gel NP0342BOX) (Fig. S10).

Enzymatic polysaccharide cleavage was quantified by 3,5-dinitrosalicylic acid (DNS) assay [53] in 96 well clear, flat bottom plates (Molecular Devices X6011). Fifty µg enzyme was incubated with 0.25% polysaccharide in 25 mM potassium phosphate buffer pH = 7 for 30 minutes at 37°C, 1 volume DNS reagent was added, incubated at 95°C for 15 minutes, and read at OD_600_. Enzyme activities (nmol reducing sugar per mg enzyme per min) were calculated from DNS readings by subtracting blanks (+polysaccharide,-enzyme) and calculating sugar produced using standard curves (Fig. S11). Polysaccharide cleavage by enzyme pairs was tested on amorphous cellulose, xylan, glucomannan, and homogalacturonan as the reducing sugar (mM) produced by 25 µg of each enzyme alone and combined with a second enzyme (25 µg Cphy3367 for cellulose, 5 µg Cphy2105 for xylan, 5 µg Cphy1071 for glucomannan, 25 µg Cphy1687 for homogalacturonan). Reducing sugar was measured by DNS assay after 30 minutes incubation as described above. Enzyme binding to insoluble polysaccharides was quantified using a method adapted from [54]. Fifty µg enzyme was suspended in 200 µl 0.1 M phosphate buffer pH = 7 with 6 mg polysaccharide and incubated with rotation for 5 h at 4°C. The polysaccharide was collected by centrifuging at 12 kg for 5 min and the enzyme concentration in the supernatant was calculated before and after incubation by Bradford assay, yielding the percentage of enzyme bound to the polysaccharide.

## Supporting Information

Figure S1 *C. phytofermentans* growth curves on 3 g l^−1^ di- and monosaccharides: A D-cellobiose, B D-glucose, C D-galactose, D D-xylose, E D-mannose, F D-galacturonic acid, G D-arabinose, H L-rhamnose. Blue curve is mean density (OD_600_) of 6 cultures; red curves show one standard deviation.
(PNG)

Figure S2 *C. phytofermentans* growth on solid GS2 medium containing 3 g l^−1^ polysaccharides. Plates were incubated anaerobically at 30°C for 10 days. Colonies were observed on all substrates except arabinogalactan II.
(PNG)

Figure S3 *C. phytofermentans* growth on 15 g l^−1^ A raw corn stover and B filter paper cellulose as a sole carbon source in GS2 medium. Growth was measured as consumption of insoluble substrate. Data are means of triplicate cultures; error bars are one standard deviation. Gray bars show when samples were taken for RNA-seq. The cellulose sample for RNA-seq was taken under the same conditions as those for proteomics in [45].
(PNG)

Figure S4 *C. phytofermentans* growth on mixtures of galactose and mannose (3 g l^−1^ total for all treatments): A galactomannan (80% mannose, 20% galactose), B D-galactose, C D-mannose, D 80% D-mannose and 20% D-galactose, E 50% D-mannose and 50% D-galactose, F 20% D-mannose and 80% D-galactose. Blue line shows mean OD_600_ of 6 cultures, red lines show range of one standard deviation. Growth on individual sugars shows that *C.phytofermentans* grows faster and to higher density on D-galactose than D-mannose. Growth is diauxic on galactomannan and sugar mixtures A, D, E, and F supporting that galactose is metabolized preferentially before mannose.
(PNG)

Figure S5 *C. phytofermentans* growth on mixtures of glucose and xylose (3 g l^−1^ total for all treatments): A xyloglucan (45% glucose, 35% xylose), B D-glucose, C D-xylose, D 75% D-glucose and 25% D-xylose, E 50% D-glucose and 50% D-xylose, F 25% D-glucose and 75% D-xylose. Blue line shows mean OD_600_ of 6 cultures, red lines show range of one standard deviation. Growth on individual sugars shows that *C.phytofermentans* grows faster and to higher density on D-glucose than on D-xylose. Growth is diauxic on xyloglucan and mixtures of glucose and xylose A, D, E, F supporting that D-glucose is metabolized preferentially before D-xylose.
(PNG)

Figure S6 *C. phytofermentans* growth on mixtures of xylan and xylose (3 g l^−1^ total): A xylan, B D-xylose, C 25% D-xylose and 75% xylan, D 50% D-xylose and 50% xylan, E 75% D-xylose and 25% xylan. Black curve is the mean OD_600_ of 6 cultures; the red curves show the range of 1 standard deviation. Growth curves show that xylan is metabolized before its constituent monomer, D-xylose.
(PNG)

Figure S7 Expression values (log_2_(RPKM)) for RNA sequencing of replicate cultures of all carbon source treatments are highly correlated. A–H are single-end reads and I–M are paired-end reads. Spearman correlation coefficients are shown on each panel.
(PNG)

Figure S8 Reads from dUTP sequencing are highly strand-specific with an average of 33,715 times more reads mapping to the expected strand for each of the 8 rRNA operons (16S-5S-23S) in the glucose samples. Operons 1–3 are transcribed in the positive direction; Operons 4–8 are in the reverse direction. Reads mapping to the expected strand are in magenta and the opposing strand are green. Note, dUTP sequencing reads map to the opposite strand. Thus, genes transcribed in the positive orientation are sequenced with reads on the reverse strand.
(PNG)

Figure S9 Forward and reverse sequence alignments of 56 CAZyme genes cloned into pET-22B(+) confirm that genes had the correct sequences.
(PDF)

Figure S10 Purified CAZyme visualized on 12% SDS-PAGE gels (Nupage bis-Tris novex gel IM-8042). Masses (kDa) of full proteins are shown for each CAZyme; masses with asterisks are secreted proteins for which the N-terminal secretion signal was not cloned, resulting in an expected size slightly smaller than the full protein mass shown. Mass ladders with blue borders are from images that were cropped by omitting intermediate lanes.
(PNG)

Figure S11 Sugar standard curves from DNS assays used to convert polysaccharide DNS readings to sugar concentrations.
(PNG)

Figure S12 Comparison of mRNA expression versus enzyme activity for purified CAZymes on A homogalacturonan, B galactan, C arabinan, D xylan, E galactomannan, F cellulose, G starch. mRNA expression is expressed as log_2_(RPKM) on the polysaccharide relative to glucose. Enzyme activity is log_2_(nmol sugar per mg enzyme per minute).
(PNG)

Table S1 Plant polysaccharides used in this study: natural abundances, plant source of tested compound, chemical structure, and sugar composition. References: [56] [57] [58] [59] [60] [61] [62] [63].
(XLS)

Table S2 *C. phytofermentans* CAZymes in the CAZy database [15] including gene name, NCBI accession, gene (bp) and protein (amino acid) length, predicted protein molecular weight (kDa), N-terminal secretion signal cleavage position (SignalP [52] Y-max position), CAZy classification, and annotation. The 56 CAZymes purified in this study are shown in blue.
(XLS)

Table S3 Vendor product information for mono-, di-, and polysaccharides used in this study.
(XLS)

Table S4 *C. phytofermentans* growth rates (generation time in hours) and cell yields (maximum OD_600_) growing on polysaccharides and di-/monosaccharides. Growth rate on each substrate was calculated by log-transforming the mean growth curve (Fig. 1,S1) and calculating the slop of a linear regression fit to the portion of the curve describing exponential growth. Regressions were fit to each of the two growth phases on galactomannan and xyloglucan.
(XLS)

Table S5 Mapping of RNA-seq reads to the *C. phytofermentans* genome, excluding reads mapping to rRNA operons. Reads were mapped using Bowtie [48] to report the single, best alignment between RNA-seq reads in.fastq files and the *C. phytofermentans* NCBI genome file (NC_010001.gbk). Reads were mapped using the following command: bowtie –best -k 1 [bowtie database of NC_010001.fna] –un unmappedReads.txt -q [.fastq file] mappedReads.txt.
(XLS)

Table S6 mRNA expression (RPKM) of all *C. phytofermentans* genes (page 1) and of only CAZymes (page 2) during steady state growth on sugars and polysaccharides. RPKM is defined as the sum of nucleotides from the reads that overlap with a given annotation entry normalized per million mapped nucleotides and the length of the annotation item. RPKM were calculated using RSEQtools [49] from mapped reads using MRFquant files. rRNA reads were removed from mrf files before running mrfQuant so that expression levels are normalized to mRNA reads. Samples are labeled whether they were sequenced using single-end (S) or paired-end (P) reads. The 56 CAZymes purified in this study are shown in blue on page 2.
(XLS)

Table S7 Differential expression of all *C. phytofermentans* genes on nine polysaccharides relative to glucose. Differentially expressed genes (DEseq p-value<10^−5^ and a >4-fold differential expression) are shown in blue. Sheets show differential expression relative to glucose of the following carbon sources: homogalacturonan, galactan, arabinan, xylan, galactomannan, cellulose, cellobiose, starch, stover. Paired-end sequenced polysaccharide samples are compared to the paired-end glucose sample; Single-end sequenced polysaccharide samples are compared to the single-end glucose sample (see Table S6).
(XLS)

Table S8 Differentially expressed CAZyme genes on nine polysaccharides relative to glucose. Page 1 shows all 92 differentially expressed CAZymes and the polysaccharides upon which they were up-regulated. Subsequent pages show the expression values of CAZymes up-regulated on specific polysaccharides. Purified CAZymes are shown in blue. Proportion of CAZyme genes with significant mRNA expression changes that were purified: homogalacturonan (25/27), galactan (8/13), arabinan (37/54), xylan (19/19), galactomannan (28/45), cellobiose (13/15), cellulose (26/27), starch (14/32), stover (33/40).
(XLS)

Table S9 Membership of CAZyme genes in K-means clusters. The 92 differentially-expressed CAZymes were clustered based on their expression (log_2_ expression ratios relative to glucose) as shown in Fig. 3.
(XLS)

Table S10 Primers for LIC cloning of 56 CAZymes. LIC sequences that overlap with the pET-22B(+) cloning plasmid are shown in red. His-tag sequences are in green. Genes of putatively secreted proteins were cloned without their predicted N-terminal secretion signals (Table S1). Genes were cloned with C-terminal His-tags, except for Cphy1687 and Cphy2105, which were re-cloned with N-terminal His-tags to improve yield.
(XLS)

Table S11 Polysaccharide cleavage activities (nmol reducing sugar per mg enzyme per minute) of 56 purified CAZymes on 14 polysaccharides. Enzyme (50 µg) was incubated with 0.25% polysaccharide in 25 mM potassium phosphate buffer pH = 7 for 30 minutes at 37°C. Activities: >160 red, >80 orange, >40 yellow, >20 green.
(XLS)

## References

[1] Shekhar C (2011) Future fuel: could biomass be the new petroleum? Chem Biol 18: 1199–1200 10.1016/j.chembiol.2011.10.010 22035783

[2] Schwarz WH (2001) The cellulosome and cellulose degradation by anaerobic bacteria. Appl Microbiol Biotechnol 56: 634–649 10.1007/s002530100710 11601609

[3] Edwards JL, Smith DL, Connolly J, McDonald JE, Cox MJ, et al (2010) Identification of carbohydrate metabolism genes in the metagenome of a marine biofilm community shown to be dominated by gammaproteobacteria and bacteroidetes. Genes 1: 371–384 10.3390/genes1030371 24710093 PMC3966224

[4] McNeil NI (1984) The contribution of the large intestine to energy supplies in man. Am J Clin Nutr 39: 338–342. 6320630 10.1093/ajcn/39.2.338

[5] Pauly M, Keegstra K (2008) Cell-wall carbohydrates and their modification as a resource for biofuels. Plant J Cell Mol Biol 54: 559–568 10.1111/j.1365-313X.2008.03463.x 18476863

[6] El Kaoutari A, Armougom F, Gordon JI, Raoult D, Henrissat B (2013) The abundance and variety of carbohydrate-active enzymes in the human gut microbiota. Nat Rev Microbiol 11: 497–504 10.1038/nrmicro3050 23748339

[7] Deutschmann R, Dekker RFH (2012) From plant biomass to bio-based chemicals: latest developments in xylan research. Biotechnol Adv 30: 1627–1640 10.1016/j.biotechadv.2012.07.001 22776161

[8] Bauer WD, Talmadge KW, Keegstra K, Albersheim P (1973) The Structure of Plant Cell Walls: II. The Hemicellulose of the Walls of Suspension-cultured Sycamore Cells. Plant Physiol 51: 174–187 10.1104/pp.51.1.174 16658281 PMC367376

[9] Burke D, Kaufman P, McNeil M, Albersheim P (1974) The Structure of Plant Cell Walls: VI. A Survey of the Walls of Suspension-cultured Monocots. Plant Physiol 54: 109–115 10.1104/pp.54.1.109 16658824 PMC541512

[10] Capek P, Alföldi J, Lisková D (2002) An acetylated galactoglucomannan from *Picea abies* L. Karst. Carbohydr Res 337: 1033–1037 10.1016/S0008-6215(02)00090-3 12039544

[11] Buckeridge MS (2010) Seed cell wall storage polysaccharides: models to understand cell wall biosynthesis and degradation. Plant Physiol 154: 1017–1023 10.1104/pp.110.158642 20855518 PMC2971584

[12] Caffall KH, Mohnen D (2009) The structure, function, and biosynthesis of plant cell wall pectic polysaccharides. Carbohydr Res 344: 1879–1900 10.1016/j.carres.2009.05.021 19616198

[13] Ridley BL, O'Neill MA, Mohnen D (2001) Pectins: structure, biosynthesis, and oligogalacturonide-related signaling. Phytochemistry 57: 929–967 10.1016/S0031-9422(01)00113-3 11423142

[14] Mohnen D (2008) Pectin structure and biosynthesis. Curr Opin Plant Biol 11: 266–277 10.1016/j.pbi.2008.03.006 18486536

[15] Lombard V, Golaconda Ramulu H, Drula E, Coutinho PM, Henrissat B (2014) The carbohydrate-active enzymes database (CAZy) in 2013. Nucleic Acids Res 42: D490–495 10.1093/nar/gkt1178 24270786 PMC3965031

[16] Lynd LR, Weimer PJ, van Zyl WH, Pretorius IS (2002) Microbial cellulose utilization: fundamentals and biotechnology. Microbiol Mol Biol Rev MMBR 66: 506–577 10.1128/MMBR.66.3.506-577.2002 12209002 PMC120791

[17] Tolonen AC, Petit E, Blanchard JL, Warnick T, Leschine SB (2013) Technologies to Study Plant Biomass Fermentation Using the Model Bacterium *Clostridium phytofermentans*. In: J. Sun, S.Y. Ding, J.D. Peterson (Eds.), Biological conversion of biomass for fuels and chemicals, Royal Society of Chemistry, Cambridge, pp. 114–139. doi:10.1039/9781849734738-00114

[18] Warnick TA, Methé BA, Leschine SB (2002) *Clostridium phytofermentans* sp. nov., a cellulolytic mesophile from forest soil. Int J Syst Evol Microbiol 52: 1155–1160 10.1099/ijs.0.02125-0 12148621

[19] Hatfield RD, Weimer PJ (1995) Degradation characteristics of isolated and in situ cell wall lucerne pectic polysaccharides by mixed ruminal microbes. J Sci Food Agric 69: 185–196 10.1002/jsfa.2740690208

[20] Martens EC, Lowe EC, Chiang H, Pudlo NA, Wu M, et al (2011) Recognition and Degradation of Plant Cell Wall Polysaccharides by Two Human Gut Symbionts. PLoS Biol 9: e1001221 10.1371/journal.pbio.1001221 22205877 PMC3243724

[21] Muir M, Williams L, Ferenci T (1985) Influence of transport energization on the growth yield of *Escherichia coli* . J Bacteriol 163: 1237–1242. 3928598 10.1128/jb.163.3.1237-1242.1985 PMC219265

[22] Zhang Y-HP, Lynd LR (2005) Cellulose utilization by *Clostridium thermocellum*: bioenergetics and hydrolysis product assimilation. Proc Natl Acad Sci U S A 102: 7321–7325 10.1073/pnas.0408734102 15883376 PMC1129095

[23] Nihira T, Nakai H, Chiku K, Kitaoka M (2012) Discovery of nigerose phosphorylase from *Clostridium phytofermentans* . Appl Microbiol Biotechnol 93: 1513–1522 10.1007/s00253-011-3515-9 21808968

[24] Nakajima M, Nishimoto M, Kitaoka M (2009) Characterization of three beta-galactoside phosphorylases from *Clostridium phytofermentans*: discovery of d-galactosyl-beta1->4-l-rhamnose phosphorylase. J Biol Chem 284: 19220–19227 10.1074/jbc.M109.007666 19491100 PMC2740546

[25] Anders S, Huber W (2010) Differential expression analysis for sequence count data. Genome Biol 11: R106 10.1186/gb-2010-11-10-r106 20979621 PMC3218662

[26] Tolonen AC, Cerisy T, El-Sayyed H, Boutard M, Salanoubat M, et al (2014) Fungal lysis by a soil bacterium fermenting cellulose. Environ Microbiol 10.1111/1462-2920.12495 24798076

[27] Tolonen AC, Chilaka AC, Church GM (2009) Targeted gene inactivation in *Clostridium phytofermentans* shows that cellulose degradation requires the family 9 hydrolase Cphy3367. Mol Microbiol 74: 1300–1313 10.1111/j.1365-2958.2009.06890.x 19775243 PMC2810439

[28] Zhang X-Z, Sathitsuksanoh N, Zhang Y-HP (2010) Glycoside hydrolase family 9 processive endoglucanase from *Clostridium phytofermentans*: heterologous expression, characterization, and synergy with family 48 cellobiohydrolase. Bioresour Technol 101: 5534–5538 10.1016/j.biortech.2010.01.152 20206499

[29] Zhang X-Z, Zhang Z, Zhu Z, Sathitsuksanoh N, Yang Y, et al (2010) The noncellulosomal family 48 cellobiohydrolase from *Clostridium phytofermentans* ISDg: heterologous expression, characterization, and processivity. Appl Microbiol Biotechnol 86: 525–533 10.1007/s00253-009-2231-1 19830421

[30] Knox JP (2008) Revealing the structural and functional diversity of plant cell walls. Curr Opin Plant Biol 11: 308–313 10.1016/j.pbi.2008.03.001 18424171

[31] Abbott DW, Hrynuik S, Boraston AB (2007) Identification and characterization of a novel periplasmic polygalacturonic acid binding protein from *Yersinia enterolitica* . J Mol Biol 367: 1023–1033 10.1016/j.jmb.2007.01.030 17292916

[32] Aspeborg H, Coutinho PM, Wang Y, Brumer H, Henrissat B (2012) Evolution, substrate specificity and subfamily classification of glycoside hydrolase family 5 (GH5). BMC Evol Biol 12: 186 10.1186/1471-2148-12-186 22992189 PMC3526467

[33] Liao H, Zhang X-Z, Rollin JA, Zhang Y-HP (2011) A minimal set of bacterial cellulases for consolidated bioprocessing of lignocellulose. Biotechnol J 6: 1409–1418 10.1002/biot.201100157 21751395

[34] Benz JP, Chau BH, Zheng D, Bauer S, Glass NL, et al (2014) A comparative systems analysis of polysaccharide-elicited responses in *Neurospora crassa* reveals carbon source-specific cellular adaptations. Mol Microbiol 91: 275–299 10.1111/mmi.12459 24224966 PMC3900418

[35] Nölling J, Breton G, Omelchenko MV, Makarova KS, Zeng Q, et al (2001) Genome sequence and comparative analysis of the solvent-producing bacterium *Clostridium acetobutylicum* . J Bacteriol 183: 4823–4838 10.1128/JB.183.16.4823-4838.2001 11466286 PMC99537

[36] Al Makishah NH, Mitchell WJ (2013) Dual substrate specificity of an N-acetylglucosamine phosphotransferase system in *Clostridium beijerinckii* . Appl Environ Microbiol 79: 6712–6718 10.1128/AEM.01866-13 23995920 PMC3811504

[37] Servinsky MD, Kiel JT, Dupuy NF, Sund CJ (2010) Transcriptional analysis of differential carbohydrate utilization by *Clostridium acetobutylicum* . Microbiol Read Engl 156: 3478–3491 10.1099/mic.0.037085-0 20656779

[38] Chen M, Chen L, Zou Y, Xue M, Liang M, et al (2011) Wide sugar substrate specificity of galactokinase from *Streptococcus pneumoniae* TIGR4. Carbohydr Res 346: 2421–2425 10.1016/j.carres.2011.08.014 21903203

[39] Servinsky MD, Germane KL, Liu S, Kiel JT, Clark AM, et al (2012) Arabinose is metabolized via a phosphoketolase pathway in *Clostridium acetobutylicum* ATCC 824. J Ind Microbiol Biotechnol 39: 1859–1867 10.1007/s10295-012-1186-x 22922942

[40] Ng TK, Ben-Bassat A, Zeikus JG (1981) Ethanol Production by Thermophilic Bacteria: Fermentation of Cellulosic Substrates by Cocultures of *Clostridium thermocellum* and *Clostridium thermohydrosulfuricum* . Appl Environ Microbiol 41: 1337–1343. 16345787 10.1128/aem.41.6.1337-1343.1981 PMC243920

[41] Xu C, Huang R, Teng L, Wang D, Hemme CL, et al (2013) Structure and regulation of the cellulose degradome in *Clostridium cellulolyticum* . Biotechnol Biofuels 6: 73 10.1186/1754-6834-6-73 23657055 PMC3656788

[42] Tolonen AC, Haas W (2014) Quantitative Proteomics Using Reductive Dimethylation for Stable Isotope Labeling. J Vis Exp (89) e51416 10.3791/51416 PMC4210151 25045933

[43] Johnson EA, Madia A, Demain AL (1981) Chemically Defined Minimal Medium for Growth of the Anaerobic Cellulolytic Thermophile *Clostridium thermocellum* . Appl Environ Microbiol 41: 1060–1062. 16345748 10.1128/aem.41.4.1060-1062.1981 PMC243859

[44] Hong J, Ye X, Wang Y, Zhang Y-HP (2008) Bioseparation of recombinant cellulose-binding module-proteins by affinity adsorption on an ultra-high-capacity cellulosic adsorbent. Anal Chim Acta 621: 193–199 10.1016/j.aca.2008.05.041 18573384

[45] Tolonen AC, Haas W, Chilaka AC, Aach J, Gygi SP, et al (2011) Proteome-wide systems analysis of a cellulosic biofuel-producing microbe. Mol Syst Biol 7: 461 10.1038/msb.2010.116 21245846 PMC3049413

[46] DeAngelis MM, Wang DG, Hawkins TL (1995) Solid-phase reversible immobilization for the isolation of PCR products. Nucleic Acids Res 23: 4742–4743 10.1093/nar/23.22.4742 8524672 PMC307455

[47] Rohland N, Reich D (2012) Cost-effective, high-throughput DNA sequencing libraries for multiplexed target capture. Genome Res 22: 939–946 10.1101/gr.128124.111 22267522 PMC3337438

[48] Langmead B, Trapnell C, Pop M, Salzberg SL (2009) Ultrafast and memory-efficient alignment of short DNA sequences to the human genome. Genome Biol 10: R25 10.1186/gb-2009-10-3-r25 19261174 PMC2690996

[49] Habegger L, Sboner A, Gianoulis TA, Rozowsky J, Agarwal A, et al (2011) RSEQtools: a modular framework to analyze RNA-Seq data using compact, anonymized data summaries. Bioinforma Oxf Engl 27: 281–283 10.1093/bioinformatics/btq643 PMC3018817 21134889

[50] Mandlik A, Livny J, Robins WP, Ritchie JM, Mekalanos JJ, et al (2011) RNA-Seq-based monitoring of infection-linked changes in *Vibrio cholerae* gene expression. Cell Host Microbe 10: 165–174 10.1016/j.chom.2011.07.007 21843873 PMC3166260

[51] Aslanidis C, de Jong PJ (1990) Ligation-independent cloning of PCR products (LIC-PCR). Nucleic Acids Res 18: 6069–6074 10.1093/nar/18.20.6069. 2235490 PMC332407

[52] Petersen TN, Brunak S, von Heijne G, Nielsen H (2011) SignalP 4.0: discriminating signal peptides from transmembrane regions. Nat Methods 8: 785–786 10.1038/nmeth.1701 21959131

[53] Miller GL (1959) Use of Dinitrosalicylic Acid Reagent for Determination of Reducing Sugar. Anal Chem 31: 426–428 10.1021/ac60147a030

[54] Watanabe T, Ito Y, Yamada T, Hashimoto M, Sekine S, et al (1994) The roles of the C-terminal domain and type III domains of chitinase A1 from *Bacillus circulans* WL-12 in chitin degradation. J Bacteriol 176: 4465–4472. 8045877 10.1128/jb.176.15.4465-4472.1994 PMC196264

[55] Petit E, LaTouf WG, Coppi MV, Warnick TA, Currie D, et al (2013) Involvement of a bacterial microcompartment in the metabolism of fucose and rhamnose by *Clostridium phytofermentans* . PloS One 8: e54337 10.1371/journal.pone.0054337 23382892 PMC3557285

[56] Zablackis E, Huang J, Müller B, Darvill AG, Albersheim P (1995) Characterization of the cell-wall polysaccharides of *Arabidopsis thaliana* leaves. Plant Physiol 107: 1129–1138 10.1104/pp.107.4.1129 7770522 PMC157245

[57] Prasad S, Singh A, Joshi HC (2007) Ethanol as an alternative fuel from agricultural, industrial and urban residues. Resour Conserv Recycl 50: 1–39 10.1016/j.resconrec.2006.05.007

[58] Chow PS, Landhäusser SM (2004) A method for routine measurements of total sugar and starch content in woody plant tissues. Tree Physiol 24: 1129–1136 10.1093/treephys/24.10.1129 15294759

[59] Scheller HV, Ulvskov P (2010) Hemicelluloses. Annu Rev Plant Biol 61: 263–289 10.1146/annurev-arplant-042809-112315 20192742

[60] Dahal P, Nevins DJ, Bradford KJ (1997) Relationship of Endo-[beta]-D-Mannanase Activity and Cell Wall Hydrolysis in Tomato Endosperm to Germination Rates. Plant Physiol 113: 1243–1252 10.1104/pp.113.4.1243 12223672 PMC158247

[61] Pettolino FA, Walsh C, Fincher GB, Bacic A (2012) Determining the polysaccharide composition of plant cell walls. Nat Protoc 7: 1590–1607 10.1038/nprot.2012.081 22864200

[62] Oxenboll Sørensen S, Pauly M, Bush M, Skjøt M, McCann MC, et al (2000) Pectin engineering: modification of potato pectin by in vivo expression of an endo-1,4-beta-D-galactanase. Proc Natl Acad Sci U S A 97: 7639–7644 10.1073/pnas.130568297 10852969 PMC16598

[63] Moore JP, Nguema-Ona E, Chevalier L, Lindsey GG, Brandt WF, et al (2006) Response of the leaf cell wall to desiccation in the resurrection plant *Myrothamnus flabellifolius* . Plant Physiol 141: 651–662 10.1104/pp.106.077701 16603665 PMC1475438

