# Supplementary figures and images for: Functional Diversity of Carbohydrate-Active Enzymes Enabling a Bacterium to Ferment Plant Biomass
Source: PLoS Genet. 2014 Nov 13;10(11):e1004773. doi: 10.1371/journal.pgen.1004773 (PMC4230839; doi:10.1371/journal.pgen.1004773)

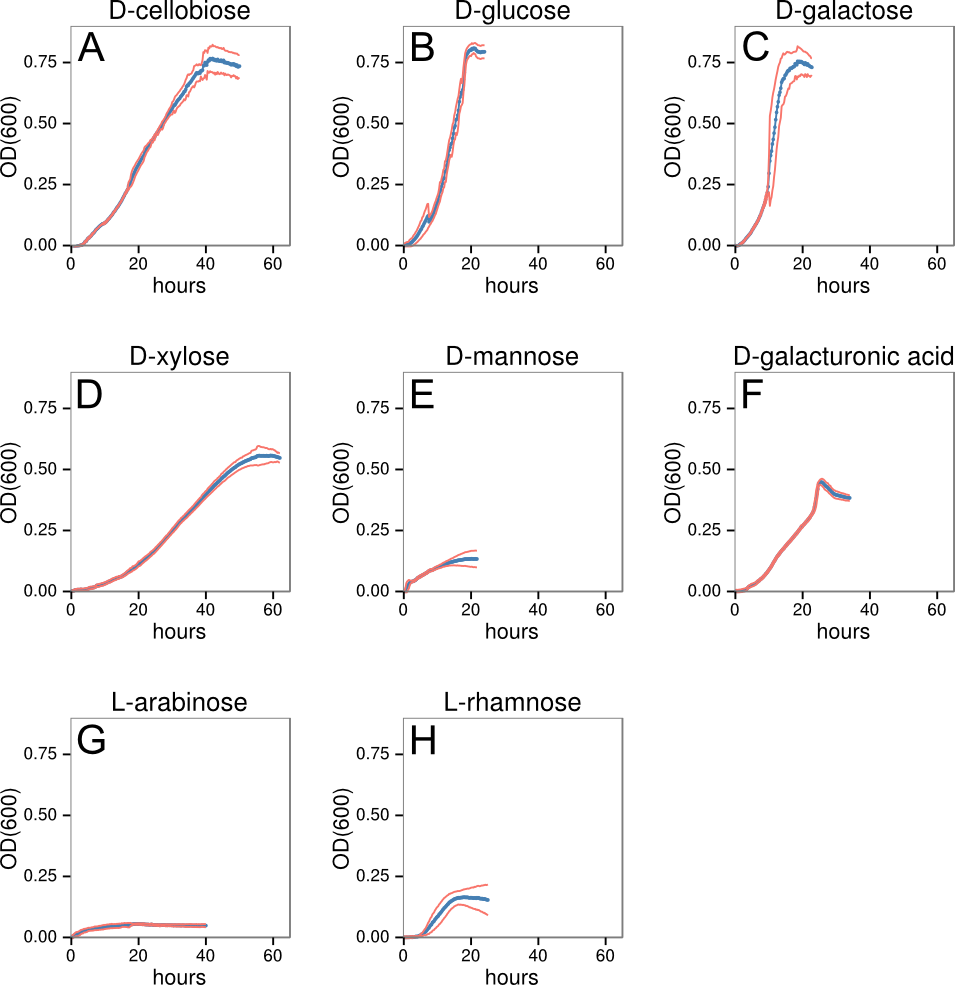

Supplement: Figure S1 — C. phytofermentans growth curves on 3 g l−1 di- and monosaccharides: A D-cellobiose, B D-glucose, C D-galactose, D D-xylose, E D-mannose, F D-galacturonic acid, G D-arabinose, H L-rhamnose. Blue curve is mean density (OD600) of 6 cultures; red curves show one standard deviation. (PNG) [file pgen.1004773.s001.png]

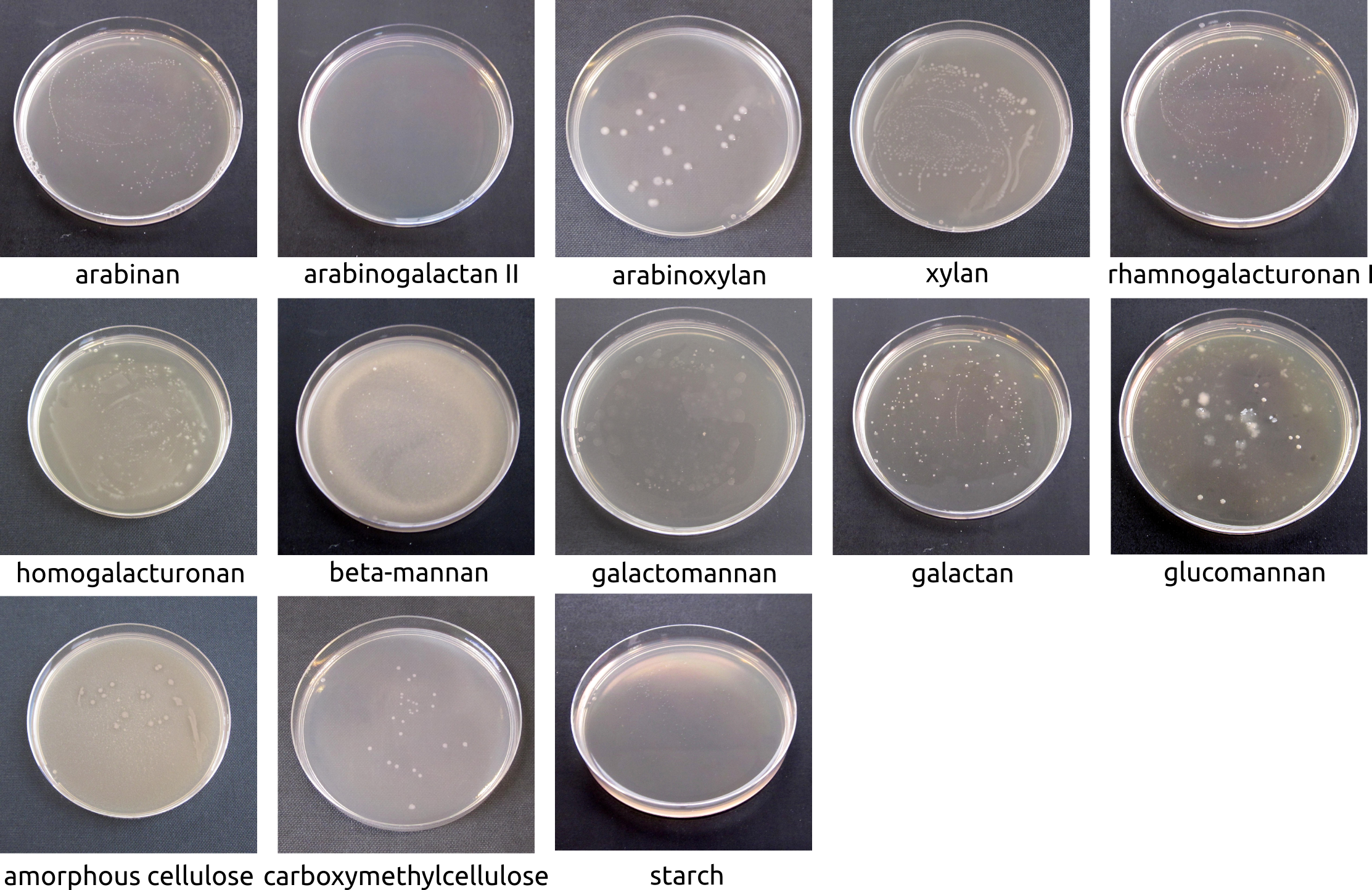

Supplement: Figure S2 — C. phytofermentans growth on solid GS2 medium containing 3 g l−1 polysaccharides. Plates were incubated anaerobically at 30°C for 10 days. Colonies were observed on all substrates except arabinogalactan II. (PNG) [file pgen.1004773.s002.png]

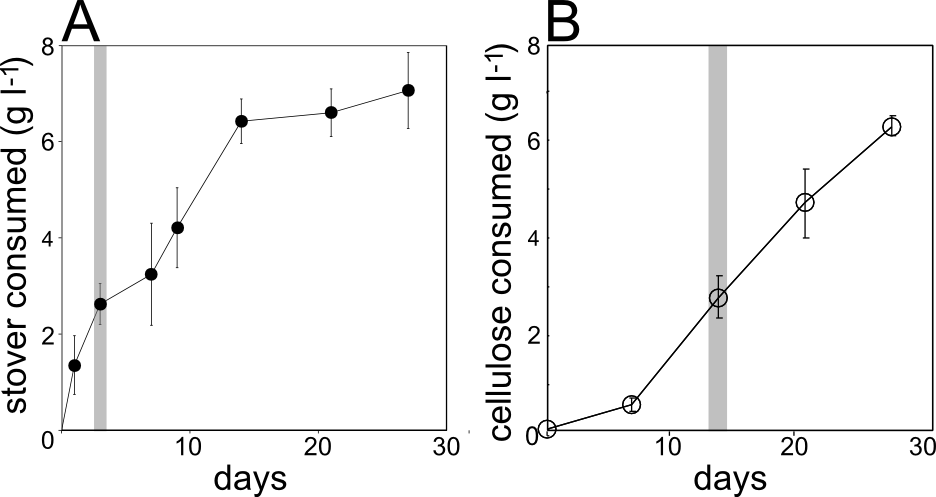

Supplement: Figure S3 — C. phytofermentans growth on 15 g l−1 A raw corn stover and B filter paper cellulose as a sole carbon source in GS2 medium. Growth was measured as consumption of insoluble substrate. Data are means of triplicate cultures; error bars are one standard deviation. Gray bars show when samples were taken for RNA-seq. The cellulose sample for RNA-seq was taken under the same conditions as those for proteomics in [45]. (PNG) [file pgen.1004773.s003.png]

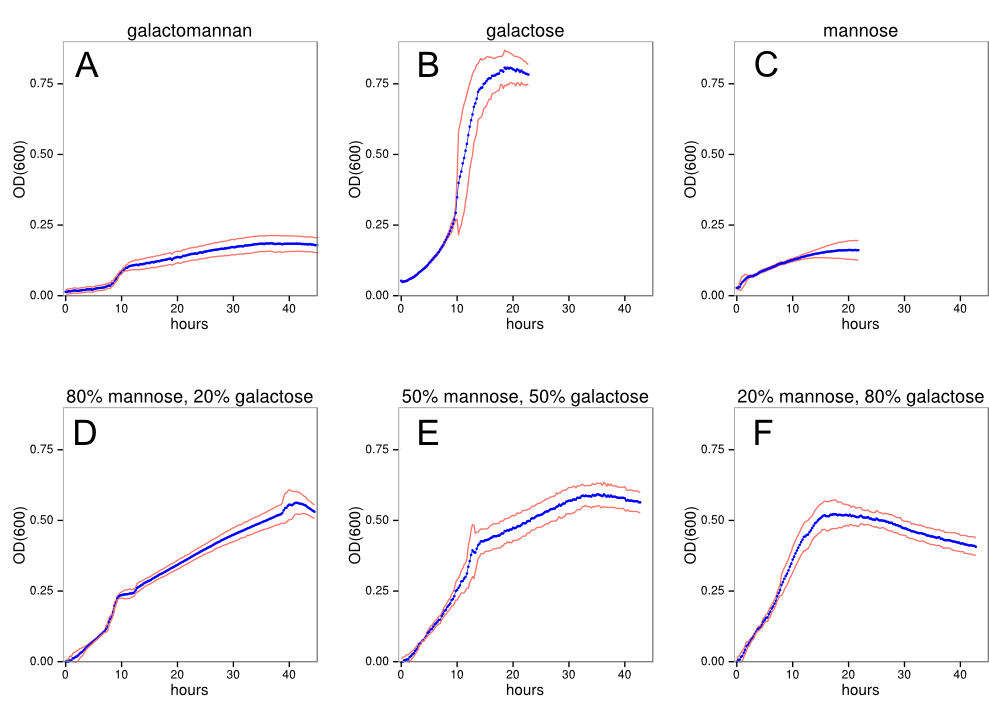

Supplement: Figure S4 — C. phytofermentans growth on mixtures of galactose and mannose (3 g l−1 total for all treatments): A galactomannan (80% mannose, 20% galactose), B D-galactose, C D-mannose, D 80% D-mannose and 20% D-galactose, E 50% D-mannose and 50% D-galactose, F 20% D-mannose and 80% D-galactose. Blue line shows mean OD600 of 6 cultures, red lines show range of one standard deviation. Growth on individual sugars shows that C.phytofermentans grows faster and to higher density on D-galactose than D-mannose. Growth is diauxic on galactomannan and sugar mixtures A, D, E, and F supporting that galactose is metabolized preferentially before mannose. (PNG) [file pgen.1004773.s004.png]

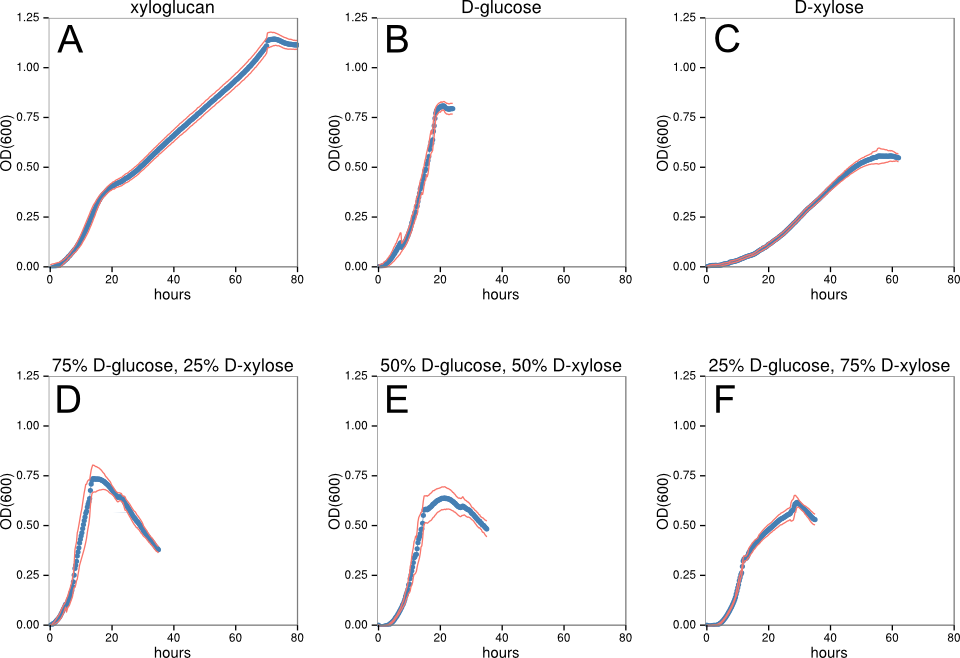

Supplement: Figure S5 — C. phytofermentans growth on mixtures of glucose and xylose (3 g l−1 total for all treatments): A xyloglucan (45% glucose, 35% xylose), B D-glucose, C D-xylose, D 75% D-glucose and 25% D-xylose, E 50% D-glucose and 50% D-xylose, F 25% D-glucose and 75% D-xylose. Blue line shows mean OD600 of 6 cultures, red lines show range of one standard deviation. Growth on individual sugars shows that C.phytofermentans grows faster and to higher density on D-glucose than on D-xylose. Growth is diauxic on xyloglucan and mixtures of glucose and xylose A, D, E, F supporting that D-glucose is metabolized preferentially before D-xylose. (PNG) [file pgen.1004773.s005.png]

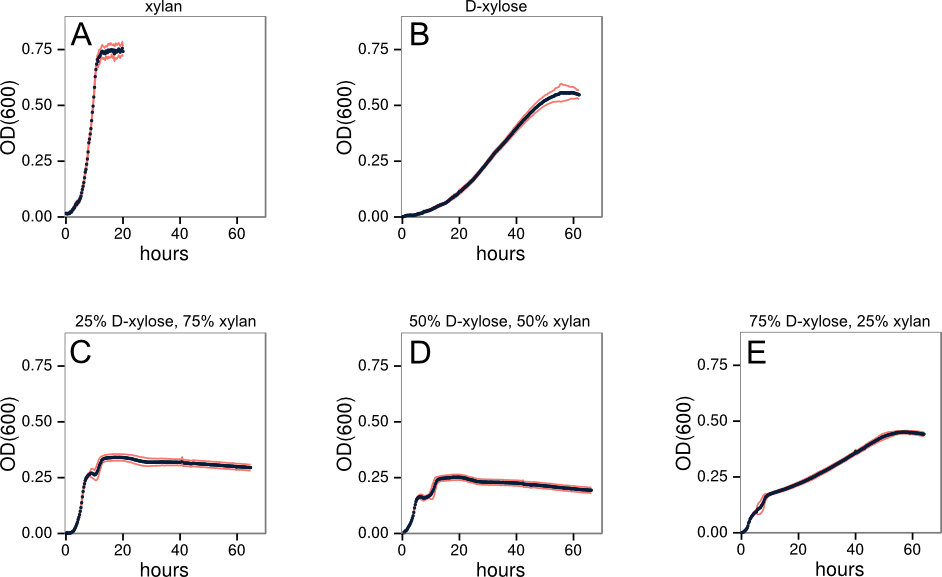

Supplement: Figure S6 — C. phytofermentans growth on mixtures of xylan and xylose (3 g l−1 total): A xylan, B D-xylose, C 25% D-xylose and 75% xylan, D 50% D-xylose and 50% xylan, E 75% D-xylose and 25% xylan. Black curve is the mean OD600 of 6 cultures; the red curves show the range of 1 standard deviation. Growth curves show that xylan is metabolized before its constituent monomer, D-xylose. (PNG) [file pgen.1004773.s006.png]

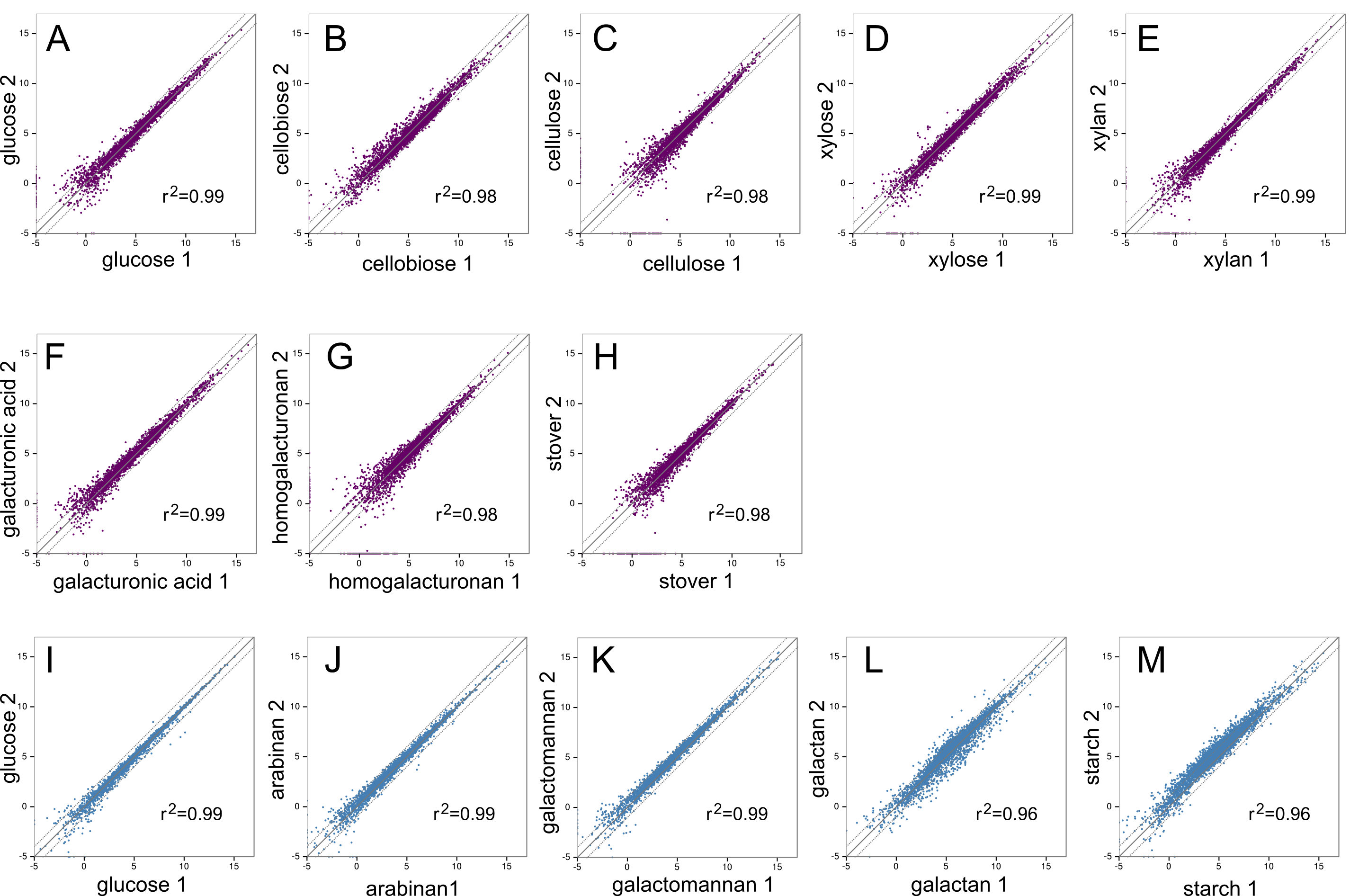

Supplement: Figure S7 — Expression values (log2(RPKM)) for RNA sequencing of replicate cultures of all carbon source treatments are highly correlated. A–H are single-end reads and I–M are paired-end reads. Spearman correlation coefficients are shown on each panel. (PNG) [file pgen.1004773.s007.png]

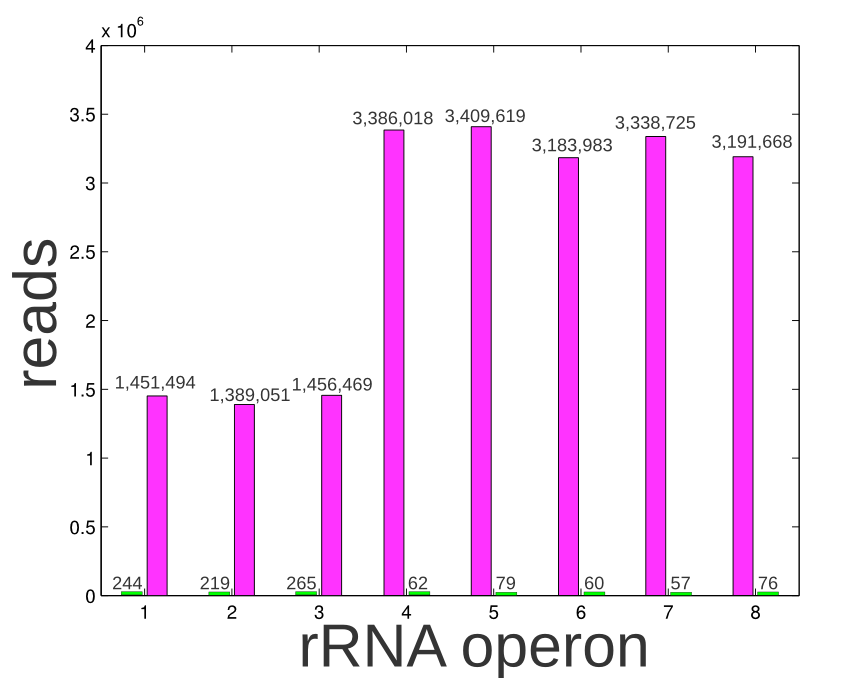

Supplement: Figure S8 — Reads from dUTP sequencing are highly strand-specific with an average of 33,715 times more reads mapping to the expected strand for each of the 8 rRNA operons (16S-5S-23S) in the glucose samples. Operons 1–3 are transcribed in the positive direction; Operons 4–8 are in the reverse direction. Reads mapping to the expected strand are in magenta and the opposing strand are green. Note, dUTP sequencing reads map to the opposite strand. Thus, genes transcribed in the positive orientation are sequenced with reads on the reverse strand. (PNG) [file pgen.1004773.s008.png]

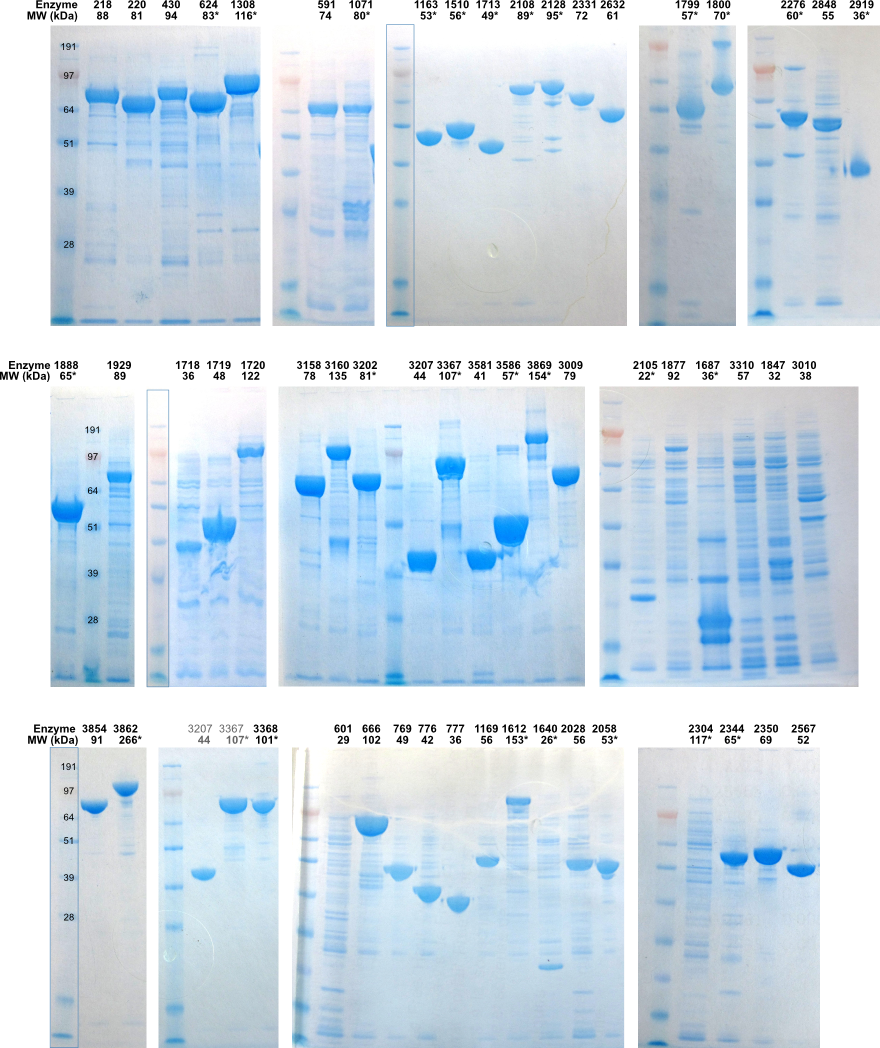

Supplement: Figure S10 — Purified CAZyme visualized on 12% SDS-PAGE gels (Nupage bis-Tris novex gel IM-8042). Masses (kDa) of full proteins are shown for each CAZyme; masses with asterisks are secreted proteins for which the N-terminal secretion signal was not cloned, resulting in an expected size slightly smaller than the full protein mass shown. Mass ladders with blue borders are from images that were cropped by omitting intermediate lanes. (PNG) [file pgen.1004773.s010.png]

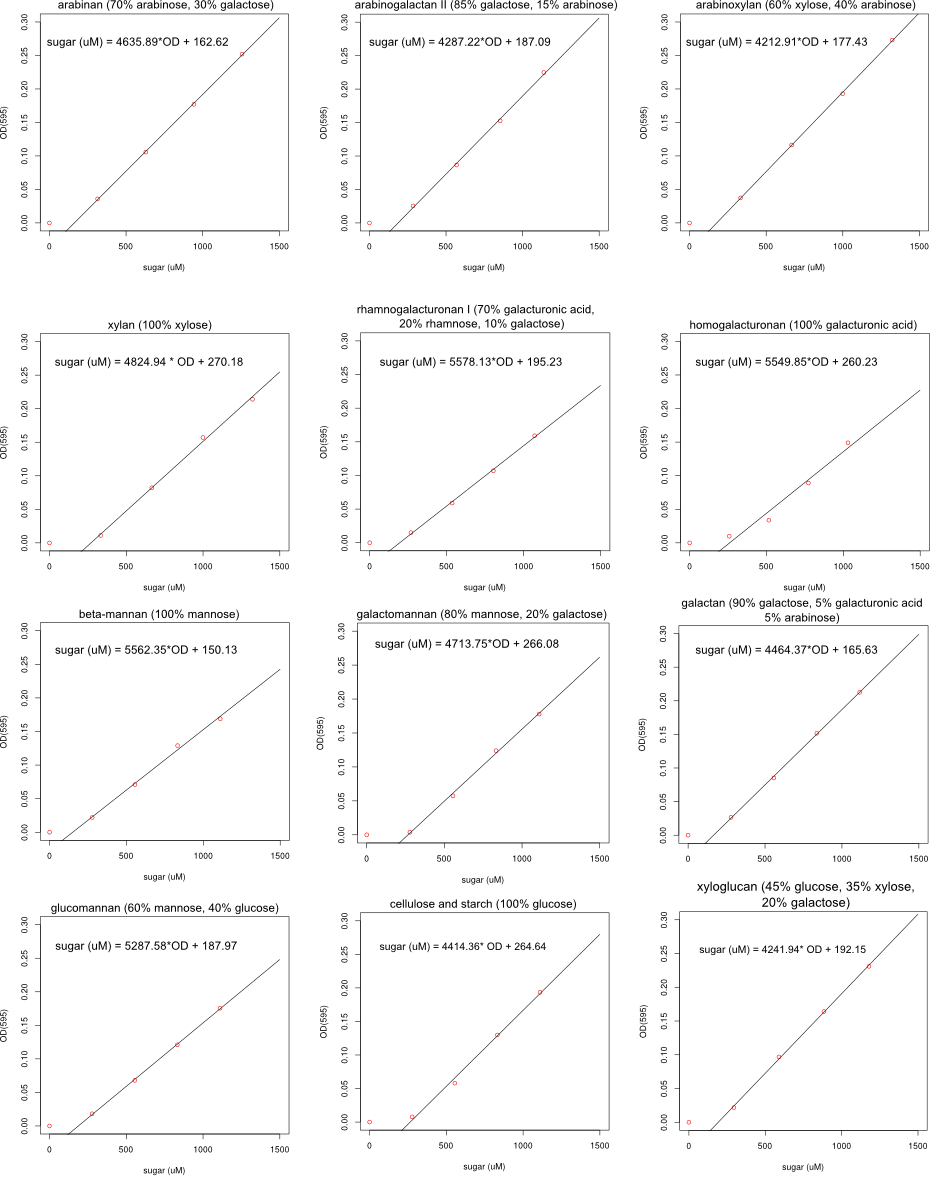

Supplement: Figure S11 — Sugar standard curves from DNS assays used to convert polysaccharide DNS readings to sugar concentrations. (PNG) [file pgen.1004773.s011.png]

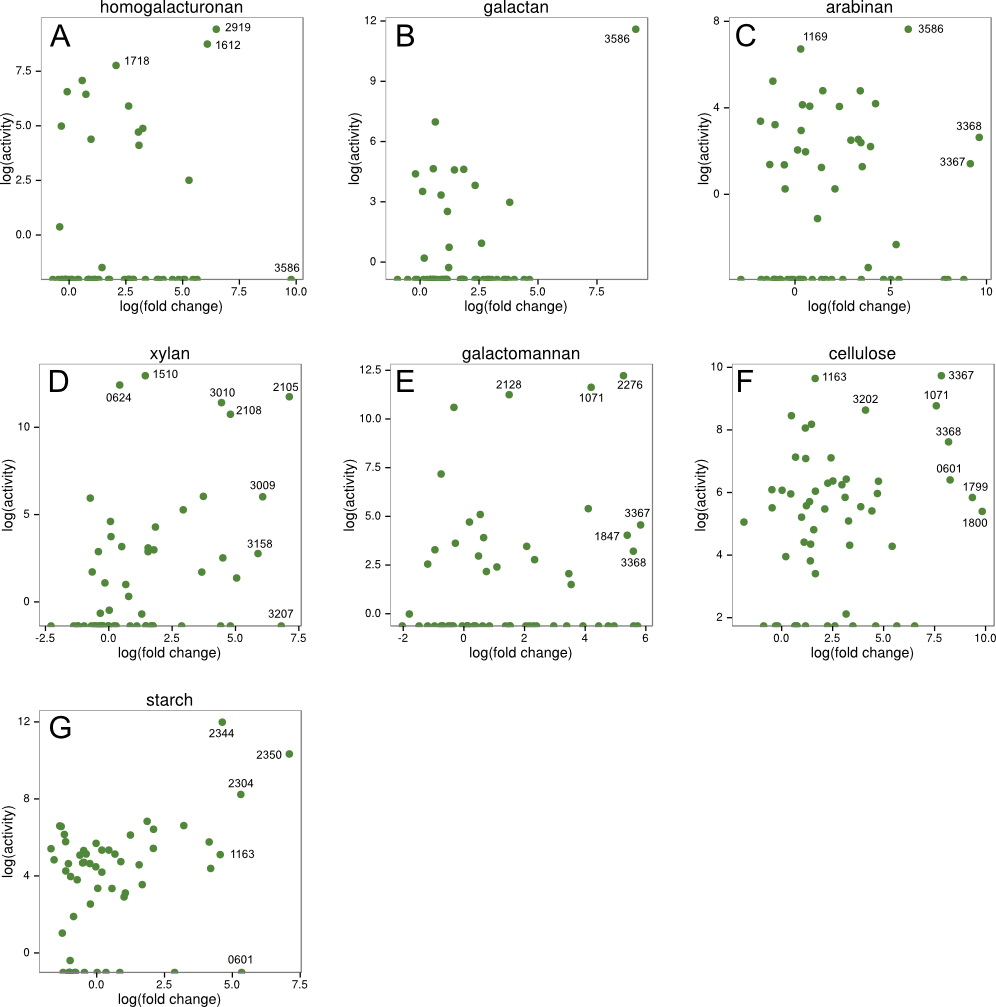

Supplement: Figure S12 — Comparison of mRNA expression versus enzyme activity for purified CAZymes on A homogalacturonan, B galactan, C arabinan, D xylan, E galactomannan, F cellulose, G starch. mRNA expression is expressed as log2(RPKM) on the polysaccharide relative to glucose. Enzyme activity is log2(nmol sugar per mg enzyme per minute). (PNG) [file pgen.1004773.s012.png]
