# Supplementary material for: Functional Diversity of Carbohydrate-Active Enzymes Enabling a Bacterium to Ferment Plant Biomass
Source: PLoS Genet. 2014 Nov 13;10(11):e1004773. doi: 10.1371/journal.pgen.1004773 (PMC4230839; doi:10.1371/journal.pgen.1004773)
Supplement: Figure S9 — Forward and reverse sequence alignments of 56 CAZyme genes cloned into pET-22B(+) confirm that genes had the correct sequences. (PDF) [file pgen.1004773.s009.pdf]

Forward and reverse sequence alignments of 56 CAZy with correct sequences that were transformed into BL21 cells for enzyme purification

cphy0218_1

Query= HR0ADKBY78ZA01_CAZY1C1P16_1.SCF
>Cphy0218 forward

```
Query: 67  aaagaaggagataggatcATGaagttcagtaatggatgttggttacaaaagaaaggtact 126
           ||||||||||||||||||||||||||||||||||||||||||||||||||||||||||||
Sbjct: 1   aaagaaggagataggatcatgaagttcagtaatggatgttggttacaaaagaaaggtact 60


Query: 127 gaatgtttttcaccagtgcaggtttatgattatcaaatcaaagaaaacgaggtaagaata 186
           ||||||||||||||||||||||||||||||||||||||||||||||||||||||||||||
Sbjct: 61  gaatgtttttcaccagtgcaggtttatgattatcaaatcaaagaaaacgaggtaagaata 120


Query: 187 cttaccacaacacatcaaatcaatcaccgcggtgataccttaggtggcgtgaatcttacg 246
           ||||||||||||||||||||||||||||||||||||||||||||||||||||||||||||
Sbjct: 121 cttaccacaacacatcaaatcaatcaccgcggtgataccttaggtggcgtgaatcttacg 180


Query: 247 atatttattacagcaccggcaccggaagttttgcgtgttaagacttatcactatatggga 306
           ||||||||||||||||||||||||||||||||||||||||||||||||||||||||||||
Sbjct: 181 atatttattacagcaccggcaccggaagttttgcgtgttaagacttatcactatatggga 240


Query: 307 gttagaaagaagagtcctgagtttgagttagacttatcaggggcttgtacactagagtgt 366
           ||||||||||||||||||||||||||||||||||||||||||||||||||||||||||||
Sbjct: 241 gttagaaagaagagtcctgagtttgagttagacttatcaggggcttgtacactagagtgt 300


Query: 367 gaggacacagaggatattttaataattaaaaatggaagtcttcgtttggaagtacaaaaa 426
           ||||||||||||||||||||||||||||||||||||||||||||||||||||||||||||
Sbjct: 301 gaggacacagaggatattttaataattaaaaatggaagtcttcgtttggaagtacaaaaa 360


Query: 427 tcaaacgcagcttttgcttattatcgtgggaatgagaaattaacctccagcggttggcga 486
           ||||||||||||||||||||||||||||||||||||||||||||||||||||||||||||
Sbjct: 361 tcaaacgcagcttttgcttattatcgtgggaatgagaaattaacctccagcggttggcga 420


Query: 487 gatctagcctatatgaaaacagattggcaagggcttgcttacgatgatggtggtgaagag 546
           ||||||||||||||||||||||||||||||||||||||||||||||||||||||||||||
Sbjct: 421 gatctagcctatatgaaaacagattggcaagggcttgcttacgatgatggtggtgaagag 480


Query: 547 gatacttacatgagagagcaattaacgctttctgtaggtgaacttgtttatggacttggt 606
           ||||||||||||||||||||||||||||||||||||||||||||||||||||||||||||
Sbjct: 481 gatacttacatgagagagcaattaacgctttctgtaggtgaacttgtttatggacttggt 540


Query: 607 gagagatttacaccgtttgtaaaaaatggtcaatctcttgatatctggaatgaagatggc 666
           ||||||||||||||||||||||||||||||||||||||||||||||||||||||||||||
Sbjct: 541 gagagatttacaccgtttgtaaaaaatggtcaatctcttgatatctggaatgaagatggc 600
```

```
Query: 667 ggtacctctaccgaacaatcttataagaacattcca-tttacataacaaacaaaaggcat 725
           |||||||||||||||||||||||||||||||||||| ||||||||||||||||| |  ||
Sbjct: 601 ggtacctctaccgaacaatcttataagaacattccatttacataacaaacaaaggctat 660


Query: 726 ggtgtctttgttaatcatcccgaaaaggtttcatttgaaaattggctctgaaaaggtgaa 785
           |||||||||||||||||||| || ||||||||||||| ||||||||||||| | |||| |
Sbjct: 661 ggtgtctttgttaatcatccggagaaggtttcatttg-aaattggctctgagatggtg-a 718


Query: 786 ccaaagtaggcttttcagtacctgggaaaatgcctcga 823
           ||||||||||||||||||||||| ||| ||||||||||
Sbjct: 719 ccaaagtaggcttttcagtacct-ggagaatgcctcga 755


Query= HR0ADKBY78ZA01_CAZY1C1P17_1.SCF

>Cphy0218 reverse

Query: 67   gtgtaatggatagtgatcTTAatggtgatggtgatgatgagaaattgcaactatagcaac 126
            ||||||||||||||||||||||||||||||||||||||||||||||||||||||||||||
Sbjct: 2397 gtgtaatggatagtgatcttaatggtgatggtgatgatgagaaattgcaactatagcaac 2338


Query: 127  cacagattcctgtgccaaaataatcgtatcgtttccatccactttaaatgaaacaccttc 186
            ||||||||||||||||||||||||||||||||||||||||||||||||||||||||||||
Sbjct: 2337 cacagattcctgtgccaaaataatcgtatcgtttccatccactttaaatgaaacaccttc 2278


Query: 187  tacgctatttacgacatagttctttaagacaatacttaccggtacttttgatgtaagatt 246
            ||||||||||||||||||||||||||||||||||||||||||||||||||||||||||||
Sbjct: 2277 tacgctatttacgacatagttctttaagacaatacttaccggtacttttgatgtaagatt 2218


Query: 247  tattgttatcttaccaccatttaatactgcactcattactaccgccttttctccggtttc 306
            ||||||||||||||||||||||||||||||||||||||||||||||||||||||||||||
Sbjct: 2217 tattgttatcttaccaccatttaatactgcactcattactaccgccttttctccggtttc 2158


Query: 307  ttgataaacagtagttgctacctctaccccctcttgtaactcatatattttaactcaag 366
            ||||||||||||||||||||||||||||||||||||||||||||||||||||||||||||
Sbjct: 2157 ttgataaacagtagttgctacctctaccccctcttgtaactcatatattttaactcaag 2098


Query: 367  atccttcgtatactcatagactgctccttgacctttcggaccagaggcaatgatgctacc 426
            ||||||||||||||||||||||||||||||||||||||||||||||||||||||||||||
Sbjct: 2097 atccttcgtatactcatagactgctccttgacctttcggaccagaggcaatgatgctacc 2038


Query: 427  ttgttttacatagagtggtatgcttaggtaactatgttcttcttcataccatctcccacc 486
            ||||||||||||||||||||||||||||||||||||||||||||||||||||||||||||
Sbjct: 2037 ttgttttacatagagtggtatgcttaggtaactatgttcttcttcataccatctcccacc 1978


Query: 487  ctcttttacttcaccggtcagataattggtccatttccccttaggtaagtaataatgagc 546
            ||||||||||||||||||||||||||||||||||||||||||||||||||||||||||||
Sbjct: 1977 ctcttttacttcaccggtcagataattggtccatttccccttaggtaagtaataatgagc 1918
```

```
Query: 547  catactattctcattaaatatcggagcaactagtaaactgtcgccgagaaaatactgctt 606
            ||||||||||||||||||||||||||||||||||||||||||||||||||||||||||||
Sbjct: 1917 catactattctcattaaatatcggagcaactagtaaactgtcgccgagaaaatactgctt 1858


Query: 607  atctaggtaagagcaatttggatctttggtatattccatcaccatacttctcatcattgg 666
            ||||||||||||||||||||||||||||||||||||||||||||||||||||||||||||
Sbjct: 1857 atctaggtaagagcaatttggatctttggtatattccatcaccatacttctcatcattgg 1798


Query: 667  aatacctgattctgaggtctccacagcattacgataaaggtatggcataagggaaccttt 726
            ||||||||||||||||||||||||||||||||||||||||||||||||||||||||||||
Sbjct: 1797 aatacctgattctgaggtctccacagcattacgataaaggtatggcataagggaaccttt 1738


Query: 727  taactcagtaaagaaacgaaccacatcgactgcttcttcatcatacgcccaaggaacacg 786
            ||||||||||||||||||||||||||||||||||||||||||||||||||||||||||||
Sbjct: 1737 taactcagtaaagaaacgaaccacatcgactgcttcttcatcatacgcccaaggaacacg 1678


Query: 787  atacgaagtactaccatgaagtctggaatgggtggataat-aaccaaaagctgcccagcg 845
            |||||||||||||||||||||||||||||||||||||||| |||||||||||||||||||
Sbjct: 1677 atacgaagtactaccatgaagtctggaatgggtggataataaaccaaaagctgcccagcg 1618


Query: 846  cttatatacatccggtgttgaagtactctcaatccctccgatatcatgaatccaataccc 905
            ||||||||||||||||||||||||||||||||  ||||||||||||||| ||||||||||
Sbjct: 1617 cttatatacatccggtgttgaagtactctcaaatcctccgatatcatgactccaataccc 1558


Query: 906  aaaatccagacattgttaaaggataaaccctccgcgaaga 945
             |||||||||||| ||| |||||| || ||||||||||||
Sbjct: 1557 -aaatccagacatcgtt-aaggat-aagcctccgcgaaga 1521

#######

# cphy1071_1

Query= HR0ADKBY78ZD01_CAZY2C1P16_1.SCF

>Cphy1071* forward

Query: 72  agaaggagataggatcATGgagaaattttcaacgaaattagaagcagagaatggaaagat 131
           ||||||||||||||||||||||||||||||||||||||||||||||||||||||||||||
Sbjct: 3   agaaggagataggatcatggagaaattttcaacgaaattagaagcagagaatggaaagat 62


Query: 132 gactggtactgtaacggtagccactacaaggccagggtatagtggaactggatatgtcac 191
           ||||||||||||||||||||||||||||||||||||||||||||||||||||||||||||
Sbjct: 63  gactggtactgtaacggtagccactacaaggccagggtatagtggaactggatatgtcac 122


Query: 192 cggttttactcaaaacagcagtaattcctggtcaacttctgtagtgattccgacaactgg 251
           ||||||||||||||||||||||||||||||||||||||||||||||||||||||||||||
Sbjct: 123 cggttttactcaaaacagcagtaattcctggtcaacttctgtagtgattccgacaactgg 182
```

```
Query: 252 tcactacacatttacaatccgaagtgctgcagatagttataaagaaaattatctttatat 311
           ||||||||||||||||||||||||||||||||||||||||||||||||||||||||||||
Sbjct: 183 tcactacacatttacaatccgaagtgctgcagatagttataaagaaaattatctttatat 242


Query: 312 taatggtacacaagctggaattatttatagtacaggtgatggtaaatggggagatactgt 371
           ||||||||||||||||||||||||||||||||||||||||||||||||||||||||||||
Sbjct: 243 taatggtacacaagctggaattatttatagtacaggtgatggtaaatggggagatactgt 302


Query: 372 catcgaaagcgtatacttagaggctggaactatcactttatccgtaaaggaaaattgggg 431
           ||||||||||||||||||||||||||||||||||||||||||||||||||||||||||||
Sbjct: 303 catcgaaagcgtatacttagaggctggaactatcactttatccgtaaaggaaaattgggg 362


Query: 432 ttggtttgacctaaattacattgcaattgaggcgggaagtggtgttgataactctgttta 491
           ||||||||||||| ||||||||||||||||||||||||||||||||||||||||||||||
Sbjct: 363 ttggtttgacctagattacattgcaattgaggcgggaagtggtgttgataactctgttta 422


Query: 492 tcagaaggcaacgtctactctagtaaacccgaatgcaaatgaaaaaacaaagaatattat 551
           ||||||||||||||||||||||||||||||||||||||||||||||||||||||||||||
Sbjct: 423 tcagaaggcaacgtctactctagtaaacccgaatgcaaatgaaaaaacaaagaatattat 482


Query: 552 ggcatatctaaagagtatctacgggaaaaagactttagcaggtcagtcctgtacgttaaa 611
           ||||||||||||||||||||||||||||||||||||||||||||||||||||||||||||
Sbjct: 483 ggcatatctaaagagtatctacgggaaaaagactttagcaggtcagtcctgtacgttaaa 542


Query: 612 cgccagcactgaaatttgaagcacttttataaagcaactggaaaaatatcctgcaattcc 671
           ||| |||||||||| ||||||||| |||||||||||||||| |||||||||||||||||
Sbjct: 543 cgcaagcactgaaa-ttgaagcac-tttataaagcaactgg-aaaatatcctgcaattcg 599


Query: 672 tatgcttgatttaatcttttgttctccagcaagctactggtattccacaggaaaagttga 731
           |||||||||||||||||||||||||||||||||| ||||| ||||||||||| |||||||
Sbjct: 600 tatgcttgatttaatcttttgttctccagcaagcgactggcattccacaggagaagttga 659


Query: 732 tttggcccttgactgggatnnnnnnnngaggactaacgacatttcaatggccattgggca 791
           |||||| ||||||||||||        ||||||||||||||||||| ||| |||| ||||
Sbjct: 660 tttggcacttgactgggat-aaaaaaaggaggactaacgacatttcagtgg-catt-ggca 716


Query: 792 tgctccaaaaggtggtgc 809
           ||||||||||||||||||
Sbjct: 717 tgctccaaaaggtggtgc 734

Query= HR0ADKBY78ZD01_CAZY2C1P17_1.SCF
>Cphy1071* reverse


Query: 67   gtgtaatggatagtgatcTTAatggtgatggtgatgatgtggctccacacctagtaccaa 126
             ||||||||||||||||||||||||||||||||||||||||||||||||||||||||||||
Sbjct: 2109 gtgtaatggatagtgatcttaatggtgatggtgatgatgtggctccacacctagtaccaa 2050
```

```
Query: 127  cttaccattatagtacacaacaacttttgtactatctccataagaataatcattttcttg 186
            ||||||||||||||||||||||||||||||||||||||||||||||||||||||||||||
Sbjct: 2049 cttaccattatagtacacaacaacttttgtactatctccataagaataatcattttcttg 1990


Query: 187  gttatacaagctccaatcactcttcgtaaatctgatattcactataaaggtagaatccgc 246
            ||||||||||||||||||||||||||||||||||||||||||||||||||||||||||||
Sbjct: 1989 gttatacaagctccaatcactcttcgtaaatctgatattcactataaaggtagaatccgc 1930


Query: 247  ggataatttatcagttgtgttaaacgatacatcaagatacgaatctgcttttgtttttgg 306
            ||||||||||||||||||||||||||||||||||||||||||||||||||||||||||||
Sbjct: 1929 ggataatttatcagttgtgttaaacgatacatcaagatacgaatctgcttttgtttttgg 1870


Query: 307  taccgccattgagttaaaggttccattaactaccgatgtatatggaatgtaccatggatc 366
            ||||||||||||||||||||||||||||||||||||||||||||||||||||||||||||
Sbjct: 1869 taccgccattgagttaaaggttccattaactaccgatgtatatggaatgtaccatggatc 1810


Query: 367  tttatttaattgcattgcagcagtatcacaatagaagttctccttcgcagttccctcctt 426
            ||||||||||||||||||||||||||||||||||||||||||||||||||||||||||||
Sbjct: 1809 tttatttaattgcattgcagcagtatcacaatagaagttctccttcgcagttccctcctt 1750


Query: 427  cgtataataataacgaatcactagcttagataaatctatatcgcttcctttctcatgttt 486
            ||||||||||||||||||||||||||||||||||||||||||||||||||||||||||||
Sbjct: 1749 cgtataataataacgaatcactagcttagataaatctatatcgcttcctttctcatgttt 1690


Query: 487  aatcttaaaatcagcatttattgtgtttgttgatagattgttattacctgtattggttaa 546
            ||||||||||||||||||||||||||||||||||||||||||||||||||||||||||||
Sbjct: 1689 aatcttaaaatcagcatttattgtgtttgttgatagattgttattacctgtattggttaa 1630


Query: 547  agaaagagtaaaactacctggaattggtgttactgatggtgtaaccgttggtgtaggtgt 606
            ||||||||||||||||||||||||||||||||||||||||||||||||||||||||||||
Sbjct: 1629 agaaagagtaaaactacctggaattggtgttactgatggtgtaaccgttggtgtaggtgt 1570


Query: 607  tactgttggcttaactgttggtgtcggtgttgctgtcggtgttgctgttggcgttaccgt 666
            ||||||||||||||||||||||||||||||||||||||||||||||||||||||||||||
Sbjct: 1569 tactgttggcttaactgttggtgtcggtgttgctgtcggtgttgctgttggcgttaccgt 1510


Query: 667  tggcttaactgttggcgtcagtgttactgttggcgttactgtaagcttaacctgttggtg 726
            ||||||||||||||||||| ||||||||||||||||||||||| |||||| |||||||||
Sbjct: 1509 tggcttaactgttggcgtcggtgttactgttggcgttactgtaggcttaa-ctgttggtg 1451


Query: 727  ttggtgttggtgttaccgttggagttgttccattaaaaatttaggtagtttaacccaagt 786
            ||||||||||||||||||||||||||||||||||||||  |||||||||||| || | ||
Sbjct: 1450 ttggtgttggtgttaccgttggagttgttccattaaaa--ttaggtagtttatccaaggt 1393


Query: 787  taacacctccttacggttataaactttttacaccataaaagcctcagtatatgttccatt 846
            || |||||| |||| ||||||||| |||| ||||||| ||||||||||||||||||||||
```

```
Sbjct: 1392 tatcacctcattactgttataaaccttttcaccatagaagcctcagtatatgttccatt 1333


Query: 847  taggttgcccgaataaatcgataaagaaaatcccataacatactggacaccaaaaccaga 906
             |||||||||||||| ||||||||| ||||   ||||| ||||||| | |||| |||||||
Sbjct: 1332 taggttgcccgaat-aatcgataaggaaatctccataccatactgcaaaccataaccaga 1274


Query: 907  agg 909
             |||
Sbjct: 1273 agg 1271

#######

# Cphy1510_2

Query= HR0ADKBY78ZG04_CAZY11C2P16_1.SCF
>Cphy1510* forward


Query: 68  aaagaaggagataggatcATGaccaataagacctatgattttaattcgatgacttatcaa 127
            ||||||||||||||||||||||||||||||||||||||||||||||||||||||||||||
Sbjct: 1   aaagaaggagataggatcatgaccaataagacctatgattttaattcgatgacttatcaa 60


Query: 128 tccacatggggagttacatattctatcagtaatggatcaggaacatttaatttcactggt 187
            ||||||||||||||||||||||||||||||||||||||||||||||||||||||||||||
Sbjct: 61  tccacatggggagttacatattctatcagtaatggatcaggaacatttaatttcactggt 120


Query: 188 caataccgtgaaattaagttcaatcttccggaaacgctagatatgtctcaatgtactagt 247
            ||||||||||||||||||||||||||||||||||||||||||||||||||||||||||||
Sbjct: 121 caataccgtgaaattaagttcaatcttccggaaacgctagatatgtctcaatgtactagt 180


Query: 248 gtaacattcaatgcttccagtccaaatggacagattgcatttaagctttacgatacttct 307
            ||||||||||||||||||||||||||||||||||||||||||||||||||||||||||||
Sbjct: 181 gtaacattcaatgcttccagtccaaatggacagattgcatttaagctttacgatacttct 240


Query: 308 ggaaatcaggtggctgtagtgtataactttaattccaatacctcagactgtaccttcgca 367
            ||||||||||||||||||||||||||||||||||||||||||||||||||||||||||||
Sbjct: 241 ggaaatcaggtggctgtagtgtataactttaattccaatacctcagactgtaccttcgca 300


Query: 368 ccaaatagtacggcaaaggtaaacagtattggaataatggcgcaagggacaaataactac 427
            ||||||||||||||||||||||||||||||||||||||||||||||||||||||||||||
Sbjct: 301 ccaaatagtacggcaaaggtaaacagtattggaataatggcgcaagggacaaataactac 360


Query: 428 tcagcagttgtgaatcgagttacatttacaatgacaggagggtcttctggcactggttct 487
            ||||||||||||||||||||||||||||||||||||||||||||||||||||||||||||
Sbjct: 361 tcagcagttgtgaatcgagttacatttacaatgacaggagggtcttctggcactggttct 420


Query: 488 tcaactttattaaacacttatggaaatatattaaaaaactctggaactgctgttaattta 547
            ||||||||||||||||||||||||||||||||||||||||||||||||||||||||||||
Sbjct: 421 tcaactttattaaacacttatggaaatatattaaaaaactctggaactgctgttaattta 480
```

```
Query: 548 agtcagctgcaaaattcaaatacactaagtgtgattaagacgcaatataatagtatcaca 607
           ||||||||||||||||||||||||||||||||||||||||||||||||||||||||||||
Sbjct: 481 agtcagctgcaaaattcaaatacactaagtgtgattaagacgcaatataatagtatcaca 540


Query: 608 ttagagaatgaaatgaagccagatgcagttcttggaagttcatcaacattaatgactgtt 667
           ||||||||||||||||||||||||||||||||||||||||||||||||||||||||||||
Sbjct: 541 ttagagaatgaaatgaagccagatgcagttcttggaagttcatcaacattaatgactgtt 600


Query: 668 gctcaagcaaaatcgaatggttattatattccttctagctacacggaaagtacagttcca 727
           ||||||||||||||||||||||||||||||||||||||||||||||||||||||||||||
Sbjct: 601 gctcaagcaaaatcgaatggttattatattccttctagctacacggaaagtacagttcca 660


Query: 728 actcttaaatttagtaccatcgatgcagttctacagatttgctacaataacgggctaaag 787
           ||||||||||||||||||||||||||||||||||||||||||||||||||||||||||||
Sbjct: 661 actcttaaatttagtaccatcgatgcagttctacagatttgctacaataacgggctaaag 720


Query: 788 cttagaggacatacattagtatggcattccccaacaccgggattggttctttagaacagg 847
           ||||||||||||||||||||||||||||||| |||||| |||||||||||||||||||||
Sbjct: 721 cttagaggacatacattagtatggcattcccaaacacc-ggattggttctttagaacagg 779


Query: 848 ttaaaggttctagtggatcgtatgttagccaagctgttaatggatgccaaaatggaaaat 907
           ||| | |||||||||||||||||||||||||||||||| |||||||| | ||||| ||||
Sbjct: 780 ttata-gttctagtggatcgtatgttagccaagctgtt-atggatgcaagaatgg-aaat 836


Query: 908 gtttaataagtcttatatga 927
           ||||| || |||||||||||
Sbjct: 837 gtttattaggtcttatatga 856

Query= HR0ADKBY78ZG04_CAZY11C2P17_1.SCF
>Cphy1510* reverse


Query: 66   gtgtaatggatagtgatcTTAatggtgatggtgatgatgatatcctgcatctgtaaatgc 125
            ||||||||||||||||||||||||||||||||||||||||||||||||||||||||||||
Sbjct: 1518 gtgtaatggatagtgatcttaatggtgatggtgatgatgatatcctgcatctgtaaatgc 1459


Query: 126  ttgtaacgctttattataagattcttttggagtagttaaattactgaataacaaaggatt 185
            ||||||||||||||||||||||||||||||||||||||||||||||||||||||||||||
Sbjct: 1458 ttgtaacgctttattataagattcttttggagtagttaaattactgaataacaaaggatt 1399


Query: 186  ttgggaggcacgccaagatacgctatcatatagtccccaccatgtaataccagtgatatt 245
            ||||||||||||||||||||||||||||||||||||||||||||||||||||||||||||
Sbjct: 1398 ttgggaggcacgccaagatacgctatcatatagtccccaccatgtaataccagtgatatt 1339


Query: 246  cccacctgctttctttagagaaagaatagcagtcatcaaatcatatacataagtagcttg 305
            ||||||||||||||||||||||||||||||||||||||||||||||||||||||||||||
Sbjct: 1338 cccacctgctttctttagagaaagaatagcagtcatcaaatcatatacataagtagcttg 1279
```

```
Query: 306  tgtggaagctgatgtattcgtaacatcaagttcagtaacctgtacttcatatcctgcatt 365
            ||||||||||||||||||||||||||||||||||||||||||||||||||||||||||||
Sbjct: 1278 tgtggaagctgatgtattcgtaacatcaagttcagtaacctgtacttcatatcctgcatt 1219


Query: 366  caagaacttctgcatagctgttttatatgccgatacagaaggataggaggtatttaagtg 425
            ||||||||||||||||||||||||||||||||||||||||||||||||||||||||||||
Sbjct: 1218 caagaacttctgcatagctgttttatatgccgatacagaaggataggaggtatttaagtg 1159


Query: 426  agactgcattccaacaccagcgcaaagtttcgtaccagagttaataaaatttactaatga 485
            ||||||||||||||||||||||||||||||||||||||||||||||||||||||||||||
Sbjct: 1158 agactgcattccaacaccagcgcaaagtttcgtaccagagttaataaaatttactaatga 1099


Query: 486  taggatctgatctgtaacctcatatgtgttgtaatcattataaaacaattttactgaatt 545
            ||||||||||||||||||||||||||||||||||||||||||||||||||||||||||||
Sbjct: 1098 taggatctgatctgtaacctcatatgtgttgtaatcattataaaacaattttactgaatt 1039


Query: 546  cgttaatccaaaactgctaagacaatcatacgcatactggaaagctttctttacataaga 605
            ||||||||||||||||||||||||||||||||||||||||||||||||||||||||||||
Sbjct: 1038 cgttaatccaaaactgctaagacaatcatacgcatactggaaagctttctttacataaga 979


Query: 606  tggtgtggtaccaaggttggatccataaacttgagaccatccagaggtagaagcatgcaa 665
            ||||||||||||||||||||||||||||||||||||||||||||||||||||||||||||
Sbjct: 978  tggtgtggtaccaaggttggatccataaacttgagaccatccagaggtagaagcatgcaa 919


Query: 666  atactcatttacaacatcccaagcatatactacacttccatagcttccattataaatatg 725
            ||||||||||||||||||||||||||||||||||||||||||||||||||||||||||||
Sbjct: 918  atactcatttacaacatcccaagcatatactacacttccatagcttccattataaatatg 859


Query: 726  actcatataagacctaataaacatttccattcttgcatccataacagcttggctaacata 785
            ||||||||||||||||||||||||||||||||||||||||||||||||||||||||||||
Sbjct: 858  actcatataagacctaataaacatttccattcttgcatccataacagcttggctaacata 799


Query: 786  cgatccactagaactataacctgttctaaagaaccaatccggtgtttgggaatgccatac 845
            ||||||||||||||||||||||||||||||||||||||||||||||||||||||||||||
Sbjct: 798  cgatccactagaactataacctgttctaaagaaccaatccggtgtttgggaatgccatac 739


Query: 846  taatgtatgtcctctaagctttaaccccgttattgtaacaaatctgtagaactgcatcga 905
            |||||||||||||||||||||| | |||||||||||| ||||||||||||||||||||||
Sbjct: 738  taatgtatgtcctctaagcttt-agcccgttattgtagcaaatctgtagaactgcatcga 680


Query: 906  tggtactaaatttaagagttggaactgtactttcc 940
            |||||||||||||||||||||||||||||||||||
Sbjct: 679  tggtactaaatttaagagttggaactgtactttcc 645
```

#######

```
# cphy1713_1

Query= HR0ADKBY78ZG01_CAZY3C1P16_1.SCF
>Cphy1713* forward


Query: 75  aaagaaggagataggatcATGtcttccgctgtttctgcaattttggaggaggccctttt 134
           ||||||||||||||||||||||||||||||||||||||||||||||||||||||||||||
Sbjct: 1   aaagaaggagataggatcatgtcttccgctgtttctgcaattttggaggaggccctttt 60


Query: 135 gttacaggcggtcaaccagtaatgaatcaattaaaagcttctggttttaacacagtaatt 194
           ||||||||||||||||||||||||||||||||||||||||||||||||||||||||||||
Sbjct: 61  gttacaggcggtcaaccagtaatgaatcaattaaaagcttctggttttaacacagtaatt 120


Query: 195 atctggtcagtacatcctcatccgaatggagacttatacatcaacgatgaacttgtatgt 254
           ||||||||||||||||||||||||||||||||||||||||||||||||||||||||||||
Sbjct: 121 atctggtcagtacatcctcatccgaatggagacttatacatcaacgatgaacttgtatgt 180


Query: 255 caaaacggtaattacgtaggagaccctgcatgggctactgcatggcaatccttaaaaact 314
           ||||||||||||||||||||||||||||||||||||||||||||||||||||||||||||
Sbjct: 181 caaaacggtaattacgtaggagaccctgcatgggctactgcatggcaatccttaaaaact 240


Query: 315 ggtaccacctctgtcactcgcgttgagataagcattggagcttggggatgcagtgatttt 374
           ||||||||||||||||||||||||||||||||||||||||||||||||||||||||||||
Sbjct: 241 ggtaccacctctgtcactcgcgttgagataagcattggagcttggggatgcagtgatttt 300


Query: 375 gagaatattcaggcattaattaatgcaaatggtaccggttcaaatacaatactatatcgt 434
           ||||||||||||||||||||||||||||||||||||||||||||||||||||||||||||
Sbjct: 301 gagaatattcaggcattaattaatgcaaatggtaccggttcaaatacaatactatatcgt 360


Query: 435 aatttttagctctgaaaaatgctactggcgctgatgcggtaaatttcgatgatgaatcc 494
           ||||||||||||||||||||||||||||||||||||||||||||||||||||||||||||
Sbjct: 361 aatttttagctctgaaaaatgctactggcgctgatgcggtaaatttcgatgatgaatcc 420


Query: 495 ttatacaatgtaaatacaacggtacaatttggacaaatgtgtgctgctatgggcatgaag 554
           ||||||||||||||||||||||||||||||||||||||||||||||||||||||||||||
Sbjct: 421 ttatacaatgtaaatacaacggtacaatttggacaaatgtgtgctgctatgggcatgaag 480


Query: 555 gtaacattatgtccctacacttcctcaagcttttggagtagtgtaaaatcccaacttggt 614
           ||||||||||||||||||||||||||||||||||||||||||||||||||||||||||||
Sbjct: 481 gtaacattatgtccctacacttcctcaagcttttggagtagtgtaaaatcccaacttggt 540


Query: 615 tccattgttgatcgtgtgtacttgcaatgctacgatggcggagcaggtaatagcccttct 674
           ||||||||||||||||||||||||||||||||||||||||||||||||||||||||||||
Sbjct: 541 tccattgttgatcgtgtgtacttgcaatgctacgatggcggagcaggtaatagcccttct 600


Query: 675 tcctggaaatccatcatgggcacgaatgtgattcccggactctggtgcttgcataccgga 734
           ||||||||||||||||||||||||||||||||||||||||||||||||||||||| ||||
```

```
Sbjct: 601 tcctggaaatccatcatgggcacgaatgtgattcccggactctggtgcttgcatagcgga 660


Query: 735 agtcaaggagactctgctaactcggttaaatcaaaccttaccaattggaagtcctcctcc 794
           ||||||||||||||||||| ||||||||||||||| ||||||||||||||||||||||||
Sbjct: 661 agtcaaggagactctgctagctcggttaaatcaaagcttaccaattggaagtcctcctcc 720


Query: 795 gaaggtggatttttctggctatatgaagatttaatgaaattatcaagtcccaatttcaac 854
           |||||||||||| ||||||||||||| ||||||||||| ||||||||| |||| ||||||
Sbjct: 721 gaaggtggatttatctggctatatgacgatttaatgaagttatcaagt-ccaaattcaac 779


Query: 855 acccaaacatgcgaatgcaattaaattcattatttaccacta 896
           | || |  |||||||||||||| |||||| ||||||||||||
Sbjct: 780 agccgactatgcgaatgcaatt-aattcagtatttaccacta 820

Query= HR0ADKBY78ZG01_CAZY3C1P17_1.SCF

>Cphy1713* reverse


Query: 73   gtgtaatggatagtgatcTTAatggtgatggtgatgatgatataattcaaactcataaat 132
            ||||||||||||||||||||||||||||||||||||||||||||||||||||||||||||
Sbjct: 1314 gtgtaatggatagtgatcttaatggtgatggtgatgatgatataattcaaactcataaat 1255


Query: 133  cctagcaatattggaagtagtttgcgttccggcagtaatgtataagcgaacgtaacgggc 192
            ||||||||||||||||||||||||||||||||||||||||||||||||||||||||||||
Sbjct: 1254 cctagcaatattggaagtagtttgcgttccggcagtaatgtataagcgaacgtaacgggc 1195


Query: 193  actaaaagttattacagtacgatctgtaatattggaagtgttatttgttacggtatccac 252
            ||||||||||||||||||||||||||||||||||||||||||||||||||||||||||||
Sbjct: 1194 actaaaagttattacagtacgatctgtaatattggaagtgttatttgttacggtatccac 1135


Query: 253  atcagtccaattgatgccatccgtgctttttgtaacttaaaatccttggtattataaga 312
            ||||||||||||||||||||||||||||||||||||||||||||||||||||||||||||
Sbjct: 1134 atcagtccaattgatgccatccgtgctttttgtaacttaaaatccttggtattataaga 1075


Query: 313  ggtgctctcgcccccaacaccggcatgttgtacaacccatcttgatatgttataggtttg 372
            ||||||||||||||||||||||||||||||||||||||||||||||||||||||||||||
Sbjct: 1074 ggtgctctcgcccccaacaccggcatgttgtacaacccatcttgatatgttataggtttg 1015


Query: 373  acctaagtcgattttaaccatttaacagcatcagagttatcagagcaccatttactatt 432
            ||||||||||||||||||||||||||||||||||||||||||||||||||||||||||||
Sbjct: 1014 acctaagtcgattttaaccatttaacagcatcagagttatcagagcaccatttactatt 955


Query: 433  accatagccatagcctgcaatagaaccgtctactgctttgtccggtgtttcatttgttac 492
            ||||||||||||||||||||||||||||||||||||||||||||||||||||||||||||
Sbjct: 954  accatagccatagcctgcaatagaaccgtctactgctttgtccggtgtttcatttgttac 895


Query: 493  atactgatttgctgttgctgatttattgagagcaatattatttgaagttggtggtgttgg 552
```

```
            ||||||||||||||||||||||||||||||||||||||||||||||||||||||||||||
Sbjct: 894  atactgatttgctgttgctgatttattgagagcaatattatttgaagttggtggtgttgg 835


Query: 553  tgtaggcgtaggtgtagtggtaaatactgaattaattgcattcgcatagtcggctgttga 612
            ||||||||||||||||||||||||||||||||||||||||||||||||||||||||||||
Sbjct: 834  tgtaggcgtaggtgtagtggtaaatactgaattaattgcattcgcatagtcggctgttga 775


Query: 613  atttggacttgataacttcattaaatcgtcatatagccagataaatccaccttcggagga 672
            ||||||||||||||||||||||||||||||||||||||||||||||||||||||||||||
Sbjct: 774  atttggacttgataacttcattaaatcgtcatatagccagataaatccaccttcggagga 715


Query: 673  ggacttccaattggtaagctttgatttaaccgagctagcagagtctccttgacttccgct 732
            ||||||||||||||||||||||||||||||||||||||||||||||||||||||||||||
Sbjct: 714  ggacttccaattggtaagctttgatttaaccgagctagcagagtctccttgacttccgct 655


Query: 733  atgcaagcaccagagtccgggaatcacattcgtgcccatgatggatttccaggaagaagg 792
            ||||||||||||||||||||||||||||||||||||||||||||||||||||||||||||
Sbjct: 654  atgcaagcaccagagtccgggaatcacattcgtgcccatgatggatttccaggaagaagg 595


Query: 793  gctattacctgctccgccatcgtagcattgcaagtacacacgatcaacaatgggaacaaa 852
            ||||||||||||||||||||||||||||||||||||||||||||||||||| ||||| ||
Sbjct: 594  gctattacctgctccgccatcgtagcattgcaagtacacacgatcaacaat-ggaaccaa 536


Query: 853  gttgggaatttacactacttccaaaagcttgaggaaagtgtagggacataatggttacct 912
            ||||||| |||||||||| ||||||||||||||| ||||||||||||||||| |||||||
Sbjct: 535  gttgggattttacactac-tccaaaagcttgagg-aagtgtagggacataat-gttacct 479


Query: 913  tcatgccccatagcaccacacattttgtccaaattgtaccgttgtatttacattggataa 972
            ||||| ||||||||| |||||| |||||||||||||||||||||||||||||||| ||||
Sbjct: 478  tcatg-cccatagcagcacaca-tttgtccaaattgtaccgttgtatttacattgtataa 421


Query: 973  gg 974
            ||
Sbjct: 420  gg 419


#######

# cphy2108_1

Query= HR0ADKBY78ZB02_CAZY4C1P16_1.SCF

>Cphy2108* forward


Query: 68  aaagaaggagataggatcATGgaaaccaacatctataaagtagattggagcaaatttaat 127
           ||||||||||||||||||||||||||||||||||||||||||||||||||||||||||||
Sbjct: 1   aaagaaggagataggatcatggaaaccaacatctataaagtagattggagcaaatttaat 60
```

```
Query: 128 gagggtgacaaaatcagcggtcccatggagggtttaggtagatcaggcggagcagatatc 187
           ||||||||||||||||||||||||||||||||||||||||||||||||||||||||||||
Sbjct: 61  gagggtgacaaaatcagcggtcccatggagggtttaggtagatcaggcggagcagatatc 120


Query: 188 acggttacgggctcctctaccaaatcattttacatatctaatcgtaaagataactgggat 247
           ||||||||||||||||||||||||||||||||||||||||||||||||||||||||||||
Sbjct: 121 acggttacgggctcctctaccaaatcattttacatatctaatcgtaaagataactgggat 180


Query: 248 gcacttgatattcagaacgatcttttgaagttagatcgagatgcaacctatgaaatcaca 307
           ||||||||||||||||||||||||||||||||||||||||||||||||||||||||||||
Sbjct: 181 gcacttgatattcagaacgatcttttgaagttagatcgagatgcaacctatgaaatcaca 240


Query: 308 gttaccggtcatgttgacagcaatgtagatactaagaatgctagtgtcaagcttggcggt 367
           ||||||||||||||||||||||||||||||||||||||||||||||||||||||||||||
Sbjct: 241 gttaccggtcatgttgacagcaatgtagatactaagaatgctagtgtcaagcttggcggt 300


Query: 368 gtaacaaggaaaacgggcgaggatgatggatacccagagttcaaaaaagagaaactacaa 427
           ||||||||||||||||||||||||||||||||||||||||||||||||||||||||||||
Sbjct: 301 gtaacaaggaaaacgggcgaggatgatggatacccagagttcaaaaaagagaaactacaa 360


Query: 428 tcgggcaagagttttgtactaacctatgaacttaaactttctgatcaaattccggacgct 487
           ||||||||||||||||||||||||||||||||||||||||||||||||||||||||||||
Sbjct: 361 tcgggcaagagttttgtactaacctatgaacttaaactttctgatcaaattccggacgct 420


Query: 488 tcaagaaatctgtgggtgctccgtgttcagaccgacgaaccaagcggaagtagggccgga 547
           ||||||||||||||||||||||||||||||||||||||||||||||||||||||||||||
Sbjct: 421 tcaagaaatctgtgggtgctccgtgttcagaccgacgaaccaagcggaagtagggccgga 480


Query: 548 gatcttgtaccgttctatgtggatgatatcgtgattattcaaacaaaggcatccactgct 607
           ||||||||||||||||||||||||||||||||||||||||||||||||||||||||||||
Sbjct: 481 gatcttgtaccgttctatgtggatgatatcgtgattattcaaacaaaggcatccactgct 540


Query: 608 cctgtagctgtgactggtgaccttatgtcactttacgaacttaatgcggacaaaactctc 667
           ||||||||||||||||||||||||||||||||||||||||||||||||||||||||||||
Sbjct: 541 cctgtagctgtgactggtgaccttatgtcactttacgaacttaatgcggacaaaactctc 600


Query: 668 aaggttggagaatcattatcaagtccagctctgaaagtttctggtaatgctaagatagtt 727
           ||||||||||||||||||||||||||||||||||||||||||||||||||||||||||||
Sbjct: 601 aaggttggagaatcattatcaagtccagctctgaaagtttctggtaatgctaagatagtt 660


Query: 728 gttgtagaaagcaccgatggtaccgtgtcattacaactgaaagaccgtgttaataattat 787
           ||||||||| ||||||||||||||||||||||||||||||||||||||||||||||||||
Sbjct: 661 gttgtagaaggcaccgatggtaccgtgtcattacaactgaaagaccgtgttaataattat 720


Query: 788 gacggtggaaacttccctcgtgatgcactgaaaataaccgataaatttatgtctggaaca 847
           ||||||| | || ||| |||||||||||||||||||| |||||||||||||||||| |||
Sbjct: 721 gacggtgtagacatccttcgtgatgcactgaaaataaacgataaatttatgtctggtaca 780
```

```
Query: 848 tccacgatttgaggtaaaaggccatgtaagaagatggtttctgatttaagttaattccca 907
           | ||||| ||||||||||||||||||| ||| ||||| |||||||||||| ||| |||||
Sbjct: 781 tacacga-ttgaggtaaaaggccatgt-agaggatgg-ttctgatttaag-taaatccca 836


Query: 908 gtttg 912
           |||||
Sbjct: 837 gtttg 841


Query= HR0ADKBY78ZB02_CAZY4C1P17_1.SCF   1289

>Cphy2108* reverse


Query: 66   gtgtaatggatagtgatcTTAatggtgatggtgatgatgtatattctttatattcttctc 125
            ||||||||||||||||||||||||||||||||||||||||||||||||||||||||||||
Sbjct: 2385 gtgtaatggatagtgatcttaatggtgatggtgatgatgtatattctttatattcttctc 2326


Query: 126  tcttcgttttgttaagatcgtaactggtacgaataccaatataatcagaatagctactgc 185
            ||||||||||||||||||||||||||||||||||||||||||||||||||||||||||||
Sbjct: 2325 tcttcgttttgttaagatcgtaactggtacgaataccaatataatcagaatagctactgc 2266


Query: 186  agtcaaaatcatttgcttactgctgtcttcccctgttttcgggatatctttaccaggtat 245
            ||||||||||||||||||||||||||||||||||||||||||||||||||||||||||||
Sbjct: 2265 agtcaaaatcatttgcttactgctgtcttcccctgttttcgggatatctttaccaggtat 2206


Query: 246  ggtttcaacagccttttcctctgctaccttttcctttgctacatcaattacagcaaagaa 305
            ||||||||||||||||||||||||||||||||||||||||||||||||||||||||||||
Sbjct: 2205 ggtttcaacagccttttcctctgctaccttttcctttgctacatcaattacagcaaagaa 2146


Query: 306  tgcgtcctttggagcaaaatttttatcaaacagtaatggataaccttggaaacgccagct 365
            ||||||||||||||||||||||||||||||||||||||||||||||||||||||||||||
Sbjct: 2145 tgcgtcctttggagcaaaatttttatcaaacagtaatggataaccttggaaacgccagct 2086


Query: 366  ttgaggatctgttttaccccagaaggtgacacgttcgatgttgtccgcatatttttttgta 425
            ||||||||||||||||||||||||||||||||||||||||||||||||||||||||||||
Sbjct: 2085 ttgaggatctgttttaccccagaaggtgacacgttcgatgttgtccgcatatttttttgta 2026


Query: 426  tacttcaaacaactgcttgtataaatccgcctgaagtttctcttcttcctttgtaggaga 485
            ||||||||||||||||||||||||||||||||||||||||||||||||||||||||||||
Sbjct: 2025 tacttcaaacaactgcttgtataaatccgcctgaagtttctcttcttcctttgtaggaga 1966


Query: 486  gtcagtacgttgcttgtaggtcatgtaatcgccatgaggaacatccagctcagatacact 545
            ||||||||||||||||||||||||||||||||||||||||||||||||||||||||||||
Sbjct: 1965 gtcagtacgttgcttgtaggtcatgtaatcgccatgaggaacatccagctcagatacact 1906


Query: 546  gatcttagcacctgtttttatgaaacgcttgatgctagcttctacagtggaaacctttaa 605
```

```
            ||||||||||||||||||||||||||||||||||||||||||||||||||||||||||||
Sbjct: 1905 gatcttagcacctgtttttatgaaacgcttgatgctagcttctacagtggaaacctttaa 1846


Query: 606  atctccggtccaataatgtgcctgcattccgattccttcgatgagcaatctgtcaggctc 665
            ||||||||||||||||||||||||||||||||||||||||||||||||||||||||||||
Sbjct: 1845 atctccggtccaataatgtgcctgcattccgattccttcgatgagcaatctgtcaggctc 1786


Query: 666  agtgttccgtttatcggtcttccactttgtgtttaattcttccaccatcaaggcgatcgc 725
            ||||||||||||||||||||||||||||||||||||||||||||||||||||||||||||
Sbjct: 1785 agtgttccgtttatcggtcttccactttgtgtttaattcttccaccatcaaggcgatcgc 1726


Query: 726  ttcacacttacctgcctctgtctcattgaagtcgttataatacagcactgcatcaagagc 785
            ||||||||||||||||||||||||||||||||||||||||||||||||||||||| ||||
Sbjct: 1725 ttcacacttacctgcctctgtctcattgaagtcgttataatacagcactgcatcaggagc 1666


Query: 786  ggcaagacgtgtaaacacgaaagcgtcatagatataatcagagccatcctcgcccttgct 845
            ||||||||||||||||||||||||||||||||||||||||||||||||||||||||||||
Sbjct: 1665 ggcaagacgtgtaaacacgaaagcgtcatagatataatcagagccatcctcgcccttgct 1606


Query: 846  cttttcagcaccgtttttataggcttgataccaaggaaaaccattttcgttatctttcaa 905
            ||| ||||||||||||| ||||||||||||||||||| |||||||| ||||||| |||||
Sbjct: 1605 cttatcagcaccgttttcataggcttgataccaaggagaaccatttccgttatc-ttcaa 1547


Query: 906  ttttgcgtaatacatccctccagccagcaaggaatttcggatacccctgg 955
             ||||||||||||||||||||||||||| ||| ||||||||||| |||||
Sbjct: 1546 atttgcgtaatacatccctccagccagc-agggatttcggatacacctgg 1498
```

#######

Cphy2632_2

Query= HR0ADKBY78ZB05_CAZY12C2P16_1.SCF

>Cphy2632 forward

```
Query: 69  aaagaaggagataggatcATGtggatagcagatttggaaaatggcaactataggaatcca 128
           ||||||||||||||||||||||||||||||||||||||||||||||||||||||||||||
Sbjct: 1   aaagaaggagataggatcatgtggatagcagatttggaaaatggcaactataggaatcca 60


Query: 129 ttactttatgcagattattctgatccagatgcaattagagttggtgacgattattttatg 188
           ||||||||||||||||||||||||||||||||||||||||||||||||||||||||||||
Sbjct: 61  ttactttatgcagattattctgatccagatgcaattagagttggtgacgattattttatg 120


Query: 189 gttgcatcaagtttttgcaatgcaccagctttacctttactacattctaaggatcttgtg 248
           ||||||||||||||||||||||||||||||||||||||||||||||||||||||||||||
Sbjct: 121 gttgcatcaagtttttgcaatgcaccagctttacctttactacattctaaggatcttgtg 180


Query: 249 aactggaaagtagtgaattatgtcttagataaaattccagagtttcgatatagaaatcca 308
```

```
           ||||||||||||||||||||||||||||||||||||||||||||||||||||||||||||
Sbjct: 181 aactggaaagtagtgaattatgtcttagataaaattccagagtttcgatatagaaatcca 240


Query: 309 atgcatggatgcggtgtatgggcaccttctatcagatatcatgaaggaacatattatgtg 368
           ||||||||||||||||||||||||||||||||||||||||||||||||||||||||||||
Sbjct: 241 atgcatggatgcggtgtatgggcaccttctatcagatatcatgaaggaacatattatgtg 300


Query: 369 tgtttcccaatgccagatgagggaatctacatgactacaaccaaagatccatttggcaag 428
           ||||||||||||||||||||||||||||||||||||||||||||||||||||||||||||
Sbjct: 301 tgtttcccaatgccagatgagggaatctacatgactacaaccaaagatccatttggcaag 360


Query: 429 tggagtgagcctgtgaatattagaccgggtgcaggctggattgatccatgtccattctgg 488
           ||||||||||||||||||||||||||||||||||||||||||||||||||||||||||||
Sbjct: 361 tggagtgagcctgtgaatattagaccgggtgcaggctggattgatccatgtccattctgg 420


Query: 489 gatgaagatggaaaagcgtatcttgttgcaggtgtagcaaagagtcgtataggatataaa 548
           ||||||||||||||||||||||||||||||||||||||||||||||||||||||||||||
Sbjct: 421 gatgaagatggaaaagcgtatcttgttgcaggtgtagcaaagagtcgtataggatataaa 480


Query: 549 agtgtccttcacatggtggagatgcaaccagatggtatgggacttatcggggaagatgtt 608
           ||||||||||||||||||||||||||||||||||||||||||||||||||||||||||||
Sbjct: 481 agtgtccttcacatggtggagatgcaaccagatggtatgggacttatcggggaagatgtt 540


Query: 609 agaatctttgatggcgataccaacggaaatgtaacaacagaaggtcctaagatgcataag 668
           ||||||||||||||||||||||||||||||||||||||||||||||||||||||||||||
Sbjct: 541 agaatctttgatggcgataccaacggaaatgtaacaacagaaggtcctaagatgcataag 600


Query: 669 aagaatggttggtattatgtcttttgcaccagcgggtggcgtaaagacaggttggcagtt 728
           ||||||||||||||||||||| ||||||||||||||||||||||||||||||||||||||
Sbjct: 601 aagaatggttggtattatgtc-tttgcaccagcgggtggcgtaaagacaggttggcagtt 659


Query: 729 atttcttcgttctaagaatgtatttggcccttacgaacaccaagttgtgatgaaacaagg 788
           | |||||||||||||||||||||||||||||||||||||| |||||||||||||||||||
Sbjct: 660 agttcttcgttctaagaatgtatttggcccttacgaacacaaagttgtgatgaaacaagg 719


Query: 789 ggatactcttggtaacggacctccatcaaggtgcttgggtt 829
           ||||||||||| |||||||||| ||||||||||||||||||
Sbjct: 720 ggatactcttgttaacggacct-catcaaggtgcttgggtt 759

Query= HR0ADKBY78ZB05_CAZY12C2P17_1.SCF   1306      0   1306  SCF

>Cphy2632 reverse


Query: 68   gtgtaatggatagtgatcTTAatggtgatggtgatgatgctgatatctaacgtagtctac 127
            ||||||||||||||||||||||||||||||||||||||||||||||||||||||||||||
Sbjct: 1686 gtgtaatggatagtgatcttaatggtgatggtgatgatgctgatatctaacgtagtctac 1627
```

```
Query: 128  ttctacataacctccaccattcatggtgttatcatggttacagaagacaccatttttaac 187
            ||||||||||||||||||||||||||||||||||||||||||||||||||||||||||||
Sbjct: 1626 ttctacataacctccaccattcatggtgttatcatggttacagaagacaccatttttaac 1567


Query: 188  acctacccatcttcctgctttagcattaatcgtaaatgcttccttaaactcttcaccgtc 247
            ||||||||||||||||||||||||||||||||||||||||||||||||||||||||||||
Sbjct: 1566 acctacccatcttcctgctttagcattaatcgtaaatgcttccttaaactcttcaccgtc 1507


Query: 248  aaggctataagacattgtgatagtctcctgaggaacatgctttacttgaatgtccatttc 307
            ||||||||||||||||||||||||||||||||||||||||||||||||||||||||||||
Sbjct: 1506 aaggctataagacattgtgatagtctcctgaggaacatgctttacttgaatgtccatttc 1447


Query: 308  catattatctacatatcctacgcactttacttcatatttaaggaatactgtcttttctct 367
            ||||||||||||||||||||||||||||||||||||||||||||||||||||||||||||
Sbjct: 1446 catattatctacatatcctacgcactttacttcatatttaaggaatactgtcttttctct 1387


Query: 368  atctttaaaatcactatctgttaatgaaatgcttactttattctggtccgcataggaaac 427
            ||||||||||||||||||||||||||||||||||||||||||||||||||||||||||||
Sbjct: 1386 atctttaaaatcactatctgttaatgaaatgcttactttattctggtccgcataggaaac 1327


Query: 428  atcacaatcaaagttttgaattcccgtaatcttctcgatcgcgtaagaaccattcttttt 487
            ||||||||||||||||||||||||||||||||||||||||||||||||||||||||||||
Sbjct: 1326 atcacaatcaaagttttgaattcccgtaatcttctcgatcgcgtaagaaccattcttttt 1267


Query: 488  gcttactgccatagcactatattccatccctaaggacacaattcctgcagtatctccatc 547
            ||||||||||||||||||||||||||||||||||||||||||||||||||||||||||||
Sbjct: 1266 gcttactgccatagcactatattccatccctaaggacacaattcctgcagtatctccatc 1207


Query: 548  ctgtaatcctgcaaaactcatcttagtgatacaagaaaattcaggcgctggccacttctg 607
            ||||||||||||||||||||||||||||||||||||||||||||||||||||||||||||
Sbjct: 1206 ctgtaatcctgcaaaactcatcttagtgatacaagaaaattcaggcgctggccacttctg 1147


Query: 608  taaaagaagattacgataatcgctaattggacgttgtggcacagaaggtactgcattcaa 667
            ||||||||||||||||||||||||||||||||||||||||||||||||||||||||||||
Sbjct: 1146 taaaagaagattacgataatcgctaattggacgttgtggcacagaaggtactgcattcaa 1087


Query: 668  tttgatttttgaattgcctaattcataccaatcaatgtctggattcgcgttccactgcca 727
            ||||||||||||||||||||||||||||||||||||||||||||||||||||||||||||
Sbjct: 1086 tttgatttttgaattgcctaattcataccaatcaatgtctggattcgcgttccactgcca 1027


Query: 728  ctgtaagccaaagctatctccttcaaattcatctgtagtatctggctcacaaaccggata 787
            ||||||||||| ||||||||||||||||||||||||||||||||||||||||||||||||
Sbjct: 1026 ctgtaagccaaggctatctccttcaaattcatctgtagtatctggctcacaaaccggata 967


Query: 788  taatggattattttgaagttctccctctgtcttacccgaccttaggcttctaatattccc 847
            |||||||||||||||||||||| ||||||||||| ||||| |||||||||| ||||| ||
Sbjct: 966  taatggattattttgaagttcttcctctgtctta-ccgacgttaggcttcttatatt-cc 909
```

```
Query: 848  attactggttcgccctaatcatttccctctttattcacaccaataattggccattctttt 907
            |||||||||||||| ||||||||||||||||||||||||||||||||||||||||| |||
Sbjct: 908  attactggttcgccgtaatcatttccctctttattcacaccaataattggccattcattt 849


Query: 908  tcccaatggcttggtttaaaaagaacgattctccccgcttccaacacatcttggaaaatg 967
            ||||||||  |||||| || | ||||||||||||| ||| |  ||||||||| |||||||
Sbjct: 848  tcccaatgcattggttgaagatgaacgattctccctgctgcatacacatctt-gaaaatg 790


Query: 968  taaaaacc 975
            ||||||||
Sbjct: 789  taaaaacc 782

######

# cphy2919_1

Query= HR0ADKBY78ZE02_CAZY5C1P16_1.SCF

>Cphy2919* forward


Query: 74  aaagaaggagataggatcATGactacatattattatgtttcaactacaggtagcgactcc 133
           ||||||||||||||||||||||||||||||||||||||||||||||||||||||||||||
Sbjct: 1   aaagaaggagataggatcatgactacatattattatgtttcaactacaggtagcgactcc 60


Query: 134 aataatggcactactaaggaaacagcttttaaaactcttacaaaagctttaactaaagct 193
           ||||||||||||||||||||||||||||||||||||||||||||||||||||||||||||
Sbjct: 61  aataatggcactactaaggaaacagcttttaaaactcttacaaaagctttaactaaagct 120


Query: 194 agtgcaggaacaacaatttttgttttaaatggaacttacagttattctacgacatttaaa 253
           ||||||||||||||||||||||||||||||||||||||||||||||||||||||||||||
Sbjct: 121 agtgcaggaacaacaatttttgttttaaatggaacttacagttattctacgacatttaaa 180


Query: 254 ctaactagtaatggtacggcttctgagccaataaaaatattaaactatagcggacatagc 313
           ||||||||||||||||||||||||||||||||||||||||||||||||||||||||||||
Sbjct: 181 ctaactagtaatggtacggcttctgagccaataaaaatattaaactatagcggacatagc 240


Query: 314 cctgttattgatttttctagtcaagaatatgcggatagttcaagaggttttcaaatatcg 373
           ||||||||||||||||||||||||||||||||||||||||||||||||||||||||||||
Sbjct: 241 cctgttattgatttttctagtcaagaatatgcggatagttcaagaggttttcaaatatcg 300


Query: 374 ggaaattattggattatcgctggtttaacgataactggtgctggggataatggtattcac 433
           ||||||||||||||||||||||||||||||||||||||||||||||||||||||||||||
Sbjct: 301 ggaaattattggattatcgctggtttaacgataactggtgctggggataatggtattcac 360


Query: 434 ataagtggaaactataatcgagtgcaagattgttttatcactaagtgcggagatactggt 493
           ||||||||||||||||||||||||||||||||||||||||||||||||||||||||||||
Sbjct: 361 ataagtggaaactataatcgagtgcaagattgttttatcactaagtgcggagatactggt 420
```

```
Query: 494 ttacaaatcagtaatggtggttcttataatacaattactcgtgttacttccacttacaat 553
           ||||||||||||||||||||||||||||||||||||||||||||||||||||||||||||
Sbjct: 421 ttacaaatcagtaatggtggttcttataatacaattactcgtgttacttccacttacaat 480


Query: 554 tatgataagaaaacgaatggtgagaatgcggatggttttgcagcaaaattaggtatcgga 613
           ||||||||||||||||||||||||||||||||||||||||||||||||||||||||||||
Sbjct: 481 tatgataagaaaacgaatggtgagaatgcggatggttttgcagcaaaattaggtatcgga 540


Query: 614 cctggaaatgtttttacttcctgtaaagcatacaataattccgacgatggatttgacttt 673
           ||||||||||||||||||||||||||||||||||||||||||||||||||||||||||||
Sbjct: 541 cctggaaatgtttttacttcctgtaaagcatacaataattccgacgatggatttgacttt 600


Query: 674 tatgatgcgaaaaatgcagtaaaggtatatgactgtgaagcatcctataacggagtaccg 733
           ||||||||||||||||||||||||||||||||||||||||||||||||||||||||| ||
Sbjct: 601 tatgatgcgaaaaatgcagtaaaggtatatgactgtgaagcatcctataacggagtagcg 660


Query: 734 gatggcaatggaaatggttttaaagtaggaggaaataactcagcggaatatcattatctt 793
           |||||||||||||||||||||||||||||||||||||||||||||||  |||||||||||
Sbjct: 661 gatggcaatggaaatggttttaaagtaggaggaaataactcagcggataatcattatctt 720


Query: 794 gaaaattgtactgccacaggaaatcgttctaaaggatataaccaaaaataacaataatgg 853
           ||||||||||||||||||||||||||||||| ||||||| ||| |||||||||||| |||
Sbjct: 721 gaaaattgtactgccacaggaaatcgttctagaggatatgacc-aaaataacaatactgg 779


Query: 854 ttatattacactagtaaattggactggt 881
           ||||||||||||||||||||| ||||||
Sbjct: 780 ttatattacactagtaaattgtactggt 807


Query= HR0ADKBY78ZE02_CAZY5C1P17_1.SCF

>Cphy2919* reverse


Query: 67  gtgtaatggatagtgatcTTAatggtgatggtgatgatgctgataaaatgaacagttcgt 126
           ||||||||||||||||||||||||||||||||||||||||||||||||||||||||||||
Sbjct: 984 gtgtaatggatagtgatcttaatggtgatggtgatgatgctgataaaatgaacagttcgt 925


Query: 127 aacggtagctcccacaattttatctttgcttgctccgccagaagagatacaaccagtaaa 186
           ||||||||||||||||||||||||||||||||||||||||||||||||||||||||||||
Sbjct: 924 aacggtagctcccacaattttatctttgcttgctccgccagaagagatacaaccagtaaa 865


Query: 187 cttatgtgttccagaagccggtgcttttgggaaatagaaatttacgttgtttttgtacc 246
           ||||||||||||||||||||||||||||||||||||||||||||||||||||||||||||
Sbjct: 864 cttatgtgttccagaagccggtgcttttgggaaatagaaatttacgttgtttttgtacc 805


Query: 247 agtacaatttactagtgtaatataaccagtattgttattttggtcatatcctctagaacg 306
```

```
            ||||||||||||||||||||||||||||||||||||||||||||||||||||||||||||
Sbjct: 804 agtacaatttactagtgtaatataaccagtattgttattttggtcatatcctctagaacg 745


Query: 307 atttcctgtggcagtacaattttcaagataatgattatccgctgagttatttcctcctac 366
            ||||||||||||||||||||||||||||||||||||||||||||||||||||||||||||
Sbjct: 744 atttcctgtggcagtacaattttcaagataatgattatccgctgagttatttcctcctac 685


Query: 367 tttaaaaccatttccattgccatccgctactccgttataggatgcttcacagtcatatac 426
            ||||||||||||||||||||||||||||||||||||||||||||||||||||||||||||
Sbjct: 684 tttaaaaccatttccattgccatccgctactccgttataggatgcttcacagtcatatac 625


Query: 427 ctttactgcatttttcgcatcataaaagtcaaatccatcgtcggaattattgtatgcttt 486
            ||||||||||||||||||||||||||||||||||||||||||||||||||||||||||||
Sbjct: 624 ctttactgcatttttcgcatcataaaagtcaaatccatcgtcggaattattgtatgcttt 565


Query: 487 acaggaagtaaaaacatttccaggtccgatacctaattttgctgcaaaaccatccgcatt 546
            ||||||||||||||||||||||||||||||||||||||||||||||||||||||||||||
Sbjct: 564 acaggaagtaaaaacatttccaggtccgatacctaattttgctgcaaaaccatccgcatt 505


Query: 547 ctcaccattcgttttcttatcataattgtaagtggaagtaacacgagtaattgtattata 606
            ||||||||||||||||||||||||||||||||||||||||||||||||||||||||||||
Sbjct: 504 ctcaccattcgttttcttatcataattgtaagtggaagtaacacgagtaattgtattata 445


Query: 607 agaaccaccattactgatttgtaaaccagtatctccgcacttagtgataaaacaatcttg 666
            ||||||||||||||||||||||||||||||||||||||||||||||||||||||||||||
Sbjct: 444 agaaccaccattactgatttgtaaaccagtatctccgcacttagtgataaaacaatcttg 385


Query: 667 cactcgattatagtttccacttatgtgaataccattatccccagcaccagttatcgttaa 726
            ||||||||||||||||||||||||||||||||||||||||||||||||||||||||||||
Sbjct: 384 cactcgattatagtttccacttatgtgaataccattatccccagcaccagttatcgttaa 325


Query: 727 accagcgataatccaataatttcccgatatttgaaaacctcttgaactatccgcatattc 786
            ||||||||||||||||||||||||||||||||||||||||||||||||||||||||||||
Sbjct: 324 accagcgataatccaataatttcccgatatttgaaaacctcttgaactatccgcatattc 265


Query: 787 ttgactagaaaaatcaataacagggctatgtccgctatagtttaatatttttattggctc 846
            ||||||||||||||||||||||||||||||||||||||||||||||||||||||||||||
Sbjct: 264 ttgactagaaaaatcaataacagggctatgtccgctatagtttaatatttttattggctc 205


Query: 847 agaagccgtaccattactagttagtttaaatgtcgtagaataactgtaagttccatttaa 906
            ||||||||||||||||||||||||||||||||||||||||||||||||||||||||||||
Sbjct: 204 agaagccgtaccattactagttagtttaaatgtcgtagaataactgtaagttccatttaa 145


Query: 907 aacaaaaattgttgttcctgcactagctttagttaaagccttttgtaagagttttaaaa 965
            ||||||||||||||||||||||||||||||||||||||| ||||||||||||||||||||
Sbjct: 144 aacaaaaattgttgttcctgcactagctttagttaaag-cttttgtaagagttttaaaa 87
```

```
#######

# cphy3202_2

Query= HR0ADKBY78ZG03_CAZY8C2P16_1.SCF

>Cphy3202* forward

Query: 66  aaagaaggagataggatcATGacagcagacggtcttacctctcaacagtatgttgaggca 125
           ||||||||||||||||||||||||||||||||||||||||||||||||||||||||||||
Sbjct: 1   aaagaaggagataggatcatgacagcagacggtcttacctctcaacagtatgttgaggca 60


Query: 126 atgggcgaaggctggaacttaggaaattcctttgatggttttgattctgatacttcaaaa 185
           ||||||||||||||||||||||||||||||||||||||||||||||||||||||||||||
Sbjct: 61  atgggcgaaggctggaacttaggaaattcctttgatggttttgattctgatacttcaaaa 120


Query: 186 ccagatcaaggcgagaccgcttggggaaatcctaaggttacaaaagagctaatccatgca 245
           ||||||||||||||||||||||||||||||||||||||||||||||||||||||||||||
Sbjct: 121 ccagatcaaggcgagaccgcttggggaaatcctaaggttacaaaagagctaatccatgca 180


Query: 246 gtcaaacaaaaaggctatagtagtatccgcataccaatgaccctatatcgtagatatacg 305
           ||||||||||||||||||||||||||||||||||||||||||||||||||||||||||||
Sbjct: 181 gtcaaacaaaaaggctatagtagtatccgcataccaatgaccctatatcgtagatatacg 240


Query: 306 gagagcaatggtgtatgcactatcgatagcgcatggatagcacgttacaaagaagtagta 365
           ||||||||||||||||||||||||||||||||||||||||||||||||||||||||||||
Sbjct: 241 gagagcaatggtgtatgcactatcgatagcgcatggatagcacgttacaaagaagtagta 300


Query: 366 gattatgcagttgcagaaggtttatacgttatgataaacattcaccatgattcctggata 425
           ||||||||||||||||||||||||||||||||||||||||||||||||||||||||||||
Sbjct: 301 gattatgcagttgcagaaggtttatacgttatgataaacattcaccatgattcctggata 360


Query: 426 tggttatcttcatgggatggaaataagagttctgtgcaatatgtaagatttactcagatg 485
           ||||||||||||||||||||||||||||||||||||||||||||||||||||||||||||
Sbjct: 361 tggttatcttcatgggatggaaataagagttctgtgcaatatgtaagatttactcagatg 420


Query: 486 tgggatcaacttgcgaaggcatttaaagattatccgttacaagtatgttttgaaacgata 545
           ||||||||||||||||||||||||||||||||||||||||||||||||||||||||||||
Sbjct: 421 tgggatcaacttgcgaaggcatttaaagattatccgttacaagtatgttttgaaacgata 480


Query: 546 aatgagccgaactttcaaaactctggaaaagttactgcacagaataaattagatatgctt 605
           |||||||||||||||||||||||||||| |||||||||||||||||||||||||||||||
Sbjct: 481 aatgagccgaactttcaaaactctggaaacgttactgcacagaataaattagatatgctt 540


Query: 606 aaccaagcggcttacaatataattcgtgcctctggtggatcaaatgcaaagagaatgatt 665
           ||||||||||||||||||||||||||||||||||||||||||||||||||||||||||||
Sbjct: 541 aaccaagcggcttacaatataattcgtgcctctggtggatcaaatgcaaagagaatgatt 600
```

```
Query: 666 gttttaccatcactaaatacgaaccatgataatagtgtaccattagctgatttcataact 725
           ||||||||||||||||||||||||||||||||||||||||||||||||||||||||||||
Sbjct: 601 gttttaccatcactaaatacgaaccatgataatagtgtaccattagctgatttcataact 660


Query: 726 aaatttgaatgattctaataatcattgccaccgttccttaatatagggaaagggtattta 785
           ||| ||||||||||||||| |||||||| ||||||| ||| ||||| ||| |||||||||
Sbjct: 661 aaa-ttgaatgattctaat-atcattgcaaccgttcattattatagtgaatgggtattta 718


Query: 786 gtgctaacccttggtaaaaacaagcttttgatgaagaattatggggaaaatggtgaatac 845
           ||||||| ||||||| || |||||| ||||||||||| |||||||| ||||||||| |||
Sbjct: 719 gtgctaa-ccttggt-aagacaagc-tttgatgaagatttatgggg-aaatggtgattac 774


Query: 846 cctccctcgggaagcggtaaaaaggcgttttgataccattt 887
            || ||||| || |||||||| |||||| |||||||||||||
Sbjct: 775 act-cctcgtgatgcggtaaataaggcg-tttgataccattt 814
```

Query= HR0ADKBY78ZG03_CAZY8C2P17_1.SCF

>Cphy3202* reverse

```
Query: 67   gtgtaatggatagtgatcTTAatggtgatggtgatgatgatttgaactaactaaagtaac 126
             ||||||||||||||||||||||||||||||||||||||||||||||||||||||||||||
Sbjct: 2193 gtgtaatggatagtgatcttaatggtgatggtgatgatgatttgaactaactaaagtaac 2134


Query: 127  atttgatggtggatcaacagacggtgtatcatgtgaagcaataaaaccaaatgttatact 186
             ||||||||||||||||||||||||||||||||||||||||||||||||||||||||||||
Sbjct: 2133 atttgatggtggatcaacagacggtgtatcatgtgaagcaataaaaccaaatgttatact 2074


Query: 187  ttgccctggatctagattcgtagcccatgctggtcccttcactgtaatggtacccgaatt 246
             ||||||||||||||||||||||||||||||||||||||||||||||||||||||||||||
Sbjct: 2073 ttgccctggatctagattcgtagcccatgctggtcccttcactgtaatggtacccgaatt 2014


Query: 247  ttgactaaccaaatctgcactccacatttggtctatatacccttataagtaaatgatac 306
             ||||||||||||||||||||||||||||||||||||||||||||||||||||||||||||
Sbjct: 2013 ttgactaaccaaatctgcactccacatttggtctatatacccttataagtaaatgatac 1954


Query: 307  aacaaaattctttagtgctgtattagaggtattggttaatgtaatattcgcctgtgtatg 366
             ||||||||||||||||||||||||||||||||||||||||||||||||||||||||||||
Sbjct: 1953 aacaaaattctttagtgctgtattagaggtattggttaatgtaatattcgcctgtgtatg 1894


Query: 367  cttatcccaagtattcgttacttcgtattttaatgtaactggacctgttcctgggattgg 426
             ||||||||||||||||||||||||||||||||||||||||||||||||||||||||||||
Sbjct: 1893 cttatcccaagtattcgttacttcgtattttaatgtaactggacctgttcctgggattgg 1834


Query: 427  agtaggagttggtgttactgtcggtgttat 456
             ||||||||||||||||||||||||||||||
Sbjct: 1833 agtaggagttggtgttactgtcggtgttat 1804
```

```
 Score =  674 bits (340), Expect = 0.0
 Identities = 362/368 (98%), Gaps = 1/368 (0%)
 Strand = Plus / Minus


Query: 574  aggggttactgctgctgcagttcctgttatgttaccatttgatttattaagattatattt 633
            ||||||||||||||||||||||||||||||||||||||||||||||||||||||||||||
Sbjct: 1686 aggggttactgctgctgcagttcctgttatgttaccatttgatttattaagattatattt 1627


Query: 634  taacttttgtccatcataaaactccatctcaaatgatatatttccatcataaacagaacc 693
            ||||||||||||||||||||||||||||||||||||||||||||||||||||||||||||
Sbjct: 1626 taacttttgtccatcataaaactccatctcaaatgatatatttccatcataaacagaacc 1567


Query: 694  atccttaaagaaatcaggcttaatggttaaaatattgttcgtatagttcgccacaaaagt 753
            ||||||||||||||||||||||||||||||||||||||||||||||||||||||||||||
Sbjct: 1566 atccttaaagaaatcaggcttaatggttaaaatattgttcgtatagttcgccacaaaagt 1507


Query: 754  tgcaccatactctaaatacatccaccagctttgattcgggccaactcgattagaacctat 813
            ||||||||||||||||||||||||||||||||||||||||||||||||||||||||||||
Sbjct: 1506 tgcaccatactctaaatacatccaccagctttgattcgggccaactcgattagaacctat 1447


Query: 814  ataagctgtagaacgacggagtttacttccgtttaattgaactggaatattaattccatt 873
            ||||||||||||||||||||||||||||||||| ||||||||||||||||||||||||||
Sbjct: 1446 ataagctgtagaacgacggagtttacttccgttaaattgaactggaatattaattccatt 1387


Query: 874  ggaaacagttccatttccattttgaaatactggtgctttgtatttcaacaaaatactcag 933
            |||||||||||||||  ||||||||||||||||||||||||||||| || |||||||||
Sbjct: 1386 ggaaacagttccattcgcattttgaaatactggtgctttgtatttc-actaaatactcat 1328


Query: 934  gccaatca 941
            ||||||||
Sbjct: 1327 gccaatca 1320

#######

# cphy3207_1

Query= HR0ADKBY78ZF05_CAZY14C1P16_1.SCF

>Cphy3207 forward


Query: 68  aaagaaggagataggatcatgaATGgtgcaataaaaagtgggtattaccggaatgtgttt 127
           ||||||||||||||||||||||||||||||||||||||||||||||||||||||||||||
Sbjct: 1   aaagaaggagataggatcatgaatggtgcaataaaaagtgggtattaccggaatgtgttt 60


Query: 128 accgaattaggctataaagaagaggacgtaactaagaaagttgaggacagctttcagacc 187
           ||||||||||||||||||||||||||||||||||||||||||||||||||||||||||||
```

```
Sbjct: 61  accgaattaggctataaagaagaggacgtaactaagaaagttgaggacagctttcagacc 120


Query: 188 ttgttctatggtctaccagaagaacgcatatattatcctgtgggtgaagatttaggatat 247
           ||||||||||||||||||||||||||||||||||||||||||||||||||||||||||||
Sbjct: 121 ttgttctatggtctaccagaagaacgcatatattatcctgtgggtgaagatttaggatat 180


Query: 248 atcgtggataccggaaatcatgatgttcgaacagaaggaatgtcttatggaatgatgatg 307
           ||||||||||||||||||||||||||||||||||||||||||||||||||||||||||||
Sbjct: 181 atcgtggataccggaaatcatgatgttcgaacagaaggaatgtcttatggaatgatgatg 240


Query: 308 tgtttgcaattagataaaaaagaggaatttgatcgcttatggaaatgggctaagacatat 367
           ||||||||||||||||||||||||||||||||||||||||||||||||||||||||||||
Sbjct: 241 tgtttgcaattagataaaaaagaggaatttgatcgcttatggaaatgggctaagacatat 300


Query: 368 atgtttatggattctggtgttaacaaaggctattttgcttggtcatgtaaaacagatggt 427
           ||||||||||||||||||||||||||||||||||||||||||||||||||||||||||||
Sbjct: 301 atgtttatggattctggtgttaacaaaggctattttgcttggtcatgtaaaacagatggt 360


Query: 428 acgaaaaattcctatggaccagcaccggatggtgaggagtattttgctttagcattattc 487
           ||||||||||||||||||||||||||||||||||||||||||||||||||||||||||||
Sbjct: 361 acgaaaaattcctatggaccagcaccggatggtgaggagtattttgctttagcattattc 420


Query: 488 tttgcttccaaccgctggggtgatggaaatggaattttcgaatatagtaaacaagcgaga 547
           ||||||||||||||||||||||||||||||||||||||||||||||||||||||||||||
Sbjct: 421 tttgcttccaaccgctggggtgatggaaatggaattttcgaatatagtaaacaagcgaga 480


Query: 548 gaacttcttcatgaatgtatccataaaggagaagaggatggaatcggagaacctatgtgg 607
           ||||||||||||||||||||||||||||||||||||||||||||||||||||||||||||
Sbjct: 481 gaacttcttcatgaatgtatccataaaggagaagaggatggaatcggagaacctatgtgg 540


Query: 608 gagccatcgaactacctgataaaattcatacctta ttgccaattttacagatccatcgta 667
           ||||||||||||||||||||||||||||||||| |||| |||||||||||||||||||||
Sbjct: 541 gagccatcgaactacctgataaaattcatacctaattg-caattttacagatccatcgta 599


Query: 668 tcatttaccacacttttaccgggttatttgcactttgggcatatgaagaaagatagaaaa 727
           |||||||||||||||||| ||||||||||||||||||||||||||||| |||||||| ||
Sbjct: 600 tcatttaccacactttta-cgggttatttgcactttgggcatatgaag-aagatagagaa 657


Query: 728 ttctttaaaaaggcaacggaagcaagtcgtttctaatcttaaacttgcatgtcatg 783
           ||||||||||||||| ||||||| ||||| |||  |||| ||||||||||||||||
Sbjct: 658 ttctttaaaaaggcagcggaagcgagtcg-ttcgtatctaaaacttgcatgtcatg 712



Query= HR0ADKBY78ZF05_CAZY14C1P17_1.SCF

>Cphy3207 reverse
```

```
Query: 67   gtgtaatggatagtgatcTTAatggtgatggtgatgatgcatccacatacgatactttcc 126
            ||||||||||||||||||||||||||||||||||||||||||||||||||||||||||||
Sbjct: 1200 gtgtaatggatagtgatcttaatggtgatggtgatgatgcatccacatacgatactttcc 1141


Query: 127  acttaaagctaagagtgcaaacatatacaagcaattgtcgtaatatcttctatcgccagt 186
            ||||||||||||||||||||||||||||||||||||||||||||||||||||||||||||
Sbjct: 1140 acttaaagctaagagtgcaaacatatacaagcaattgtcgtaatatcttctatcgccagt 1081


Query: 187  ccttaattctgtatgataaaacttgtcgacacattctttcgcaaagggaccatttgctgc 246
            ||||||||||||||||||||||||||||||||||||||||||||||||||||||||||||
Sbjct: 1080 ccttaattctgtatgataaaacttgtcgacacattctttcgcaaagggaccatttgctgc 1021


Query: 247  taaagatgcttgcgcattggtagcgattattgcaacagggtgtaaagcctcgcccttgat 306
            ||||||||||||||||||||||||||||||||||||||||||||||||||||||||||||
Sbjct: 1020 taaagatgcttgcgcattggtagcgattattgcaacagggtgtaaagcctcgcccttgat 961


Query: 307  tatcgttccatctacttgataaataccacttgcatgctcttttactgtctcacaaaagaa 366
            ||||||||||||||||||||||||||||||||||||||||||||||||||||||||||||
Sbjct: 960  tatcgttccatctacttgataaataccacttgcatgctcttttactgtctcacaaaagaa 901


Query: 367  ttgctgtaaatgattagcgcatgttacttgccattcatcagctgcaaaccagagataatc 426
            ||||||||||||||||||||||||||||||||||||||||||||||||||||||||||||
Sbjct: 900  ttgctgtaaatgattagcgcatgttacttgccattcatcagctgcaaaccagagataatc 841


Query: 427  aagaccaatgtttgcaatggttcgataagcatcgctgtaataccaatcatggcgtccaaa 486
            ||||||||||||||||||||||||||||||||||||||||||||||||||||||||||||
Sbjct: 840  aagaccaatgtttgcaatggttcgataagcatcgctgtaataccaatcatggcgtccaaa 781


Query: 487  tatttcttgatctccactatgagcagttccatcgtactctgtgtattctgcgcaaagccc 546
            ||||||||||||||||||||||||||||||||||||||||||||||||||||||||||||
Sbjct: 780  tatttcttgatctccactatgagcagttccatcgtactctgtgtattctgcgcaaagccc 721


Query: 547  agtcttttcatgacatgcaagttttagatacgaacgactcgcttccgctgcctttttaaa 606
            ||||||||||||||||||||||||||||||||||||||||||||||||||||||||||||
Sbjct: 720  agtcttttcatgacatgcaagttttagatacgaacgactcgcttccgctgcctttttaaa 661


Query: 607  gaattctctatcttcttcatatgcccaaagtgcaaataacccgtaaaagtgtggtaaatg 666
            ||||||||||||||||||||||||||||||||||||||||||||||||||||||||||||
Sbjct: 660  gaattctctatcttcttcatatgcccaaagtgcaaataacccgtaaaagtgtggtaaatg 601


Query: 667  atacgatggatctgtaaaattgcaattaggtatgaattttatcaggtagttcgatggctc 726
            ||||||||||||||||||||||||||||||||||||||||||||||||||||||||||||
Sbjct: 600  atacgatggatctgtaaaattgcaattaggtatgaattttatcaggtagttcgatggctc 541


Query: 727  ccacataggttctccgattccatcctcttctcctttatggatacattcatgaagaaattc 786
            |||||||||||||||||||||||||||||||||||||||||||||||||||||||| |||
```

```
Sbjct: 540  ccacataggttctccgattccatcctcttctcctttatggatacattcatgaagaagttc 481


Query: 787  tcccgctggtttaccatattcgaaaattccctttcctccaccccagcggttggaagcaaa 846
            || |||| |||||| ||||||||||||||| |||||  ||||||||||||||||||||||
Sbjct: 480  tctcgcttgtttactatattcgaaaattccatttccatcaccccagcggttggaagcaaa 421


Query: 847  aaataatgctaaaccaaaaatactcctcaccctcccggggctggtccataagaaattttt 906
             |||||||||||| | ||||||||||||||| | |||| |||||||||| || |||||||
Sbjct: 420  gaataatgctaaagc-aaaatactcctcaccat-ccggtgctggtccat-aggaattttt 364


Query: 907  cgtacca 913
            |||||||
Sbjct: 363  cgtacca 357

#######

# cphy3581_3

Query= HR0ADKBY78ZC04_CAZY9C3P16_1.SCF

>Cphy3581 forward


Query: 71  aaagaaggagataggatcATGatatttcgacatggcgaagtttttattaatggaaaattt 130
           ||||||||||||||||||||||||||||||||||||||||||||||||||||||||||||
Sbjct: 1   aaagaaggagataggatcatgatatttcgacatggcgaagtttttattaatggaaaattt 60


Query: 131 gaacgaaaagatattcttgttgaagatggatttgtaaaagaaatatcagagacaatcaca 190
           ||||||||||||||||||||||||||||||||||||||||||||||||||||||||||||
Sbjct: 61  gaacgaaaagatattcttgttgaagatggatttgtaaaagaaatatcagagacaatcaca 120


Query: 191 ggagatgggaatgaagtagattgtacaggtttacgcattgtacctggtttctttgatatt 250
           ||||||||||||||||||||||||||||||||||||||||||||||||||||||||||||
Sbjct: 121 ggagatgggaatgaagtagattgtacaggtttacgcattgtacctggtttctttgatatt 180


Query: 251 catacgcatggatgtttatcttacgatttctctttatcaaatccagaagaaatcaaagag 310
           ||||||||||||||||||||||||||||||||||||||||||||||||||||||||||||
Sbjct: 181 catacgcatggatgtttatcttacgatttctctttatcaaatccagaagaaatcaaagag 240


Query: 311 atgtgcgagtattatgcaaagcatggtgtaacctcaatacttgctacaccaatgacaaat 370
           |||||||||||||||||||||||||||||||||||||||||||||||| |||||||||||
Sbjct: 241 atgtgcgagtattatgcaaagcatggtgtaacctcaatacttgctacaacaatgacaaat 300


Query: 371 gaggaaaatcagtatcatcgggcaatggtttatataaaagaggtcatggatgatcaaaaa 430
           ||||||||||||||||||||||||||||||||||||||||||||||||||||||||||||
Sbjct: 301 gaggaaaatcagtatcatcgggcaatggtttatataaaagaggtcatggatgatcaaaaa 360


Query: 431 gaacactcagataaaaaagaagctgcaatcgaaggaattaatatggaaggacctttcttt 490
           ||||||||||||||||||||||||||||||||||||||||||||||||||||||||||||
```

```
Sbjct: 361 gaacactcagataaaaagaagctgcaatcgaaggaattaatatggaaggacctttcttt 420


Query: 491 ggtatagaaaagaagggtgcacatgatcctcaatatttaagaagaatttctcaagactta 550
            ||||||||||||||||||||||||||||||||||||||||||||||||||||||||||||
Sbjct: 421 ggtatagaaaagaagggtgcacatgatcctcaatatttaagaagaatttctcaagactta 480


Query: 551 tttgacgaatataatgagttatcccgggaatgcaattcgcttagtagatattgatccaac 610
            |||||||||||||||||||||| |||||||||||||||||||||||||||||||||||||
Sbjct: 481 tttgacgaatataatgagttat-ccgggaatgcaattcgcttagtagatattgatccaac 539


Query: 611 cttaaaggagcattggagtttattcaaaggaacaaagagttctttacgaatatccttag 670
            |||| | |||||||||||||||||||||||||||||||||||||||||| ||||||||||
Sbjct: 540 cttagatggagcattggagtttattcaaaggaacaaagagttctttacg-atatccttag 598


Query: 671 ctcatacaatgagtacctttgaccaacca-aaactgtcacgaaagcggggtgcaactcag 729
            |||||||||||||||||||||| ||| || || |||   ||||||| ||||||||||||
Sbjct: 599 ctcatacaatgagtacctttgatcaagcagaagctgcagcgaaagc-gggtgcaactcat 657


Query: 730 gttactcctttattttatgcgatggaacccattgttaccatcgtgaaccagggtttagtt 789
            ||||||| ||||||| ||||||| ||  ||||||||| |||||||||||| |||||||||
Sbjct: 658 gttactcatttatttaatgcgat-gagaccattgtta-catcgtgaacca-ggtttagtt 714


Query: 790 gggtccaattagtgaatttggattaattgcaaaatta 827
             ||| || ||||||| ||||| |||| |||| ||||||
Sbjct: 715 -ggtgcagttagtgagtttggtttaaatgcagaaatta 751

Query= HR0ADKBY78ZC04_CAZY9C3P17_1.SCF

>Cphy3581 reverse


Query: 68   gtgtaatggatagtgatcTTAatggtgatggtgatgatgaatttcatttcctcttacata 127
             ||||||||||||||||||||||||||||||||||||||||||||||||||||||||||||
Sbjct: 1188 gtgtaatggatagtgatcttaatggtgatggtgatgatgaatttcatttcctcttacata 1129


Query: 128  aacttgttttaaattaaaatcattatccacaagtagtaaatctgcttttcttccaagacc 187
             ||||||||||||||||||||||||||||||||||||||||||||||||||||||||||||
Sbjct: 1128 aacttgttttaaattaaaatcattatccacaagtagtaaatctgcttttcttccaagacc 1069


Query: 188  aatgcttcctactgtatcctctaatttaagagattttgcaggatagtaactagcgcttag 247
             ||||||||||||||||||||||||||||||||||||||||||||||||||||||||||||
Sbjct: 1068 aatgcttcctactgtatcctctaatttaagagattttgcaggatagtaactagcgcttag 1009


Query: 248  aatcgcttcctcaattggaatgccaaatgatattgcattctttactgcatcaaatagagt 307
             ||||||||||||||||||||||||||||||||||||||||||||||||||||||||||||
Sbjct: 1008 aatcgcttcctcaattggaatgccaaatgatattgcattctttactgcatcaaatagagt 949


Query: 308  tatcgtagaaccagcaagagtaccatcttttaagaatgccttatgatcctttactgtgat 367
```

```
             ||||||||||||||||||||||||||||||||||||||||||||||||||||||||||||
Sbjct: 948  tatcgtagaaccagcaagagtaccatcttttaagaatgccttatgatcctttactgtgat 889


Query: 368  tggtaatcctcctgctacatattcaccatctggtaaaccagtaggattgattgagtcaga 427
             ||||||||||||||||||||||||||||||||||||||||||||||||||||||||||||
Sbjct: 888  tggtaatcctcctgctacatattcaccatctggtaaaccagtaggattgattgagtcaga 829


Query: 428  gattaaaatcatctgatctggtactgctttaaacatcagctttactacagctggatgaat 487
             ||||||||||||||||||||||||||||||||||||||||||||||||||||||||||||
Sbjct: 828  gattaaaatcatctgatctggtactgctttaaacatcagctttactacagctggatgaat 769


Query: 488  atgaattccgtcacagataatttctgcatttaaaccaaactcactaactgcaccaactaa 547
             ||||||||||||||||||||||||||||||||||||||||||||||||||||||||||||
Sbjct: 768  atgaattccgtcacagataatttctgcatttaaaccaaactcactaactgcaccaactaa 709


Query: 548  acctggttcacgatgtaacaatggtctcatcgcattaaataaatgagtaacatgagttgc 607
             ||||||||||||||||||||||||||||||||||||||||||||||||||||||||||||
Sbjct: 708  acctggttcacgatgtaacaatggtctcatcgcattaaataaatgagtaacatgagttgc 649


Query: 608  acccgctttcgctgcagcttctgcttgatcaaaggtactcattgtatgagctaaggatat 667
             ||||||||||||||||||||||||||||||||||||||||||||||||||||||||||||
Sbjct: 648  acccgctttcgctgcagcttctgcttgatcaaaggtactcattgtatgagctaaggatat 589


Query: 668  cgtaaagaactctttgttcctttgaataaactccaatgctccatctaaagttggatcaat 727
             |||||||||||||||||||||||||||||||||||||||||||||||| |||||||||||
Sbjct: 588  cgtaaagaactctttgttcctttgaataaactccaatgctccatctaaggttggatcaat 529


Query: 728  atctactaaacaaattgccattccccgataactcattatatttcgttcaaataattcttg 787
             ||||||||| | ||||| ||||||| |||||||||||||| |||| |||||||| |||||
Sbjct: 528  atctactaagcgaattg-cattcccggataactcattata-ttcg-tcaaataagtcttg 472


Query: 788  aaaaatttcttcttaaaaaattggaggatcatggtgcacccttctttt 835
             | ||| ||||||||||| |  | ||||||||| |||||||||||||||
Sbjct: 471  agaaa-ttcttcttaaata--ttgaggatcat-gtgcacccttctttt 428

#######

# cphy3586_1

Query= HR0ADKBY78ZA06_CAZY15C1P16_1.SCF

>Cphy3586* forward


Query: 69  aaagaaggagataggatcATGactagttttgcttatggtgctgatgtgggttggttaagc 128
            ||||||||||||||||||||||||||||||||||||||||||||||||||||||||||||
Sbjct: 1   aaagaaggagataggatcatgactagttttgcttatggtgctgatgtgggttggttaagc 60


Query: 129 cagttagaaaaatcaggtgtaacttgggttgatgactatggttatacaaaggatgcacta 188
```

```
           ||||||||||||||||||||||||||||||||||||||||||||||||||||||||||||
Sbjct: 61  cagttagaaaaatcaggtgtaacttgggttgatgactatggttatacaaaggatgcacta 120


Query: 189 cagatactaaaggatcatggaattgattcgattcgattacgcttgtttgtaaatcctcca 248
           ||||||||||||||||||||||||||||||||||||||||||||||||||||||||||||
Sbjct: 121 cagatactaaaggatcatggaattgattcgattcgattacgcttgtttgtaaatcctcca 180


Query: 249 tccaatttcacttggacgaaaaaggatggatcaacatgtatgctggggtatactgatgcg 308
           ||||||||||||||||||||||||||||||||||||||||||||||||||||||||||||
Sbjct: 181 tccaatttcacttggacgaaaaaggatggatcaacatgtatgctggggtatactgatgcg 240


Query: 309 gctggcttaatatatatggcagaacgatcaaaatcaatgggatttcgaattatggttgat 368
           ||||||||||||||||||||||||||||||||||||||||||||||||||||||||||||
Sbjct: 241 gctggcttaatatatatggcagaacgatcaaaatcaatgggatttcgaattatggttgat 300


Query: 369 tttcattatagtgatcattttgctgaccctgcctatcaggacataccatctgcttggtca 428
           ||||||||||||||||||||||||||||||||||||||||||||||||||||||||||||
Sbjct: 301 tttcattatagtgatcattttgctgaccctgcctatcaggacataccatctgcttggtca 360


Query: 429 tcacatacctttactcagctgaaaaaagatgtttatgatcatacttattctgttatgtct 488
           ||||||||||||||||||||||||||||||||||||||||||||||||||||||||||||
Sbjct: 361 tcacatacctttactcagctgaaaaaagatgtttatgatcatacttattctgttatgtct 420


Query: 489 cagctagctacaaagggaatctatccggagtgggttcaggttgggaacgagattaacagc 548
           ||||||||||||||||||||||||||||||||||||||||||||||||||||||||||||
Sbjct: 421 cagctagctacaaagggaatctatccggagtgggttcaggttgggaacgagattaacagc 480


Query: 549 ggtatgttattaccttatggacagagcagtaataactttagccagctgacagagttactg 608
           ||||||||||||||||||||||||||||||||||||||||||||||||||||||||||||
Sbjct: 481 ggtatgttattaccttatggacagagcagtaataactttagccagctgacagagttactg 540


Query: 609 aatagtggatatgatgcggtaaaaagcggtaaagcaaatcgactaaagttgtaactcatc 668
           |||||||||||||||||||| ||||||||| |||||||||||||||||||||||||||||
Sbjct: 541 aatagtggatatgatgcggt-aaaagcggt-aagcaaatcgactaaagttgtaactcatc 598


Query: 669 ttgcagatggtaataacaatacaacgttttcaatggttctttgataattttattactaaa 728
           |||||||||||||||||||||||||| |||| ||||||||||||||||||||||||| ||
Sbjct: 599 ttgcagatggtaataacaatacaacg-tttcgatggttctttgataattttattact-aa 656


Query: 729 atatggtggaaaaaacaaatgtaattggtatgtcctactatccctaattggaaaggaagt 788
           ||||||||| ||||||| ||||||||||||||||||||||||| | |||||| |||||||
Sbjct: 657 atatggtgg-aaaaacagatgtaattggtatgtcctactatccgt-attggataggaagt 714


Query: 789 gactatacaccagccgattttcttacttggc 819
           ||||||||| ||| ||| |||||||||||||
Sbjct: 715 gactataca-cagtcga-tttcttacttggc 743
```

Query= HR0ADKBY78ZA06_CAZY15C1P17_1.SCF

>Cphy3586* reverse

```
Query: 66   gtgtaatggatagtgatcTTAatggtgatggtgatgatggtttacaatttcaagtctcca 125
            ||||||||||||||||||||||||||||||||||||||||||||||||||||||||||||
Sbjct: 1509 gtgtaatggatagtgatcttaatggtgatggtgatgatggtttacaatttcaagtctcca 1450


Query: 126  catctggcttaacgcattggtgtctgtttgttgcaccaaggccacgccttcctctgttga 185
            ||||||||||||||||||||||||||||||||||||||||||||||||||||||||||||
Sbjct: 1449 catctggcttaacgcattggtgtctgtttgttgcaccaaggccacgccttcctctgttga 1390


Query: 186  agcattctgtacagctaagagtttaccgcttcctcgattgatcagcttataatacccatc 245
            ||||||||||||||||||||||||||||||||||||||||||||||||||||||||||||
Sbjct: 1389 agcattctgtacagctaagagtttaccgcttcctcgattgatcagcttataatacccatc 1330


Query: 246  tcctgtggaacccaaactccattgttggttattaccattattagaggtatactgtattac 305
            ||||||||||||||||||||||||||||||||||||||||||||||||||||||||||||
Sbjct: 1329 tcctgtggaacccaaactccattgttggttattaccattattagaggtatactgtattac 1270


Query: 306  actagctccttcactggtggaagctcctgatacatccattaccttactgcttaatacact 365
            ||||||||||||||||||||||||||||||||||||||||||||||||||||||||||||
Sbjct: 1269 actagctccttcactggtggaagctcctgatacatccattaccttactgcttaatacact 1210


Query: 366  ttttatggtataataaccgctactatttagcgagaaagtccatttttgactactccatgc 425
            ||||||||||||||||||||||||||||||||||||||||||||||||||||||||||||
Sbjct: 1209 ttttatggtataataaccgctactatttagcgagaaagtccatttttgactactccatgc 1150


Query: 426  cccataactatattgctcaaggacagcattatcgcttgttgagccacctcttacattaat 485
            ||||||||||||||||||||||||||||||||||||||||||||||||||||||||||||
Sbjct: 1149 cccataactatattgctcaaggacagcattatcgcttgttgagccacctcttacattaat 1090


Query: 486  tgctttcccactaagtcgattggttagtatatatgttttgctggtatcaaaagatgagct 545
            ||||||||||||||||||||||||||||||||||||||||||||||||||||||||||||
Sbjct: 1089 tgctttcccactaagtcgattggttagtatatatgttttgctggtatcaaaagatgagct 1030


Query: 546  attatagcgacaatcataaaaagcatcaatagctgtggtgaaccttaatttttttcccaga 605
            ||||||||||||||||||||||||||||||||||||||||||||||||||||||||||||
Sbjct: 1029 attatagcgacaatcataaaaagcatcaatagctgtggtgaaccttaatttttttcccaga 970


Query: 606  aacaacgctggttgctccaagcttataacgatccggaagtaccgaggaattagcctctgg 665
            ||||||||||||||||||||||||||||||||||||||||||||||||||||||||||||
Sbjct: 969  aacaacgctggttgctccaagcttataacgatccggaagtaccgaggaattagcctctgg 910


Query: 666  ttcccagtaaaatacacctaaaccttttccgttcggaacagccttcaccttctcaatggt 725
            ||||||||||||||||||||||||||||||||||||||||||||||||||||||||||||
Sbjct: 909  ttcccagtaaaatacacctaaaccttttccgttcggaacagccttcaccttctcaatggt 850
```

```
Query: 726  tgcttttaacaagttataggtattggctgaatctgtttcaacccctcccacttcgcatac 785
            ||||||||||||||||||||||||||||||||||||||||||||||||||||||||||||
Sbjct: 849  tgcttttaacaagttataggtattggctgaatctgtttcaacccctcccacttcgcatac 790


Query: 786  cataacctctttattatagcgagatgccatatcattaaggtttatttgccaagtaagaaa 845
            |||||||||||||||||||||||||||||||||||||||| |||||||||||||||||||
Sbjct: 789  cataacctctttattatagcgagatgccatatcattaagg-ttatttgccaagtaagaaa 731


Query: 846  tcgactgtgtatagtcacttccaatccaatacggatagtaggacataccaattacatctg 905
            |||||||||||||||||||||| |||||||||||||||||||||||||||||||||||||
Sbjct: 730  tcgactgtgtatagtcacttcctatccaatacggatagtaggacataccaattacatctg 671


Query: 906  tttttcca-catattttagtaataaaattatc 936
            |||||||| |||| ||||||||||||||||||
Sbjct: 670  tttttccaccata-tttagtaataaaattatc 640

#######

# cphy3869_2

Query= HR0ADKBY78ZE04_CAZY10C2P16_1.SCF   1367      0   1367  SCF
         (1367 letters)

>Cphy3869* forward

          Length = 4248

 Score = 1390 bits (701), Expect = 0.0
 Identities = 717/721 (99%), Gaps = 1/721 (0%)
 Strand = Plus / Plus


Query: 67  aaagaaggagataggatcATGgaagtaagtgatgaaggaacagcatacgttacggaggat 126
           ||||||||||||||||||||||||||||||||||||||||||||||||||||||||||||
Sbjct: 1   aaagaaggagataggatcatggaagtaagtgatgaaggaacagcatacgttacggaggat 60


Query: 127 actgtagttgatagcctagatgctgatgacagtatgcctgaaggatttgaggtaatggat 186
           ||||||||||||||||||||||||||||||||||||||||||||||||||||||||||||
Sbjct: 61  actgtagttgatagcctagatgctgatgacagtatgcctgaaggatttgaggtaatggat 120


Query: 187 gtgaatgtagcttctgggggaggaatatcttcagaaaaatatgacattgatattaccacg 246
           ||||||||||||||||||||||||||||||||||||||||||||||||||||||||||||
Sbjct: 121 gtgaatgtagcttctgggggaggaatatcttcagaaaaatatgacattgatattaccacg 180


Query: 247 ggacttaaagtgaatactaattatcatggtatagaagttcttgagaatatgtcatataca 306
           ||||||||||||||||||||||||||||||||||||||||||||||||||||||||||||
Sbjct: 181 ggacttaaagtgaatactaattatcatggtatagaagttcttgagaatatgtcatataca 240


Query: 307 gcttccactcaggagatagagggtgtaacatataacggtagtattgtaggaacagggaat 366
```

```
           ||||||||||||||||||||||||||||||||||||||||||||||||||||||||||||
Sbjct: 241 gcttccactcaggagatagagggtgtaacatataacggtagtattgtaggaacagggaat 300


Query: 367 ccttcaccaagtaaaggagatattcctactacgggatctgttgtaaaggttactccagaa 426
           ||||||||||||||||||||||||||||||||||||||||||||||||||||||||||||
Sbjct: 301 ccttcaccaagtaaaggagatattcctactacgggatctgttgtaaaggttactccagaa 360


Query: 427 gacaatggtaaatttacaattgtatttaaattaaatgctgggaaaatttattattttgta 486
           ||||||||||||||||||||||||||||||||||||||||||||||||||||||||||||
Sbjct: 361 gacaatggtaaatttacaattgtatttaaattaaatgctgggaaaatttattattttgta 420


Query: 487 gatagtaataagataaatattgatagctacaaagcaggtggtacaaatgaatttattgtt 546
           ||||||||||||||||||||||||||||||||||||||||||||||||||||||||||||
Sbjct: 421 gatagtaataagataaatattgatagctacaaagcaggtggtacaaatgaatttattgtt 480


Query: 547 aaaacttatgaagtagaagcaggaaaatcctattacttctatggagatggaactaaaatt 606
           ||||||||||||||||||||||||||||||||||||||||||||||||||||||||||||
Sbjct: 481 aaaacttatgaagtagaagcaggaaaatcctattacttctatggagatggaactaaaatt 540


Query: 607 cctatgtacggtttatctatcgtctatggtgataattccgtatcttgggaagatataaaa 666
           ||||||||||||||||||||||||||||||||||||||||||||||||||||||||| ||
Sbjct: 541 cctatgtacggtttatctatcgtctatggtgataattccgtatcttgggaagatatagaa 600


Query: 667 tcgccttcatttagcaaagttgcagcaaatgataatgcgatcgcggtagattttctcggt 726
           ||||||||| ||||||||||||||||||||||||||||||||||||||||||||||||||
Sbjct: 601 tcgccttcaattagcaaagttgcagcaaatgataatgcgatcgcggtagattttctcggt 660


Query: 727 aaagtttggtaatggctatgcagattctataactattgagatgtacaaaggtgatactct 786
           |||| ||||||||||||||||||||||||||||||||||||||||| |||||||||||||
Sbjct: 661 aaag-ttggtaatggctatgcagattctataactattgagatgtacgaaggtgatactct 719


Query: 787 t 787
           |
Sbjct: 720 t 720


Query= HR0ADKBY78ZE04_CAZY10C2P17_1.SCF   1342      0   1342  SCF

>Cphy3869* reverse

          Length = 4248

 Score = 1568 bits (791), Expect = 0.0
 Identities = 864/879 (98%), Gaps = 7/879 (0%)
 Strand = Plus / Minus


Query: 68   gtgtaatggatagtgatcTTAatggtgatggtgatgatgctcttcaataataactttaat 127
            ||||||||||||||||||||||||||||||||||||||||||||||||||||||||||||
Sbjct: 4248 gtgtaatggatagtgatcttaatggtgatggtgatgatgctcttcaataataactttaat 4189
```

```
Query: 128  tgacttcttcacaccatctacgattacataaatccaatctgtttccgcagaaactgcttt 187
            ||||||||||||||||||||||||||||||||||||||||||||||||||||||||||||
Sbjct: 4188 tgacttcttcacaccatctacgattacataaatccaatctgtttccgcagaaactgcttt 4129


Query: 188  taccgttgcagttgtacttcctggattcttctttacaactgcaccatctttttttacttga 247
            ||||||||||||||||||||||||||||||||||||||||||||||||||||||||||||
Sbjct: 4128 taccgttgcagttgtacttcctggattcttctttacaactgcaccatctttttttacttga 4069


Query: 248  catccagatgatgctatcttcatccaaaccgttaactttgatttcaaatactgcttcttc 307
            ||||||||||||||||||||||||||||||||||||||||||||||||||||||||||||
Sbjct: 4068 catccagatgatgctatcttcatccaaaccgttaactttgatttcaaatactgcttcttc 4009


Query: 308  tcccactttcattcgtacagtactttccacaaattctacggttgcctttttaatagataa 367
            ||||||||||||||||||||||||||||||||||||||||||||||||||||||||||||
Sbjct: 4008 tcccactttcattcgtacagtactttccacaaattctacggttgcctttttaatagataa 3949


Query: 368  ttttctagtataaacttcctgtgtaccatcctgcattgttacagtagctgtaatgattgc 427
            ||||||||||||||||||||||||||||||||||||||||||||||||||||||||||||
Sbjct: 3948 ttttctagtataaacttcctgtgtaccatcctgcattgttacagtagctgtaatgattgc 3889


Query: 428  aacaccagattcttttgcaatcagacgtccattttgttttacctctacaatgcctggatt 487
            ||||||||||||||||||||||||||||||||||||||||||||||||||||||||||||
Sbjct: 3888 aacaccagattcttttgcaatcagacgtccattttgttttacctctacaatgcctggatt 3829


Query: 488  atttgacttgtaagtaatcgtgattggtacaattccctctgccaacttacttaatgtttt 547
            ||||||||||||||||||||||||||||||||||||||||||||||||||||||||||||
Sbjct: 3828 atttgacttgtaagtaatcgtgattggtacaattccctctgccaacttacttaatgtttt 3769


Query: 548  ttcatcaaagttagctactttaacaacgttagtcggaagttttaacccaaaccatcctaa 607
            ||||||||||||||||||||||||||||||||||||||||||||||||||||||||||||
Sbjct: 3768 ttcatcaaagttagctactttaacaacgttagtcggaagttttaacccaaaccatcctaa 3709


Query: 608  gttagaactatcaccaccaacataaagagtaccagtacgctttggagttaaatgttcaaa 667
            ||||||||||||||||||||||||||||||||||||||||||||||||||||||||||||
Sbjct: 3708 gttagaactatcaccaccaacataaagagtaccagtacgctttggagttaaatgttcaaa 3649


Query: 668  aatctccctcttttgttaattctggtgctggagttggggtagatgttggggccggtgtag 727
            ||||| ||||||||||||||||||||||||||||||||||||||||||||||||||||||
Sbjct: 3648 aatct-cctcttttgttaattctggtgctggagttggggtagatgttggggccggtgtag 3590


Query: 728  atgttggacctggcacttctggcgattgtgttggttgtggtgttacagttttcaccaact 787
            |||||||| ||||||||||||||||||||||||||||||||||||||||| ||||| |||||
Sbjct: 3589 atgttggagctggcacttctggcgattgtgttggttgtggtgttacag-tttcagcaact 3531


Query: 788  tcagggccgattgttggttcaaatgatggagtccctcctactcttgcaccgatacctgct 847
```

```
             |||||||||||||||||||||||||||||||| |||||||||||||||||||||||||||
Sbjct: 3530 tcagggccgattgttggttcaaatgatggagttcctcctactcttgcaccgatacctgct 3471


Query: 848  ggaacatttctctgttaatacaaggaatccattcatgtttattgttccctcctacttttc 907
            ||| || ||||||||||||||||||||||||||||||||||||||||| | |||| ||||
Sbjct: 3470 ggagca-ttctctgttaatacaaggaatccattcatgtttattgttccat-ctacatttc 3413


Query: 908  ttggaaatcccattgaaaagttaagcttttcaaaccaat 946
            || | ||| |||||| |||||||||| ||||||||||||
Sbjct: 3412 tt-gtaattccattg-aaagttaagc-tttcaaaccaat 3377


#######

Cphy0220

Query= HR0ADKBY80ZA01_CAZYA1C1P16_1.SCF   1314      0   1314  SCF
         (1314 letters)


>Cphy0220 forward
          Length = 2220

 Score = 1564 bits (789), Expect = 0.0
 Identities = 831/841 (98%), Gaps = 3/841 (0%)
 Strand = Plus / Plus


Query: 70  aaagaaggagataggatcATGaagaaggtaaatgaatcagtaatcgaagaattattaagt 129
           ||||||||||||||||||||||||||||||||||||||||||||||||||||||||||||
Sbjct: 1   aaagaaggagataggatcatgaagaaggtaaatgaatcagtaatcgaagaattattaagt 60


Query: 130 cagatgaccttagaagaaaagataggtctaattcatggtaatggcatctttcgtagtggt 189
           ||||||||||||||||||||||||||||||||||||||||||||||||||||||||||||
Sbjct: 61  cagatgaccttagaagaaaagataggtctaattcatggtaatggcatctttcgtagtggt 120


Query: 190 ggggtggaacgccttggcataccttccttgaagatgtcggatggcccaatgggtgttcgt 249
           ||||||||||||||||||||||||||||||||||||||||||||||||||||||||||||
Sbjct: 121 ggggtggaacgccttggcataccttccttgaagatgtcggatggcccaatgggtgttcgt 180


Query: 250 agagagtttgaagacaataggtggtttcttgcgtgcaaaacggacgattacgtatcttat 309
           ||||||||||||||||||||||||||||||||||||||||||||||||||||||||||||
Sbjct: 181 agagagtttgaagacaataggtggtttcttgcgtgcaaaacggacgattacgtatcttat 240


Query: 310 cttccgagcaatagtgcaatagcagctacatggaataaagatcttgcctatacttcaggt 369
           ||||||||||||||||||||||||||||||||||||||||||||||||||||||||||||
Sbjct: 241 cttccgagcaatagtgcaatagcagctacatggaataaagatcttgcctatacttcaggt 300


Query: 370 caagttcttggtagtgaagcaaggggaagaggaaaagatgttatcttagcgccaggtatt 429
           ||||||||||||||||||||||||||||||||||||||||||||||||||||||||||||
Sbjct: 301 caagttcttggtagtgaagcaaggggaagaggaaaagatgttatcttagcgccaggtatt 360
```

```
Query: 430 aatattaaaagaagccctctctgtggtaggaattttgaatacatgagcgaggatccaaag 489
           ||||||||||||||||||||||||||||||||||||||||||||||||||||||||||||
Sbjct: 361 aatattaaaagaagccctctctgtggtaggaattttgaatacatgagcgaggatccaaag 420


Query: 490 ctaattgaagaattagcaagtttcatgattcaagggatacaggagaatgatgtagcagct 549
           ||||||||||||||||||||||||||||||||||||||||||||||||||||||||||||
Sbjct: 421 ctaattgaagaattagcaagtttcatgattcaagggatacaggagaatgatgtagcagct 480


Query: 550 tgtgtaaagcattttgctgtaaataatcaagagactgaccgtttggcagtggatacagat 609
           ||||||||||||||||||||||||||||||||||||||||||||||||||||||||||||
Sbjct: 481 tgtgtaaagcattttgctgtaaataatcaagagactgaccgtttggcagtggatacagat 540


Query: 610 ttagaggatagagcattatttgaaatatatttgcctggttttaaggccgcaattaagaat 669
           ||||||||||||||||||||||||||||||||||||||||||||||||||||||||||||
Sbjct: 541 ttagaggatagagcattatttgaaatatatttgcctggttttaaggccgcaattaagaat 600


Query: 670 ggcgaaagttattccatcatgggagcatacaataagttccgaggtgctcagtgttgtgaa 729
           ||||||||||||||||||||||||||||||||||||||||||||||||||||||||||||
Sbjct: 601 ggcgaaagttattccatcatgggagcatacaataagttccgaggtgctcagtgttgtgaa 660


Query: 730 aattcttatctgcttgggactatcttaaggaaagaatgggaatatgatggaacctttata 789
           |||||||||||||||||||||||||||||| ||||||||||||||||||||||| |||||
Sbjct: 661 aattcttatctgcttgggactatcttaagggaagaatgggaatatgatggaaccattata 720


Query: 790 tcggattggggtggtgtccatgatacaaaggcagcagcgaattcaccactccatattgag 849
           ||||||||||||||||||||||||||||||||||||||||||||||||||| ||||||||
Sbjct: 721 tcggattggggtggtgtccatgatacaaaggcagcagcgaattcaccactcgatattgag 780


Query: 850 atggatgtgaaaaccaaattttgatgaataataactgggcaaatcccccttcttgaggcg 909
           ||||||||| ||||| ||||||||||||| |||||  ||||||| || ||||||||||||
Sbjct: 781 atggatgtg-aaacctaattttgatgaat-ataacatggcaaat-ccgcttcttgaggcg 837


Query: 910 g 910
           |
Sbjct: 838 g 838
```

Cphy0220 reverse sequence

```
Query= HR0ADKBY80ZA01_CAZYA1C1P17_1.SCF   1333      0   1333  SCF
         (1333 letters)

>Cphy0220 reverse
          Length = 2220

 Score = 1683 bits (849), Expect = 0.0
 Identities = 866/874 (99%)
 Strand = Plus / Minus
```

```
Query: 66   gtgtaatggatagtgatcTTAatggtgatggtgatgatgctcaattcttaccatcttgct 125
            ||||||||||||||||||||||||||||||||||||||||||||||||||||||||||||
Sbjct: 2220 gtgtaatggatagtgatcttaatggtgatggtgatgatgctcaattcttaccatcttgct 2161

Query: 126  caacctgatatccctagaagagctaccaacttcaatgatgaactctccgccctctaatac 185
            ||||||||||||||||||||||||||||||||||||||||||||||||||||||||||||
Sbjct: 2160 caacctgatatccctagaagagctaccaacttcaatgatgaactctccgccctctaatac 2101

Query: 186  aaatttcttactctcggtacaataataggtaaaggcagatggctttaattccatcatcaa 245
            ||||||||||||||||||||||||||||||||||||||||||||||||||||||||||||
Sbjct: 2100 aaatttcttactctcggtacaataataggtaaaggcagatggctttaattccatcatcaa 2041

Query: 246  ttctttttcctctcccggctgtnnnnnnnctttggtgaacccttttaattcatgaatagg 305
            ||||||||||||||||||||||       |||||||||||||||||||||||||||||||
Sbjct: 2040 ttctttttcctctcccggctgtaaaaaaactttggtgaacccttttaattcatgaatagg 1981

Query: 306  gcgctttacactgcaaactggatcagaaacataaacttgaattgtctcagctccagctgt 365
            ||||||||||||||||||||||||||||||||||||||||||||||||||||||||||||
Sbjct: 1980 gcgctttacactgcaaactggatcagaaacataaacttgaattgtctcagctccagctgt 1921

Query: 366  cttccctgtatttcttatactaaaacttaggaatgcgccttttcccttatttgctcttac 425
            ||||||||||||||||||||||||||||||||||||||||||||||||||||||||||||
Sbjct: 1920 cttccctgtatttcttatactaaaacttaggaatgcgccttttcccttatttgctcttac 1861

Query: 426  tgttaaatttttataagtgaaatttgtataagaaagtccatgaccaaaacaaaattctgg 485
            ||||||||||||||||||||||||||||||||||||||||||||||||||||||||||||
Sbjct: 1860 tgttaaatttttataagtgaaatttgtataagaaagtccatgaccaaaacaaaattctgg 1801

Query: 486  tgtgatattttccgtatcataataacgatatcctaccataatgccttcgttataataaac 545
            ||||||||||||||||||||||||||||||||||||||||||||||||||||||||||||
Sbjct: 1800 tgtgatattttccgtatcataataacgatatcctaccataatgccttcgttataataaac 1741

Query: 546  acttccttttgtaccaaattccccaaatttatgtgctgggcaatcatttagctcctttgg 605
            ||||||||||||||||||||||||||||||||||||||||||||||||||||||||||||
Sbjct: 1740 acttccttttgtaccaaattccccaaatttatgtgctgggcaatcatttagctcctttgg 1681

Query: 606  catcgattcaggtaattttccagatggatttacaattccaaacaatacatctgctaaagc 665
            ||||||||||||||||||||||||||||||||||||||||||||||||||||||||||||
Sbjct: 1680 catcgattcaggtaattttccagatggatttacaattccaaacaatacatctgctaaagc 1621

Query: 666  tttaccaccttgcattccattataatagctccaaacgattgttttagctttattggagaa 725
            ||||||||||||||||||||||||||||||||||||||||||||||||||||||||||||
Sbjct: 1620 tttaccaccttgcattccattataatagctccaaacgattgttttagctttattggagaa 1561

Query: 726  tttctgcatagaaaccggtgatcctgcacaaacaaccaagattgtattaggattcgcatc 785
```

```
              ||||||||||||||||||||||||||||||||||||||||||||||||||||||||||||
Sbjct: 1560 tttctgcatagaaaccggtgatcctgcacaaacaaccaagattgtattaggattcgcatc 1501


Query: 786  gagcacttcttcaattagtttttcctgagcataaggtaattcatatcttttacgatcacg 845
            ||||||||||||||||||||||||||||||||||||||||||||||||||||||||||||
Sbjct: 1500 gagcacttcttcaattagtttttcctgagcataaggtaattcatatcttttacgatcacg 1441


Query: 846  accttccatatcttgatgatgatctaatcctacaacaagaataacatcctccgtttctct 905
            ||||||||||||||||||||||||||||||||||||||||||||||||||||||||||||
Sbjct: 1440 accttccatatcttgatgatgatctaatcctacaacaagaataacatcctccgtttctct 1381


Query: 906  tggaagtaaaaccgcttcttcccgaagacgtttc 939
            || |||||||||||||||||||||||||||||||
Sbjct: 1380 tgcaagtaaaaccgcttcttcccgaagacgtttc 1347

########

Cphy0430 forward sequence

Query= HR0ADKBY80ZE01_CAZYB2C1P16_1.SCF   1380      0   1380  SCF

>Cphy0430 forward
          Length = 2550

 Score = 1334 bits (673), Expect = 0.0
 Identities = 698/705 (99%), Gaps = 1/705 (0%)
 Strand = Plus / Plus


Query: 69  aaagaaggagataggatcATGaaatttggattctttgatgatttaaacaaagaatacgtt 128
           |||||||||||||||||||||||||||||||| |||||||||||||||||||||||||||
Sbjct: 1   aaagaaggagataggatcatgaaatttggatttttgatgatttaaacaaagaatacgtt 60


Query: 129 atcacaactcctgaaacgccgtatccttggattaactatcttggatcgcaagcgttcttt 188
           ||||||||||||||||||||||||||||||||||||||||||||||||||||||||||||
Sbjct: 61  atcacaactcctgaaacgccgtatccttggattaactatcttggatcgcaagcgttcttt 120


Query: 189 tccttgatatcaaatactgctggaggttacagtttctataaagatgcaaaactacgtagg 248
           ||||||||||||||||||||||||||||||||||||||||||||||||||||||||||||
Sbjct: 121 tccttgatatcaaatactgctggaggttacagtttctataaagatgcaaaactacgtagg 180


Query: 249 attacaagatttcgttataataatgtaccattagatctcggtggtggtcgttattactac 308
           ||||||||||||||||||||||||||||||||||||||||||||||||||||||||||||
Sbjct: 181 attacaagatttcgttataataatgtaccattagatctcggtggtggtcgttattactac 240


Query: 309 ttgtacgataatggtgatttttggtctccgggttttgcccctgcaaaaaaggaactagaa 368
           ||||||||||||||||||||||||||||||||||||||||||||||||||||||||||||
Sbjct: 241 ttgtacgataatggtgatttttggtctccgggttttgcccctgcaaaaaaggaactagaa 300


Query: 369 tattatgagtgtcgtcatggcatgggttatactaaaataacaggaagaagaaatggaatt 428
```

```
           ||||||||||||||||||||||||||||||||||||||||||||||||||||||||||||
Sbjct: 301 tattatgagtgtcgtcatggcatgggttatactaaaataacaggaagaagaaatggaatt 360


Query: 429 gaaacaaagataaccttctttgtaccattagactataatggtgaagtacataaagtgtca 488
           ||||||||||||||||||||||||||||||||||||||||||||||||||||||||||||
Sbjct: 361 gaaacaaagataaccttctttgtaccattagactataatggtgaagtacataaagtgtca 420


Query: 489 attgttaatacaagtaatcaagtgaagaatgtgaaattgttttctttcctcgagtggtgt 548
           ||||||||||||||||||||||||||||||||||||||||||||||||||||||||||||
Sbjct: 421 attgttaatacaagtaatcaagtgaagaatgtgaaattgttttctttcctcgagtggtgt 480


Query: 549 ctgtggaatgcacaagaagattctacaaattttcaacgaaatttctcgacaggtgaagta 608
           ||||||||||||||||||||||||||||||||||||||||||||||||||||||||||||
Sbjct: 481 ctgtggaatgcacaagaagattctacaaattttcaacgaaatttctcgacaggtgaagta 540


Query: 609 gaagttaaagggtctgtaatctatcataagacagagtataaagaaagacgggatcattat 668
           ||||||||||||||||||||||||||||||||||||||||||||||||||||||||||||
Sbjct: 541 gaagttaaagggtctgtaatctatcataagacagagtataaagaaagacgggatcattat 600


Query: 669 gcattttctctgtaaatgcaccgattaacggattcaattctgatcgcgagagttttctt 728
           ||||||||||||||||||||||||||| ||||||| ||||||||||||||||||||||||
Sbjct: 601 gcattttctctgtaaatgcaccgattagcggattcgattctgatcgcgagagttttctt 660


Query: 729 ggaacctatcatggttttgaaaaacccgcaggtagttgccagggg 773
           || |||||||||||||||| | ||||||||||||||||| |||||
Sbjct: 661 ggtacctatcatggttttg-ataacccgcaggtagttgcaagggg 704
```

Cphy0430 reverse sequence

```
Query= HR0ADKBY80ZE01_CAZYB2C1P17_1.SCF   1294      0   1294  SCF

>Cphy0430 reverse
          Length = 2550

 Score = 1768 bits (892), Expect = 0.0
 Identities = 935/944 (99%), Gaps = 4/944 (0%)
 Strand = Plus / Minus


Query: 66   gtgtaatggatagtgatcTTAatggtgatggtgatgatgacctaagatgacttttacttt 125
            ||||||||||||||||||||||||||||||||||||||||||||||||||||||||||||
Sbjct: 2550 gtgtaatggatagtgatcttaatggtgatggtgatgatgacctaagatgacttttacttt 2491


Query: 126  atgttctaatccgtcaccaaataccggaatgatattaccctccacctctttcgcatcaac 185
            ||||||||||||||||||||||||||||||||||||||||||||||||||||||||||||
Sbjct: 2490 atgttctaatccgtcaccaaataccggaatgatattaccctccacctctttcgcatcaac 2431


Query: 186  aactagtttcttaacacctttcgaaacatgatatgggtttactattgtaatatcatacct 245
            ||||||||||||||||||||||||||||||||||||||||||||||||||||||||||||
Sbjct: 2430 aactagtttcttaacacctttcgaaacatgatatgggtttactattgtaatatcatacct 2371
```

```
Query: 246  atcaccacggaattcacgtgttatttgatatacctcccaatcagttgggattgcagggtc 305
            ||||||||||||||||||||||||||||||||||||||||||||||||||||||||||||
Sbjct: 2370 atcaccacggaattcacgtgttatttgatatacctcccaatcagttgggattgcagggtc 2311


Query: 306  aatcattaaaccatcatattctggtttaattccaagtatatattgtgatatcgctacaaa 365
            ||||||||||||||||||||||||||||||||||||||||||||||||||||||||||||
Sbjct: 2310 aatcattaaaccatcatattctggtttaattccaagtatatattgtgatatcgctacaaa 2251


Query: 366  attccaagatgcagttccggttaaccaagaatttttgcttcaccaaaacgtctggcatc 425
            ||||||||||||||||||||||||||||||||||||||||||||||||||||||||||||
Sbjct: 2250 attccaagatgcagttccggttaaccaagaatttttgcttcaccaaaacgtctggcatc 2191


Query: 426  cttacctgcaaccatctgagcgtagacatatggctccaaacgatgaatttcagagtattc 485
            ||||||||||||||||||||||||||||||||||||||||||||||||||||||||||||
Sbjct: 2190 cttacctgcaaccatctgagcgtagacatatggctccaaacgatgaatttcagagtattc 2131


Query: 486  ttctgtatacgctggtgcaattttgtatagtaatcaaaggctttatccccacggcctat 545
            ||||||||||||||||||||||||||||||||||||||||||||||||||||||||||||
Sbjct: 2130 ttctgtatacgctggtgcaattttgtatagtaatcaaaggctttatccccacggcctat 2071


Query: 546  cactgcttccgcacacataatccaggcattgttatgacagaagatacctgcattttcttt 605
            ||||||||||||||||||||||||||||||||||||||||||||||||||||||||||||
Sbjct: 2070 cactgcttccgcacacataatccaggcattgttatgacagaagatacctgcattttcttt 2011


Query: 606  atatccagctggataggtagaaatctcgccatattccacataatatcgtgtaaatgctgg 665
            ||||||||||||||||||||||||||||||||||||||||||||||||||||||||||||
Sbjct: 2010 atatccagctggataggtagaaatctcgccatattccacataatatcgtgtaaatgctgg 1951


Query: 666  attatttagaactaatccgaattttgtacccaaacgtttttctacagaatctaaagcttt 725
            ||||||||||||||||||||||||||||||||||||||||||||||||||||||||||||
Sbjct: 1950 attatttagaactaatccgaattttgtacccaaacgtttttctacagaatctaaagcttt 1891


Query: 726  ttgtgctttcccatcgttaagaccgcattttcccataacgcagaagccttgcgattcgat 785
            ||||||||||||||||||||||||||||||||||||||||||||||||||||||||||||
Sbjct: 1890 ttgtgctttcccatcgttaagaccgcattttcccataacgcagaagccttgcgattcgat 1831


Query: 786  aaatattttccttcttcacactcatcactaccaatttttcttccaaaatcatcgtaggc 845
            ||||||||||||||||||||||||||||||||||||||||||||||||||||||||||||
Sbjct: 1830 aaatattttccttcttcacactcatcactaccaatttttcttccaaaatcatcgtaggc 1771


Query: 846  acgtaaaaaccaagatccatcatagccatgcttcataaatacatctcgcaaattctctac 905
            |||||||||||||||||||||||||||||||||||||| |||||||||||| |||||||||
Sbjct: 1770 acgtaaaaaccaagatccatcatagccatgcttcataattacatctcgcatattctctac 1711


Query: 906  ttctttcatggcgcgaatagactcttcgttaatttctaccttaacccataagaatagcat 965
```

```
            ||||||||||||||||||||||||||||||| || ||||||| | |||||||||||||||
Sbjct: 1710 ttctttcatggcgcgaatagactcttcgttatttcctacctt-atccataagaatagcat 1652


Query: 966  atttcttccccaatataagtaaaacattccgggcaatcataaca 1009
            | |||||||||||||||||| ||||||||| |||||||||||||
Sbjct: 1651 a-ttcttccccaatataagt-aaacattcc-ggcaatcataaca 1611

#######

Cphy0624 forward sequence

Query= HR0ADKBY80ZH01_CAZYC2C4P16_1.SCF   1292      0   1292  SCF


>Cphy0624* forward
          Length = 2238

 Score = 1608 bits (811), Expect = 0.0
 Identities = 830/835 (99%), Gaps = 1/835 (0%)
 Strand = Plus / Plus


Query: 69  aaagaaggagataggatcATGtctacaaatgaagcagtagtatatggtaatctaatttat 128
           ||||||||||||||||||||||||||||||||||||||||||||||||||||||||||||
Sbjct: 1   aaagaaggagataggatcatgtctacaaatgaagcagtagtatatggtaatctaatttat 60


Query: 129 cacgactttgaagcaagcaccaatgggtggggaccaagaggtgacaatgcagaggttgtg 188
           ||||||||||||||||||||||||||||||||||||||||||||||||||||||||||||
Sbjct: 61  cacgactttgaagcaagcaccaatgggtggggaccaagaggtgacaatgcagaggttgtg 120


Query: 189 gcacaaagtacagaagaggcatattcagggttacatagtttaaaaattagcaaacgcacg 248
           ||||||||||||||||||||||||||||||||||||||||||||||||||||||||||||
Sbjct: 121 gcacaaagtacagaagaggcatattcagggttacatagtttaaaaattagcaaacgcacg 180


Query: 249 caaacatggcatggtgctacctgcgatttgacaaaggaacttacgattggagaaacttat 308
           ||||||||||||||||||||||||||||||||||||||||||||||||||||||||||||
Sbjct: 181 caaacatggcatggtgctacctgcgatttgacaaaggaacttacgattggagaaacttat 240


Query: 309 gtatttggaatttatttaaaatataaaggtagctcttattccaatacacaaaagtttagc 368
           ||||||||||||||||||||||||||||||||||||||||||||||||||||||||||||
Sbjct: 241 gtatttggaatttatttaaaatataaaggtagctcttattccaatacacaaaagtttagc 300


Query: 369 ttacaatttcagtacaatgatggtgtgaatgatcagtacaaaacaattaagactttagaa 428
           ||||||||||||||||||||||||||||||||||||||||||||||||||||||||||||
Sbjct: 301 ttacaatttcagtacaatgatggtgtgaatgatcagtacaaaacaattaagactttagaa 360


Query: 429 gtaaccaaagataaatggacattaattcaaggtgagtatacagttccagcagatgccgct 488
           ||||||||||||||||||||||||||||||||||||||||||||||||||||||||||||
Sbjct: 361 gtaaccaaagataaatggacattaattcaaggtgagtatacagttccagcagatgccgct 420
```

```
Query: 489 aatgcaaaggtctatgtcgaaacagaatataagagttcaccatcaagtcaagatctctta 548
           ||||||||||||||||||||||||||||||||||||||||||||||||||||||||||||
Sbjct: 421 aatgcaaaggtctatgtcgaaacagaatataagagttcaccatcaagtcaagatctctta 480


Query: 549 gacttttacattgatgatttcactgcaacgccagcaactttaccacagattcaaaaagat 608
           ||||||||||||||||||||||||||||||||||||||||||||||||||||||||||||
Sbjct: 481 gacttttacattgatgatttcactgcaacgccagcaactttaccacagattcaaaaagat 540


Query: 609 attcctagtttaaaggatgtattttcaagttacttctttgttggtggagcggcaactgca 668
           ||||||||||||||||||||||||||||||||||||||||||||||||||||||||||||
Sbjct: 541 attcctagtttaaaggatgtattttcaagttacttctttgttggtggagcggcaactgca 600


Query: 669 ggtgagattgcaccagctccggcaaaagatttagtagcgaaacattacaataggttaact 728
           ||||||||||||||||||||||||||||||||||||||||||||||||||||||||||||
Sbjct: 601 ggtgagattgcaccagctccggcaaaagatttagtagcgaaacattacaataggttaact 660


Query: 729 cctggtaatgagttaaaaccagactctgtattagattactctgcaaccataacctatatg 788
           ||||||||||||||||||||||||||||||||||||||||||||||||||| ||||||||
Sbjct: 661 cctggtaatgagttaaaaccagactctgtattagattactctgcaaccatagcctatatg 720


Query: 789 gatgcaaatggtggtaatcaagtgaatcctcaggtgaatcttagagctgcgaaaacttta 848
           ||||||||||||||||||||||||||||||||||||||||||||||||||||||||||||
Sbjct: 721 gatgcaaatggtggtaatcaagtgaatcctcaggtgaatcttagagctgcgaaaacttta 780


Query: 849 ctggaatatgcaagaaacaataatataccaatggcgtggacataccttaatatgg 903
           ||||||||||||||| |||||||||||||| | |||||||||||||||| |||||
Sbjct: 781 ctggaatatgcaagagacaataatataccagt-gcgtggacataccttagtatgg 834
```

Cphy0624 reverse sequence

```
Query= HR0ADKBY80ZH01_CAZYC2C4P17_1.SCF   1299      0   1299  SCF

>Cphy0624* reverse

          Length = 2238

 Score = 1709 bits (862), Expect = 0.0
 Identities = 900/912 (98%), Gaps = 2/912 (0%)
 Strand = Plus / Minus


Query: 65   gtgtaatggatagtgatcTTAatggtgatggtgatgatgttgaactttgactccattata 124
            ||||||||||||||||||||||||||||||||||||||||||||||||||||||||||||
Sbjct: 2238 gtgtaatggatagtgatcttaatggtgatggtgatgatgttgaactttgactccattata 2179


Query: 125  gtaaacctttaacacttcattggatatcgtaccaaaatccgtccaatccttcttagcaaa 184
            ||||||||||||||||||||||||||||||||||||||||||||||||||||||||||||
Sbjct: 2178 gtaaacctttaacacttcattggatatcgtaccaaaatccgtccaatccttcttagcaaa 2119
```

```
Query: 185  tcgtaaatcaagaacaagctttcctgttccttctgacagcatcgtactcttactgatgct 244
            ||||||||||||||||||||||||||||||||||||||||||||||||||||||||||||
Sbjct: 2118 tcgtaaatcaagaacaagctttcctgttccttctgacagcatcgtactcttactgatgct 2059


Query: 245  aaccacaagtttttgattcgtcaactgcccagaaacataaccatttaacggtgtgtaata 304
            ||||||||||||||||||||||||||||||||||||||||||||||||||||||||||||
Sbjct: 2058 aaccacaagtttttgattcgtcaactgcccagaaacataaccatttaacggtgtgtaata 1999


Query: 305  tggttcaactgttaattgcaacgcagcgttatctacccaaacattatgctcttgattgat 364
            ||||||||||||||||||||||||||||||||||||||||||||||||||||||||||||
Sbjct: 1998 tggttcaactgttaattgcaacgcagcgttatctacccaaacattatgctcttgattgat 1939


Query: 365  gattccatcggcagtaaactcaatagatacctttgacaaatcaatcgttccaccaagtgc 424
            ||||||||||||||||||||||||||||||||||||||||||||||||||||||||||||
Sbjct: 1938 gattccatcggcagtaaactcaatagatacctttgacaaatcaatcgttccaccaagtgc 1879


Query: 425  atttattgtgtattgctggcttatcgtgttcccattgtttttcgtagttactactaccac 484
            ||||||||||||||||||||||||||||||||||||||||||||||||||||||||||||
Sbjct: 1878 atttattgtgtattgctggcttatcgtgttcccattgtttttcgtagttactactaccac 1819


Query: 485  cggttttgctccctccactggtactgtagtaggagttggtgtcggagctattgttggttt 544
            ||||||||||||||||||||||||||||||||||||||||||||||||||||||||||||
Sbjct: 1818 cggttttgctccctccactggtactgtagtaggagttggtgtcggagctattgttggttt 1759


Query: 545  tggcgttactgttggtgttatggtaggagttattgttggctttggtgttacagttggtgt 604
            ||||||||||||||||||||||||||||||||||||||||||||||||||||||||||||
Sbjct: 1758 tggcgttactgttggtgttatggtaggagttattgttggctttggtgttacagttggtgt 1699


Query: 605  tactgtaggtgttatcgttggttttggcgttacagttggtgttactgtaggtgttatcgt 664
            ||||||||||||||||||||||||||||||||||||||||||||||||||||||||||||
Sbjct: 1698 tactgtaggtgttatcgttggttttggcgttacagttggtgttactgtaggtgttatcgt 1639


Query: 665  tggttttggtgttacagttggagttattgatggcgttactgttggggtaatcccatcaat 724
            ||||||||||||||||||||||||||||||||||||||||||||||||||||||||||||
Sbjct: 1638 tggttttggtgttacagttggagttattgatggcgttactgttggggtaatcccatcaat 1579


Query: 725  gatagaatagtatgctgactttgcttgataatttccatcaaataataatggatatccacc 784
            ||||||||||||||||||||||||||||||||||||||||||||||||||||||||||||
Sbjct: 1578 gatagaatagtatgctgactttgcttgataatttccatcaaataataatggatatccacc 1519


Query: 785  taaccaactagtcttatccgttacaccccagaagataactcctgtaatgttcatgccctc 844
            ||||||||||||||||||||||||||||||||||||||||||||||||||||||||||||
Sbjct: 1518 taaccaactagtcttatccgttacaccccagaagataactcctgtaatgttcatgccctc 1459


Query: 845  nnnnnnncaaactaatgaccttatttatcaaaagtttatatctggctgcctgagcagcta 904
                   |||||||||||||||||||||||||||||||||||| |||||||||||||||
Sbjct: 1458 -ttttttcaaactaatgaccttatttatcaaaagtttatatctgtctgcctgagcagcta 1400
```

```
Query: 905  aagcagatgtactattgtctggcggctttatatctaactccgttagttgtaaattctaat 964
            ||||||||||||||||||||||| |||||||||||||||||||||||||| |||||||||
Sbjct: 1399 aagcagatgtactattgtctggctgctttatatctaactccgttagttgt-aattctaat 1341


Query: 965  ccaaaaggatta 976
            || |||| ||||
Sbjct: 1340 cctaaagtatta 1329

#######

Cphy1308 forward sequence

Query= HR0ADKBY80ZC02_CAZYD2C3P16_1.SCF   1327      0   1327  SCF
         (1327 letters)

>Cphy1308* forward

          Length = 3228

 Score = 1663 bits (839), Expect = 0.0
 Identities = 882/891 (98%), Gaps = 4/891 (0%)
 Strand = Plus / Plus


Query: 67   aaagaaggagataggatcATGgaaattacatccaatgcaacagtttatattaaaaatgct 126
           ||||||||||||||||||||||||||||||||||||||||||||||||||||||||||||
Sbjct: 1   aaagaaggagataggatcatggaaattacatccaatgcaacagtttatattaaaaatgct 60


Query: 127 accacaggttcttatctttatgcttctggttctagtgtaaatgctggaggttttcaagag 186
           ||||||||||||||||||||||||||||||||||||||||||||||||||||||||||||
Sbjct: 61  accacaggttcttatctttatgcttctggttctagtgtaaatgctggaggttttcaagag 120


Query: 187 aaaaacccatattacatatggagtataactaagaaaacggacggtacttttggattcaa 246
           ||||||||||||||||||||||||||||||||||||||||||||||||||||||||||||
Sbjct: 121 aaaaacccatattacatatggagtataactaagaaaacggacggtacttttggattcaa 180


Query: 247 aacatgggaacaaactcgtatttatgtttagaacatgcgagcggaaaagcactattagag 306
           ||||||||||||||||||||||||||||||||||||||||||||||||||||||||||||
Sbjct: 181 aacatgggaacaaactcgtatttatgtttagaacatgcgagcggaaaagcactattagag 240


Query: 307 actacaatatacgaagtatggatgagtagtaaatggtacatcgaaggtaatgcggattct 366
           ||||||||||||||||||||||||||||||||||||||||||||||||||||||||||||
Sbjct: 241 actacaatatacgaagtatggatgagtagtaaatggtacatcgaaggtaatgcggattct 300


Query: 367 tctacaattgataatatgtggaagagcacaagaagtagtggaactggtggttatttatat 426
           ||||||||||||||||||||||||||||||||||||||||||||||||||||||||||||
Sbjct: 301 tctacaattgataatatgtggaagagcacaagaagtagtggaactggtggttatttatat 360


Query: 427 acaaacagtggaaattcagatgtattctacggaacaacagcgcaacgatggatttttgaa 486
```

```
           ||||||||||||||||||||||||||||||||||||||||||||||||||||||||||||
Sbjct: 361 acaaacagtggaaattcagatgtattctacggaacaacagcgcaacgatggatttttgaa 420


Query: 487 gactacagttcaacttccaatattccagaggatggtgttaaaggtaaattaacaaataaa 546
           ||||||||||||||||||||||||||||||||||||||||||||||||||||||||||||
Sbjct: 421 gactacagttcaacttccaatattccagaggatggtgttaaaggtaaattaacaaataaa 480


Query: 547 gttgcagcagatgcaatcgtagttcctgctccaaacagcgatgtttcaagcattggtgcc 606
           ||||||||||||||||||||||||||||||||||||||||||||||||||||||||||||
Sbjct: 481 gttgcagcagatgcaatcgtagttcctgctccaaacagcgatgtttcaagcattggtgcc 540


Query: 607 acgatgccatatgtacgttatgattccgaatatgcagtattaggtggtggcgcaaggctt 666
           ||||||||||||||||||||||||||||||||||||||||||||||||||||||||||||
Sbjct: 541 acgatgccatatgtacgttatgattccgaatatgcagtattaggtggtggcgcaaggctt 600


Query: 667 gcaacctcaacgaattgggatttaacgaatattgctagccaggcatccaatcaatcttat 726
           ||||||||||||||||||||||||||||||||||||||||||||||||||||||||||||
Sbjct: 601 gcaacctcaacgaattgggatttaacgaatattgctagccaggcatccaatcaatcttat 660


Query: 727 gtcgttcttccatcgagcggttcttatgcggaatggagagtaaattcttctggtaatggt 786
           ||||||||||||||||||||||||||||||||||||||||||||||||||||||||||||
Sbjct: 661 gtcgttcttccatcgagcggttcttatgcggaatggagagtaaattcttctggtaatggt 720


Query: 787 gttgttatgagatttaccttaccggatactccgaacggaatgggacaaaatggttcctta 846
           ||||||||||||||||||||||||||||||||||||||||||||||||||||||||||||
Sbjct: 721 gttgttatgagatttaccttaccggatactccgaacggaatgggacaaaatggttcctta 780


Query: 847 aacgtatatgtgaatggctccaaagtaaaaaacagttaatttaacttcgtattatatgtg 906
            ||||||||||||||||||||||||| |||||||||||||||||| ||||||||||||||
Sbjct: 781 gacgtatatgtgaatggctccaaagt-aaaaacagttaatttaacctcgtattatatgtg 839


Query: 907 gcaatatttaaaatgggggtgcaaatgaaaaccccaggtgggtggcactgc 957
           ||||||||| |||||| ||||||| || | ||||||||| |||||||||||
Sbjct: 840 gcaatattt-aaatggtggtgcaagtg-ataccccaggt-ggtggcactgc 887
```

Cphy1308 reverse sequence

```
Query= HR0ADKBY80ZC02_CAZYD2C3P17_1.SCF   1337      0   1337  SCF

>Cphy1308* reverse

          Length = 3225

 Score = 1614 bits (814), Expect = 0.0
 Identities = 861/870 (98%), Gaps = 5/870 (0%)
 Strand = Plus / Minus


Query: 67   gtgtaatggatagtgatcTTAatggtgatggtgatgatgacgtactgtcatcgttttcgt 126
```

```
            ||||||||||||||||||||||||||||||||||||||||||||||||||||||||||||
Sbjct: 3225 gtgtaatggatagtgatcttaatggtgatggtgatgatgacgtactgtcatcgttttcgt 3166


Query: 127  caaagtgttattatctgtattggattctggataccgattaatatcattgacccaagccat 186
            ||||||||||||||||||||||||||||||||||||||||||||||||||||||||||||
Sbjct: 3165 caaagtgttattatctgtattggattctggataccgattaatatcattgacccaagccat 3106


Query: 187  aacattaaaggtacctgccgtagcagtataggttggactgccagctggaccactatttgc 246
            ||||||||||||||||||||||||||||||||||||||||||||||||||||||||||||
Sbjct: 3105 aacattaaaggtacctgccgtagcagtataggttggactgccagctggaccactatttgc 3046


Query: 247  tgttaccgtcactgactgtcctggcattatctgagttgtattgttatcggaccaagaaac 306
            ||||||||||||||||||||||||||||||||||||||||||||||||||||||||||||
Sbjct: 3045 tgttaccgtcactgactgtcctggcattatctgagttgtattgttatcggaccaagaaac 2986


Query: 307  acaggtaccattcacttggaattgtaatccgttgattgcccctactggagctactccggt 366
            ||||||||||||||||||||||||||||||||||||||||||||||||||||||||||||
Sbjct: 2985 acaggtaccattcacttggaattgtaatccgttgattgcccctactggagctactccggt 2926


Query: 367  tccttgattcttaataactgcactaaatgtgatcttattcccgcttactgggcttgctgg 426
            ||||||||||||||||||||||||||||||||||||||||||||||||||||||||||||
Sbjct: 2925 tccttgattcttaataactgcactaaatgtgatcttattcccgcttactgggcttgctgg 2866


Query: 427  agaccaagtaatatccgttacaaccatatctggattaccataaggttgccccgttggggt 486
            ||||||||||||||||||||||||||||||||||||||||||||||||||||||||||||
Sbjct: 2865 agaccaagtaatatccgttacaaccatatctggattaccataaggttgccccgttggggt 2806


Query: 487  aggtgtcggtgtaaccgttggttgtaccgctccttctgttacagtaagtggttttgttaa 546
            ||||||||||||||||||||||||||||||||||||||||||||||||||||||||||||
Sbjct: 2805 aggtgtcggtgtaaccgttggttgtaccgctccttctgttacagtaagtggttttgttaa 2746


Query: 547  catattattattggtattagactcaggataacgattaacatcgtttacccatgcagtaat 606
            ||||||||||||||||||||||||||||||||||||||||||||||||||||||||||||
Sbjct: 2745 catattattattggtattagactcaggataacgattaacatcgtttacccatgcagtaat 2686


Query: 607  ggtataggtacctgtcttcgctgcccatgttgcagagccagaaggcccacttacggctgt 666
            ||||||||||||||||||||||||||||||||||||||||||||||||||||||||||||
Sbjct: 2685 ggtataggtacctgtcttcgctgcccatgttgcagagccagaaggcccacttacggctgt 2626


Query: 667  tactgtaatggattgacctggttgaatctgagttttgtcattatctgaccaagaaacact 726
            ||||||||||||||||||||||||||||||||||||||||||||||||||||||||||||
Sbjct: 2625 tactgtaatggattgacctggttgaatctgagttttgtcattatctgaccaagaaacact 2566


Query: 727  agtaccatttacttgaaattgaactccattaattgatccaagtggcgctacccccagttc 786
            |||||||||||||||||||||||||||||||||||||||||||||||| |||||||||
Sbjct: 2565 agtaccatttacttgaaattgaactccattaattgatccaagtggcgcta-ccccagttc 2507
```

```
Query: 787  ctatattttaataactgcactaaaagtaactttgattgccatttggttggatttgctgg 846
            |||||||||||||||||||||||||||||| |||||||||| |||||||||||||||||
Sbjct: 2506 ctatattttaataactgcactaaaagtaac-ttgattgcca-ttggttggatttgctgg 2449


Query: 847  tgaccacgtaatatctgtaacaatcatatctggattaccatatggaacagaaagggtttg 906
            |||||||||||||||||||||||||||||||||||||||||||||||||| ||||  |||
Sbjct: 2448 tgaccacgtaatatctgtaacaatcatatctggattaccatatggaacag-aaggtgttg 2390


Query: 907  gctttactgttggtgaatggggtagctgtt 936
            || |||||||||||| |||| |||||||||
Sbjct: 2389 gcgttactgttggtg-atggtgtagctgtt 2361

#######

Cphy1888 forward sequence

Query= HR0ADKBY80ZE02_CAZYE1C1P16_1.SCF   1343      0   1343  SCF

>Cphy1888* forward

          Length = 1803

 Score = 1542 bits (778), Expect = 0.0
 Identities = 818/826 (99%), Gaps = 4/826 (0%)
 Strand = Plus / Plus


Query: 69  aaagaaggagataggatcATGacgacgacaacacatgaattaaatgtatcaaattcaatg 128
           ||||||||||||||||||||||||||||||||||||||||||||||||||||||||||||
Sbjct: 1   aaagaaggagataggatcatgacgacgacaacacatgaattaaatgtatcaaattcaatg 60


Query: 129 acagtaggacaatactcatcagattttactttaaatggatttacatttattacaggagga 188
           ||||||||||||||||||||||||||||||||||||||||||||||||||||||||||||
Sbjct: 61  acagtaggacaatactcatcagattttactttaaatggatttacatttattacaggagga 120


Query: 189 tctatatgggaagtagatagctctagccggtcttacggtggtgtaaactttacgcagaga 248
           ||||||||||||||||||||||||||||||||||||||||||||||||||||||||||||
Sbjct: 121 tctatatgggaagtagatagctctagccggtcttacggtggtgtaaactttacgcagaga 180


Query: 249 gttaaatcgggcggaaagggtaccattagtaaaagagcaatctcatttacagcttctgga 308
           ||||||||||||||||||||||||||||||||||||||||||||||||||||||||||||
Sbjct: 181 gttaaatcgggcggaaagggtaccattagtaaaagagcaatctcatttacagcttctgga 240


Query: 309 gcaggtcaattaacagtttatgcaatgagttctggaagtacttcaagaaatgtaacgctt 368
           ||||||||||||||||||||||||||||||||||||||||||||||||||||||||||||
Sbjct: 241 gcaggtcaattaacagtttatgcaatgagttctggaagtacttcaagaaatgtaacgctt 300


Query: 369 tatgggaacggtaaggatctagaaagctttactgcagtgcaagatgtcattactgctatg 428
           ||||||||||||||||||||||||||||||||||||||||||||||||||||||||||||
Sbjct: 301 tatgggaacggtaaggatctagaaagctttactgcagtgcaagatgtcattactgctatg 360
```

```
Query: 429 aattttacgattccaaattccggtacttatgttatttatccaccagacgatggtattagc 488
           ||||||||||||||||||||||||||||||||||||||||||||||||||||||||||||
Sbjct: 361 aattttacgattccaaattccggtacttatgttatttatccaccagacgatggtattagc 420


Query: 489 tactactatttaaaggtagttaaaaccgactcaacacctacaccaactccaacagtaaaa 548
           ||||||||||||||||||||||||||||||||||||||||||||||||||||||||||||
Sbjct: 421 tactactatttaaaggtagttaaaaccgactcaacacctacaccaactccaacagtaaaa 480


Query: 549 ccgacccaaacacctacaccaactccaactacatcaaatgaagtttacgtctcaaacaat 608
           ||||||||||||||||||||||||||||||||||||||||||||||||||||||||||||
Sbjct: 481 ccgacccaaacacctacaccaactccaactacatcaaatgaagtttacgtctcaaacaat 540


Query: 609 ggttctgcaagtgcaagtggtacatattccaatccaaaatccttagagggagcgattagt 668
           ||||||||||||||||||||||||||||||||||||||||||||||||||||||||||||
Sbjct: 541 ggttctgcaagtgcaagtggtacatattccaatccaaaatccttagagggagcgattagt 600


Query: 669 tcggcgaaagctggacaaaccattttatgttaccaggtacttattcttatagtacacaa 728
           |||||||||||||||||||||||||||||||||||||||||||||||||||||||||| |
Sbjct: 601 tcggcgaaagctggacaaaccattttatgttaccaggtacttattcttatagtacac-a 659


Query: 729 aataacaattccagcaggaaccaatggtacatccagtgcacgaattaaattaatgccata 788
           ||||||||||||||||||||||||||||||||||||||||||||||||||||||||||||
Sbjct: 660 aataacaattccagcaggaaccaatggtacatccagtgcacgaattaaattaatgccata 719


Query: 789 caaataatggtagtgtaactcttaatttcctcatctcacccatacggtaatccggatact 848
           | |||||||||||||||||||||||||| ||||||||| |||||||||||||| ||||||
Sbjct: 720 c-aataatggtagtgtaactcttaattt-ctcatctcagccatacggtaatcctgatact 777


Query: 849 aatttacgaagtattcaactggatgcaaatttttgggcatatctat 894
           ||||||||| ||||||||||||||||||||| || |||||||||||
Sbjct: 778 aatttacgaggtattcaactggatgcaaattatt-ggcatatctat 822

Cphy1888 reverse sequence
Query= HR0ADKBY80ZE02_CAZYE1C1P17_1.SCF   1345      0   1345  SCF

>Cphy1888* reverse

          Length = 1803

 Score = 1556 bits (785), Expect = 0.0
 Identities = 839/849 (98%), Gaps = 6/849 (0%)
 Strand = Plus / Minus


Query: 66   gtgtaatggatagtgatcTTAatggtgatggtgatgatgatttcctagattagctcccat 125
            ||||||||||||||||||||||||||||||||||||||||||||||||||||||||||||
Sbjct: 1803 gtgtaatggatagtgatcttaatggtgatggtgatgatgatttcctagattagctcccat 1744
```

```
Query: 126  tgtcttgtatgcagaagaactacttaactttaagaaattacctaagttaatagaaccgtc 185
            ||||||||||||||||||||||||||||||||||||||||||||||||||||||||||||
Sbjct: 1743 tgtcttgtatgcagaagaactacttaactttaagaaattacctaagttaatagaaccgtc 1684


Query: 186  ggaatttcttaatgtttgatgtaagtttgaactactagctggagctgataaggacacaaa 245
            ||||||||||||||||||||||||||||||||||||||||||||||||||||||||||||
Sbjct: 1683 ggaatttcttaatgtttgatgtaagtttgaactactagctggagctgataaggacacaaa 1624


Query: 246  gtctgaggtcttaacacctgttgaatttaatgttccataaattccacctcttgtatttac 305
            ||||||||||||||||||||||||||||||||||||||||||||||||||||||||||||
Sbjct: 1623 gtctgaggtcttaacacctgttgaatttaatgttccataaattccacctcttgtatttac 1564


Query: 306  acttgtagccgttgttataagataatacttagaactattataaaaactacagttcgtagc 365
            ||||||||||||||||||||||||||||||||||||||||||||||||||||||||||||
Sbjct: 1563 acttgtagccgttgttataagataatacttagaactattataaaaactacagttcgtagc 1504


Query: 366  tgttcccttatatttgtcacttgaaacaggctttgtactgaatgtaataatattggtaaa 425
            ||||||||||||||||||||||||||||||||||||||||||||||||||||||||||||
Sbjct: 1503 tgttcccttatatttgtcacttgaaacaggctttgtactgaatgtaataatattggtaaa 1444


Query: 426  ataattgttggatgtatccgctcttgcaaaatcaaaattagacttatttccatccgctaa 485
            ||||||||||||||||||||||||||||||||||||||||||||||||||||||||||||
Sbjct: 1443 ataattgttggatgtatccgctcttgcaaaatcaaaattagacttatttccatccgctaa 1384


Query: 486  actattattaaaagaagtacaatttttagtgtaataggacctggattagaattatcggt 545
            ||||||||||||||||||||||||||||||||||||||||||||||||||||||||||||
Sbjct: 1383 actattattaaaagaagtacaatttttagtgtaataggacctggattagaattatcggt 1324


Query: 546  aaagccatggttttattttcaaatgcgatacaattttctacggtgtgagctactgcgat 605
            ||||||||||||||||||||||||||||||||||||||||||||||||||||||||||||
Sbjct: 1323 aaagccatggttttattttcaaatgcgatacaattttctacggtgtgagctactgcgat 1264


Query: 606  acttgagccaccgagcttaaatccattaccgtcactatttgcagtgaaaacacctgtact 665
            ||||||||||||||||||||||||||||||||||||||||||||||||||||||||||||
Sbjct: 1263 acttgagccaccgagcttaaatccattaccgtcactatttgcagtgaaaacacctgtact 1204


Query: 666  tgttgcaccgttacggaatgaaacacagtttctcaaagttatactaccaatcgctccggt 725
            ||||||||||||||||||||||||||||||||||||||||||||||||||||||||||||
Sbjct: 1203 tgttgcaccgttacggaatgaaacacagtttctcaaagttatactaccaatcgctccggt 1144


Query: 726  atctgttttcgtatataaatcccaaccatcatctacgttattgtatgccatacatccatc 785
            ||||||||||||||||||||||||||||||||||||||||||||||||||||||||||||
Sbjct: 1143 atctgttttcgtatataaatcccaaccatcatctacgttattgtatgccatacatccatc 1084


Query: 786  aaacacatttccctcctcccaagtttaattttgcagcaaaatccatctgcattctcacca 845
            |||||||||||||||  | |||| ||||||||||||| ||||||||||||||||||||||
Sbjct: 1083 aaacacatttccctctccacaag-ttaattttgcagc-aaatccatctgcattctcacca 1026
```

```
Query: 846  gtagcctggatcgtaattggtaaagggaggtaccagtttttaatggaggttattggaagg 905
            |||| |||||||||||||| |||| ||||||| ||||||||||| |||||||||||||||
Sbjct: 1025 gtag-ctggatcgtaattgttaaa-ggaggta-cagtttttaat-gaggttattggaagg 970


Query: 906  ccaacctga 914
            |||||||||
Sbjct: 969  ccaacctga 961
```

#######

Cphy1929 forward sequence

```
Query= HR0ADKBY80ZH02_CAZYF1C4P16_1.SCF   1319      0   1319  SCF

>Cphy1929 forward

          Length = 2448

 Score = 1562 bits (788), Expect = 0.0
 Identities = 831/840 (98%), Gaps = 4/840 (0%)
 Strand = Plus / Plus


Query: 69  aaagaaggagataggatcATGaattacggttacttcgacgaagcaaacagagaatatgta 128
           ||||||||||||||||||||||||||||||||||||||||||||||||||||||||||||
Sbjct: 1   aaagaaggagataggatcatgaattacggttacttcgacgaagcaaacagagaatatgta 60


Query: 129 attacaaaaccgaataccccagctccatgggcaaattatttgggttcccccgagtatggt 188
           ||||||||||||||||||||||||||||||||||||||||||||||||||||||||||||
Sbjct: 61  attacaaaaccgaataccccagctccatgggcaaattatttgggttcccccgagtatggt 120


Query: 189 gctattatatcgaataacgcaggtggctatagctttgtgaagagtggtgcaaatggtaga 248
           ||||||||||||||||||||||||||||||||||||||||||||||||||||||||||||
Sbjct: 121 gctattatatcgaataacgcaggtggctatagctttgtgaagagtggtgcaaatggtaga 180


Query: 249 atatcaagatatatattcaatcaggaagacaaaccaggaagatatatttatttacgtgat 308
           ||||||||||||||||||||||||||||||||||||||||||||||||||||||||||||
Sbjct: 181 atatcaagatatatattcaatcaggaagacaaaccaggaagatatatttatttacgtgat 240


Query: 309 gatgtatcaggggattattggtcagcatcatggcagccagttggtaaagatttatcggtt 368
           ||||||||||||||||||||||||||||||||||||||||||||||||||||||||||||
Sbjct: 241 gatgtatcaggggattattggtcagcatcatggcagccagttggtaaagatttatcggtt 300


Query: 369 tatcaaaatgaatgccgtcatggtaccgcttataccaaaattagtgctgattattctaat 428
           ||||||||||||||||||||||||||||||||||||||||||||||||||||||||||||
Sbjct: 301 tatcaaaatgaatgccgtcatggtaccgcttataccaaaattagtgctgattattctaat 360


Query: 429 attcattccgaagctttatattatgttcctttaaacaagacacatgaagtttggaagttg 488
           ||||||||||||||||||||||||||||||||||||||||||||||||||||||||||||
```

```
Sbjct: 361 attcattccgaagctttatattatgttcctttaaacaagacacatgaagtttggaagttg 420


Query: 489 tctgtaaccaataccgacaataaagaacgaaaaatttccaccttcggcttcattgaattt 548
           ||||||||||||||||||||||||||||||||||||||||||||||||||||||||||||
Sbjct: 421 tctgtaaccaataccgacaataaagaacgaaaaatttccaccttcggcttcattgaattt 480


Query: 549 acgaatgaggactattatgaaaatgatcaagttaatctccagtatacactctttattacc 608
           ||||||||||||||||||||||||||||||||||||||||||||||||||||||||||||
Sbjct: 481 acgaatgaggactattatgaaaatgatcaagttaatctccagtatacactctttattacc 540


Query: 609 aaaacttattttaaagagaataagatcctacaaacaattaacgaaaatactggcaagaat 668
           ||||||||||||||||||||||||||||||||||||||||||||||||||||||||||||
Sbjct: 541 aaaacttattttaaagagaataagatcctacaaacaattaacgaaaatactggcaagaat 600


Query: 669 gcagaaggtacgaattaccgtgaacgtttctttggtagtgtaggtcaacctattgtttcc 728
           ||||||||||||||||||||||||||||||||||||||||||||||||||||||||||||
Sbjct: 601 gcagaaggtacgaattaccgtgaacgtttctttggtagtgtaggtcaacctattgtttcc 660


Query: 729 tataatggtgacaaggcatcctttattgggcgttatcatagttatggaaatccagttgcc 788
           ||||||||||||||||||||||||||||||||||||||||||||||||||||||||||||
Sbjct: 661 tataatggtgacaaggcatcctttattgggcgttatcatagttatggaaatccagttgcc 720


Query: 789 ggtatcaatggatgctgcgataatactttaaaattataattctaacgcatgcggtgccct 848
           | |||||||||||||||||||||||||| |||||||||||||||||||||||||||||||
Sbjct: 721 gttatcaatggatgctgcgataatacttt-aaattataattctaacgcatgcggtgccct 779


Query: 849 tcactcccata-taactcttgcccctggtgaaacaaaaggaattataaaacatattaggt 907
           ||||| ||||| |||||||||| ||||||||||| ||||||||||  | |||||||||||
Sbjct: 780 tcact-ccatattaactcttgcacctggtgaaac-aaaggaattagtatacatattaggt 837

Cphy1929 reverse sequence



Query= HR0ADKBY80ZH02_CAZYF1C4P17_1.SCF   1333      0   1333  SCF

>Cphy1929 reverse

          Length = 2448

 Score = 1606 bits (810), Expect = 0.0
 Identities = 836/842 (99%), Gaps = 2/842 (0%)
 Strand = Plus / Minus


Query: 68   gtgtaatggatagtgatcTTAatggtgatggtgatgatgcattactaataatacttcatt 127
            ||||||||||||||||||||||||||||||||||||||||||||||||||||||||||||
Sbjct: 2448 gtgtaatggatagtgatcttaatggtgatggtgatgatgcattactaataatacttcatt 2389


Query: 128  ttctacagatagtgattccgcaggaatgtaatttgaagaaagtattgttccatttaagta 187
```

```
           ||||||||||||||||||||||||||||||||||||||||||||||||||||||||||||
Sbjct: 2388 ttctacagatagtgattccgcaggaatgtaatttgaagaaagtattgttccatttaagta 2329

Query: 188  acattcgcggtaaccaccttctgcaccatcaaagttttgtatcgtaatatttaacttctt 247
            ||||||||||||||||||||||||||||||||||||||||||||||||||||||||||||
Sbjct: 2328 acattcgcggtaaccaccttctgcaccatcaaagttttgtatcgtaatatttaacttctt 2269

Query: 248  attgcggaacatcttagtaattttaacttctttccaattggatggtatggaaggggagag 307
            ||||||||||||||||||||||||||||||||||||||||||||||||||||||||||||
Sbjct: 2268 attgcggaacatcttagtaattttaacttctttccaattggatggtatggaaggggagag 2209

Query: 308  taatagaccatcgaagttaggacgtatacctaagataccttcgacgcagccaaccatcac 367
            ||||||||||||||||||||||||||||||||||||||||||||||||||||||||||||
Sbjct: 2208 taatagaccatcgaagttaggacgtatacctaagataccttcgacgcagccaaccatcac 2149

Query: 368  agttgatgcagttcctgtcaaccagtgtacatgggatcggccttcaaatgggctatcaac 427
            ||||||||||||||||||||||||||||||||||||||||||||||||||||||||||||
Sbjct: 2148 agttgatgcagttcctgtcaaccagtgtacatgggatcggccttcaaatgggctatcaac 2089

Query: 428  cgattctgtaaattgcccatgaacatatggttcaagaacacggacatctgcattatcatt 487
            ||||||||||||||||||||||||||||||||||||||||||||||||||||||||||||
Sbjct: 2088 cgattctgtaaattgcccatgaacatatggttcaagaacacggacatctgcattatcatt 2029

Query: 488  ctgagaagatggagagctctccttaaaatactgataggcgcaatcaccatgacccattaa 547
            ||||||||||||||||||||||||||||||||||||||||||||||||||||||||||||
Sbjct: 2028 ctgagaagatggagagctctccttaaaatactgataggcgcaatcaccatgacccattaa 1969

Query: 548  ggattctgcaaggatgatccaaccttgtggctgcgagaagataccaccattctctttggt 607
            ||||||||||||||||||||||||||||||||||||||||||||||||||||||||||||
Sbjct: 1968 ggattctgcaaggatgatccaaccttgtggctgcgagaagataccaccattctctttggt 1909

Query: 608  tgatttattaaataagatagcaagagcaccgtcaaatgcatggtcaacgtaggatggagc 667
            ||||||||||||||||||||||||||||||||||||||||||||||||||||||||||||
Sbjct: 1908 tgatttattaaataagatagcaagagcaccgtcaaatgcatggtcaacgtaggatggagc 1849

Query: 668  cattaagcggcatccatacgctgtatttaattcgcgatgaacactctctaatgctttttc 727
            ||||||||||||||||||||||||||||||||||||||||||||||||||||||||||||
Sbjct: 1848 cattaagcggcatccatacgctgtatttaattcgcgatgaacactctctaatgctttttc 1789

Query: 728  agcttggtccttagtagcaagaccactaatcactgaccatgactgtggatttagccacat 787
            ||||||||||||||||||||||||||||||||||||||||||||||||||||||||||||
Sbjct: 1788 agcttggtccttagtagcaagaccactaatcactgaccatgactgtggatttagccacat 1729

Query: 788  atttgcttctggatctttcttagaaccgattacttgaccatcttctttaaagccacggat 847
            ||||||||||||||||||||||||||||||||||||||||||||||||||||||||||||
Sbjct: 1728 atttgcttctggatctttcttagaaccgattacttgaccatcttctttaaagccacggat 1669
```

```
Query: 848  aaaacgaatcttcttccaaacaatatttctgggagggttccaccaagttctggttgtact 907
            |||||| |||||||||| |||||||||||| ||| |||| |||||||||||| |||||||
Sbjct: 1668 aaaacg-atcttcttcccaacaatatttct-ggatggtttcaccaagttctgtttgtact 1611

#######

Cphy2128 forward sequence

Query= HR0ADKBY80ZD03_CAZYG2C4P16_1.SCF   1311      0   1311  SCF
         (1311 letters)

>Cphy2128* forward

          Length = 2544

 Score = 1532 bits (773), Expect = 0.0
 Identities = 793/797 (99%), Gaps = 2/797 (0%)
 Strand = Plus / Plus


Query: 66  aaagaaggagataggatcATGactgcgttgttcgttatcgaaagcgaaaatacgcgacta 125
           ||||||||||||||||||||||||||||||||||||||||||||||||||||||||||||
Sbjct: 1   aaagaaggagataggatcatgactgcgttgttcgttatcgaaagcgaaaatacgcgacta 60


Query: 126 tcatctgagttacaagttgtgacggcaatatatggacaaccaaagcctggatactcggga 185
           ||||||||||||||||||||||||||||||||||||||||||||||||||||||||||||
Sbjct: 61  tcatctgagttacaagttgtgacggcaatatatggacaaccaaagcctggatactcggga 120


Query: 186 gcaggatttgtttggatgcagaactcaggtacattaactttcgatgtcactgttccagag 245
           ||||||||||||||||||||||||||||||||||||||||||||||||||||||||||||
Sbjct: 121 gcaggatttgtttggatgcagaactcaggtacattaactttcgatgtcactgttccagag 180


Query: 246 acaggtatgtatacgatatctactcgttatatgcaagagttaagtacggatggcagactc 305
           ||||||||||||||||||||||||||||||||||||||||||||||||||||||||||||
Sbjct: 181 acaggtatgtatacgatatctactcgttatatgcaagagttaagtacggatggcagactc 240


Query: 306 caatacttaagcgtaaatggtatcaataaaggttcctttatgttgccatatactacagtg 365
           ||||||||||||||||||||||||||||||||||||||||||||||||||||||||||||
Sbjct: 241 caatacttaagcgtaaatggtatcaataaaggttcctttatgttgccatatactacagtg 300


Query: 366 tggtcagattttaattttggtatttataaattgaccaaaggtagcaacacgatacaaatt 425
           ||||||||||||||||||||||||||||||||||||||||||||||||||||||||||||
Sbjct: 301 tggtcagattttaattttggtatttataaattgaccaaaggtagcaacacgatacaaatt 360


Query: 426 aaggctggttggggattcgcttattttgatacattcaccgtggattatgctaaactagat 485
           ||||||||||||||||||||||||||||||||||||||||||||||||||||||||||||
Sbjct: 361 aaggctggttggggattcgcttattttgatacattcaccgtggattatgctaaactagat 420


Query: 486 agtttaaatgtagcgccaacccttaccgatagccaggcaacatcagaaacaaaacttcta 545
           ||||||||||||||||||||||||||||||||||||||||||||||||||||||||||||
```

```
Sbjct: 421 agtttaaatgtagcgccaacccttaccgatagccaggcaacatcagaaacaaaacttcta 480


Query: 546 atgaaatacttaacggaggtttacggtaaacatatcatctctggccaacaagagatctat 605
           ||||||||||||||||||||||||||||||||||||||||||||||||||||||||||||
Sbjct: 481 atgaaatacttaacggaggtttacggtaaacatatcatctctggccaacaagagatctat 540


Query: 606 ggtggtggaaataacggtaattcagaattagagtttgattggattcacaatttaacaggt 665
           ||||||||||||||||||||||||||||||||||||||||||||||||||||||||||||
Sbjct: 541 ggtggtggaaataacggtaattcagaattagagtttgattggattcacaatttaacaggt 600


Query: 666 aagtatccagcgattagagggtttgattttatgaactataatccattatacggttgggaa 725
           ||||||||||||||||||||||||||||||||||||||||||||||||||||||||||||
Sbjct: 601 aagtatccagcgattagagggtttgattttatgaactataatccattatacggttgggaa 660


Query: 726 gatggtacaacaaatcgtattatagattgggttaataataaaaatggtattgcaacggga 785
           ||||||||||||||||||||||||||||||||||||||||||||||||||||||||||||
Sbjct: 661 gatggtacaacaaatcgtattatagattgggttaataataaaaatggtattgcaacggga 720


Query: 786 tgctggcatattactgttccaaaagattttaattcctataagcctgggtgattttgtggg 845
           |||||||||||||||||||||||||||||||||||||||||| || ||||||||||| ||
Sbjct: 721 tgctggcatattactgttccaaaagattttaattcctataag-cttggtgattttgt-gg 778


Query: 846 atgggcaaaagacaacc 862
           || ||||||||||||||
Sbjct: 779 attggcaaaagacaacc 795


Cphy2128 reverse sequence


Query= HR0ADKBY80ZD03_CAZYG2C4P17_1.SCF   1319      0   1319  SCF

>Cphy2128* reverse

          Length = 2544

 Score = 1542 bits (778), Expect = 0.0
 Identities = 825/834 (98%), Gaps = 5/834 (0%)
 Strand = Plus / Minus


Query: 65   gtgtaatggatagtgatcTTAatggtgatggtgatgatgtggctcgattccccatacaag 124
            ||||||||||||||||||||||||||||||||||||||||||||||||||||||||||||
Sbjct: 2544 gtgtaatggatagtgatcttaatggtgatggtgatgatgtggctcgattccccatacaag 2485


Query: 125  cttattgcttaaatatcccgtaactttgtcagaattaacgtattggctactggatgcatt 184
            ||||||||||||||||||||||||||||||||||||||||||||||||||||||||||||
Sbjct: 2484 cttattgcttaaatatcccgtaactttgtcagaattaacgtattggctactggatgcatt 2425


Query: 185  aaatgagtaatcatctgcttggttgtaatttgaccaattcgacttagcaaatcttgcttg 244
```

```
           ||||||||||||||||||||||||||||||||||||||||||||||||||||||||||||
Sbjct: 2424 aaatgagtaatcatctgcttggttgtaatttgaccaattcgacttagcaaatcttgcttg 2365


Query: 245  aatttctgcattttgcccagcttttaacgttccagcagaagcagtaaaggaaatctccaa 304
            ||||||||||||||||||||||||||||||||||||||||||||||||||||||||||||
Sbjct: 2364 aatttctgcattttgcccagcttttaacgttccagcagaagcagtaaaggaaatctccaa 2305


Query: 305  gtaataatcagctccgctaactggtttagcaagcttaacaaattcaccttttacgttttc 364
            ||||||||||||||||||||||||||||||||||||||||||||||||||||||||||||
Sbjct: 2304 gtaataatcagctccgctaactggtttagcaagcttaacaaattcaccttttacgttttc 2245


Query: 365  actaccaacacttgcccaatcacaccagaagttctgtgatatttctccatcaatcgtata 424
            ||||||||||||||||||||||||||||||||||||||||||||||||||||||||||||
Sbjct: 2244 actaccaacacttgcccaatcacaccagaagttctgtgatatttctccatcaatcgtata 2185


Query: 425  gtaataacgaagttttatatcacttagattgatatctgtgtttccagtgttaatcagctt 484
            ||||||||||||||||||||||||||||||||||||||||||||||||||||||||||||
Sbjct: 2184 gtaataacgaagttttatatcacttagattgatatctgtgtttccagtgttaatcagctt 2125


Query: 485  aaatttaggtgatattccattggtggatgcgctcgtatttccgttaaaggcttgaattac 544
            ||||||||||||||||||||||||||||||||||||||||||||||||||||||||||||
Sbjct: 2124 aaatttaggtgatattccattggtggatgcgctcgtatttccgttaaaggcttgaattac 2065


Query: 545  taaatttcctgtaattacgggagcagtggtatctatgacattcaatgttaatacagggtt 604
            ||||||||||||||||||||||||||||||||||||||||||||||||||||||||||||
Sbjct: 2064 taaatttcctgtaattacgggagcagtggtatctatgacattcaatgttaatacagggtt 2005


Query: 605  gtttccagcactaaattggaatgttaatttgctttccccaagtgatagtgtagatagata 664
            ||||||||||||||||||||||||||||||||||||||||||||||||||||||||||||
Sbjct: 2004 gtttccagcactaaattggaatgttaatttgctttccccaagtgatagtgtagatagata 1945


Query: 665  ggattttgtaaaacaattgtattgcccgataccgtataatctttatttagtaaaaagtt 724
            ||||||||||||||||||||||||||||||||||||||||||||||||||| |||||||
Sbjct: 1944 ggattttgtaaaacaattgtattgcccgataccgtataatctttatttagt-aaaagtt 1886


Query: 725  tattactattatttagaacacttacaagcgaaagcccatttaagtttgaccgttacgttg 784
            |||||||||||||||||||||||||||||||||||||||||||| |||||||||||||||
Sbjct: 1885 tattactattatttagaacacttacaagcgaaagcccatttaag-ttgaccgttacgttg 1827


Query: 785  atatttgctgcttatcaactgctttatcaaaggttgcatttaaccgagtaattgtaagc 844
            |||||||||||||||||||||||||||||||||||||||| | | |||||||||| |||
Sbjct: 1826 atatttgctgcttatcaactgctttatcaaaggttgcattaaccggagtaattgt-agc 1768


Query: 845  actttggcgttgaatctacaacattaacttttaaaaccaggatctttccctttg 898
            || |||||||||||||||| |||||||||||||||| |||||||||||||||||
Sbjct: 1767 ac-ttggcgttgaatctacgacattaacttttaaaa-caggatctttccctttg 1716
```

```
########

Cphy3009 forward sequence

Query= HR0ADKBY80ZG03_CAZYH2C3P16_1.SCF   1252      0   1252  SCF

>Cphy3009
          Length = 2208

 Score = 1741 bits (878), Expect = 0.0
 Identities = 934/953 (98%), Gaps = 2/953 (0%)
 Strand = Plus / Plus


Query: 68   aaagaaggagataggatcATGacagcagagaagacacaacaagaatttcaacaacgagca 127
            ||||||||||||||||||||||||||||||||||||||||||||||||||||||||||||
Sbjct: 1    aaagaaggagataggatcatgacagcagagaagacacaacaagaatttcaacaacgagca 60


Query: 128  acagaacttgtaaagaaaatgacactagaagaaaaagtttttcaaaccttacattccgca 187
            ||||||||||||||||||||||||||||||||||||||||||||||||||||||||||||
Sbjct: 61   acagaacttgtaaagaaaatgacactagaagaaaaagtttttcaaaccttacattccgca 120


Query: 188  ccttccataccaagattggacattaaagcgtacaattattggaacgaagcattgcatgga 247
            ||||||||||||||||||||||||||||||||||||||||||||||||||||||||||||
Sbjct: 121  ccttccataccaagattggacattaaagcgtacaattattggaacgaagcattgcatgga 180


Query: 248  gtagcaagagctggcgtagccaccgtttttccgcaggcgattggtttagcggcaacattt 307
            ||||||||||||||||||||||||||||||||||||||||||||||||||||||||||||
Sbjct: 181  gtagcaagagctggcgtagccaccgtttttccgcaggcgattggtttagcggcaacattt 240


Query: 308  gatgaagatttaatcgaagagattgcagatactatttctaccgaaggaagaggtaagttt 367
            ||||||||||||||||||||||||||||||||||||||||||||||||||||||||||||
Sbjct: 241  gatgaagatttaatcgaagagattgcagatactatttctaccgaaggaagaggtaagttt 300


Query: 368  aatgcacaacaaaaatatggagatcacgatatctacaaaggtttgaccttctggtcaccg 427
            ||||||||||||||||||||||||||||||||||||||||||||||||||||||||||||
Sbjct: 301  aatgcacaacaaaaatatggagatcacgatatctacaaaggtttgaccttctggtcaccg 360


Query: 428  aatgtgaatatcttccgtgatccacgttggggtcgtggacatgagaccttcggagaagac 487
            ||||||||||||||||||||||||||||||||||||||||||||||||||||||||||||
Sbjct: 361  aatgtgaatatcttccgtgatccacgttggggtcgtggacatgagaccttcggagaagac 420


Query: 488  ccattcttaagtggtaccttaggaggacgttttgttgacggtatccaagggcatgatgaa 547
            ||||||||||||||||||||||||||||||||||||||||||||||||||||||||||||
Sbjct: 421  ccattcttaagtggtaccttaggaggacgttttgttgacggtatccaagggcatgatgaa 480


Query: 548  acctacttaaaagcagcagcttgtgcaaagcatttcgcagttcactctggaccagaagat 607
            ||||||||||||||||||||||||||||||||||||||||||||||||||||||||||||
Sbjct: 481  acctacttaaaagcagcagcttgtgcaaagcatttcgcagttcactctggaccagaagat 540
```

```
Query: 608  attcgccatagtttcaatgccgaagtttcggagcaggatttgcgtgaaacttatctacca 667
            ||||||||||||||||||||||||||||||||||||||||||||||||||||||||||||
Sbjct: 541  attcgccatagtttcaatgccgaagtttcggagcaggatttgcgtgaaacttatctacca 600


Query: 668  gcttttaagaaacttgtaaaagagcataaggtggaagcagtaatgggagcttacaatcgt 727
            ||||||||||||||||||||||||||||||||||||||||||||||||||||||||||||
Sbjct: 601  gcttttaagaaacttgtaaaagagcataaggtggaagcagtaatgggagcttacaatcgt 660


Query: 728  acaaatggagaaccatgctgtggtagtaagacacttttagaagatattttacgtggagaa 787
            ||||||||||||||||||||||||||||||||||||||||||||||||||||||||||||
Sbjct: 661  acaaatggagaaccatgctgtggtagtaagacacttttagaagatattttacgtggagaa 720


Query: 788  tgggaatttgttggtcatgtgacctcagattgttgggcaatcaaagatttccatgagcac 847
            ||||||||||||||||||||||||||||||||||||||||||||||||||||||||||||
Sbjct: 721  tgggaatttgttggtcatgtgacctcagattgttgggcaatcaaagatttccatgagcac 780


Query: 848  catatggttacttcaaatgcagtagagtccagtgcctttggcaatgaatcgtggttgcga 907
            |||||||||||||||||||||||||||| ||||  | |||||||||||||||||||||||
Sbjct: 781  catatggttacttcaaatgcagtagagt-cagttgccttggcaatgaatcgtggttgcga 839


Query: 908  cttaaactgtggaaatctttatgttaaattactacaagcagtacgggatggtttaatggg 967
            ||||||||||||||||||||||||||| ||||||||||||||||||||||||||| | ||
Sbjct: 840  cttaaactgtggaaatctttatgttaatttactacaagcagtacgggatggtttagt-gg 898


Query: 968  nnnnnnnnncaattgatactgctcctaaacgtctctttacaacaaaaatgaaa 1020
                     ||||||||||||||| || ||||||||||||||||| |||||||
Sbjct: 899  aagaagagacaattgatactgctcttatacgtctctttacaacaagaatgaaa 951


Cphy3009 reverse sequence
Query= HR0ADKBY80ZG03_CAZYH2C3P17_1.SCF   1301      0   1301  SCF

>Cphy3009
          Length = 2208

 Score = 1764 bits (890), Expect = 0.0
 Identities = 924/933 (99%), Gaps = 3/933 (0%)
 Strand = Plus / Minus


Query: 65   gtgtaatggatagtgatcTTAatggtgatggtgatgatgctcaccaagtaaaactgtttc 124
            ||||||||||||||||||||||||||||||||||||||||||||||||||||||||||||
Sbjct: 2208 gtgtaatggatagtgatcttaatggtgatggtgatgatgctcaccaagtaaaactgtttc 2149


Query: 125  aggtgcatggaactgtattttaattggattcttaccagttaactgaatacttctagtatc 184
            ||||||||||||||||||||||||||||||||||||||||||||||||||||||||||||
Sbjct: 2148 aggtgcatggaactgtattttaattggattcttaccagttaactgaatacttctagtatc 2089


Query: 185  tggttgtgaatcacttagatatagctcatactccccttgatttaagataaattcaccttc 244
```

```
            ||||||||||||||||||||||||||||||||||||||||||||||||||||||||||||
Sbjct: 2088 tggttgtgaatcacttagatatagctcatactccccttgatttaagataaattcaccttc 2029

Query: 245  ctcattatataaaccaaaggctctgtcatctaactcaatcatgatatctttttcttctcc 304
            ||||||||||||||||||||||||||||||||||||||||||||||||||||||||||||
Sbjct: 2028 ctcattatataaaccaaaggctctgtcatctaactcaatcatgatatctttttcttctcc 1969

Query: 305  tgggagtaagctcactttttaaggccttttagctgacagtttggagcatcttctcctac 364
            ||||||||||||||||||||||||||||||||||||||||||||||||||||||||||||
Sbjct: 1968 tgggagtaagctcactttttaaggccttttagctgacagtttggagcatcttctcctac 1909

Query: 365  atatttaacatatgcttgtgttgtcaccgaaccttcataatcaccggtattctttactct 424
            ||||||||||||||||||||||||||||||||||||||||||||||||||||||||||||
Sbjct: 1908 atatttaacatatgcttgtgttgtcaccgaaccttcataatcaccggtattctttactct 1849

Query: 425  taccatacactctacgttggaacctttcctaaagtatctgtatttacataaagtaatgt 484
            ||||||||||||||||||||||||||||||||||||||||||||||||||||||||||||
Sbjct: 1848 taccatacactctacgttggaacctttcctaaagtatctgtatttacataaagtaatgt 1789

Query: 485  gtgctcaaaggtagtataacttaagccatatccaaatggataaagagcctcattcttcat 544
            ||||||||||||||||||||||||||||||||||||||||||||||||||||||||||||
Sbjct: 1788 gtgctcaaaggtagtataacttaagccatatccaaatggataaagagcctcattcttcat 1729

Query: 545  ataacgatacgtacggttcttcatagcataatctgtaaactcaggtaattcctcagtcgt 604
            ||||||||||||||||||||||||||||||||||||||||||||||||||||||||||||
Sbjct: 1728 ataacgatacgtacggttcttcatagcataatctgtaaactcaggtaattcctcagtcgt 1669

Query: 605  acgatagaatgttacaggaagctttccttctgggtttccatctccaaaaatcaattctgc 664
            ||||||||||||||||||||||||||||||||||||||||||||||||||||||||||||
Sbjct: 1668 acgatagaatgttacaggaagctttccttctgggtttccatctccaaaaatcaattctgc 1609

Query: 665  aatcgctctaccaccttgtgctccaggataccagccttgtaatatagcaggaatatgctc 724
            ||||||||||||||||||||||||||||||||||||||||||||||||||||||||||||
Sbjct: 1608 aatcgctctaccaccttgtgctccaggataccagccttgtaatatagcaggaatatgctc 1549

Query: 725  atctgcccatggcactgccaaagcactaccggagagaaggattaaaataacaggttttcc 784
            ||||||||||||||||||||||||||||||||||||||||||||||||||||||||||||
Sbjct: 1548 atctgcccatggcactgccaaagcactaccggagagaaggattaaaataacaggttttcc 1489

Query: 785  acattcataaatagtttttaatacatcttctttgattccaggaagcgctaatgtcttctt 844
            ||||||||||||||||||||||||||||||||  ||||||||||||||||||||||||||
Sbjct: 1488 acattcataaatagtttttaatacatcttcttgaattccaggaagcgctaatgtcttctt 1429

Query: 845  atctccactagcaaattgattttccctggtcgccttcttctccttctaatcctggatcaa 904
            ||||||||||||||||||| ||||||||||||||||||||||||||||||||||||||||
Sbjct: 1428 atctccactagcaaattga-tttccctggtcgccttcttctccttctaatcctggatcaa 1370
```

```
Query: 905  gacccaggcatgctattacaacatccctatgttcacaaactgcacgtacctcaccaatac 964
            |||||||||||||||||| ||||| ||||||||||||||||||||||||| ||||||
Sbjct: 1369 gacccaggcatgctattacgacatcactatgttcacaaactgcacgtacctcagcaatac 1310


Query: 965  ggtcattttccttggacttaaattaaagagtttt 997
            ||||||||||||  ||||||||||| |||||||
Sbjct: 1309 ggtcattttcct--gacttaaattagagagttt 1279


#######

Cphy3158 forward sequence

Query= HR0ADKBY80ZA04_CAZYI1C1P16_1.SCF   1304      0   1304  SCF
         (1304 letters)

>Cphy3158
          Length = 2109

 Score = 1639 bits (827), Expect = 0.0
 Identities = 863/876 (98%), Gaps = 1/876 (0%)
 Strand = Plus / Plus


Query: 69   aaagaaggagataggatcATGgaagaagaagtagtattaacttttaatatnnnnnnngga 128
           ||||||||||||||||||||||||||||||||||||||||||||||||||       |||
Sbjct: 1    aaagaaggagataggatcatggaagaagaagtagtattaacttttaatataaaaaaagga 60


Query: 129 gatggaacaatggggtttgaaaaagcttggttaaattactataagagtgataatcttgcg 188
           ||||||||||||||||||||||||||||||||||||||||||||||||||||||||||||
Sbjct: 61  gatggaacaatggggtttgaaaaagcttggttaaattactataagagtgataatcttgcg 120


Query: 189 atgcgagattattttacagtcgttacatacaataaagacgactctattgtaaaaagtgcg 248
           ||||||||||||||||||||||||||||||||||||||||||||||||||||||||||||
Sbjct: 121 atgcgagattattttacagtcgttacatacaataaagacgactctattgtaaaaagtgcg 180


Query: 249 gtcttagaattaacaacagcagcaaaagagctattttcaatcgatatcgaaagcagcctt 308
           ||||||||||||||||||||||||||||||||||||||||||||||||||||||||||||
Sbjct: 181 gtcttagaattaacaacagcagcaaaagagctattttcaatcgatatcgaaagcagcctt 240


Query: 309 tgtgatatgaaatcagatgttgataaaacgagaaaagggattcattttcagattggtgat 368
           ||||||||||||||||||||||||||||||||||||||||||||||||||||||||||||
Sbjct: 241 tgtgatatgaaatcagatgttgataaaacgagaaaagggattcattttcagattggtgat 300


Query: 369 aataaagatctagaaaatgagggatatcgtatttcgttagcagacgagatgctattgata 428
           ||||||||||||||||||||||||||||||||||||||||||||||||||||||||||||
Sbjct: 301 aataaagatctagaaaatgagggatatcgtatttcgttagcagacgagatgctattgata 360


Query: 429 gaggcttacacctcaaatggtattctctatggaacttttgatttgattcgtagcaccatt 488
           ||||||||||||||||||||||||||||||||||||||||||||||||||||||||||||
```

```
Sbjct: 361 gaggcttacacctcaaatggtattctctatggaacttttgatttgattcgtagcaccatt 420


Query: 489 cttggagcagatgtgcattccttcaatagaactatctttccaaagaatcctcttcgaatg 548
           ||||||||||||||||||||||||||||||||||||||||||||||||||||||||||||
Sbjct: 421 cttggagcagatgtgcattccttcaatagaactatctttccaaagaatcctcttcgaatg 480


Query: 549 ttaaatcattgggataatatggacggtagtattgaaagaggttattctggtaattctttc 608
           ||||||||||||||||||||||||||||||||||||||||||||||||||||||||||||
Sbjct: 481 ttaaatcattgggataatatggacggtagtattgaaagaggttattctggtaattctttc 540


Query: 609 ttttttgaacatggtgaagttattgtaaatgaacgtactaaggcctatgcaagacttgtt 668
           ||||||||||||||||||||||||||||||||||||||||||||||||||||||||||||
Sbjct: 541 ttttttgaacatggtgaagttattgtaaatgaacgtactaaggcctatgcaagacttgtt 600


Query: 669 tcctccgttggtataaacgcagttgtgataaacaatgtaaatgtccgtggtacagcaact 728
           ||||||||||||||||||||||||||||||||||||||||||||||||||||||||||||
Sbjct: 601 tcctccgttggtataaacgcagttgtgataaacaatgtaaatgtccgtggtacagcaact 660


Query: 729 aatttaataacaaatcgctacatagataaattaaaacaaatcggtgaaatttttgcttct 788
           ||||||||||||||||||||||||||||||||||||||||||||||||||||||||||||
Sbjct: 661 aatttaataacaaatcgctacatagataaattaaaacaaatcggtgaaatttttgcttct 720


Query: 789 tatggaattaagctttatctttcttttaattttgcggctccaatggaacttggtggttta 848
           ||||||||||||||||||||||||| ||||||||||||||||||||||||||||||||||
Sbjct: 721 tatggaattaagctttatctttctttaaattttgcggctccaatggaacttggtggttta 780


Query: 849 acttctgctgacccactcgacgaggaaatacgtctttgggggaagaaagaagtgcaatta 908
           |||||||||||||||||||||||||| ||||||||||| |||||||||||||||||||||
Sbjct: 781 acttctgctgacccactcgacgaggaagtacgtctttggtggaagaaagaagtgcaatta 840


Query: 909 ttatttaaaagaaatacctccctttggcggattttt 944
           |||||| |||||||||||  ||||||||||||||||
Sbjct: 841 ttattt-aaagaaataccatcctttggcggattttt 875


Cphy3158 reverse sequence


Query= HR0ADKBY80ZA04_CAZYI1C1P17_1.SCF   1308      0   1308  SCF
         (1308 letters)

>Cphy3158
          Length = 2109

 Score = 1509 bits (761), Expect = 0.0
 Identities = 819/833 (98%), Gaps = 4/833 (0%)
 Strand = Plus / Minus


Query: 66   gtgtaatggatagtgatcTTAatggtgatggtgatgatgatataactccctccccttttt 125
```

```
              ||||||||||||||||||||||||||||||||||||||||||||||||||||||||||||
Sbjct: 2109 gtgtaatggatagtgatcttaatggtgatggtgatgatgatataactccctccccttttt 2050


Query: 126  atcccctatcgctgtcttcctataaaaataagaacaaatcacgtctctccattccttcga 185
             ||||||||||||||||||||||||||||||||||||||||||||||||||||||||||||
Sbjct: 2049 atcccctatcgctgtcttcctataaaaataagaacaaatcacgtctctccattccttcga 1990


Query: 186  atgttccagctgttcctgtaatctatgcaataccctgtgatatggtacttcttccaactt 245
             ||||||||||||||||||||||||||||||||||||||||||||||||||||||||||||
Sbjct: 1989 atgttccagctgttcctgtaatctatgcaataccctgtgatatggtacttcttccaactt 1930


Query: 246  tccttttagttcttcccataacttaaccatctcttccacatcctctactccctcaaaatg 305
             ||||||||||||||||||||||||||||||||||||||||||||||||||||||||||||
Sbjct: 1929 tccttttagttcttcccataacttaaccatctcttccacatcctctactccctcaaaatg 1870


Query: 306  agaatcatagatatgttggattaaggtcttacctgtctttaactgataggtatatggaat 365
             ||||||||||||||||||||||||||||||||||||||||||||||||||||||||||||
Sbjct: 1869 agaatcatagatatgttggattaaggtcttacctgtctttaactgataggtatatggaat 1810


Query: 366  gcgatggaagaataaaagcaactcttctgggcatgtttcaggtgttccatagagctcttg 425
             ||||||||||||||||||||||||||||||||||||||||||||||||||||||||||||
Sbjct: 1809 gcgatggaagaataaaagcaactcttctgggcatgtttcaggtgttccatagagctcttg 1750


Query: 426  gtttggctgatgatactgtgttgtgtatcctgttccattcttggttcggtctacaccaat 485
             ||||||||||||||||||||||||||||||||||||||||||||||||||||||||||||
Sbjct: 1749 gtttggctgatgatactgtgttgtgtatcctgttccattcttggttcggtctacaccaat 1690


Query: 486  tgctgtatggtctgctttatgataggttccccaacggtcgtattcatacccgtcaacatt 545
             ||||||||||||||||||||||||||||||||||||||||||||||||||||||||||||
Sbjct: 1689 tgctgtatggtctgctttatgataggttccccaacggtcgtattcatacccgtcaacatt 1630


Query: 546  aggtccgtaatgatgagaaggatttaccatccatccaataccaagcggagaagtatattt 605
             ||||||||||||||||||||||||||||||||||||||||||||||||||||||||||||
Sbjct: 1629 aggtccgtaatgatgagaaggatttaccatccatccaataccaagcggagaagtatattt 1570


Query: 606  ctcatatgctggccaggacatcattaggattttggatatataatctataacatgctcctt 665
             ||||||||||||||||||||||||||||||||||||||||||||||||||||||||||||
Sbjct: 1569 ctcatatgctggccaggacatcattaggattttggatatataatctataacatgctcctt 1510


Query: 666  catgccaaatgttaaggtgatccattcttttgctatctcctctgaatgtaaatcaggatc 725
             |||||||||||||||||||||||||||||||||||||||||||||   ||||||||||||
Sbjct: 1509 catgccaaatgttaaggtgatccattcttttgctatctcctctgatgttaaatcaggatc 1450


Query: 726  aaaactcagttctaccaatatccatagagattcgctgctgccaagtcatgaccagtccag 785
             ||||||||| |||||| |||||||||||||||||||||||||||||||||||||||||||
Sbjct: 1449 aaaactcag-tctacc-atatccatagagattcgctgctgccaagtcatgaccagtccag 1392
```

```
Query: 786  ttatcatcattcccctgtatttgcaactgccgccattccagcgttttattgccaaaagt 845
            ||||||||||| |||||||||||||||||| ||||||||||||||||||||||| ||||
Sbjct: 1391 ttatcatcatt-ccctgtatttgcaactgctgccattccagcgttttattgccataagt 1333


Query: 846  gttgccactaacgaaatcaaatacccgatcattttcttctttacaaaaagtt 898
            |||||||||||||| ||| ||||||  |||| |||||||||||||| |||||
Sbjct: 1332 gttgccactaacgatatcgaataccgtatca-tttcttctttacaataagtt 1281

#######

Cphy3160 forward sequence

Query= HR0ADKBY80ZD04_CAZYJ1C4P16_1.SCF   1352      0   1352  SCF

>Cphy3160
          Length = 3612

 Score = 1542 bits (778), Expect = 0.0
 Identities = 815/822 (99%), Gaps = 4/822 (0%)
 Strand = Plus / Plus


Query: 68  aaagaaggagataggatcATGagcagtcaagtacataacaatcgaagattaatgaatgat 127
           ||||||||||||||||||||||||||||||||||||||||||||||||||||||||||||
Sbjct: 1   aaagaaggagataggatcatgagcagtcaagtacataacaatcgaagattaatgaatgat 60


Query: 128 ggctggagtttttccaaacatcagctaggcgctaccttacaacaagtgttagatgcaaaa 187
           ||||||||||||||||||||||||||||||||||||||||||||||||||||||||||||
Sbjct: 61  ggctggagtttttccaaacatcagctaggcgctaccttacaacaagtgttagatgcaaaa 120


Query: 188 acagaatggcatcccgttgatttgccccacgactggttaatttataatacccacgatttg 247
           ||||||||||||||||||||||||||||||||||||||||||||||||||||||||||||
Sbjct: 121 acagaatggcatcccgttgatttgccccacgactggttaatttataatacccacgatttg 180


Query: 248 tatgaaacaggagaaggttggtacagacgtacgttatccatggaacacctgaaaaataag 307
           ||||||||||||||||||||||||||||||||||||||||||||||||||||||||||||
Sbjct: 181 tatgaaacaggagaaggttggtacagacgtacgttatccatggaacacctgaaaaataag 240


Query: 308 gagcgatatatattacgtttcgagggcgtgtatatgaactcgaccctctatgtcaatgga 367
           ||||||||||||||||||||||||||||||||||||||||||||||||||||||||||||
Sbjct: 241 gagcgatatatattacgtttcgagggcgtgtatatgaactcgaccctctatgtcaatgga 300


Query: 368 aggtttgctggagagtggaaatacggttactcaacatttgaattcgatataaccgagttt 427
           ||||||||||||||||||||||||||||||||||||||||||||||||||||||||||||
Sbjct: 301 aggtttgctggagagtggaaatacggttactcaacatttgaattcgatataaccgagttt 360


Query: 428 ttaacagaaggtggcaatgaaattgtgatgcaagttatctacgagtcaccgaactcgaga 487
           ||||||||||||||||||||||||||||||||||||||||||||||||||||||||||||
Sbjct: 361 ttaacagaaggtggcaatgaaattgtgatgcaagttatctacgagtcaccgaactcgaga 420
```

```
Query: 488 tggtattccggtgcaggtatttatcgtaatgtctggttccatacctatcctgaagtacat 547
           ||||||||||||||||||||||||||||||||||||||||||||||||||||||||||||
Sbjct: 421 tggtattccggtgcaggtatttatcgtaatgtctggttccatacctatcctgaagtacat 480


Query: 548 attaacccagatggaatttatatttcaacagaaaaagtggatgaggatttttacgttttc 607
           ||||||||||||||||||||||||||||||||||||||||||||||||||||||||||||
Sbjct: 481 attaacccagatggaatttatatttcaacagaaaaagtggatgaggatttttacgttttc 540


Query: 608 ctatcaaccgaagtttcctctttaccgatcagtatggaacctgagcttacaataccagag 667
           ||||||||||||||||||||||||||||||||||||||||||||||||||||||||||||
Sbjct: 541 ctatcaaccgaagtttcctctttaccgatcagtatggaacctgagcttacaataccagag 600


Query: 668 gattttcatggattagacaaggattccacatctacactcgaacttcgccatactgtatgg 727
           ||||||||||||||||||||||||||||||||||||||||||||||||||||||||||||
Sbjct: 601 gattttcatggattagacaaggattccacatctacactcgaacttcgccatactgtatgg 660


Query: 728 tcgccaagtcatgaattagtttgcacccatactgtcgatgttacaagtgaggtaataaag 787
           |||||||||||||||||||||||||||||||||||||||||||||||||||||| |||||
Sbjct: 661 tcgccaagtcatgaattagtttgcacccatactgtcgatgttacaagtgaggtagtaaag 720


Query: 788 aatgccttttatgtacaaaatagtaaactacatattattttcacctcttcctttgggagc 847
           |||||||||||||||||| |||||||||||||||||| ||||||||||| ||||||||||
Sbjct: 721 aatgccttttatgtacaagatagtaaactacatatta-tttcacctctt-ctttgggagc 778


Query: 848 tttcgtcacccaatttatatccaagtgcaaacttgatattat 889
           |||||||||| ||||||||| ||||||||||| |||||||||
Sbjct: 779 tttcgtcaccaaatttatat-caagtgcaaac-tgatattat 818
```

Cphy3160 reverse sequence

```
Query= HR0ADKBY80ZD04_CAZYJ1C4P17_1.SCF   1324      0   1324  SCF

>Cphy3160
          Length = 3612

 Score = 1635 bits (825), Expect = 0.0
 Identities = 875/888 (98%), Gaps = 4/888 (0%)
 Strand = Plus / Minus


Query: 67   gtgtaatggatagtgatcTTAatggtgatggtgatgatgttcaaacttaaaccactcaaa 126
            ||||||||||||||||||||||||||||||||||||||||||||||||||||||||||||
Sbjct: 3612 gtgtaatggatagtgatcttaatggtgatggtgatgatgttcaaacttaaaccactcaaa 3553


Query: 127  gtcaaaattactgcccggtaggaatataaaggtaacagtatatttacctgtcttcttaga 186
            ||||||||||||||||||||||||||||||||||||||||||||||||||||||||||||
Sbjct: 3552 gtcaaaattactgcccggtaggaatataaaggtaacagtatatttacctgtcttcttaga 3493
```

```
Query: 187  gaattcaaactcttttacttcgtactcattcgtaaaagtaaattctgcaagttcttcaat 246
            ||||||||||||||||||||||||||||||||||||||||||||||||||||||||||||
Sbjct: 3492 gaattcaaactcttttacttcgtactcattcgtaaaagtaaattctgcaagttcttcaat 3433


Query: 247  gctattatagagcatactaccttcttctgaaataaactttacatgaatggtgttcttctc 306
            ||||||||||||||||||||||||||||||||||||||||||||||||||||||||||||
Sbjct: 3432 gctattatagagcatactaccttcttctgaaataaactttacatgaatggtgttcttctc 3373


Query: 307  aatcggagaatgaccacagatagagagtttcgtaatgccctgttctccgaaatccatctc 366
            ||||||||||||||||||||||||||||||||||||||||||||||||||||||||||||
Sbjct: 3372 aatcggagaatgaccacagatagagagtttcgtaatgccctgttctccgaaatccatctc 3313


Query: 367  tgaaaattccaaggatacgttattcccgattccttcaatgcgaggagctttctttgtaaa 426
            ||||||||||||||||||||||||||||||||||||||||||||||||||||||||||||
Sbjct: 3312 tgaaaattccaaggatacgttattcccgattccttcaatgcgaggagctttctttgtaaa 3253


Query: 427  ggtatctccatatatgaggtcacaatcagtagcgaataattttgcaaatcctttttgtgg 486
            ||||||||||||||||||||||||||||||||||||||||||||||||||||||||||||
Sbjct: 3252 ggtatctccatatatgaggtcacaatcagtagcgaataattttgcaaatcctttttgtgg 3193


Query: 487  attggtaaactgaaatccttgaacgcttaatcgtttaaatacaacgaaggaaattgttac 546
            ||||||||||||||||||||||||||||||||||||||||||||||||||||||||||||
Sbjct: 3192 attggtaaactgaaatccttgaacgcttaatcgtttaaatacaacgaaggaaattgttac 3133


Query: 547  gatacctttactctacgctttaatcgataggtttccggttgatggtgattccatattgg 606
            ||||||||||||||||||||||||||||||||||||||||||||||||||||||||||||
Sbjct: 3132 gatacctttactctacgctttaatcgataggtttccggttgatggtgattccatattgg 3073


Query: 607  ctttttactaaatgaacagtctgctagtaattcacttccttcttcatgtgggataccttc 666
            ||||||||||||||||||||||||||||||||||||||||||||||||||||||||||||
Sbjct: 3072 ctttttactaaatgaacagtctgctagtaattcacttccttcttcatgtgggataccttc 3013


Query: 667  ccaaatctcaaaagttggaagctcagtatcaagacagtagattggaacagtaatctcgtc 726
            ||||||||||||||||||||||||||||||||||||||||||||||||||||||||||||
Sbjct: 3012 ccaaatctcaaaagttggaagctcagtatcaagacagtagattggaacagtaatctcgtc 2953


Query: 727  agatccataggagccaaagtctacattttaaaacaaatgtaagtatccattggtagcag 786
            ||||||||||||||||||||||||||||||||||||||||||||||||||||||||||||
Sbjct: 2952 agatccataggagccaaagtctacattttaaaacaaatgtaagtatccattggtagcag 2893


Query: 787  agaggagaaaccgcgctcaattccgttggtaaggtttgtattggaccaagagtataaacc 846
            |||||||| |||||||||||||||||||||||||||||||||||||||||||||||||||
Sbjct: 2892 agaggagataccgcgctcaattccgttggtaaggtttgtattggaccaagagtataaacc 2833


Query: 847  accatatataaattcctacggattcatggaagcttctccaaggtcatggaaagttagcct 906
            ||||||||||||||| ||||||||||||| |||||||||||||||||||||  | |||  
Sbjct: 2832 accatatataaattcatacggattcatggtagcttctccaaggtcatggaagttaagc-- 2775
```

```
Query: 907  tcttttcaaaaatcatacgttaccttatccgcaccatttcctacagg 954
            ||| ||||| |||||||||| ||||||||| |||||| ||||||||||
Sbjct: 2774 tctatttcagaaatcatacg-taccttatctgcacca-ttcctacagg 2729

#######

Cphy3367 forward sequence


Query= HR0ADKBY80ZH04_CAZYK2C4P16_1.SCF   1327      0   1327  SCF

>Cphy3367*
          Length = 2940

 Score =  821 bits (414), Expect = 0.0
 Identities = 426/430 (99%)
 Strand = Plus / Plus


Query: 67  aaagaaggagataggatcATGgaaaccaattataattacggagaagctcttcaaaaatca 126
           ||||||||||||||||||||||||||||||||||||||||||||||||||||||||||||
Sbjct: 1   aaagaaggagataggatcatggaaaccaattataattacggagaagctcttcaaaaatca 60


Query: 127 atcatgttttatgagtttcaacgttctggtaaactgccaagtaccattcggaataattgg 186
           ||||||||||||||||||||||||||||||||||||||||||||||||||||||||||||
Sbjct: 61  atcatgttttatgagtttcaacgttctggtaaactgccaagtaccattcggaataattgg 120


Query: 187 agaggtgactctggtttaaccgatggagcagatgttggtttggatctaactggtggctgg 246
           ||||||||||||||||||||||||||||||||||||||||||||||||||||||||||||
Sbjct: 121 agaggtgactctggtttaaccgatggagcagatgttggtttggatctaactggtggctgg 180


Query: 247 tatgatgctggtgatcatgtaaaatttaatcttcctttggcttatactgtaacaatgtta 306
           ||||||||||||||||||||||||||||||||||||||||||||||||||||||||||||
Sbjct: 181 tatgatgctggtgatcatgtaaaatttaatcttcctttggcttatactgtaacaatgtta 240


Query: 307 gcatgggcagtatatgaagaagaggctactctttcaaaggcaggccaattaagttattta 366
           ||||||||||||||||||||||||||||||||||||||||||||||||||||||||||||
Sbjct: 241 gcatgggcagtatatgaagaagaggctactctttcaaaggcaggccaattaagttattta 300


Query: 367 ttagatgaaattaagtggtctagtgattacctaattaaatgtcatccacaagcaaatgta 426
           ||||||||||||||||||||||||||||||||||||||||||||||||||||||||||||
Sbjct: 301 ttagatgaaattaagtggtctagtgattacctaattaaatgtcatccacaagcaaatgta 360


Query: 427 tttcattatcaggttggtaatggaaatacagatcactcttggtggggacctgccgaatct 486
           ||| ||||||||||||||||||||||||||||||||||||||||||||||||| |||  |
Sbjct: 361 ttttattatcaggttggtaatggaaatacagatcactcttggtggggacctgctgaagtt 420


Query: 487 atgcagatgg 496
           ||||||||||
```

```
Sbjct: 421 atgcagatgg 430


Cphy3367 reverse sequence
Query= HR0ADKBY80ZH04_CAZYK2C4P17_1.SCF   1369      0   1369  SCF

>Cphy3367*
          Length = 2940

 Score =  952 bits (480), Expect = 0.0
 Identities = 548/564 (97%), Gaps = 5/564 (0%)
 Strand = Plus / Minus


Query: 67   gtgtaatggatagtgatcTTAatggtgatggtgatgatgtggttcgactccccaaaccaa 126
            ||||||||||||||||||||||||||||||||||||||||||||||||||||||||||||
Sbjct: 2940 gtgtaatggatagtgatcttaatggtgatggtgatgatgtggttcgactccccaaaccaa 2881


Query: 127  aacatcagagatatatactgttgtcttattccaatcagcataacttgagttactatcacc 186
            ||||||||||||||||||||||||||||||||||||||||||||||||||||||||||||
Sbjct: 2880 aacatcagagatatatactgttgtcttattccaatcagcataacttgagttactatcacc 2821


Query: 187  aaacgaataatcatctgtttgtgtatagtttgtccagtctaccttagaaaatcttccttg 246
            ||||||||||||||||||||||||||||||||||||||||||||||||||||||||||||
Sbjct: 2820 aaacgaataatcatctgtttgtgtatagtttgtccagtctaccttagaaaatcttccttg 2761


Query: 247  aacttcaatactttgccctgcgtttaaagttccggcagcggacttaaatgaaaattctag 306
            ||||||||||||||||||||||||||||||||||||||||||||||||||||||||||||
Sbjct: 2760 aacttcaatactttgccctgcgtttaaagttccggcagcggacttaaatgaaaattctag 2701


Query: 307  atagtaatctgcatttgtttttggtgtactcatctttacgaaagtaccatttacattgga 366
            ||||||||||||||||||||||||||||||||||||||||||||||||||||||||||||
Sbjct: 2700 atagtaatctgcatttgtttttggtgtactcatctttacgaaagtaccatttacattgga 2641


Query: 367  actaccaatcgtcgaataatcacaccagaatgcctgatccttttcgccattgattgtata 426
            ||||||||||||||||||||||||||||||||||||||||||||||||||||||||||||
Sbjct: 2640 actaccaatcgtcgaataatcacaccagaatgcctgatccttttcgccattgattgtata 2581


Query: 427  ataatagcgaatcttaacatcggataagttgattgcagtagttccggtgttaattaattt 486
            ||||||||||||||||||||||||||||||||||||||||||||||||||||||||||||
Sbjct: 2580 ataatagcgaatcttaacatcggataagttgattgcagtagttccggtgttaattaattt 2521


Query: 487  aattcttggtgcaatgccattcgttgttgcagaagaattttccttaaacattttgaaatt 546
            |||||||||||||||||||||||||||||||||||||||| | ||||||| |||||| ||
Sbjct: 2520 aattcttggtgcaatgccattcgttgttgcagaagaatttccattaaaca-tttgaagtt 2462


Query: 547  taatattttcctgttggagtatatactgaagaatccttatcggtaactggctaa-actgg 605
            ||||| |||||||||||||||   || ||||||||||||  ||||||| ||||| |||||
Sbjct: 2461 taata-tttcctgttggagtaacaacagaagaatccttaatggtaact-gctaagactgg 2404
```

```
Query: 606  actctgtccctttattcttttcaa 629
            | | ||||||||||||| ||||||
Sbjct: 2403 a-tttgtccctttattcatttcaa 2381
```

#######

Cphy0591-4 forward sequence

>cphy0591

```
Query: 69   aaagaaggagataggatcATGaataaattagggagcattccattaaaagacatttgtatc 128
            ||||||||||||||||||||||||||||||||||||||||||||||||||||||||||||
Sbjct: 1    aaagaaggagataggatcatgaataaattagggagcattccattaaaagacatttgtatc 60


Query: 129  caagatgaattttggaatcggtatgttgagcttgtgagaaagataattattccatatcaa 188
            ||||||||||||||||||||||||||||||||||||||||||||||||||||||||||||
Sbjct: 61   caagatgaattttggaatcggtatgttgagcttgtgagaaagataattattccatatcaa 120


Query: 189  tgggatatcttaaatgataatttagaaggcgttgaaacgagtcatagcattatgaatttc 248
            ||||||||||||||||||||||||||||||||||||||||||||||||||||||||||||
Sbjct: 121  tgggatatcttaaatgataatttagaaggcgttgaaacgagtcatagcattatgaatttc 180


Query: 249  aaaattgccgctggaagagaacaaggggagttctatggtgcagtctttcaggataccgat 308
            ||||||||||||||||||||||||||||||||||||||||||||||||||||||||||||
Sbjct: 181  aaaattgccgctggaagagaacaaggggagttctatggtgcagtctttcaggataccgat 240


Query: 309  ttagcaaagtggattgaggcagttgcctattcacttgcaacagacccagattcaaaattg 368
            ||||||||||||||||||||||||||||||||||||||||||||||||||||||||||||
Sbjct: 241  ttagcaaagtggattgaggcagttgcctattcacttgcaacagacccagattcaaaattg 300


Query: 369  gaggaattggtagatgatgtagtggaacttattgaggaagcgcaatgtttagatggctac 428
            ||||||||||||||||||||||||||||||||||||||||||||||||||||||||||||
Sbjct: 301  gaggaattggtagatgatgtagtggaacttattgaggaagcgcaatgtttagatggctac 360


Query: 429  attaatacttactttactataaaggaaccggatcttcgctttacaaatttaaaagaaggt 488
            ||||||||||||||||||||||||||||||||||||||||||||||||||||||||||||
Sbjct: 361  attaatacttactttactataaaggaaccggatcttcgctttacaaatttaaaagaaggt 420


Query: 489  catgaattgtattgtgctgggcatttaatggaggcagctgttgcttattataaggcaact 548
            ||||||||||||||||||||||||||||||||||||||||||||||||||||||||||||
Sbjct: 421  catgaattgtattgtgctgggcatttaatggaggcagctgttgcttattataaggcaact 480


Query: 549  ggaaaggaaagatttatacatattgtagagagatttgcggatttaatttgtgatgaattt 608
            ||||||||||||||||||||||||||||||||||||||||||||||||||||||||||||
Sbjct: 481  ggaaaggaaagatttatacatattgtagagagatttgcggatttaatttgtgatgaattt 540
```

```
Query: 609  tcaccagnnnnnnntggtgcagggtatccaggacatcaggaggtagagattggattaatc 668
            |||||||       ||||||||||||||||||||||||||||||||||||||||||||||
Sbjct: 541  tcaccagaaaaaaatggtgcagggtatccaggacatcaggaggtagagattggattaatc 600


Query: 669  aaattatatgaagtcacagggaagaagaagtacattgagcaagcaagattatttttagaa 728
            ||||||||||||||||||||||||||||||||||||||||||||||||||||||||||||
Sbjct: 601  aaattatatgaagtcacagggaagaagaagtacattgagcaagcaagattatttttagaa 660


Query: 729  agaagaggaacttccccaaactattttcttgaggaagnnnnnnncccaaagtttaagcct 788
            |||||||||||||||||||||||||||||||||||||       ||||||||||||||||
Sbjct: 661  agaagaggaacttccccaaactattttcttgaggaagaaaaaaacccaaagtttaagcct 720


Query: 789  atatttcctgagtttatgcattatgataccgcctattctcaatcccacaaaccaatccgt 848
            ||||||||||||||||||||||||||||||||||||||||||||||||||||||||||||
Sbjct: 721  atatttcctgagtttatgcattatgataccgcctattctcaatcccacaaaccaatccgt 780


Query: 849  gaacaaacgacagccgaaagtcctgcggtacgggcggtatatatgtattctgcaatggct 908
            |||||||||||||| ||| ||| |||||||||||||||||||||||||||||||||||||
Sbjct: 781  gaacaaacgacagcagaaggtcatgcggtacgggcggtatatatgtattctgcaatggct 840


Query: 909  gactttgcaccaccctataatgaccaagaacctgttaaggtgtttgtgaaaccctatgga 968
            ||| ||||| || ||||||||||| ||||| ||||||| |||||||||||||||||||||
Sbjct: 841  gacattgcagcagcctataatgacaaagaa-ctgttaa-gtgtttgtgaaaccctatgga 898


Query: 969  agaatattggaaggaaagcgtatgtatctaaacgggtgggattgggtagttccggaatat 1028
            |||||||| | |||||||||||||||||| ||||||||| ||| ||||||||||||||||
Sbjct: 899  agaatatt-gtaggaaagcgtatgtatct-aacgggtggcatt-ggtagttccggaatat 955


Query: 1029 tagaa 1033
            |||||
Sbjct: 956  tagaa 960

#######

Cphy0591-4 reverse sequence

Query= HR0ADKBY82ZA04_0591C4P17_1.SCF   1375      0   1375  SCF

>cphy0591

Query: 66   gtgtaatggatagtgatcTTAatggtgatggtgatgatgtacaaattcttttatccaaac 125
            ||||||||||||||||||||||||||||||||||||||||||||||||||||||||||||
Sbjct: 1995 gtgtaatggatagtgatcttaatggtgatggtgatgatgtacaaattcttttatccaaac 1936


Query: 126  aatcatctctcctgtttttcgatttgcccaatatggatatggtataaacttaannnnnnn 185
            |||||||||||||||||||||||||||||||||||||||||||||||||||||
Sbjct: 1935 aatcatctctcctgtttttcgatttgcccaatatggatatggtataaacttaatttttttt 1876
```

```
Query: 186  ctctgcaaatttcacgggatgttccgaatataaagtttcattctcccattcctcttgtat 245
            ||||||||||||||||||||||||||||||||||||||||||||||||||||||||||||
Sbjct: 1875 ctctgcaaatttcacgggatgttccgaatataaagtttcattctcccattcctcttgtat 1816


Query: 246  cattttctttccatcacattgaatcaccatcgttccctttagtagcgtatcatcataaaa 305
            ||||||||||||||||||||||||||||||||||||||||||||||||||||||||||||
Sbjct: 1815 cattttctttccatcacattgaatcaccatcgttccctttagtagcgtatcatcataaaa 1756


Query: 306  ctcttctaattcagtatctgaatctatataatacgctggaaggtttgattcgttatccac 365
            ||||||||||||||||||||||||||||||||||||||||||||||||||||||||||||
Sbjct: 1755 ctcttctaattcagtatctgaatctatataatacgctggaaggtttgattcgttatccac 1696


Query: 366  ctcttccatacaataaactagcggtcccttcatgattgccactttcccaatatcttcttt 425
            ||||||||||||||||||||||||||||||||||||||||||||||||||||||||||||
Sbjct: 1695 ctcttccatacaataaactagcggtcccttcatgattgccactttcccaatatcttcttt 1636


Query: 426  cactagcggattcgctctaatcatccgtggcttcaacgtaaactccatatcaatcgtttt 485
            ||||||||||||||||||||||||||||||||||||||||||||||||||||||||||||
Sbjct: 1635 cactagcggattcgctctaatcatccgtggcttcaacgtaaactccatatcaatcgtttt 1576


Query: 486  ctttgaagaagttatctctatttcaagataaccgttcgttatttctttttgggtcaattc 545
            ||||||||||||||||||||||||||||||||||||||||||||||||||||||||||||
Sbjct: 1575 ctttgaagaagttatctctatttcaagataaccgttcgttatttctttttgggtcaattc 1516


Query: 546  taaaccatttacaaatacacaatactccttagcatattcaggaattcgtatataaagttt 605
            ||||||||||||||||||||||||||||||||||||||||||||||||||||||||||||
Sbjct: 1515 taaaccatttacaaatacacaatactccttagcatattcaggaattcgtatataaagttt 1456


Query: 606  tccacaaaacccttcctctccatccacctccatatgaactttgccatcatacggaaacct 665
            ||||||||||||||||||||||||||||||||||||||||||||||||||||||||||||
Sbjct: 1455 tccacaaaacccttcctctccatccacctccatatgaactttgccatcatacggaaacct 1396


Query: 666  tgttgccaaccgcagtgtagcttccctgttttgcaattttacagtagtctgatttgaaat 725
            ||||||||||||||||||||||||||||||||||||||||||||||||||||||||||||
Sbjct: 1395 tgttgccaaccgcagtgtagcttccctgttttgcaattttacagtagtctgatttgaaat 1336


Query: 726  aaataagttaacccatatcgagttctcatcgtaaaagtagatatactcaccaagtgaagc 785
            ||||||||||||||||||||||||||||||||||||||||||||||||||||||||||||
Sbjct: 1335 aaataagttaacccatatcgagttctcatcgtaaaagtagatatactcaccaagtgaagc 1276


Query: 786  caatgttctcgctacatttggtgggcaacaagcaacaccaaaccatggctgacgtatcgg 845
            ||||||||||||||||||||||||||||||||||||||||||||||||||||||||||||
Sbjct: 1275 caatgttctcgctacatttggtgggcaacaagcaacaccaaaccatggctgacgtatcgg 1216


Query: 846  ttttacatgttctttaaaagttctcttaatgcaattgccaggccaaacttctaccgggtt 905
            |||||||||||||||| |||||||||||||||||||||||||||||||||||| ||||||
Sbjct: 1215 ttttacatgttctttagaagttctcttaatgcaattgccaggccaaacttctaacgggtt 1156
```

```
Query: 906  tacatnnnnnnnnctttttttccatcc-ttgctattcccccaagcacgggattaaaaagtg 964
            |||||        | ||||||||||| ||||||||||| ||||||||| |||| ||||||
Sbjct: 1155 tacataaaaaaagc-tttttccatccattgctattcccgcaagcacggtattataaagtg 1097


Query: 965  ttctttccactacatcc 981
             |||||| |||||||||
Sbjct: 1096 ctctttcaactacatcc 1080
```

#######

Cphy1163-1 forward sequence

Query= HR0ADKBY82ZA06_1163C1P16_1.SCF   1392   0   1392 SCF

>cphy1163

```
Query: 68  aaagaaggagataggatcATGgatacgaataatgatgattggctacattgtgtaggcaat 127
           ||||||||||||||||||||||||||||||||||||||||||||||||||||||||||||
Sbjct: 1   aaagaaggagataggatcatggatacgaataatgatgattggctacattgtgtaggcaat 60


Query: 128 aaaatttatgacatgaatggcaatgaggtttggctgaccggtgcgaattggtttggtttt 187
           ||||||||||||||||||||||||||||||||||||||||||||||||||||||||||||
Sbjct: 61  aaaatttatgacatgaatggcaatgaggtttggctgaccggtgcgaattggtttggtttt 120


Query: 188 aactgtactgaaaatgtatttcatggtgcatggtacgatattaaggggatgttaactaat 247
           ||||||||||||||||||||||||||||||||||||||||||||||||||||||||||||
Sbjct: 121 aactgtactgaaaatgtatttcatggtgcatggtacgatattaaggggatgttaactaat 180


Query: 248 attgcaaacagaggaataggatttttaagagttccaatttcaacggaacttttgtatagt 307
           ||||||||||||||||||||||||||||||||||||||||||||||||||||||||||||
Sbjct: 181 attgcaaacagaggaataggatttttaagagttccaatttcaacggaacttttgtatagt 240


Query: 308 tggatgataggcaaacctaataaagtttcaagtgtgaccgctgtcaataatccaccttat 367
           ||||||||||||||||||||||||||||||||||||||||||||||||||||||||||||
Sbjct: 241 tggatgataggcaaacctaataaagtttcaagtgtgaccgctgtcaataatccaccttat 300


Query: 368 tatgtatgcaaccctgatttttatgatcctacaacaaatagtgttaaaaatagtatggaa 427
           ||||||||||||||||||||||||||||||||||||||||||||||||||||||||||||
Sbjct: 301 tatgtatgcaaccctgatttttatgatcctacaacaaatagtgttaaaaatagtatggaa 360


Query: 428 atatttgatatcattatgggatactgcaaacaattggggatcaaagtaatggtagatgtt 487
           ||||||||||||||||||||||||||||||||||||||||||||||||||||||||||||
Sbjct: 361 atatttgatatcattatgggatactgcaaacaattggggatcaaagtaatggtagatgtt 420


Query: 488 catagtccggatgcaaataattcaggtcataactatccgttatggtatgggttaactacg 547
           ||||||||||||||||||||||||||||||||||||||||||||||||||||||||||||
Sbjct: 421 catagtccggatgcaaataattcaggtcataactatccgttatggtatgggttaactacg 480
```

```
Query: 548 actactgcaggtgaaataacgacagataagtggatcaatactcaagcatggctggctgat 607
           ||||||||||||||||||||||||||||||||||||||||||||||||||||||||||||
Sbjct: 481 actactgcaggtgaaataacgacagataagtggatcaatactcaagcatggctggctgat 540


Query: 608 aaatacaaaaatgacgatactattctggcatttgatataaaaaatgaacctcatggacag 667
           ||||||||||||||||||||||||||||||||||||||||||||||||||||||||||||
Sbjct: 541 aaatacaaaaatgacgatactattctggcatttgatataaaaaatgaacctcatggacag 600


Query: 668 aggggatatagtactacaacacctactaatataccaaaatgggataattccccaaatgag 727
           |||||||||||||||||||||||||||||||| ||||||||||||||||| || |||||
Sbjct: 601 aggggatatagtactacaacacctactaatatagcaaaatgggataattccacagatgag 660


Query: 728 aataactggaattatgcggcggaaaagatgtgcgaaagctatacttgctaaaaaccccta 787
           ||||||||||| |||||||||| ||||||||||||||||||||||||||||||| |||||
Sbjct: 661 aataactggaagtatgcggcgg-aaagatgtgcgaaagctatacttgctaaaaa-cccta 718


Query: 788 aattattaattatgattgaatgtgttgaacaataaccctaaaaccgaaaaaggttatact 847
           |||||||||||||||||||| |||||||||||| |||||||||| |||||||||||||
Sbjct: 719 aattattaattatgattgaaggtgttgaacaat-accctaaaactgaaaaaggttataac 777


Query: 848 tattataccccggatgtatgggga 871
           ||| |||| |||||||||||||||
Sbjct: 778 tataatacaccggatgtatgggga 801
```

#######

Cphy1163-1 reverse sequence

Query= HR0ADKBY82ZA06_1163C1P17_1.SCF   1378   0   1378 SCF

>cphy1163

```
Query: 66   gtgtaatggatagtgatcTTAatggtgatggtgatgatgttttccataatattgccctaa 125
            ||||||||||||||||||||||||||||||||||||||||||||||||||||||||||||
Sbjct: 1368 gtgtaatggatagtgatcttaatggtgatggtgatgatgttttccataatattgccctaa 1309


Query: 126  tgatataccatttacaccaagaggtgtctgatggtctagaccaataaatttaccatttga 185
            ||||||||||||||||||||||||||||||||||||||||||||||||||||||||||||
Sbjct: 1308 tgatataccatttacaccaagaggtgtctgatggtctagaccaataaatttaccatttga 1249


Query: 186  ctgccataatgcaggttttaataaagcgtattttgcttcatcccaagtttgccaatcata 245
            ||||||||||||||||||||||||||||||||||||||||||||||||||||||||||||
Sbjct: 1248 ctgccataatgcaggttttaataaagcgtattttgcttcatcccaagtttgccaatcata 1189


Query: 246  tcctagtaaacctccagtatcccctgagttcggattgatacaccagaatgtatggtggat 305
            ||||||||||||||||||||||||||||||||||||||||||||||||||||||||||||
Sbjct: 1188 tcctagtaaacctccagtatcccctgagttcggattgatacaccagaatgtatggtggat 1129
```

```
Query: 306  acgattatttactatataatctcttaataatgtcatccatttctggttctttccaccatc 365
            ||||||||||||||||||||||||||||||||||||||||||||||||||||||||||||
Sbjct: 1128 acgattatttactatataatctcttaataatgtcatccatttctggttctttccaccatc 1069


Query: 366  cataaaacctccccactcacctatcaaaagtggtgcaatacctttatctttaatatatgc 425
            ||||||||||||||||||||||||||||||||||||||||||||||||||||||||||||
Sbjct: 1068 cataaaacctccccactcacctatcaaaagtggtgcaatacctttatctttaatatatgc 1009


Query: 426  ccaagtattataccaataatcatctaatagggtctgagttgtaaaatccttatcaaacca 485
            ||||||||||||||||||||||||||||||||||||||||||||||||||||||||||||
Sbjct: 1008 ccaagtattataccaataatcatctaatagggtctgagttgtaaaatccttatcaaacca 949


Query: 486  tggttggttgtatacggaaggaccatagtcatgaggggaatagacgatctgactattgag 545
            ||||||||||||||||||||||||||||||||||||||||||||||||||||||||||||
Sbjct: 948  tggttggttgtatacggaaggaccatagtcatgaggggaatagacgatctgactattgag 889


Query: 546  agtgcctatattaattggataatcctttactcctcttaaatttccaccccaccaagcact 605
            ||||||||||||||||||||||||||||||||||||||||||||||||||||||||||||
Sbjct: 888  agtgcctatattaattggataatcctttactcctcttaaatttccaccccaccaagcact 829


Query: 606  ataccatggagactgatcaccagtagctccccatacatccggtgtattatagttataacc 665
            ||||||||||||||||||||||||||||||||||||||||||||||||||||||||||||
Sbjct: 828  ataccatggagactgatcaccagtagctccccatacatccggtgtattatagttataacc 769


Query: 666  tttttcagttttagggtattgttcaacaccttcaatcataattaataatttagggttttt 725
            ||||||||||||||||||||||||||||||||||||||||||||||||||||||||||||
Sbjct: 768  tttttcagttttagggtattgttcaacaccttcaatcataattaataatttagggttttt 709


Query: 726  agcaagtatagctttcgcacatctttccgccgcatacttccagttattctcatcttgtgg 785
            |||||||||||||||||||||||||||||||||||||||||||||||||||||| |||||
Sbjct: 708  agcaagtatagctttcgcacatctttccgccgcatacttccagttattctcatc-tgtgg 650


Query: 786  aattatcccatttgctatattagtaggtgttgaagtactatatcccccctctgtccctga 845
            |||||||||||||||||||||||||||||||| |||||||||| ||||||||||| |||
Sbjct: 649  aattatcccatttgctatattagtaggtgttgtagtactatat-cccctctgtccatga 591


Query: 846  ggttcatttttatatcaaatgccagattagtatcgcctttttgtattttatcacccag 905
            ||||||||||||||||||||||||||| |||||||| | |||||||| ||||||| ||||
Sbjct: 590  ggttcatttttatatcaaatgccagaatagtatcgtcattttgta-tttatcagccag 532


Query: 906  ccatgcttgagtattgaatcccttatctgtccttatttcccctgcagtaattcgaattaa 965
            |||||||||||||||||  | |||||||||| ||||||| |||||||| | ||| | | |
Sbjct: 531  ccatgcttgagtattgatccacttatctgtcgttatttcacctgcagt-agtcgtagtta 473


Query: 966  accctacccataacggaaagttaatgacctgaattatttgcctcccggattataaaactc 1025
            ||||   |||||||||| |||| ||||||||||||||||| | ||||| ||| ||  ||
```

```
Sbjct: 472  acccataccataacggatagtt-atgacctgaattatttgcat-ccggactatgaacatc 415


Query: 1026 taccattaactttgatccccaattgtttgcagaatccccataaggataaaaaatatttcc 1085
             ||||||| |||||||||||||||||||||||| || ||||||| ||||  ||||||||||
Sbjct: 414  taccatt-actttgatccccaattgtttgcagtat-cccataatgatatcaaatatttcc 357


Query: 1086 ataccatttt 1096
             |||| ||||||
Sbjct: 356  atactatttt 346


#######

Cphy1687-1 forward sequence

Query= HR0ADKBY82ZB01_1687C1P16_1.SCF   1377      0   1377  SCF

>Cphy1687


Query: 67   aaagaaggagataggatcATGcatcatcaccatcaccatactaccagatatgaatgtgag 126
            ||||||||||||||||||||||||||||||||||||||||||||||||||||||||||||
Sbjct: 1    aaagaaggagataggatcatgcatcatcaccatcaccatactaccagatatgaatgtgag 60


Query: 127  aatatgacccttggcggtcagtacgccggaaaaattagttcgccgttcactggagtcgct 186
            ||||||||||||||||||||||||||||||||||||||||||||||||||||||||||||
Sbjct: 61   aatatgacccttggcggtcagtacgccggaaaaattagttcgccgttcactggagtcgct 120


Query: 187  ttatacgctaataatgattactgccaaaccggtaatattacctggaacaatacccagaag 246
            ||||||||||||||||||||||||||||||||||||||||||||||||||||||||||||
Sbjct: 121  ttatacgctaataatgattactgccaaaccggtaatattacctggaacaatacccagaag 180


Query: 247  actattagtatcagggggagttcaagtaattcgaataccgctacagtggttgtgaagatg 306
            ||||||||||||||||||||||||||||||||||||||||||||||||||||||||||||
Sbjct: 181  actattagtatcagggggagttcaagtaattcgaataccgctacagtggttgtgaagatg 240


Query: 307  aacggaaatgaaatgggtaaggtgaatttcaccggtactactcctacggtacagtccttt 366
            ||||||||||||||||||||||||||||||||||||||||||||||||||||||||||||
Sbjct: 241  aacggaaatgaaatgggtaaggtgaatttcaccggtactactcctacggtacagtccttt 300


Query: 367  acctgcaccccgcaatccggttcctacccggtccagcttattgtgaccaacgataatggg 426
            ||||||||||||||||||||||||||||||||||||||||||||||||||||||||||||
Sbjct: 301  acctgcaccccgcaatccggttcctacccggtccagcttattgtgaccaacgataatggg 360


Query: 427  acttgggatgtctacgtggattacttggagatcagcgataccagtagtggtggaggcggc 486
            ||||||||||||||||||||||||||||||||||||||||||||||||||||||||||||
Sbjct: 361  acttgggatgtctacgtggattacttggagatcagcgataccagtagtggtggaggcggc 420


Query: 487  accagcggtaatgtttatcttacctttgatgacggaccacttaatggaaactcaccaacc 546
```

```
            ||||||||||||||||||||||||||||||||||||||||||||||||||||||||||||
Sbjct: 421  accagcggtaatgtttatcttacctttgatgacggaccacttaatggaaactcaccaacc 480


Query: 547  ctgattaataacctaaaaagtgcaggatgcagccaggccactttattcgtctggggtaac 606
            ||||||||||||||||||||||||||||||||||||||||||||||||||||||||||||
Sbjct: 481  ctgattaataacctaaaaagtgcaggatgcagccaggccactttattcgtctggggtaac 540


Query: 607  aggatcagcagcaaccagactggctggaatgcctacttaaattccggatttagcctccag 666
            ||||||||||||||||||||||||||||||||||||||||||||||||||||||||||||
Sbjct: 541  aggatcagcagcaaccagactggctggaatgcctacttaaattccggatttagcctccag 600


Query: 667  aatcatagctggacgcactcgcatatgaccagctggagctatcaacaggtctataacgac 726
            ||||||||||||||||||||||||||||||||||||||||||||||||||||||||||||
Sbjct: 601  aatcatagctggacgcactcgcatatgaccagctggagctatcaacaggtctataacgac 660


Query: 727  ctccaacaatgtaaccaagctatccaaaacgccgggaaaccaaagcctacgaagataagg 786
            ||||||||||||||||||||||||||||||||||||||||||||||||||||||||||||
Sbjct: 661  ctccaacaatgtaaccaagctatccaaaacgccgggaaaccaaagcctacgaagataagg 720


Query: 787  cttccttatctcgagagcaactcaacgatacagcaagcttgttcagcactgggcctgtcg 846
            ||||||||||||||||||||||||||||||||||||||||||||||||||||||||||||
Sbjct: 721  cttccttatctcgagagcaactcaacgatacagcaagcttgttcagcactgggcctgtcg 780


Query: 847  atcgtcagtcctaatgttgacactcaggactggaacggcgcccgcacacagtccatcgtt 906
            |||||||||||||||||||||||||||||||||||||||||| |||||||||| ||||||
Sbjct: 781  atcgtcagtcctaatgttgacactcaggactggaacggcgccagcacacagtcaatcgtt 840


Query: 907  aatgcttgccacaacttggaggcccggtggtaacccgttgatgcatgagggatatcagaa 966
            ||||||||| |||||||| ||| ||||||||||||||||||||||||| ||||||||||
Sbjct: 841  aatgcttgcaacaacttgcagg-ccggtggtaacccgttgatgcatgatggatatcagac 899


Query: 967  cactaactcggctataaccccatcgtttcgaaatctaaggaaccg 1012
             ||||||||||||||| || ||||||||||| ||||||||||||||
Sbjct: 900  gactaactcggctatagccaccatcgtttcg-aatctaaggaaccg 944

#######

Cphy1687-1 reverse sequence

Query= HR0ADKBY82ZB01_1687C1P17_1.SCF   1399      0   1399  SCF

>Cphy1687

Query: 65   gtgtaatggatagtgatcTTAgtattgtgcaaaaccgagaccacggttccttagattcga 124
            ||||||||||||||||||||||||||||||||||||||||||||||||||||||||||||
Sbjct: 987  gtgtaatggatagtgatcttagtattgtgcaaaaccgagaccacggttccttagattcga 928


Query: 125  aacgatggtggctatagccgagttagtcgtctgatatccatcatgcatcaacgggttacc 184
            ||||||||||||||||||||||||||||||||||||||||||||||||||||||||||||
```

```
Sbjct: 927  aacgatggtggctatagccgagttagtcgtctgatatccatcatgcatcaacgggttacc 868

Query: 185  accggcctgcaagttgttgcaagcattaacgattgactgtgtgctggcgccgttccagtc 244
            ||||||||||||||||||||||||||||||||||||||||||||||||||||||||||||
Sbjct: 867  accggcctgcaagttgttgcaagcattaacgattgactgtgtgctggcgccgttccagtc 808

Query: 245  ctgagtgtcaacattaggactgacgatcgacaggcccagtgctgaacaagcttgctgtat 304
            ||||||||||||||||||||||||||||||||||||||||||||||||||||||||||||
Sbjct: 807  ctgagtgtcaacattaggactgacgatcgacaggcccagtgctgaacaagcttgctgtat 748

Query: 305  cgttgagttgctctcgagataaggaagccttatcttcgtaggctttggtttcccggcgtt 364
            ||||||||||||||||||||||||||||||||||||||||||||||||||||||||||||
Sbjct: 747  cgttgagttgctctcgagataaggaagccttatcttcgtaggctttggtttcccggcgtt 688

Query: 365  ttggatagcttggttacattgttggaggtcgttatagacctgttgatagctccagctggt 424
            ||||||||||||||||||||||||||||||||||||||||||||||||||||||||||||
Sbjct: 687  ttggatagcttggttacattgttggaggtcgttatagacctgttgatagctccagctggt 628

Query: 425  catatgcgagtgcgtccagctatgattctggaggctaaatccggaatttaagtaggcatt 484
            ||||||||||||||||||||||||||||||||||||||||||||||||||||||||||||
Sbjct: 627  catatgcgagtgcgtccagctatgattctggaggctaaatccggaatttaagtaggcatt 568

Query: 485  ccagccagtctggttgctgctgatcctgttaccccagacgaataaagtggcctggctgca 544
            ||||||||||||||||||||||||||||||||||||||||||||||||||||||||||||
Sbjct: 567  ccagccagtctggttgctgctgatcctgttaccccagacgaataaagtggcctggctgca 508

Query: 545  tcctgcactttttaggttattaatcagggttggtgagtttccattaagtggtccgtcatc 604
            ||||||||||||||||||||||||||||||||||||||||||||||||||||||||||||
Sbjct: 507  tcctgcactttttaggttattaatcagggttggtgagtttccattaagtggtccgtcatc 448

Query: 605  aaaggtaagataaacattaccgctggtgccgcctccaccactactggtatcgctgatctc 664
            ||||||||||||||||||||||||||||||||||||||||||||||||||||||||||||
Sbjct: 447  aaaggtaagataaacattaccgctggtgccgcctccaccactactggtatcgctgatctc 388

Query: 665  caagtaatccacgtagacatcccaagtcccattatcgttggtcacaataagctggaccgg 724
            ||||||||||||||||||||||||||||||||||||||||||||||||||||||||||||
Sbjct: 387  caagtaatccacgtagacatcccaagtcccattatcgttggtcacaataagctggaccgg 328

Query: 725  gtaggaaccggattgcggggtgcaggtaaaggactgtaccgtaggagtagtaccggtgaa 784
            ||||||||||||||||||||||||||||||||||||||||||||||||||||||||||||
Sbjct: 327  gtaggaaccggattgcggggtgcaggtaaaggactgtaccgtaggagtagtaccggtgaa 268

Query: 785  attcaccttacccatttcatttccgttcatcttcacaaccactgtagcggtattcgaatt 844
            ||||||||||||||||||||||||||||||||||||||||||||||||||||||||||||
Sbjct: 267  attcaccttacccatttcatttccgttcatcttcacaaccactgtagcggtattcgaatt 208
```

```
Query: 845  acttgaactccccctgatactaatagtcttctgggtattgttccaggtaatattaccggt 904
            ||||||||||||||||||||||||||||||||||||||||||||||||||||||||||||
Sbjct: 207  acttgaactccccctgatactaatagtcttctgggtattgttccaggtaatattaccggt 148


Query: 905  ttggcagtaatcattattagcgtataaagcaactccagtgaacggcaaaataatttttcc 964
            |||||||||||||||||||||||||||||| ||||||||||||||| || ||||||||||
Sbjct: 147  ttggcagtaatcattattagcgtataaagcgactccagtgaacggcgaactaatttttcc 88


Query: 965  gggctactgacccccaagggtcatattctcccattcatatctgg 1008
            ||  |||||||| ||||||||||||||||| |||||||||||||
Sbjct: 87   ggcgtactgaccgccaagggtcatattctcacattcatatctgg 44
```

#######

Cphy1718-1 forward sequence

Query= HR0ADKBY82ZB06_1718C1P16_1.SCF   1387   0   1387 SCF

>cphy1718

```
Query: 83   aaagaaggagataggatcATGaaaaatataccatgggaaccaagacctgttgattgtgag 142
            ||||||||||||||||||||||||||||||||||||||||||||||||||||||||||||
Sbjct: 1    aaagaaggagataggatcatgaaaaatataccatgggaaccaagacctgttgattgtgag 60


Query: 143  gatgttgtttggagatattcaaaaaatccgattatacatagaaatgaaatcaaaagaagc 202
            ||||||||||||||||||||||||||||||||||||||||||||||||||||||||||||
Sbjct: 61   gatgttgtttggagatattcaaaaaatccgattatacatagaaatgaaatcaaaagaagc 120


Query: 203  aatagtatatttaatagtgcggtggtaccttttaaagatggttatgcaggggtatttagg 262
            ||||||||||||||||||||||||||||||||||||||||||||||||||||||||||||
Sbjct: 121  aatagtatatttaatagtgcggtggtaccttttaaagatggttatgcaggggtatttagg 180


Query: 263  tgtgatgataaaagaagagaaatgttactccatgctggatttagtgtagatggagtgaag 322
            ||||||||||||||||||||||||||||||||||||||||||||||||||||||||||||
Sbjct: 181  tgtgatgataaaagaagagaaatgttactccatgctggatttagtgtagatggagtgaag 240


Query: 323  tggaatataaatcctgaaccaatcgagtttcaatctgaagtagaggatagcgaaccattt 382
            ||||||||||||||||||||||||||||||||||||||||||||||||||||||||||||
Sbjct: 241  tggaatataaatcctgaaccaatcgagtttcaatctgaagtagaggatagcgaaccattt 300


Query: 383  gaatatgggtatgatccaagagtatgtttcattgaagatagatattatgttacttggtgt 442
            ||||||||||||||||||||||||||||||||||||||||||||||||||||||||||||
Sbjct: 301  gaatatgggtatgatccaagagtatgtttcattgaagatagatattatgttacttggtgt 360


Query: 443  aatggatatcatggtcctaccattggaattgcatatacctttgattttgaaacctttcac 502
            ||||||||||||||||||||||||||||||||||||||||||||||||||||||||||||
Sbjct: 361  aatggatatcatggtcctaccattggaattgcatatacctttgattttgaaacctttcac 420


Query: 503  cagatggaaaatatatttttaccttataacaggaatggtgttctgtttccaagaaaaatc 562
```

```
             ||||||||||||||||||||||||||||||||||||||||||||||||||||||||||||
Sbjct: 421  cagatggaaaatatattttaccttataacaggaatggtgttctgtttccaagaaaaatc 480


Query: 563  aatgagaaatatgcgatcttaagcagaccgagtgatacgggacatactgcttttggtgat 622
             |||| |||||||||||||||||||||||||||||||||||||||||||||||||||||||
Sbjct: 481  aatgggaaatatgcgatcttaagcagaccgagtgatacgggacatactgcttttggtgat 540


Query: 623  atttttatagtgagtcaccggatttgatccattggggaagacatcgtcacgtaatggca 682
             ||||||||||||||||||||||||||||||||||||||||||||||||||||||||||||
Sbjct: 541  atttttatagtgagtcaccggatttgatccattggggaagacatcgtcacgtaatggca 600


Query: 683  cctagaggttggtggcaatccacaaaaattggagccggacctgtaccgattgaaacaaaa 742
             ||||||||||||||||||||||||||||||||||||||||||||||||||||||||||||
Sbjct: 601  cctagaggttggtggcaatccacaaaaattggagccggacctgtaccgattgaaacaaaa 660


Query: 743  gagggttggctactgttttatcatggtgtactgacctcctgtaatgggtatgtgtatagt 802
             ||||||||||||||||||||||||||||||||||||||||||||||||||||||||||| 
Sbjct: 661  gagggttggctactgttttatcatggtgtactgacctcctgtaatgggtatgtgtatagc 720


Query: 803  tttggagcaccaatgctagacccttatgagccttggaaagtaaagtatagaaacgaacct 862
             ||||||||| |||||||||||| | ||||||||||||||||||||||||||  |||||  
Sbjct: 721  tttggagcagcaatgctagaccttaatgagccttggaaagtaaagtatagaacagaacca 780


Query: 863  tatttattatccccacaaacctttatatgaatgcgttggtgatgtaccaaaatgtgtttt 922
             ||||||||||| ||||||| | |||||||||||||||||||||||||||   ||||||
Sbjct: 781  tatttattatcaccacaaa-cattatatgaatgcgttggtgatgtaccaaatgttgtttt 839


Query: 923  cccctgggcacctttccttgaagaagaaagtgggaaaatttgcattttacatgggttgtg 982
             ||| || ||| |||| | ||||||||||||||| | || ||||| |||||   || ||||
Sbjct: 840  cccatgtgcagctttacatgaagaagaaagtggcagaa-ttgcaatttactatggctgtg 898


Query: 983  caaatactgttacgggggatgcttttaccttgagggaagaacctaattgatttt 1037
             || |||||||||| || | |||| |||||| ||||||| | | ||||||||||||
Sbjct: 899  cagatactgttacaggtgtatgc-tttaccatgagggatg-agctaattgatttt 951
```

#######

Cphy1718-1 reverse sequence

Query= HR0ADKBY82ZB06_1718C1P17_1.SCF   1374   0   1374 SCF

>cphy1718

```
Query: 65   gtgtaatggatagtgatcTTAatggtgatggtgatgatgaagcttggaattctttttcat 124
             ||||||||||||||||||||||||||||||||||||||||||||||||||||||||||||
Sbjct: 1011 gtgtaatggatagtgatcttaatggtgatggtgatgatgaagcttggaattctttttcat 952


Query: 125  aaaatcaattagctcatccctcatggtaaagcatacacctgtaacagtatctgcacagcc 184
```

```
            ||||||||||||||||||||||||||||||||||||||||||||||||||||||||||||
Sbjct: 951  aaaatcaattagctcatccctcatggtaaagcatacacctgtaacagtatctgcacagcc 892

Query: 185  atagtaaattgcaattctgccactttcttcttcatgtaaagctgcacatgggaaaacaac 244
            ||||||||||||||||||||||||||||||||||||||||||||||||||||||||||||
Sbjct: 891  atagtaaattgcaattctgccactttcttcttcatgtaaagctgcacatgggaaaacaac 832

Query: 245  atttggtacatcaccaacgcattcatataatgtttgtggtgataataaatatggttctgt 304
            ||||||||||||||||||||||||||||||||||||||||||||||||||||||||||||
Sbjct: 831  atttggtacatcaccaacgcattcatataatgtttgtggtgataataaatatggttctgt 772

Query: 305  tctatactttactttccaaggctcattaaggtctagcattgctgctccaaagctatacac 364
            ||||||||||||||||||||||||||||||||||||||||||||||||||||||||||||
Sbjct: 771  tctatactttactttccaaggctcattaaggtctagcattgctgctccaaagctatacac 712

Query: 365  atacccattacaggaggtcagtacaccatgataaaacagtagccaaccctcttttgtttc 424
            ||||||||||||||||||||||||||||||||||||||||||||||||||||||||||||
Sbjct: 711  atacccattacaggaggtcagtacaccatgataaaacagtagccaaccctcttttgtttc 652

Query: 425  aatcggtacaggtccggctccaattttttgtggattgccaccaacctctaggtgccattac 484
            ||||||||||||||||||||||||||||||||||||||||||||||||||||||||||||
Sbjct: 651  aatcggtacaggtccggctccaattttttgtggattgccaccaacctctaggtgccattac 592

Query: 485  gtgacgatgtcttccccaatggatcaaatccggtgactcactataaaaaatatcaccaaa 544
            ||||||||||||||||||||||||||||||||||||||||||||||||||||||||||||
Sbjct: 591  gtgacgatgtcttccccaatggatcaaatccggtgactcactataaaaaatatcaccaaa 532

Query: 545  agcagtatgtcccgtatcactcggtctgcttaagatcgcatatttctcattgatttttct 604
            |||||||||||||||||||||||||||||||||||||||||||||| |||||||||||||
Sbjct: 531  agcagtatgtcccgtatcactcggtctgcttaagatcgcatatttcccattgatttttct 472

Query: 605  tggaaacagaacaccattcctgttataaggtaaaaatatattttccatctggtgaaaggt 664
            ||||||||||||||||||||||||||||||||||||||||||||||||||||||||||||
Sbjct: 471  tggaaacagaacaccattcctgttataaggtaaaaatatattttccatctggtgaaaggt 412

Query: 665  ttcaaaatcaaaggtatatgcaattccaatggtaggaccatgatatccattacaccaagt 724
            ||||||||||||||||||||||||||||||||||||||||||||||||||||||||||||
Sbjct: 411  ttcaaaatcaaaggtatatgcaattccaatggtaggaccatgatatccattacaccaagt 352

Query: 725  aacataatatctatcttcaatgaaacatactcttggatcatacccatattcaaatggttc 784
            ||||||||||||||||||||||||||||||||||||||||||||||||||||||||||||
Sbjct: 351  aacataatatctatcttcaatgaaacatactcttggatcatacccatattcaaatggttc 292

Query: 785  gctatcctctacttcagattgaaactcgattggttcaggatttatattccacttcactcc 844
            ||||||||||||||||||||||||||||||||||||||||||||||||||||||||||||
Sbjct: 291  gctatcctctacttcagattgaaactcgattggttcaggatttatattccacttcactcc 232
```

```
Query: 845  atctacactaaaatcagcatggagtaacatttctcttcttttatcatcacacctaaatac 904
            ||||||||||||  ||||||||||||||||||||||||||||||||||||||||||||||
Sbjct: 231  atctacactaaatccagcatggagtaacatttctcttcttttatcatcacacctaaatac 172


Query: 905  ccctgcataaccatctttaaaaggtaccaccgcactaataaatatactattgcttctttt 964
            ||||||||||||||||||||||||||||||||||||| ||||||||||||||||||||||
Sbjct: 171  ccctgcataaccatctttaaaaggtaccaccgcactattaaatatactattgcttctttt 112


Query: 965  gatttcatttctatgtataatcggatttttgattatctccaaacaacatcctcacaatc 1024
            |||||||||||||||||||||||||||||||| |||||||||||||||||||||||||||
Sbjct: 111  gatttcatttctatgtataatcggatttttgaatatctccaaacaacatcctcacaatc 52


Query: 1025 aacaggtccttggttccccagggtaaatttttcatgatcccatcttccttcttt 1079
            ||||||| ||||||| |||| |||| | ||||||||||||| ||| |||||||||
Sbjct: 51   aacaggt-cttggtt-cccatggtata-tttttcatgatcctatc-tccttcttt 1
```

#######

Cphy1719-1 forward sequence

```
Query= HR0ADKBY82ZC01_1719C1P16_1.SCF   1373      0   1373  SCF

>Cphy1719

Query: 68   aaagaaggagataggatcATGgaaacttcaaaagcgatagaagaattatttgagacggta 127
            ||||||||||||||||||||||||||||||||||||||||||||||||||||||||||||
Sbjct: 1    aaagaaggagataggatcatggaaacttcaaaagcgatagaagaattatttgagacggta 60


Query: 128  aaacagaaattaacgggtatggaaaaagtatatatggcatttgaaaaatgttttctcaat 187
            ||||||||||||||||||||||||||||||||||||||||||||||||||||||||||||
Sbjct: 61   aaacagaaattaacgggtatggaaaaagtatatatggcatttgaaaaatgttttctcaat 120


Query: 188  acaatcaccactacagttaaaaggttagacgatggttcttcctatgtaattacaggtgat 247
            ||||||||||||||||||||||||||||||||||||||||||||||||||||||||||||
Sbjct: 121  acaatcaccactacagttaaaaggttagacgatggttcttcctatgtaattacaggtgat 180


Query: 248  attcctgctatgtggcttagagattccacttgtcaaatccgcccgtatctagtattagca 307
            ||||||||||||||||||||||||||||||||||||||||||||||||||||||||||||
Sbjct: 181  attcctgctatgtggcttagagattccacttgtcaaatccgcccgtatctagtattagca 240


Query: 308  aataaagatttagcgatagctcagatgataaaggggctaattcataggcaatttaaatac 367
            ||||||||||||||||||||||||||||||||||||||||||||||||||||||||||||
Sbjct: 241  aataaagatttagcgatagctcagatgataaaggggctaattcataggcaatttaaatac 300


Query: 368  atacggctagatccttatgcaaatgcctttaacgaaagcgccaatggacattgctgggaa 427
            ||||||||||||||||||||||||||||||||||||||||||||||||||||||||||||
Sbjct: 301  atacggctagatccttatgcaaatgcctttaacgaaagcgccaatggacattgctgggaa 360
```

```
Query: 428  caagatgaatgcggcatgggaccttgggtatgggaacgaaaatatgagattgattcttta 487
            ||||||||||||||||||||||||||||||||||||||||||||||||||||||||||||
Sbjct: 361  caagatgaatgcggcatgggaccttgggtatgggaacgaaaatatgagattgattcttta 420


Query: 488  tgctttccaatacagttagcgtggttatattggaaaacgacaggtgataccactcacttc 547
            ||||||||||||||||||||||||||||||||||||||||||||||||||||||||||||
Sbjct: 421  tgctttccaatacagttagcgtggttatattggaaaacgacaggtgataccactcacttc 480


Query: 548  gataaaaattttaagaatgccattaaagtaattatgcatacatggaagctggagcaaaat 607
            ||||||||||||||||||||||||||||||||||||||||||||||||||||||||||||
Sbjct: 481  gataaaaattttaagaatgccattaaagtaattatgcatacatggaagctggagcaaaat 540


Query: 608  catgaggggaaatctcagtaccattttattcgaaaaaatacctttttaaggatacgtta 667
            ||||||||||||||||||||||||||||||||||||||||||||||||||||||||||||
Sbjct: 541  catgaggggaaatctcagtaccattttattcgaaaaaatacctttttaaggatacgtta 600


Query: 668  tcgagagaagggaaaggggcgttagtaaagccaaatatcggattgacttggtcaggcttt 727
            ||||||||||||||||||||||||||||||||||||||||||||||||||||||||||||
Sbjct: 601  tcgagagaagggaaaggggcgttagtaaagccaaatatcggattgacttggtcaggcttt 660


Query: 728  cgtccaagtgatgatgcttgtacctatggctatttaattccatcgaacatgttcgctacc 787
            ||||||||||||||||||||||||||||||||||||||||||||||||||||||||||||
Sbjct: 661  cgtccaagtgatgatgcttgtacctatggctatttaattccatcgaacatgttcgctacc 720


Query: 788  gttgtattaaactatgtggaagagattgcaacagaggttctaaaagatgacagcatagca 847
            ||||||||||||||||||||||||||||||||||||||||||||||||||||||||||||
Sbjct: 721  gttgtattaaactatgtggaagagattgcaacagaggttctaaaagatgacagcatagca 780


Query: 848  aaagaagctcttctgttaagaaaagaaatcggggacggatttgagaattaccgtattgtg 907
            ||||||||||||||||||||||||||||||||||||||| ||||||||||| ||||||||
Sbjct: 781  aaagaagctcttctgttaagaaaagaaatcggggacggaattgagaattacggtattgtg 840


Query: 908  aacacaaaggaatttggccgcatctattgctatgaaacagatgggtatggcccatataat 967
            |||||| ||||||||||||||||||||   |||||||||||||||||||||| |||||||
Sbjct: 841  aacacagaggaatttggccgcatctatgcatatgaaacagatgggtatggccaatataat 900


Query: 968  ttaatggatgacgccaatgtaccaagtttactatctattctttaaatcg 1016
            |||||||||||||| ||||||||||||||||||||||||| ||| ||||
Sbjct: 901  ttaatggatgacgcgaatgtaccaagtttactatctattccttatatcg 949
```

#######

Cphy1719-1 reverse sequence

Query= HR0ADKBY82ZC01_1719C1P17_1.SCF   1386   0   1386 SCF

>Cphy1719

```
Query: 65   gtgtaatggatagtgatcTTAatggtgatggtgatgatgaattcctgcctttataccaca 124
            ||||||||||||||||||||||||||||||||||||||||||||||||||||||||||||
Sbjct: 1338 gtgtaatggatagtgatcttaatggtgatggtgatgatgaattcctgcctttataccaca 1279

Query: 125  aaaatctagtaccaattcagcaaaaagtgagttcgcccatgcaaaccattctctggtgta 184
            ||||||||||||||||||||||||||||||||||||||||||||||||||||||||||||
Sbjct: 1278 aaaatctagtaccaattcagcaaaaagtgagttcgcccatgcaaaccattctctggtgta 1219

Query: 185  tattacggcatcatccgcataaattccttcatgcattaacaaatcttcacctaatgtatt 244
            ||||||||||||||||||||||||||||||||||||||||||||||||||||||||||||
Sbjct: 1218 tattacggcatcatccgcataaattccttcatgcattaacaaatcttcacctaatgtatt 1159

Query: 245  catgattcggtttatacactgaagcttatcttctactttctcgctcgttaaaccttccat 304
            ||||||||||||||||||||||||||||||||||||||||||||||||||||||||||||
Sbjct: 1158 catgattcggtttatacactgaagcttatcttctactttctcgctcgttaaaccttccat 1099

Query: 305  tgcaaccgctatatgccagacatagtttgatagagtatgagcacttccgattcctgataa 364
            ||||||||||||||||||||||||||||||||||||||||||||||||||||||||||||
Sbjct: 1098 tgcaaccgctatatgccagacatagtttgatagagtatgagcacttccgattcctgataa 1039

Query: 365  catcttaccttcaaagtaataagggtttgaatgacttaatataaatcgccttgtattatt 424
            ||||||||||||||||||||||||||||||||||||||||||||||||||||||||||||
Sbjct: 1038 catcttaccttcaaagtaataagggtttgaatgacttaatataaatcgccttgtattatt 979

Query: 425  agcaacctcttcttcctttcccacatatccgatataaggaatagatagtaaacttggtac 484
            ||||||||||||||||||||||||||||||||||||||||||||||||||||||||||||
Sbjct: 978  agcaacctcttcttcctttcccacatatccgatataaggaatagatagtaaacttggtac 919

Query: 485  attcgcgtcatccattaaattatattggccatacccatctgtttcatatgcatagatgcg 544
            ||||||||||||||||||||||||||||||||||||||||||||||||||||||||||||
Sbjct: 918  attcgcgtcatccattaaattatattggccatacccatctgtttcatatgcatagatgcg 859

Query: 545  gccaaattcctctgtgttcacaataccgtaattctcaattccgtccccgatttcttttct 604
            ||||||||||||||||||||||||||||||||||||||||||||||||||||||||||||
Sbjct: 858  gccaaattcctctgtgttcacaataccgtaattctcaattccgtccccgatttcttttct 799

Query: 605  taacagaagagcttcttttgctatgctgtcatcttttagaacctctgttgcaatctcttc 664
            ||||||||||||||||||||||||||||||||||||||||||||||||||||||||||||
Sbjct: 798  taacagaagagcttcttttgctatgctgtcatcttttagaacctctgttgcaatctcttc 739

Query: 665  cacatagtttaatacaacggtagcgaacatgttcgatggaattaaatagccataggtaca 724
            ||||||||||||||||||||||||||||||||||||||||||||||||||||||||||||
Sbjct: 738  cacatagtttaatacaacggtagcgaacatgttcgatggaattaaatagccataggtaca 679

Query: 725  agcatcatcacttggacgaaagcctgaccaagtcaatccgatatttggctttactaacgc 784
            ||||||||||||||||||||||||||||||||||||||||||||||||||||||||||||
```

```
Sbjct: 678  agcatcatcacttggacgaaagcctgaccaagtcaatccgatatttggctttactaacgc 619


Query: 785  ccctttcccttctctcgataacgtatccttaaaaaaggtattttttcgaataaaatggta 844
             ||||||||||||||||||||||||||||||||||||||||||||||||||||||||||||
Sbjct: 618  ccctttcccttctctcgataacgtatccttaaaaaaggtattttttcgaataaaatggta 559


Query: 845  ctgaaatttcccctcatgattttgctccagcttccaggtatgcataattacttttatggc 904
             |||| ||||||||||||||||||||||||||||||| ||||||||||||||||| |||||
Sbjct: 558  ctgagatttcccctcatgattttgctccagcttccatgtatgcataattactttaatggc 499


Query: 905  cttcttaaaattttatccaagtgagtggtatcacctggtcgtttccaattataaccacc 964
              |||||||||||||||| |||||||||||||||||| ||||||| | | |||||||| |
Sbjct: 498  attcttaaaattttatcgaagtgagtggtatcacct-gtcgttttccaatataacca-c 441


Query: 965  gctaacggtattgggaaagcataaagaaatcaatcccaaatttcgttccccatacccaa 1024
             |||||| ||||| ||||||||||||| |||||||| || ||||||||| |||||||||||
Sbjct: 440  gctaactgtatt-ggaaagcataaag-aatcaatctcatatttcgtt-cccatacccaa 384


Query: 1025 ggtcccatgccgca 1038
             ||||||||||||||
Sbjct: 383  ggtcccatgccgca 370


#######

Cphy1720-2 forward sequence

Query= HR0ADKBY82ZC07_1720C2P16_1.SCF   1369      0   1369  SCF

>cphy1720

Query: 69  aaagaaggagataggatcATGttttatacagagcgaaaattggaaagaagaattgatgaa 128
            ||||||||||||||||||||||||||||||||||||||||||||||||||||||||||||
Sbjct: 1   aaagaaggagataggatcatgttttatacagagcgaaaattggaaagaagaattgatgaa 60


Query: 129 ttaagcaattatcgatatagagatgtgatatcttttcaggagttatatgtaaaagaggat 188
            ||||||||||||||||||||||||||||||||||||||||||||||||||||||||||||
Sbjct: 61  ttaagcaattatcgatatagagatgtgatatcttttcaggagttatatgtaaaagaggat 120


Query: 189 gtcgagaaattagtaagaccagaggtaccaacaacctttggcgattttaagacaatgaat 248
            ||||||||||||||||||||||||||||||||||||||||||||||||||||||||||||
Sbjct: 121 gtcgagaaattagtaagaccagaggtaccaacaacctttggcgattttaagacaatgaat 180


Query: 249 gttggtgaaacctggtctggccgcgattgttatctttggctacataaggaagttaccgta 308
            ||||||||||||||||||||||||||||||||||||||||||||||||||||||||||||
Sbjct: 181 gttggtgaaacctggtctggccgcgattgttatctttggctacataaggaagttaccgta 240


Query: 309 cctgctgattgggatnnnnnnngagtggtaggtatttttgatttcggaaataccggagct 368
            |||||||||||||||       ||||||||||||||||||||||||||||||||||||||
```

```
Sbjct: 241 cctgctgattgggataaaaaaagagtggtaggtatttttgatttcggaaataccggagct 300


Query: 369 ggtaataattctggttttgaatccctattgtacctaaatgagcaaccctatcagggagtt 428
           ||||||||||||||||||||||||||||||||||||||||||||||||||||||||||||
Sbjct: 301 ggtaataattctggttttgaatccctattgtacctaaatgagcaaccctatcagggagtt 360


Query: 429 gatgtaaatcataaagaagnnnnnnngaaggaagatttggttggtaagaacgtaaacttc 488
           |||||||||||||||||||       ||||||||||||||||||||||||||||||||||
Sbjct: 361 gatgtaaatcataaagaagtttttttgaaggaagatttggttggtaagaacgtaaacttc 420


Query: 489 acatttcgtttgtggtcaggacttgaaggcggaggagtaccacatgcacaggagcacaaa 548
           ||||||||||||||||||||||||||||||||||||||||||||||||||||||||||||
Sbjct: 421 acatttcgtttgtggtcaggacttgaaggcggaggagtaccacatgcacaggagcacaaa 480


Query: 549 ataaatcgtgctgacttagcatatttagatgagaaaacagacgatttatattatttatct 608
           ||||||||||||||||||||||||||||||||||||||||||||||||||||||||||||
Sbjct: 481 ataaatcgtgctgacttagcatatttagatgagaaaacagacgatttatattatttatct 540


Query: 609 ttgatgatacttgatactttaaaaaatcttgatgagaataatgccattcgcagcgaatta 668
           ||||||||||||||||||||||||||||||||||||||||||||||||||||||||||||
Sbjct: 541 ttgatgatacttgatactttaaaaaatcttgatgagaataatgccattcgcagcgaatta 600


Query: 669 aaaattcatttagataatgcctttaaattaattgactggtcttatccaaatagcgaagag 728
           ||||||||||||||||||||||||||||||||||||||||||||||||||||||||||||
Sbjct: 601 aaaattcatttagataatgcctttaaattaattgactggtcttatccaaatagcgaagag 660


Query: 729 ttttaccagtcggtgcacgaagcggatgattaccttaatgagaagattgatggtatggat 788
           ||||||||||||||||||||||||||||||||||||||||||||||||||||||||||||
Sbjct: 661 ttttaccagtcggtgcacgaagcggatgattaccttaatgagaagattgatggtatggat 720


Query: 789 aagaactctcttgtcaatgtcaaatgcataggccatacccatatcgatgtggcatggtta 848
           ||||||||||||||||||||||||||||||||||||||||||||||||||||||||||||
Sbjct: 721 aagaactctcttgtcaatgtcaaatgcataggccatacccatatcgatgtggcatggtta 780


Query: 849 tggagactaaagcatacaagagaaaaatgtgcacgttccttctctactgttttaagattg 908
           ||||||||||||||||||||||||||||||||||||||||||||||||||||||||||||
Sbjct: 781 tggagactaaagcatacaagagaaaaatgtgcacgttccttctctactgttttaagattg 840


Query: 909 atgaaaaagtatccggattacatattccctgcaaacgcagcccccaactattacaaaact 968
           ||| || ||||||||||||||||||| ||||||||||||| || |||||||   || ||
Sbjct: 841 atggaagagtatccggattacatatt-cctgcaaacgcag-ccacaactatacgaataca 898


Query: 969 tcaaaaatgacaatcctgaaata 991
           ||||||||||| |||||||||||
Sbjct: 899 tcaaaaatgactatcctgaaata 921
```

#######

Cphy1720-2 reverse sequence

Query= HR0ADKBY82ZC07_1720C2P17_1.SCF   1369   0   1369 SCF

>cphy1720

```
Query: 66   gtgtaatggatagtgatcTTAatggtgatggtgatgatgaaatattcccttagctaaaat 125
            ||||||||||||||||||||||||||||||||||||||||||||||||||||||||||||
Sbjct: 3186 gtgtaatggatagtgatcttaatggtgatggtgatgatgaaatattcccttagctaaaat 3127


Query: 126  cgtcttaatttcatatggttttacttctaaaatgatatccgaatcataatgggtatcatc 185
            ||||||||||||||||||||||||||||||||||||||||||||||||||||||||||||
Sbjct: 3126 cgtcttaatttcatatggttttacttctaaaatgatatccgaatcataatgggtatcatc 3067


Query: 186  catcggtctttcccgtaaatcagcctcaacccattctgttactttaaaaccagtctgtaa 245
            ||||||||||||||||||||||||||||||||||||||||||||||||||||||||||||
Sbjct: 3066 catcggtctttcccgtaaatcagcctcaacccattctgttactttaaaaccagtctgtaa 3007


Query: 246  cttaactatattctttgagccactataatcatggaagcgtacgactaagtactctttatc 305
            ||||||||||||||||||||||||||||||||||||||||||||||||||||||||||||
Sbjct: 3006 cttaactatattctttgagccactataatcatggaagcgtacgactaagtactctttatc 2947


Query: 306  ctctgactgtttcattgcatcaatcgtaatgtactcattatcgatggagacaaagctata 365
            ||||||||||||||||||||||||||||||||||||||||||||||||||||||||||||
Sbjct: 2946 ctctgactgtttcattgcatcaatcgtaatgtactcattatcgatggagacaaagctata 2887


Query: 366  attattaggaagaacacattcgccagcaatcgcttctaatttttgattgagtgcaaatgc 425
            ||||||||||||||||||||||||||||||||||||||||||||||||||||||||||||
Sbjct: 2886 attattaggaagaacacattcgccagcaatcgcttctaatttttgattgagtgcaaatgc 2827


Query: 426  ttccttctcagtttctcctttcacaaaatcacctttatgaggtaacaaagagtaggtgaa 485
            ||||||||||||||||||||||||||||||||||||||||||||||||||||||||||||
Sbjct: 2826 ttccttctcagtttctcctttcacaaaatcacctttatgaggtaacaaagagtaggtgaa 2767


Query: 486  agaatgctcgccttggtcttgtaaataatccggatgcatcgctgtcttaatcaaagttaa 545
            ||||||||||||||||||||||||||||||||||||||||||||||||||||||||||||
Sbjct: 2766 agaatgctcgccttggtcttgtaaataatccggatgcatcgctgtcttaatcaaagttaa 2707


Query: 546  tcgaagtaaattatcctttacatcatgaccatacttacaattattcagtaagcttacacc 605
            ||||||||||||||||||||||||||||||||||||||||||||||||||||||||||||
Sbjct: 2706 tcgaagtaaattatcctttacatcatgaccatacttacaattattcagtaagcttacacc 2647


Query: 606  ataattatattcggacaaatctacccatttgtgagccacagtctcaaacttagcttggtc 665
            ||||||||||||||||||||||||||||||||||||||||||||||||||||||||||||
Sbjct: 2646 ataattatattcggacaaatctacccatttgtgagccacagtctcaaacttagcttggtc 2587


Query: 666  ccaactagtattccaatgatttggtcgttttacattaccatattggatatcataggttgc 725
```

```
            ||||||||||||||||||||||||||||||||||||||||||||||||||||||||||||
Sbjct: 2586 ccaactagtattccaatgatttggtcgttttacattaccatattggatatcataggttgc 2527


Query: 726  ataggtagatcttatatcaaccgggaaggctacctttagtaattgtcgtttttcatgata 785
            ||||||||||||||||||||||||||||||||||||||||||||||||||||||||||||
Sbjct: 2526 ataggtagatcttatatcaaccgggaaggctacctttagtaattgtcgtttttcatgata 2467


Query: 786  atccacataggtttcaaaatcaattcttctgctatatgcatacaaggtcatatcttgttt 845
            ||||||||||||||||||||||||||||||||||||||||||||||||||||||||||||
Sbjct: 2466 atccacataggtttcaaaatcaattcttctgctatatgcatacaaggtcatatcttgttt 2407


Query: 846  gatcacagaattcatatacttccattggaatgagaatacagctctaagtgttcctatttc 905
            ||||||||||||||||||||||||||||||||||||||||||||||||||||||||||||
Sbjct: 2406 gatcacagaattcatatacttccattggaatgagaatacagctctaagtgttcctatttc 2347


Query: 906  ctttatctccaagtgttctaagtcacttacttctctcatcttttcctgat-nnnnnnntc 964
            ||||||||| |||||||||| |||||||||||||||||||||||||||||        ||
Sbjct: 2346 ctttatctcaaagtgttctaggtcacttacttctctcatcttttcctgatagaaaatatc 2287


Query: 965  aatatcccctgcatcattatccataaggcttatcttcaaaaacttgaaaaacatttggct 1024
            |||||||| ||||||||||||||| ||||||||||||||||||||||| ||||||| |||
Sbjct: 2286 aatatcccatgcatcattatccat-aggcttatcttcaaaaacttgaagaacattt-gct 2229


Query: 1025 cggtctcccaggtggcatttacctggtcttttattttcctttatcataaa 1074
            || ||| |||||| ||| |||||| ||| |||||||| ||||||||||||
Sbjct: 2228 cgttct-ccaggt-gcaattacct-gtcgtttatttt-ctttatcataaa 2183
```

#######

Cphy1847-2 forward sequence

Query= HR0ADKBY82ZD02_1847C2P16_1.SCF   1404   0   1404 SCF

>cphy1847

```
Query: 69  aaagaaggagataggatcATGaagtttcggtttgaagatttaagaaaggttgattacaaa 128
           ||||||||||||||||||||||||||||||||||||||||||||||||||||||||||||
Sbjct: 1   aaagaaggagataggatcatgaagtttcggtttgaagatttaagaaaggttgattacaaa 60


Query: 129 ggatatattaaaagtaattacnnnnnnntaattgcgtgggcagta 173
           |||||||||||||||||||||       |||||||||||||||||
Sbjct: 61  ggatatattaaaagtaattacaaaaaaataattgcgtgggcagta 105



Query: 187 gttatggattaggtaatgtgacagcacgtggtgtagctcattaccaagaaacatttgctg 246
           ||||||||||||||||||||||||||||||||||||||||||||||||||||||||||||
Sbjct: 119 gttatggattaggtaatgtgacagcacgtggtgtagctcattaccaagaaacatttgctg 178
```

```
Query: 247 tgaatgaaaaggtagataattggggtctaggtttcggacaagaaggaactcaaccaaaag 306
           ||||||||||||||||||||||||||||||||||||||||||||||||||||||||||||
Sbjct: 179 tgaatgaaaaggtagataattggggtctaggtttcggacaagaaggaactcaaccaaaag 238


Query: 307 gtactgtatctgtggaacaattaaaggaatatgatgcttactacatcggtgcgaatactg 366
           ||||||||||||||||||||||||||||||||||||||||||||||||||||||||||||
Sbjct: 239 gtactgtatctgtggaacaattaaaggaatatgatgcttactacatcggtgcgaatactg 298


Query: 367 acaaagtaatatatttaacttttgattgcggctatgaaaatggtaacacagctaaaatat 426
           ||||||||||||||||||||||||||||||||||||||||||||||||||||||||||||
Sbjct: 299 acaaagtaatatatttaacttttgattgcggctatgaaaatggtaacacagctaaaatat 358


Query: 427 tagatgccttnnnnnnncacaatgtttcggctactttctttgtagttgggcattttcttg 486
           ||||||||||       |||||||||||||||||||||||||||||||||||||||||||
Sbjct: 359 tagatgccttaaaaaaacacaatgtttcggctactttctttgtagttgggcattttcttg 418


Query: 487 aaactagccctgaccttgtaaaacgaatggtagcggaagggcatgcagttgggaatcata 546
           ||||||||||||||||||||||||||||||||||||||||||||||||||||||||||||
Sbjct: 419 aaactagccctgaccttgtaaaacgaatggtagcggaagggcatgcagttgggaatcata 478


Query: 547 cctaccatcactacgatatgtctaaaatctctagcttggatagttttcaaaaagaagtag 606
           ||||||||||||||||||||||||||||||||||||||||||||||||||||||||||||
Sbjct: 479 cctaccatcactacgatatgtctaaaatctctagcttggatagttttcaaaaagaagtag 538


Query: 607 atgatgtggcaaaactatttgaacaaattacaggacaaaagttgatgaagtattatcgtc 666
           ||||||||||||||||||||||||||||||||||||||||||||||||||||||||||||
Sbjct: 539 atgatgtggcaaaactatttgaacaaattacaggacaaaagttgatgaagtattatcgtc 598


Query: 667 caccacaaggtaaatacagtacagaaaatctaaaattggcgaaagagctaggatataaga 726
           ||||||||||||||||||||||||||||||||||||||||||||||||||||||||||||
Sbjct: 599 caccacaaggtaaatacagtacagaaaatctaaaattggcgaaagagctaggatataaga 658


Query: 727 cattcttttggaatctcgcttatgttgattgggatcaaaacaaacaaccaaccaaaaatt 786
           |||||||||||| ||||||||||||||||||||||||||||||||| ||||||||| || 
Sbjct: 659 cattcttttggagtctcgcttatgttgattgggatcaaaacaaacagccaaccaaagatc 718


Query: 787 aggcatatagtaatttattaaaagagaatacataatggggcagtagttccacttcttaat 846
           ||||||||||||| ||||| ||||||||||||||||||||||||||||| ||||| ||||
Sbjct: 719 aggcatatagtaagttatt-aaagagaatacataatggggcagtagttctacttcataat 777


Query: 847 acttccactacaaatggagaaaattttaaagaattactggacaaatgggaagagatgggc 906
           ||||| |||||||||||||| | |||  | |||||||||   ||||||||||||||||||
Sbjct: 778 acttctactacaaatggagagattttagatgaattactgacaaaatgggaagagatgggc 837


Query: 907 catgtttttaaacctttatca 927
            ||||||||||||| ||||||
Sbjct: 838 tatgtttttaaaccattatca 858
```

#######

Cphy1847-2 reverse sequence

Query= HR0ADKBY82ZD02_1847C2P17_1.SCF   1367   0   1367 SCF

>cphy1847

```
Query: 65  gtgtaatggatagtgatcTTAatggtgatggtgatgatgctgaacatcagcaagttctga 124
           ||||||||||||||||||||||||||||||||||||||||||||||||||||||||||||
Sbjct: 915 gtgtaatggatagtgatcttaatggtgatggtgatgatgctgaacatcagcaagttctga 856


Query: 125 taatggtttaaaaacatagcccatctcttcccattttgtcagtaattcatctaaaatctc 184
           ||||||||||||||||||||||||||||||||||||||||||||||||||||||||||||
Sbjct: 855 taatggtttaaaaacatagcccatctcttcccattttgtcagtaattcatctaaaatctc 796


Query: 185 tccatttgtagtagaagtattatgaagtagaactactgccccattatgtattctctttaa 244
           ||||||||||||||||||||||||||||||||||||||||||||||||||||||||||||
Sbjct: 795 tccatttgtagtagaagtattatgaagtagaactactgccccattatgtattctctttaa 736


Query: 245 taacttactatatgcctgatctttggttggctgtttgttttgatcccaatcaacataagc 304
           ||||||||||||||||||||||||||||||||||||||||||||||||||||||||||||
Sbjct: 735 taacttactatatgcctgatctttggttggctgtttgttttgatcccaatcaacataagc 676


Query: 305 gagactccaaaagaatgtcttatatcctagctctttcgccaattttagattttctgtact 364
           ||||||||||||||||||||||||||||||||||||||||||||||||||||||||||||
Sbjct: 675 gagactccaaaagaatgtcttatatcctagctctttcgccaattttagattttctgtact 616


Query: 365 gtatttaccttgtggtggacgataatacttcatcaacttttgtcctgtaatttgttcaaa 424
           ||||||||||||||||||||||||||||||||||||||||||||||||||||||||||||
Sbjct: 615 gtatttaccttgtggtggacgataatacttcatcaacttttgtcctgtaatttgttcaaa 556


Query: 425 tagttttgccacatcatctacttctttttgaaaactatccaagctagagattttagacat 484
           ||||||||||||||||||||||||||||||||||||||||||||||||||||||||||||
Sbjct: 555 tagttttgccacatcatctacttctttttgaaaactatccaagctagagattttagacat 496


Query: 485 atcgtagtgatggtaggtatgattcccaactgcatgcccttccgctaccattcgttttac 544
           ||||||||||||||||||||||||||||||||||||||||||||||||||||||||||||
Sbjct: 495 atcgtagtgatggtaggtatgattcccaactgcatgcccttccgctaccattcgttttac 436


Query: 545 aaggtcagggctagtttcaagaaaatgcccaactacaaagaaagtagccgaaacattgtg 604
           ||||||||||||||||||||||||||||||||||||||||||||||||||||||||||||
Sbjct: 435 aaggtcagggctagtttcaagaaaatgcccaactacaaagaaagtagccgaaacattgtg 376


Query: 605 nnnnnnnaaggcatctaatattttagctgtgttaccattttcatagccgcaatcaaaagt 664
                  |||||||||||||||||||||||||||||||||||||||||||||||||||||
Sbjct: 375 tttttttaaggcatctaatattttagctgtgttaccattttcatagccgcaatcaaaagt 316
```

```
Query: 665 taaatatattactttgtcagtattcgcaccgatgtagtaagcatcatattcctttaattg 724
           ||||||||||||||||||||||||||||||||||||||||||||||||||||||||||||
Sbjct: 315 taaatatattactttgtcagtattcgcaccgatgtagtaagcatcatattcctttaattg 256


Query: 725 ttccacagatacagtaccttttggttgagttccttcttgtccgaaacctagaccccaatt 784
           ||||||||||||||||||||||||||||||||||||||||||||||||||||||||||||
Sbjct: 255 ttccacagatacagtaccttttggttgagttccttcttgtccgaaacctagaccccaatt 196


Query: 785 atctaccttttcattcacagcaaatgtttcttggtaatgagctacaccacgtgctgtcac 844
           ||||||||||||||||||||||||||||||||||||||||||||||||||||||||||||
Sbjct: 195 atctaccttttcattcacagcaaatgtttcttggtaatgagctacaccacgtgctgtcac 136


Query: 845 attacctaatccataac 861
           |||||||||||||||||
Sbjct: 135 attacctaatccataac 119


#######

Query= HR0ADKBY82ZD06_1877C1P16_1.SCF   1402      0   1402  SCF

>cphy1877

Query: 68  aaagaaggagataggatcATGaatcaaaatacatacgattttaagacaagtccagttgct 127
           ||||||||||||||||||||||||||||||||||||||||||||||||||||||||||||
Sbjct: 1   aaagaaggagataggatcatgaatcaaaatacatacgattttaagacaagtccagttgct 60


Query: 128 aatcaaaaagctgttatacaaggagattgttatcgttttacagtactgacatctagatta 187
           ||||||||||||||||||||||||||||||||||||||||||||||||||||||||||||
Sbjct: 61  aatcaaaaagctgttatacaaggagattgttatcgttttacagtactgacatctagatta 120


Query: 188 attcgattagaatataatgaggatggttatttcgaagatttagcaactcaagtggtaata 247
           ||||||||||||||||||||||||||||||||||||||||||||||||||||||||||||
Sbjct: 121 attcgattagaatataatgaggatggttatttcgaagatttagcaactcaagtggtaata 180


Query: 248 aaccgtgagtttccagtaccagaataccgtgtaaaagaaggggaagatagcttagaagta 307
           ||||||||||||||||||||||||||||||||||||||||||||||||||||||||||||
Sbjct: 181 aaccgtgagtttccagtaccagaataccgtgtaaaagaaggggaagatagcttagaagta 240


Query: 308 ataactgagcacttacatcttacctacaataaaaagctgtttagtaaaaatggtttatgt 367
           ||||||||||||||||||||||||||||||||||||||||||||||||||||||||||||
Sbjct: 241 ataactgagcacttacatcttacctacaataaaaagctgtttagtaaaaatggtttatgt 300


Query: 368 attcaagtaaaaggaaatttaagtgcttatcgaagtatatggcattttggtgagacagga 427
           ||||||||||||||||||||||||||||||||||||||||||||||||||||||||||||
Sbjct: 301 attcaagtaaaaggaaatttaagtgcttatcgaagtatatggcattttggtgagacagga 360
```

```
Query: 428 gagaacttaaaaggaacagcaaggacgttggatgagacggatggtgaaatagcgttagaa 487
           ||||||||||||||||||||||||||||||||||||||||||||||||||||||||||||
Sbjct: 361 gagaacttaaaaggaacagcaaggacgttggatgagacggatggtgaaatagcgttagaa 420


Query: 488 gatggtttactttcacgaaatgggtttgcaatcattgatgatagtcgttccattttatta 547
           ||||||||||||||||||||||||||||||||||||||||||||||||||||||||||||
Sbjct: 421 gatggtttactttcacgaaatgggtttgcaatcattgatgatagtcgttccattttatta 480


Query: 548 aataaagatggatgggtaaaacctagagaacaaaaaggaattgacttatactttttttgga 607
           ||||||||||||||||||||||||||||||||||||||||||||||||||||||||||||
Sbjct: 481 aataaagatggatgggtaaaacctagagaacaaaaaggaattgacttatactttttttgga 540


Query: 608 tacggaagagagtacctgaaatgtctcaaccgattattatgtactatgtggtaagacccc 667
           ||||||||||||||||||||||||||||| ||||||||||||||||||||||||||||||
Sbjct: 541 tacggaagagagtacctgaaatgtctcaa-cgattattatgtactatgtggtaagacccc 599


Query: 668 catgttacctcgttatactcttggtaattggtggagccgctttctacccatattcaaaaa 727
           |||||||||||||||||||||||||||||||||||||||| ||||||||||||||| || 
Sbjct: 600 catgttacctcgttatactcttggtaattggtggagccgc-ttctacccatattcagaag 658


Query: 728 atgaatataaacagttagtttcgtaaatttgaaacaaaaaatgtaccattttcccatggc 787
           |||||||||||||||||| ||||||||||||||||| | ||||||||||||| |||||||
Sbjct: 659 atgaatataaacagttag-ttcgtaaatttgaaacagagaatgtaccatttt-ccatggc 716


Query: 788 cgttattggtatggattgggatctgactaaccttgatcccaaataccggaagtgggtgga 847
            ||||||| |||||||||| |||||||||| |||||||| ||||| ||||||||||||||
Sbjct: 717 agttattgatatggattggcatctgactaaacttgatccgaaata-cggaagtgggtgga 775


Query: 848 ccgggataaccttggaatagaaaagtactttaaagagcc 886
            | |||||  | |||||||| | ||||||||||||||||
Sbjct: 776 -caggatatacatggaatag-agagtactttaaagagcc 812


#######


Query= HR0ADKBY82ZD06_1877C1P17_1.SCF   1342      0   1342  SCF

>cphy1877

Query: 65   gtgtaatggatagtgatcttaatggtgatggtgatgatgataagccattataacttcgta 124
            ||||||||||||||||||||||||||||||||||||||||||||||||||||||||||||
Sbjct: 2448 gtgtaatggatagtgatcttaatggtgatggtgatgatgataagccattataacttcgta 2389


Query: 125  taaagcaccaagtaattcatctggtaaatgtaaagcctgaatctctgccatagcaagctc 184
            ||||||||||||||||||||||||||||||||||||||||||||||||||||||||||||
Sbjct: 2388 taaagcaccaagtaattcatctggtaaatgtaaagcctgaatctctgccatagcaagctc 2329


Query: 185  tctgctacgatgtttttaataattcgataaattaaactcttacaatcaaagtcaatctg 244
```

```
            ||||||||||||||||||||||||||||||||||||||||||||||||||||||||||||
Sbjct: 2328 tctgctacgatgtttttaataattcgataaattaaactcttacaatcaaagtcaatctg 2269

Query: 245  tgtcttatctagtaaattaaaaacttgtacaattatatcattttctataatttgatcgca 304
            ||||||||||||||||||||||||||||||||||||||||||||||||||||||||||||
Sbjct: 2268 tgtcttatctagtaaattaaaaacttgtacaattatatcattttctataatttgatcgca 2209

Query: 305  ttcaggcagcttaataacaagagccttattcacagtaattgagaatacctgaatatgcat 364
            ||||||||||||||||||||||||||||||||||||||||||||||||||||||||||||
Sbjct: 2208 ttcaggcagcttaataacaagagccttattcacagtaattgagaatacctgaatatgcat 2149

Query: 365  aatatttctttccgcatcatatcgtttcgtaaaggatatttcttttccctcaagaaatac 424
            ||||||||||||||||||||||||||||||||||||||||||||||||||||||||||||
Sbjct: 2148 aatatttctttccgcatcatatcgtttcgtaaaggatatttcttttccctcaagaaatac 2089

Query: 425  actaactaaatcactatctttgcagccaacgatgtcaatctgatagtttctttcttctgg 484
            ||||||||||||||||||||||||||||||||||||||||||||||||||||||||||||
Sbjct: 2088 actaactaaatcactatctttgcagccaacgatgtcaatctgatagtttctttcttctgg 2029

Query: 485  aatgataccattattttttacttctacattgaattttaaattcggagtctttattccaggt 544
            ||||||||||||||||||||||||||||||||||||||||||||||||||||||||||||
Sbjct: 2028 aatgataccattattttttacttctacattgaattttaaattcggagtctttattccaggt 1969

Query: 545  aaattgcatcgaagtaattacttccctaacttcactttgcactgaagtaagagagttatc 604
            ||||||||||||||||||||||||||||||||||||||||||||||||||||||||||||
Sbjct: 1968 aaattgcatcgaagtaattacttccctaacttcactttgcactgaagtaagagagttatc 1909

Query: 605  ttcatacaagtcaaatcttccgtccgcaccagcaaagaccttgattaaaagatccgatgg 664
            ||||||||||||||||||||||||||||||||||||||||||||||||||||||||||||
Sbjct: 1908 ttcatacaagtcaaatcttccgtccgcaccagcaaagaccttgattaaaagatccgatgg 1849

Query: 665  attctcggcactgttggatacggcatcctttaccaccatcggaataatagctccggcacg 724
            ||||||||||||||||||||||||||||||||||||||||||||||||||||||||||||
Sbjct: 1848 attctcggcactgttggatacggcatcctttaccaccatcggaataatagctccggcacg 1789

Query: 725  tgcaaagaccggtatcgttgaaagttcacgatagaatcgtattcggcgtcccccacgata 784
            ||||||||||||||||||||||||||||||||||||||||||||||||||||||||||||
Sbjct: 1788 tgcaaagaccggtatcgttgaaagttcacgatagaatcgtattcggcgtcccccacgata 1729

Query: 785  cactcttccggtaaagaaatcaaaatatattccttccgggagccatccatcaaatccacc 844
            ||||||||||||||||||||||||||||||||||||||||||||||||||||||||||||
Sbjct: 1728 cactcttccggtaaagaaatcaaaatatattccttccgggagccatccatcaaatccacc 1669

Query: 845  catattgagttcaaggtttaaaggttttgttatcggacatgcgattaactctgagccaaa 904
            ||||||||||||||||||||||||||||||||||||||||||||||||||||||||||||
Sbjct: 1668 catattgagttcaaggtttaaaggttttgttatcggacatgcgattaactctgagccaaa 1609
```

```
Query: 905  gtaatattgattcggaacatggtatgcttccctcgtcctccgggttttgataatacattg 964
            ||||||||||||||||||||||||||||| |||||| |||||||||||||||||||||||
Sbjct: 1608 gtaatattgattcggaacatggtatgctt-cctcgttctccgggttttgataatacattg 1550


Query: 965  gttggatcagtggtttattctcactatggaataaataattcattgtgtaaaaatacggaa 1024
            ||||||||||||||||||||||||||||||||||||||||||||||||| |||||||| |
Sbjct: 1549 gttggatcagtggtttattctcactatggaataaataattcattgtgtataaatacggta 1490


Query: 1025 ttaattgatggccgaagttgtaaaaaccca 1054
            ||||||||||| || |||||||||||||||
Sbjct: 1489 ttaattgatgg-cgtagttgtaaaaaccca 1461


#######

Query= HR0ADKBY82ZE02_2105C2P16_1.SCF   1352      0   1352  SCF

>cphy2105 foward

Query: 68  aaagaaggagataggatcATGcatcatcaccatcaccatactgattactggcagaattgg 127
           ||||||||||||||||||||||||||||||||||||||||||||||||||||||||||||
Sbjct: 1   aaagaaggagataggatcatgcatcatcaccatcaccatactgattactggcagaattgg 60


Query: 128 actgatggtggtggaacagtgaacgctaccaatggttctggtggtaactacagtgttaac 187
           ||||||||||||||||||||||||||||||||||||||||||||||||||||||||||||
Sbjct: 61  actgatggtggtggaacagtgaacgctaccaatggttctggtggtaactacagtgttaac 120


Query: 188 tggacgaattgtggtaattttgttgtaggtaaaggttggggtactggaaatgcatcaaga 247
           ||||||||||||||||||||||||||||||||||||||||||||||||||||||||||||
Sbjct: 121 tggacgaattgtggtaattttgttgtaggtaaaggttggggtactggaaatgcatcaaga 180


Query: 248 gttgtaaattacaatgctggtgtattttcaccatctggtaatggttatttaactttctat 307
           ||||||||||||||||||||||||||||||||||||||||||||||||||||||||||||
Sbjct: 181 gttgtaaattacaatgctggtgtattttcaccatctggtaatggttatttaactttctat 240


Query: 308 ggttggacgagaaattcactcattgaatattatgttgttgatagttggggtacttataga 367
           ||||||||||||||||||||||||||||||||||||||||||||||||||||||||||||
Sbjct: 241 ggttggacgagaaattcactcattgaatattatgttgttgatagttggggtacttataga 300


Query: 368 ccaactggaactttaaagggtacagtttctagcgatggtggaacatatgatatttataca 427
           ||||||||||||||||||||||||||||||||||||||||||||||||||||||||||||
Sbjct: 301 ccaactggaactttaaagggtacagtttctagcgatggtggaacatatgatatttataca 360


Query: 428 agtacgagaactaatgcaccttccatcgatggtactcagactttccaacaatactggagt 487
           ||||||||||||||||||||||||||||||||||||||||||||||||||||||||||||
Sbjct: 361 agtacgagaactaatgcaccttccatcgatggtactcagactttccaacaatactggagt 420


Query: 488 gttagacagtctaagagagctaccggaagtaacgtagcaattacttttagtaaccatgtt 547
```

```
            ||||||||||||||||||||||||||||||||||||||||||||||||||||||||||||
Sbjct: 421 gttagacagtctaagagagctaccggaagtaacgtagcaattacttttagtaaccatgtt 480


Query: 548 aatgcttggaagagtaaaggaatgaacttaggaagcagctgggcttatcaagcattatgc 607
            ||||||||||||||||||||||||||||||||||||||||||||||||||||||||||||
Sbjct: 481 aatgcttggaagagtaaaggaatgaacttaggaagcagctgggcttatcaagcattatgc 540


Query: 608 gtagaaggatatcaaagcagtggtagtgctaatgtaacggtttggtaagatcactatcca 667
            ||||||||||||||||||||||||||||||||||||||||||||||||||||||||||||
Sbjct: 541 gtagaaggatatcaaagcagtggtagtgctaatgtaacggtttggtaagatcactatcca 600


Query: 668 ttacac 673
            ||||||
Sbjct: 601 ttacac 606


#######

Query= HR0ADKBY82ZE02_2105C2P17_1.SCF   1358      0   1358  SCF

>cphy2105 reverse


Query: 66  gtgtaatggatagtgatcttaccaaaccgttacattagcactaccactgctttgatatcc 125
            ||||||||||||||||||||||||||||||||||||||||||||||||||||||||||||
Sbjct: 606 gtgtaatggatagtgatcttaccaaaccgttacattagcactaccactgctttgatatcc 547


Query: 126 ttctacgcataatgcttgataagcccagctgcttcctaagttcattcctttactcttcca 185
            ||||||||||||||||||||||||||||||||||||||||||||||||||||||||||||
Sbjct: 546 ttctacgcataatgcttgataagcccagctgcttcctaagttcattcctttactcttcca 487


Query: 186 agcattaacatggttactaaaagtaattgctacgttacttccggtagctctcttagactg 245
            ||||||||||||||||||||||||||||||||||||||||||||||||||||||||||||
Sbjct: 486 agcattaacatggttactaaaagtaattgctacgttacttccggtagctctcttagactg 427


Query: 246 tctaacactccagtattgttggaaagtctgagtaccatcgatggaaggtgcattagttct 305
            ||||||||||||||||||||||||||||||||||||||||||||||||||||||||||||
Sbjct: 426 tctaacactccagtattgttggaaagtctgagtaccatcgatggaaggtgcattagttct 367


Query: 306 cgtacttgtataaatatcatatgttccaccatcgctagaaactgtaccctttaaagttcc 365
            ||||||||||||||||||||||||||||||||||||||||||||||||||||||||||||
Sbjct: 366 cgtacttgtataaatatcatatgttccaccatcgctagaaactgtaccctttaaagttcc 307


Query: 366 agttggtctataagtaccccaactatcaacaacataatattcaatgagtgaatttctcgt 425
            ||||||||||||||||||||||||||||||||||||||||||||||||||||||||||||
Sbjct: 306 agttggtctataagtaccccaactatcaacaacataatattcaatgagtgaatttctcgt 247


Query: 426 ccaaccatagaaagttaaataaccattaccagatggtgaaaatacaccagcattgtaatt 485
            ||||||||||||||||||||||||||||||||||||||||||||||||||||||||||||
```

```
Sbjct: 246 ccaaccatagaaagttaaataaccattaccagatggtgaaaatacaccagcattgtaatt 187


Query: 486 tacaactcttgatgcatttccagtaccccaacctttacctacaacaaaattaccacaatt 545
           ||||||||||||||||||||||||||||||||||||||||||||||||||||||||||||
Sbjct: 186 tacaactcttgatgcatttccagtaccccaacctttacctacaacaaaattaccacaatt 127


Query: 546 cgtccagttaacactgtagttaccaccagaaccattggtagcgttcactgttccaccacc 605
           ||||||||||||||||||||||||||||||||||||||||||||||||||||||||||||
Sbjct: 126 cgtccagttaacactgtagttaccaccagaaccattggtagcgttcactgttccaccacc 67


Query: 606 atcagtccaattctgccagtaatcagtatggtgatggtgatgatgcatgatcctatctcc 665
           ||||||||||||||||||||||||||||||||||||||||||||||||||||||||||||
Sbjct: 66  atcagtccaattctgccagtaatcagtatggtgatggtgatgatgcatgatcctatctcc 7


Query: 666 ttcttt 671
           ||||||
Sbjct: 6   ttcttt 1

#######

Query= HR0ADKBY82ZE07_2276C2P16_1.SCF   1354      0   1354  SCF


>cphy2276 forward


Query: 69   aaagaaggagataggatcATGagtaatccaaaagatgttgttgtagacacagaagtcgta 128
            ||||||||||||||||||||||||||||||||||||||||||||||||||||||||||||
Sbjct: 1    aaagaaggagataggatcatgagtaatccaaaagatgttgttgtagacacagaagtcgta 60


Query: 129  aatacagaaacagtaaatacagatgctgcatgcacagataccccaagtacagttttaccc 188
            ||||||||||||||||||||||||||||||||||||||||||||||||||||||||||||
Sbjct: 61   aatacagaaacagtaaatacagatgctgcatgcacagataccccaagtacagttttaccc 120


Query: 189  tttactacactatcacctactatgataccgattgaacttgaagcggaagatgctgtactt 248
            ||||||||||||||||||||||||||||||||||||||||||||||||||||||||||||
Sbjct: 121  tttactacactatcacctactatgataccgattgaacttgaagcggaagatgctgtactt 180


Query: 249  agtggaaatgtaaagatagaaagtagcaaaagtggattttcaggaacaggatacattagc 308
            ||||||||||||||||||||||||||||||||||||||||||||||||||||||||||||
Sbjct: 181  agtggaaatgtaaagatagaaagtagcaaaagtggattttcaggaacaggatacattagc 240


Query: 309  ggattcgaagatgacagtgattattgtgaatttaagcttacgatagaggaagatggtttt 368
            ||||||||||||||||||||||||||||||||||||||||||||||||||||||||||||
Sbjct: 241  ggattcgaagatgacagtgattattgtgaatttaagcttacgatagaggaagatggtttt 300


Query: 369  tatgatctaaacttcattagtgcagcgcttggtggatacaaagaaaattatgttttagta 428
            ||||||||||||||||||||||||||||||||||||||||||||||||||||||||||||
Sbjct: 301  tatgatctaaacttcattagtgcagcgcttggtggatacaaagaaaattatgttttagta 360
```

```
Query: 429  gatgatgaaaatgttggtgtagcagtaaccgatcaaagcaaattctcagattcgaaatta 488
            ||||||||||||||||||||||||||||||||||||||||||||||||||||||||||||
Sbjct: 361  gatgatgaaaatgttggtgtagcagtaaccgatcaaagcaaattctcagattcgaaatta 420


Query: 489  gaaagaatatttcttacaaatggaactcataacattaagctatcaaaattctgggggtgg 548
            ||||||||||||||||||||||||||||||||||||||||||||||||||||||||||||
Sbjct: 421  gaaagaatatttcttacaaatggaactcataacattaagctatcaaaattctgggggtgg 480


Query: 549  gttgtcgtggataaattaatagtaacatcctccatagaactagatacgagtatctatgaa 608
            ||||||||||||||||||||||||||||||||||||||||||||||||||||||||||||
Sbjct: 481  gttgtcgtggataaattaatagtaacatcctccatagaactagatacgagtatctatgaa 540


Query: 609  gttcctgcaacattagtaaatcaaaacgctagtgatcgtacaaagagattgatgagttat 668
            ||||||||||||||||||||||||||||||||||||||||||||||||||||||||||||
Sbjct: 541  gttcctgcaacattagtaaatcaaaacgctagtgatcgtacaaagagattgatgagttat 600


Query: 669  ttaaccgatatctacggtaaaaagtttttatctggtcaatattgtgagaaaggaatgtat 728
            ||||||||||||||||||||||||||||||||||||||||||||||||||||||||||||
Sbjct: 601  ttaaccgatatctacggtaaaaagtttttatctggtcaatattgtgagaaaggaatgtat 660


Query: 729  ggaacagaaatttctgcaatatggaaaacaacagaagggaaatttccagcagttctaggt 788
            ||||||||||||||||||||||||||||||||||||||||||||||||||||||||||||
Sbjct: 661  ggaacagaaatttctgcaatatggaaaacaacagaagggaaatttccagcagttctaggt 720


Query: 789  cttgatcttatagattatacttcatcaagagtagaaaaaggtagcagttcgaaagctatc 848
            ||||||||||||||||||||||||||||||||||||||||||||||||||||||||||||
Sbjct: 721  cttgatcttatagattatacttcatcaagagtagaaaaaggtagcagttcgaaagctatc 780


Query: 849  caatatgccatggaatttgatgaaaaaggcggtattgttacattctgctggcactggaat 908
            ||||||||||||||||||||||||||||||||||||||||||||||||||||||||||||
Sbjct: 781  caatatgccatggaatttgatgaaaaaggcggtattgttacattctgctggcactggaat 840


Query: 909  gcaccaacgaaatatctaactggaacatggtatcgtgttttttatacggaaaaaac-aac 967
            ||||||||||||||||||||||||||||||||||||| ||||||||||||| |||| |||
Sbjct: 841  gcaccaacgaaatatctaactggaacatggtatcgtggtttttatacggaagaaacaaac 900


Query: 968  atagatttgggttagattatgaacgggcaagatgatgaaagaaatcaattgttgatgaat 1027
            |||||||||| | ||||||||||||| || ||||||||| || |||||||||||||||||
Sbjct: 901  atagatttggttaagattatgaacggtcaggatgatgaaggatatcaattgttgatgaat 960


Query: 1028 gatattgatgcctttgccaaaaaactacttaaactaaa 1065
            |||||||||||| |||| ||| ||||||||||||||||
Sbjct: 961  gatattgatgccattgcaaaagaactacttaaactaaa 998
```

######

```
Query= HR0ADKBY82ZE07_2276C2P17_1.SCF   1344      0   1344  SCF


>cphy2276 reverse


Query: 66   gtgtaatggatagtgatcttaatggtgatggtgatgatgttcataaattgggtaattctt 125
            ||||||||||||||||||||||||||||||||||||||||||||||||||||||||||||
Sbjct: 1569 gtgtaatggatagtgatcttaatggtgatggtgatgatgttcataaattgggtaattctt 1510


Query: 126  taagtctggtagtttatctagtgttataacactatcatgggcatagaccttcttaaccat 185
            ||||||||||||||||||||||||||||||||||||||||||||||||||||||||||||
Sbjct: 1509 taagtctggtagtttatctagtgttataacactatcatgggcatagaccttcttaaccat 1450


Query: 186  atactcctctgtatactgttctgatagccagttaatcaaagcggattttgtaacaaattc 245
            ||||||||||||||||||||||||||||||||||||||||||||||||||||||||||||
Sbjct: 1449 atactcctctgtatactgttctgatagccagttaatcaaagcggattttgtaacaaattc 1390


Query: 246  gccaccccatgttgcaaaaaatccccacattgcaccatcacgaattgctaaatccgggtc 305
            ||||||||||||||||||||||||||||||||||||||||||||||||||||||||||||
Sbjct: 1389 gccaccccatgttgcaaaaaatccccacattgcaccatcacgaattgctaaatccgggtc 1330


Query: 306  aaacagacaaccattttcagataatacgaccatctttttttgcttgtgtataattcatggc 365
            ||||||||||||||||||||||||||||||||||||||||||||||||||||||||||||
Sbjct: 1329 aaacagacaaccattttcagataatacgaccatctttttttgcttgtgtataattcatggc 1270


Query: 366  ctttaaatatttattaatctgagaggtataaactttttcaccaggataaatatcctcacc 425
            ||||||||||||||||||||||||||||||||||||||||||||||||||||||||||||
Sbjct: 1269 ctttaaatatttattaatctgagaggtataaactttttcaccaggataaatatcctcacc 1210


Query: 426  gataatatcaacatattcatctcccggataccattccttatcctgcccattccatagcca 485
            ||||||||||||||||||||||||||||||||||||||||||||||||||||||||||||
Sbjct: 1209 gataatatcaacatattcatctcccggataccattccttatcctgcccattccatagcca 1150


Query: 486  gattaagttatttagcccgtattcgtttgttagtttttcatataataatatgtacaattt 545
            ||||||||||||||||||||||||||||||||||||||||||||||||||||||||||||
Sbjct: 1149 gattaagttatttagcccgtattcgtttgttagtttttcatataataatatgtacaattt 1090


Query: 546  cttataggcttctgcacctttggctccccaccaaaaccacccaccgcttgcttcatgtaa 605
            ||||||||||||||||||||||||||||||||||||||||||||||||||||||||||||
Sbjct: 1089 cttataggcttctgcacctttggctccccaccaaaaccacccaccgcttgcttcatgtaa 1030


Query: 606  cggtctccataaaataggcacatctgcttcttttagtttaagtagttcttttgcaatggc 665
            ||||||||||||||||||||||||||||||||||||||||||||||||||||||||||||
Sbjct: 1029 cggtctccataaaataggcacatctgcttcttttagtttaagtagttcttttgcaatggc 970


Query: 666  atcaatatcattcatcaacaattgatatccttcatcatcctgaccgttcataatcttaac 725
```

```
            ||||||||||||||||||||||||||||||||||||||||||||||||||||||||||||
Sbjct: 969  atcaatatcattcatcaacaattgatatccttcatcatcctgaccgttcataatcttaac 910


Query: 726  caaatctatgtttgtttcttccgtataaaaaccacgataccatgttccagttagatattt 785
            ||||||||||||||||||||||||||||||||||||||||||||||||||||||||||||
Sbjct: 909  caaatctatgtttgtttcttccgtataaaaaccacgataccatgttccagttagatattt 850


Query: 786  cgttggtgcattccagtgccagcagaatgtaacaataccgccttttttcatcaaattccat 845
            ||||||||||||||||||||||||||||||||||||||||||||||||||||||||||||
Sbjct: 849  cgttggtgcattccagtgccagcagaatgtaacaataccgccttttttcatcaaattccat 790


Query: 846  ggcatattggatagctttcgaactgctaccttttttctactcttgatgaagtataatctat 905
            ||||||||||||||||||||||||||||||||||||||||||||||||||||||||||||
Sbjct: 789  ggcatattggatagctttcgaactgctaccttttttctactcttgatgaagtataatctat 730


Query: 906  aagatcaagaactagaactgctggaaatttcccttctngtgttttccatattgcagaatt 965
            |||||||||| ||||||||||||||||||||||||||  |||||||||||||||||||
Sbjct: 729  aagatcaagacctagaactgctggaaatttcccttctgttgttttccatattgcagaa-a 671


Query: 966  tttctgttcataactttcttttctccacatatttgaccgaataaaacttttaccgtaa 1025
            |||||||||   || ||| ||||| ||||   ||||||  ||||||||||||||||||
Sbjct: 670  tttctgttccatacattcctttct-cacaatattgaccagataaaacttttaccgtag 612


Query: 1026 atatcgggtaaaataactcatcaatctctttgtacgatccactaccgttttgattttact 1085
            ||||| ||| |||||||||||||||||||||||||||| ||||| |||||||| ||||||
Sbjct: 611  atatc-ggttaaataactcatcaatctctttgtacgat-cactagcgttttga-tttact 555


Query: 1086 -atgttgcaggaaacttcataaaatactcgtatcaagtt 1123
             |||||||||| |||||||| | ||||||||||| ||||
Sbjct: 554  aatgttgcagg-aacttcat-agatactcgtatctagtt 518


#######

Query= HR0ADKBY82ZF02_2848C2P16_1.SCF   1340      0   1340  SCF


>Cphy2848 forward


Query: 68   aaagaaggagataggatcATGacaagtaggaggagacttcttagttggaactataaagta 127
            ||||||||||||||||||||||||||||||||||||||||||||||||||||||||||||
Sbjct: 1    aaagaaggagataggatcatgacaagtaggaggagacttcttagttggaactataaagta 60


Query: 128  tttacagagaagggagctagtcagatgaagtatcagagtaatatggtatcagatttacaa 187
            ||||||||||||||||||||||||||||||||||||||||||||||||||||||||||||
Sbjct: 61   tttacagagaagggagctagtcagatgaagtatcagagtaatatggtatcagatttacaa 120


Query: 188  atagcttatattggtggaggttcccgaggatgggcatggactttcatgactgacctagca 247
```

```
               ||||||||||||||||||||||||||||||||||||||||||||||||||||||||||||
Sbjct: 121     atagcttatattggtggaggttcccgaggatgggcatggactttcatgactgacctagca 180

Query: 248     agagagccaaaattatctggtactgttaggctgtttgatattgataaatccgcagcagaa 307
               ||||||||||||||||||||||||||||||||||||||||||||||||||||||||||||
Sbjct: 181     agagagccaaaattatctggtactgttaggctgtttgatattgataaatccgcagcagaa 240

Query: 308     caaaatatgtttattggaaattcaatcacacaaagagaagatgccattggaaaatggaac 367
               ||||||||||||||||||||||||||||||||||||||||||||||||||||||||||||
Sbjct: 241     caaaatatgtttattggaaattcaatcacacaaagagaagatgccattggaaaatggaac 300

Query: 368     tatgaaacaaaagaaacattagaggaggctctaaccggtgcagattttattgtgatatcg 427
               ||||||||||||||||||||||||||||||||||||||||||||||||||||||||||||
Sbjct: 301     tatgaaacaaaagaaacattagaggaggctctaaccggtgcagattttattgtgatatcg 360

Query: 428     atattgcctgggacttttgatgaaatggaatctgatgtacataccccagaacgtttggga 487
               ||||||||||||||||||||||||||||||||||||||||||||||||||||||||||||
Sbjct: 361     atattgcctgggacttttgatgaaatggaatctgatgtacataccccagaacgtttggga 420

Query: 488     atttatcaatctgttggagatactgcaggtcctggtggcataatacgtgcacttcgcacc 547
               ||||||||||||||||||||||||||||||||||||||||||||||||||||||||||||
Sbjct: 421     atttatcaatctgttggagatactgcaggtcctggtggcataatacgtgcacttcgcacc 480

Query: 548     attcctatgtttgttgatatagcagaagcagtnnnnnnntatgctcctaaagcatgggta 607
               ||||||||||||||||||||||||||||||||       |||||||||||||||||||||
Sbjct: 481     attcctatgtttgttgatatagcagaagcagtaaaaaaatatgctcctaaagcatgggta 540

Query: 608     attaattatacgaatccaatgactttatgtgtgaaaacattatatcatgtatttccagag 667
               ||||||||||||||||||||||||||||||||||||||||||||||||||||||||||||
Sbjct: 541     attaattatacgaatccaatgactttatgtgtgaaaacattatatcatgtatttccagag 600

Query: 668     attaaagcatttggttgttgtcatgaagtatttggaactcagaaagttttaaaagggatc 727
               ||||||||||||||||||||||||||||||||||||||||||||||||||||||||||||
Sbjct: 601     attaaagcatttggttgttgtcatgaagtatttggaactcagaaagttttaaaagggatc 660

Query: 728     gcagaacaggtgcttggtattgaggatataccaagaaatgaagttcatgttaatgtctta 787
               ||||||||||||||||||||||||||||||||||||||||||||||||||||||||||||
Sbjct: 661     gcagaacaggtgcttggtattgaggatataccaagaaatgaagttcatgttaatgtctta 720

Query: 788     gggattaatcactttacttggtttgattatgcatcctatcaaggaatagatttattccct 847
               ||||||||||||||||||||||||||||||||||||||||||||||||||||||||||||
Sbjct: 721     gggattaatcactttacttggtttgattatgcatcctatcaaggaatagatttattccct 780

Query: 848     atctatagagattatgtaaaggaacattttgaggaaggttttatagaaaatgatgcaaac 907
               ||||||||||||||||||||||||||||||||||||||||||||||||||||||||||||
Sbjct: 781     atctatagagattatgtaaaggaacattttgaggaaggttttatagaaaatgatgcaaac 840
```

```
Query: 908  tggggcaatactacatttgcatgttctcatcctgtaaaatttgaattattccagaaatat 967
            ||||  ||||||||||||||||||||||||| |||||||||||| |||||||||||||||
Sbjct: 841  tgggcaaatactacatttgcatgttctcatcgtgtaaaatttgatttattccagaaatat 900


Query: 968  ggattgattgcagctgcaggaaatcctcacttggaaaagtttgtaccctggcgattggtc 1027
            ||||||||||||||||||||| ||| |||||||| | |||||||| |||||||||||||
Sbjct: 901  ggattgattgcagctgcaggagatcgtcacttggcagagtttgta-cctggcgattggta 959


Query: 1028 ctttaaaaatcccggaaacgtaaagaactggaagttttgattaact 1073
            ||| ||| |||| | ||||||||||| |||||||||| ||||||||
Sbjct: 960  cttgaaagatccggaaaacgtaaagagctggaagtttggattaact 1005

#######

Query= HR0ADKBY82ZF02_2848C2P17_1.SCF   1386      0   1386  SCF

>Cphy2848 reverse

Query: 66   gtgtaatggatagtgatcttaatggtgatggtgatgatgtttccagccttccggcaagta 125
            ||||||||||||||||||||||||||||||||||||||||||||||||||||||||||||
Sbjct: 1518 gtgtaatggatagtgatcttaatggtgatggtgatgatgtttccagccttccggcaagta 1459


Query: 126  ttttatagatccctgaatcatgtcctctaccagtaaatcaatttcctccgcagtagctct 185
            ||||||||||||||||||||||||||||||||||||||||||||||||||||||||||||
Sbjct: 1458 ttttatagatccctgaatcatgtcctctaccagtaaatcaatttcctccgcagtagctct 1399


Query: 186  tcctttcactaatggatcattggcgaatgcctgctttactaactctttatcacatgtaag 245
            ||||||||||||||||||||||||||||||||||||||||||||||||||||||||||||
Sbjct: 1398 tcctttcactaatggatcattggcgaatgcctgctttactaactctttatcacatgtaag 1339


Query: 246  tgcagcctttaatactagttcatggttttgtatatgtggtatggttaaatgtaagataga 305
            ||||||||||||||||||||||||||||||||||||||||||||||||||||||||||||
Sbjct: 1338 tgcagcctttaatactagttcatggttttgtatatgtggtatggttaaatgtaagataga 1279


Query: 306  gtctggcatctcccctgcaataattggacgtatggcatccctctcaaaaatagcatttgt 365
            ||||||||||||||||||||||||||||||||||||||||||||||||||||||||||||
Sbjct: 1278 gtctggcatctcccctgcaataattggacgtatggcatccctctcaaaaatagcatttgt 1219


Query: 366  ctcaaccaccactgaatctggtatattcggtatttgcctgttggtatttggaatatttac 425
            ||||||||||||||||||||||||||||||||||||||||||||||||||||||||||||
Sbjct: 1218 ctcaaccaccactgaatctggtatattcggtatttgcctgttggtatttggaatatttac 1159


Query: 426  attacttacaactctttctaaaccacagagcgcttttattaataaaattccttcttctcc 485
            ||||||||||||||||||||||||||||||||||||||||||||||||||||||||||||
Sbjct: 1158 attacttacaactctttctaaaccacagagcgcttttattaataaaattccttcttctcc 1099


Query: 486  agatgcctttaaatcaactttctcttctccactcactaaacgatggcttttctcaagtct 545
            ||||||||||||||||||||||||||||||||||||||||||||||||||||||||||||
```

```
Sbjct: 1098 agatgcctttaaatcaactttctcttctccactcactaaacgatggcttttctcaagtct 1039


Query: 546  ctgtttaaggtcttcctttctccaatctactgtagttaatccaaacttccagctctttac 605
            ||||||||||||||||||||||||||||||||||||||||||||||||||||||||||||
Sbjct: 1038 ctgtttaaggtcttcctttctccaatctactgtagttaatccaaacttccagctctttac 979


Query: 606  gttttccggatctttcaagtaccaatcgccaggtacaaactctgccaagtgacgatctcc 665
            ||||||||||||||||||||||||||||||||||||||||||||||||||||||||||||
Sbjct: 978  gttttccggatctttcaagtaccaatcgccaggtacaaactctgccaagtgacgatctcc 919


Query: 666  tgcagctgcaatcaatccatatttctggaataaatcaaattttacacgatgagaacatgc 725
            ||||||||||||||||||||||||||||||||||||||||||||||||||||||||||||
Sbjct: 918  tgcagctgcaatcaatccatatttctggaataaatcaaattttacacgatgagaacatgc 859


Query: 726  aaatgtagtatttgcccagtttgcatcattttctataaaccttcctcaaaatgttcctt 785
            ||||||||||||||||||||||||||||||||||||||||||||||||||||||||||||
Sbjct: 858  aaatgtagtatttgcccagtttgcatcattttctataaaccttcctcaaaatgttcctt 799


Query: 786  tacataatctctatagatagggaataaatctattccttgataggatgcataatcaaacca 845
            ||||||||||||||||||||||||||||||||||||||||||||||||||||||||||||
Sbjct: 798  tacataatctctatagatagggaataaatctattccttgataggatgcataatcaaacca 739


Query: 846  agtaaagtgattaatccctaaaacattaacatgaacttcatttcttggtatatcctcaat 905
            ||||||||||||||||||||| ||||||||||||||||||||||||||||||||||||||
Sbjct: 738  agtaaagtgattaatccctaagacattaacatgaacttcatttcttggtatatcctcaat 679


Query: 906  accaagcacctgttctgcgatccctttaaaactttctgagttccaaatact-catgaca 964
            |||||||||||||||||||||||||||||||||||||||||||||||||||| |||||||
Sbjct: 678  accaagcacctgttctgcgatccctttaaaactttctgagttccaaatacttcatgaca 619


Query: 965  acaaccaaatggctttaatccccggaaaaacatgaaaataatgttttcccacataaaagt 1024
            ||||||||||| |||||||| | ||||| |||||| | |||||||||| |||||||| ||
Sbjct: 618  acaaccaaatg-ctttaatctctggaaatacatgata-taatgttttcacacataaa-gt 562


Query: 1025 ccttggaattccaattaattaattacccatgcttt 1059
            | ||||| |||  || |||||||||||||||||||
Sbjct: 561  cattgga-ttcgtat-aattaattacccatgcttt 529


#######

Query= HR0ADKBY82ZF06_3010C1P16_1.SCF   1352      0   1352  SCF

>cphy3010 forward


Query: 68   aaagaaggagataggatcATGgaaaccttaaaggaaaaatataaagattatttccttatt 127
            ||||||||||||||||||||||||||||||||||||||||||||||||||||||||||||
Sbjct: 1    aaagaaggagataggatcatggaaaccttaaaggaaaaatataaagattatttccttatt 60
```

```
Query: 128  ggggcagcagtgaacgcttcaacgataaggacacatggagagttaataaaggatcatttt 187
            ||||||||||||||||||||||||||||||||||||||||||||||||||||||||||||
Sbjct: 61   ggggcagcagtgaacgcttcaacgataaggacacatggagagttaataaaggatcatttt 120


Query: 188  aactctataacatgtgaaaatgagatgaagttttctaatctttgtcccagagagaaggaa 247
            ||||||||||||||||||||||||||||||||||||||||||||||||||||||||||||
Sbjct: 121  aactctataacatgtgaaaatgagatgaagttttctaatctttgtcccagagagaaggaa 180


Query: 248  tatcagttcgaaaaagcagatacgattgtgcagtttgcaaaagataatgaactgaaaatg 307
            ||||||||||||||||||||||||||||||||||||||||||||||||||||||||||||
Sbjct: 181  tatcagttcgaaaaagcagatacgattgtgcagtttgcaaaagataatgaactgaaaatg 240


Query: 308  agaatgcataccttagtttggcacaaccaaacgcctgaatgggtatttttagatactacg 367
            ||||||||||||||||||||||||||||||||||||||||||||||||||||||||||||
Sbjct: 241  agaatgcataccttagtttggcacaaccaaacgcctgaatgggtatttttagatactacg 300


Query: 368  aaggaggggttagaggaaaggctatcaaatcatatccatatgctaggtaagcggtatgga 427
            ||||||||||||||||||||||||||||||||||||||||||||||||||||||||||||
Sbjct: 301  aaggaggggttagaggaaaggctatcaaatcatatccatatgctaggtaagcggtatgga 360


Query: 428  aaggattgttatagtgtagatgtagtaaacgaagcgatagaggataaattagatggattt 487
            ||||||||||||||||||||||||||||||||||||||||||||||||||||||||||||
Sbjct: 361  aaggattgttatagtgtagatgtagtaaacgaagcgatagaggataaattagatggattt 420


Query: 488  ttaagagaatccagttggcttcagggattagggaatgattatatagaaagggcatttctc 547
            ||||||||||||||||||||||||||||||||||||||||||||||||||||||||||||
Sbjct: 421  ttaagagaatccagttggcttcagggattagggaatgattatatagaaagggcatttctc 480


Query: 548  cttgcgaagcaagagatgccagaggctaaactgttttataacgattataatgaaaccaat 607
            ||||||||||||||||||||||||||||||||||||||||||||||||||||||||||||
Sbjct: 481  cttgcgaagcaagagatgccagaggctaaactgttttataacgattataatgaaaccaat 540


Query: 608  ccaatcaagcgggataaaatctttaaactaatcaaagaacttaaagagaaagggattcca 667
            ||||||||||||||||||||||||||||||||||||||||||||||||||||||||||||
Sbjct: 541  ccaatcaagcgggataaaatctttaaactaatcaaagaacttaaagagaaagggattcca 600


Query: 668  gtagacggtattgggatgcaatgccatatcaatatctatcattcttccatggatgaatta 727
            ||||||||||||||||||||||||||||||||||||||||||||||||||||||||||||
Sbjct: 601  gtagacggtattgggatgcaatgccatatcaatatctatcattcttccatggatgaatta 660


Query: 728  aaggaagcaattgnaaaatatttatcgcttggagtatcgattgagatcacgganatggat 787
            ||||||||||||| ||||||||||||||||||||||||||||||||||||||| ||||||
Sbjct: 661  aaggaagcaattgaaaaatatttatcgcttggagtatcgattgagatcacggaaatggat 720


Query: 788  gtatcaatgtttcgccatgaagacaggtcagaaatcttgaaaccaacgatagaacagtat 847
```

```
           ||||||||||||||||||||||||||||||||||||||||||||||||||||||||||||
Sbjct: 721  gtatcaatgtttcgccatgaagacaggtcagaaatcttgaaaccaacgatagaacagtat 780


Query: 848  aaagcacaggccagagtttacaaagagtgctttcagatatttagagaatataaaaatgat 907
            ||||||||||||||||||||||||||||||||||||||||||||||||||||||||||||
Sbjct: 781  aaagcacaggccagagtttacaaagagtgctttcagatatttagagaatataaaaatgat 840


Query: 908  attacatccgtgaccctatgggggagtatcagatgaagtcagctggctaaaccattttcc 967
            ||||||||||||||||||| |||||||||||||||| |||||||||||||||||||||||
Sbjct: 841  attacatccgtgaccctat-ggggagtatcagatgatgtcagctggctaaaccattttcc 899


Query: 968  agtaaataacccgtaaaacctggccattactgtttgaagaaatgaagaaaccaaaagaac 1027
            ||||||||| ||||| || |||||||||||||||||| || |||| ||||||||||||| 
Sbjct: 900  agtaaataa-ccgtagaaactggccattactgtttgatgagatgatgaaaccaaaagaag 958


Query: 1028 aattttatgaaattatgaatttt 1050
             ||||||||||||||||||||||
Sbjct: 959  cattttatgaaattatgaatttt 981

#######

Query= HR0ADKBY82ZF06_3010C1P17_1.SCF   1384      0   1384  SCF

>cphy3010 reverse


Query: 66   gtgtaatggatagtgatcttaatggtgatggtgatgatgaaaattcataatttcataaaa 125
            ||||||||||||||||||||||||||||||||||||||||||||||||||||||||||||
Sbjct: 1020 gtgtaatggatagtgatcttaatggtgatggtgatgatgaaaattcataatttcataaaa 961


Query: 126  tgcttcttttggtttcatcatctcatcaaacagtaatggccagtttctacggttatttac 185
            ||||||||||||||||||||||||||||||||||||||||||||||||||||||||||||
Sbjct: 960  tgcttcttttggtttcatcatctcatcaaacagtaatggccagtttctacggttatttac 901


Query: 186  tggaaaatggtttagccagctgacatcatctgatactccccatagggtcacggatgtaat 245
            ||||||||||||||||||||||||||||||||||||||||||||||||||||||||||||
Sbjct: 900  tggaaaatggtttagccagctgacatcatctgatactccccatagggtcacggatgtaat 841


Query: 246  atcattttatattctctaaatatctgaaagcactctttgtaaactctggcctgtgcttt 305
            ||||||||||||||||||||||||||||||||||||||||||||||||||||||||||||
Sbjct: 840  atcattttatattctctaaatatctgaaagcactctttgtaaactctggcctgtgcttt 781


Query: 306  atactgttctatcgttggtttcaagatttctgacctgtcttcatggcgaaacattgatac 365
            ||||||||||||||||||||||||||||||||||||||||||||||||||||||||||||
Sbjct: 780  atactgttctatcgttggtttcaagatttctgacctgtcttcatggcgaaacattgatac 721


Query: 366  atccatttccgtgatctcaatcgatactccaagcgataaatatttttcaattgcttcctt 425
            ||||||||||||||||||||||||||||||||||||||||||||||||||||||||||||
Sbjct: 720  atccatttccgtgatctcaatcgatactccaagcgataaatatttttcaattgcttcctt 661
```

```
Query: 426  taattcatccatggaagaatgatagatattgatatggcattgcatcccaataccgtctac 485
            ||||||||||||||||||||||||||||||||||||||||||||||||||||||||||||
Sbjct: 660  taattcatccatggaagaatgatagatattgatatggcattgcatcccaataccgtctac 601


Query: 486  tggaatccctttctctttaagttctttgattagtttaaagattttatcccgcttgattgg 545
            ||||||||||||||||||||||||||||||||||||||||||||||||||||||||||||
Sbjct: 600  tggaatccctttctctttaagttctttgattagtttaaagattttatcccgcttgattgg 541


Query: 546  attggtttcattataatcgttataaaacagtttagcctctggcatctcttgcttcgcaag 605
            ||||||||||||||||||||||||||||||||||||||||||||||||||||||||||||
Sbjct: 540  attggtttcattataatcgttataaaacagtttagcctctggcatctcttgcttcgcaag 481


Query: 606  gagaaatgccctttctatataatcattccctaatccctgaagccaactggattctcttaa 665
            ||||||||||||||||||||||||||||||||||||||||||||||||||||||||||||
Sbjct: 480  gagaaatgccctttctatataatcattccctaatccctgaagccaactggattctcttaa 421


Query: 666  aaatccatctaatttatcctctatcgcttcgtttactacatctacactataacaatcctt 725
            ||||||||||||||||||||||||||||||||||||||||||||||||||||||||||||
Sbjct: 420  aaatccatctaatttatcctctatcgcttcgtttactacatctacactataacaatcctt 361


Query: 726  tccataccgcttacctagcatatggatatgatttgatagcctttcctctaacccctcctt 785
            ||||||||||||||||||||||||||||||||||||||||||||||||||||||||||||
Sbjct: 360  tccataccgcttacctagcatatggatatgatttgatagcctttcctctaacccctcctt 301


Query: 786  cgtagtatctaaaaatacccattcaggcgtttggttgtgccaaactaaagtatgcattct 845
            |||||||||||||||||||||||||||||||||||||||||||||||| |||||||||||
Sbjct: 300  cgtagtatctaaaaatacccattcaggcgtttggttgtgccaaactaaggtatgcattct 241


Query: 846  cattttcagttcattatcttttgcaaactgcacaatcgtatctgctttttcgaactgata 905
            ||||||||||||||||||||||||||||||||||||||||||||||||||||||||||||
Sbjct: 240  cattttcagttcattatcttttgcaaactgcacaatcgtatctgctttttcgaactgata 181


Query: 906  ttccttctctctgggacaaagatta-aaaaattcctctca-tttcacatgttaaagaatt 963
            ||||||||||||||||||||||||| |||| ||| ||||| |||||||||||| ||| ||
Sbjct: 180  ttccttctctctgggacaaagattagaaaacttcatctcattttcacatgttatagagtt 121


Query: 964  aaaatgatcccttatttactccccctgtgtccttatcgttgaaacctttccctgctgccc 1023
            |||||||||| ||||| |||| || |||||||||||||||| || | ||| |||||||||
Sbjct: 120  aaaatgatcctttattaactctccatgtgtccttatcgttg-aagcgttcactgctgccc 62


Query: 1024 caataagg 1031
            ||||||||
Sbjct: 61   caataagg 54

#######
```

```
Query= HR0ADKBY82ZF10_2331C1P16_1.SCF   1358      0   1358  SCF

> cphy2331 forward

Query: 67   aaagaaggagataggatcATGgatgaattttggaattcaatagatggtgaaaaacaatat 126
            ||||||||||||||||||||||||||||||||||||||||||||||||||||||||||||
Sbjct: 1    aaagaaggagataggatcatggatgaattttggaattcaatagatggtgaaaaacaatat 60


Query: 127  tattatgatggtaatgatttgggatgcacttacactaaccgtagtacgaaattaaaggtt 186
            ||||||||||||||||||||||||||||||||||||||||||||||||||||||||||||
Sbjct: 61   tattatgatggtaatgatttgggatgcacttacactaaccgtagtacgaaattaaaggtt 120


Query: 187  tgggcgccaacagcttcgatggttgttgtgaatctttatcaaaatggaaatgctggaaaa 246
            ||||||||||||||||||||||||||||||||||||||||||||||||||||||||||||
Sbjct: 121  tgggcgccaacagcttcgatggttgttgtgaatctttatcaaaatggaaatgctggaaaa 180


Query: 247  ccttatatcacagaaataatgaaaaaagaggaaagtggtatctggtccgtttgtctttta 306
            ||||||||||||||||||||||||||||||||||||||||||||||||||||||||||||
Sbjct: 181  ccttatatcacagaaataatgaaaaaagaggaaagtggtatctggtccgtttgtctttta 240


Query: 307  ggtgatttggaaggagtatattatacttatcttgttacagtagatggtcaaacgaaagaa 366
            ||||||||||||||||||||||||||||||||||||||||||||||||||||||||||||
Sbjct: 241  ggtgatttggaaggagtatattatacttatcttgttacagtagatggtcaaacgaaagaa 300


Query: 367  gcagtagacccatatgcaaggacgactggtttaaatggaaaaagagcgatgattttagat 426
            ||||||||||||||||||||||||||||||||||||||||||||||||||||||||||||
Sbjct: 301  gcagtagacccatatgcaaggacgactggtttaaatggaaaaagagcgatgattttagat 360


Query: 427  ttagagaaaaccaatccaactggatttcttgaagatacaaaaccaaagtttgattccttt 486
            ||||||||||||||||||||||||||||||||||||||||||||||||||||||||||||
Sbjct: 361  ttagagaaaaccaatccaactggatttcttgaagatacaaaaccaaagtttgattccttt 420


Query: 487  ctggatgctgttatttatgaattgcatatccgcgatttatccatggaatctgactctggt 546
            ||||||||||||||||||||||||||||||||||||||||||||||||||||||||||||
Sbjct: 421  ctggatgctgttatttatgaattgcatatccgcgatttatccatggaatctgactctggt 480


Query: 547  atcaaggaaaaagggaaacttcttggacttacagagcttaatactagaaattccgatggt 606
            ||||||||||||||||||||||||||||||||||||||||||||||||||||||||||||
Sbjct: 481  atcaaggaaaaagggaaacttcttggacttacagagcttaatactagaaattccgatggt 540


Query: 607  ctaacaacaggattatcccatattctagaccttggagtaacacatattcacctactgcca 666
            ||||||||||||||||||||||||||||||||||||||||||||||||||||||||||||
Sbjct: 541  ctaacaacaggattatcccatattctagaccttggagtaacacatattcacctactgcca 600


Query: 667  tgctttgattatgcatctgtagatgaagaaaacagctctatttttaattggggatatgat 726
            ||||||||||||||||||||||||||||||||||||||||||||||||||||||||||||
Sbjct: 601  tgctttgattatgcatctgtagatgaagaaaacagctctatttttaattggggatatgat 660
```

```
Query: 727  ccagaaaattataacgttgtagaaggttcttattcaacaaatccttatgatggagctgta 786
            ||||||||||||||||||||||||||||||||||||||||||||||||||||||||||||
Sbjct: 661  ccagaaaattataacgttgtagaaggttcttattcaacaaatccttatgatggagctgta 720


Query: 787  agagtgaaagaatttaagacattagtccaatcgcttcatgagaatggacttcgtgtcatt 846
            ||||||||||||||||||||||||||||||||||||||||||||||||||||||||||||
Sbjct: 721  agagtgaaagaatttaagacattagtccaatcgcttcatgagaatggacttcgtgtcatt 780


Query: 847  atggatgttgtctatacccatacaatgaaaacagaaaaatccaatttcaataaaattgtt 906
            |||||||||||||||| ||||||||||||||||||| |||||||||||||||||||||||
Sbjct: 781  atggatgttgtctataaccatacaatgaaaacagaagaatccaatttcaataaaattgtt 840


Query: 907  ccagattactattatcgtaaaagttgaaaataaattttcagatgcttctgcttgccggaa 966
            |||||||||||||||||| ||||||| | ||||||||||||||||||||||||| ||| |
Sbjct: 841  ccagattactattatcgt-aaagttggagataaattttcagatgcttctgcttg-cggga 898


Query: 967  aggaaacagcttccaagcgtttgaaggtcccaaaagtttatttgagattctattatcta 1025
            | |||||||||||| ||||||||| ||| || ||||||||||  |||||||||||||||
Sbjct: 899  atgaaacagcttccgagcgtttgatggt-ccgaaagtttattgtagattctattatcta 956
```

#######

Query= HR0ADKBY82ZF10_2331C1P17_1.SCF   1355   0   1355 SCF

>cphy2331 reverse

```
Query: 65   gtgtaatggatagtgatcttaatggtgatggtgatgatgatctttcacaagaaccatgca 124
            ||||||||||||||||||||||||||||||||||||||||||||||||||||||||||||
Sbjct: 1998 gtgtaatggatagtgatcttaatggtgatggtgatgatgatctttcacaagaaccatgca 1939


Query: 125  agagattggttcaacttctaccgttgttccttttacttcatagagaggctccacacccga 184
            ||||||||||||||||||||||||||||||||||||||||||||||||||||||||||||
Sbjct: 1938 agagattggttcaacttctaccgttgttccttttacttcatagagaggctccacacccga 1879


Query: 185  attatttccatcaatatagacggtccaatcactctcaggaagtcttatcgttacttttc 244
            |||||||||||||||||||||||||||||||||||||||||||||||||||||||||||
Sbjct: 1878 attatttccatcaatatagacggtccaatcactctcaggaagtcttatcgttacttttc 1819


Query: 245  ctcagaagaattataaataacacatagcgtcttatctactaaatcaccttgaataagata 304
            ||||||||||||||||||||||||||||||||||||||||||||||||||||||||||||
Sbjct: 1818 ctcagaagaattataaataacacatagcgtcttatctactaaatcaccttgaataagata 1759


Query: 305  ggatatgacgtcctctctttcctctggtaagaaagttaaccgctttgatatctcttttgc 364
            ||||||||||||||||||||||||||||||||||||||||||||||||||||||||||||
Sbjct: 1758 ggatatgacgtcctctctttcctctggtaagaaagttaaccgctttgatatctcttttgc 1699
```

```
Query: 365  agattgcatgcgtagtgctttatgctctttacgaaaccttataagaccttcatagtaaga 424
            ||||||||||||||||||||||||||||||||||||||||||||||||||||||||||||
Sbjct: 1698 agattgcatgcgtagtgctttatgctctttacgaaaccttataagaccttcatagtaaga 1639


Query: 425  tacgacatcgattacattcgctttattagaccacttaatacaattgacactatcggaaga 484
            ||||||||||||||||||||||||||||||||||||||||||||||||||||||||||||
Sbjct: 1638 tacgacatcgattacattcgctttattagaccacttaatacaattgacactatcggaaga 1579


Query: 485  attgtagctattttcaacaaactctcctgctatctcgctagatggtttgtttcgtaacat 544
            ||||||||||||||||||||||||||||||||||||||||||||||||||||||||||||
Sbjct: 1578 attgtagctattttcaacaaactctcctgctatctcgctagatggtttgtttcgtaacat 1519


Query: 545  ctcctctccagcttgtaaaaatggtattccttgtgaagtaaacacaatagctgcacataa 604
            ||||||||||||||||||||||||||||||||||||||||||||||||||||||||||||
Sbjct: 1518 ctcctctccagcttgtaaaaatggtattccttgtgaagtaaacacaatagctgcacataa 1459


Query: 605  tttattctttttcactctaatttcataagtatcttccttacaagagcaagctattttatc 664
            ||||||||||||||||||||||||||||||||||||||||||||||||||||||||||||
Sbjct: 1458 tttattctttttcactctaatttcataagtatcttccttacaagagcaagctattttatc 1399


Query: 665  ccataacgtgtagttatcatgagcggatacataattaatgcactgtgctggttccaaagc 724
            ||||||||||||||||||||||||||||||||||||||||||||||||||||||||||||
Sbjct: 1398 ccataacgtgtagttatcatgagcggatacataattaatgcactgtgctggttccaaagc 1339


Query: 725  ccaaggagaatcactataattcacctttttatagtctatctgcggatgcaaggttgaagc 784
            ||||||||||||||||||||||||||||||||||||||||||||||||||||||||||||
Sbjct: 1338 ccaaggagaatcactataattcacctttttatagtctatctgcggatgcaaggttgaagc 1279


Query: 785  aactacaccaaactttacactttcctttttatcgctatcaccagttgcaaatcctttctc 844
            |||||||||||||||||||||||| |||||||||||||||||||||||||||||||||||
Sbjct: 1278 aactacaccaaactttacactttctttttttatcgctatcaccagttgcaaatcctttctc 1219


Query: 845  ctctgctagaaagacacttccctttagaccatctctaaaatcatcactgaatgccgcaat 904
            ||||||||||||||||||||||||||||||||||||||||||||||||||||||||||||
Sbjct: 1218 ctctgctagaaagacacttccctttagaccatctctaaaatcatcactgaatgccgcaat 1159


Query: 905  tccagggaacatactcatatgggctttcattgctcggttgccccgctggaaaagggctat 964
            |||||| ||||||||||||| |||||||||||||| |||| |||||||||| ||||||||
Sbjct: 1158 tccaggtaacatactcatattggctttcattgctc-gttgtcccgctggaagagggctat 1100


Query: 965  ctccccctaccccaccttcccc 986
            |||| ||||||| |||||||||
Sbjct: 1099 ctccacctacccaaccttcccc 1078


#######

Query= HR0ADKBY82ZG01_3310C1P16_1.SCF   1376      0   1376  SCF
```

```
>cphy3310 forward

Query: 68   aaagaaggagataggatcATGagccaaaagtttcgtaagttggaaggtatcgcaattgta 127
            ||||||||||||||||||||||||||||||||||||||||||||||||||||||||||||
Sbjct: 1    aaagaaggagataggatcatgagccaaaagtttcgtaagttggaaggtatcgcaattgta 60


Query: 128  gtgttattcgtatgtctgataggtctttcggttatgttagtgaataagaaaaggccagcg 187
            ||||||||||||||||||||||||||||||||||||||||||||||||||||||||||||
Sbjct: 61   gtgttattcgtatgtctgataggtctttcggttatgttagtgaataagaaaaggccagcg 120


Query: 188  aaggaggtgatttcggttcaggaaacaaatctagttttgtatgaaggcccaaagtcttta 247
            ||||||||||||||||||||||||||||||||||||||||||||||||||||||||||||
Sbjct: 121  aaggaggtgatttcggttcaggaaacaaatctagttttgtatgaaggcccaaagtcttta 180


Query: 248  cgtgatgctaccaaggaggatttggtttctgcaaacgagagctctaaagatttttcactt 307
            ||||||||||||||||||||||||||||||||||||||||||||||||||||||||||||
Sbjct: 181  cgtgatgctaccaaggaggatttggtttctgcaaacgagagctctaaagatttttcactt 240


Query: 308  cttcattgtaccgatactaaaataatggtaaatggatatgactgttatgtatatgataca 367
            ||||||||||||||||||||||||||||||||||||||||||||||||||||||||||||
Sbjct: 241  cttcattgtaccgatactaaaataatggtaaatggatatgactgttatgtatatgataca 300


Query: 368  aatgtaaatcattctagaagatggtataacgattacatgcctccaaatgcaagaactcca 427
            ||||||||||||||||||||||||||||||||||||||||||||||||||||||||||||
Sbjct: 301  aatgtaaatcattctagaagatggtataacgattacatgcctccaaatgcaagaactcca 360


Query: 428  atttcctattttgattttgaaggtatcgtagaagtaacaattaaggtacctaatattgac 487
            ||||||||||||||||||||||||||||||||||||||||||||||||||||||||||||
Sbjct: 361  atttcctattttgattttgaaggtatcgtagaagtaacaattaaggtacctaatattgac 420


Query: 488  ctagaaacagctagcgtaagtccattgcaatatggaattataccagtacttgatgtagaa 547
            ||||||||||||||||||||||||||||||||||||||||||||||||||||||||||||
Sbjct: 421  ctagaaacagctagcgtaagtccattgcaatatggaattataccagtacttgatgtagaa 480


Query: 548  aaccataccgttacatttacaattacaaaacctgaccaatataccataatgtttaataac 607
            ||||||||||||||||||||||||||||||||||||||||||||||||||||||||||||
Sbjct: 481  aaccataccgttacatttacaattacaaaacctgaccaatataccataatgtttaataac 540


Query: 608  tcaccggaaagagcggtacatttatttgcgaatgaaatagagacgaatataccatccaaa 667
            ||||||||||||||||||||||||||||||||||||||||||||||||||||||||||||
Sbjct: 541  tcaccggaaagagcggtacatttatttgcgaatgaaatagagacgaatataccatccaaa 600


Query: 668  gatgataaagatgttgtatatattggacctggagaatggaatattgaaaacattattctt 727
            ||||||||||||||||||||||||||||||||||||||||||||||||||||||||||||
Sbjct: 601  gatgataaagatgttgtatatattggacctggagaatggaatattgaaaacattattctt 660
```

```
Query: 728  gaggataatcaaacactatatatctcgggtggtgctgtaattcatggcatagtaaatgct 787
            ||||||||||||||||||||||||||||||||||||||||||||||||||||||||||||
Sbjct: 661  gaggataatcaaacactatatatctcgggtggtgctgtaattcatggcatagtaaatgct 720


Query: 788  aattttgctaagaatatcaccgttcgcggaagaggaattatcgatggttctaaatttaat 847
            ||||||||||||||||||||||||||||||||||||||||||||||||||||||||||||
Sbjct: 721  aattttgctaagaatatcaccgttcgcggaagaggaattatcgatggttctaaatttaat 780


Query: 848  ggatggaagggaaaagaagcatacataccattaaagtttgataactgcgagaacatcacg 907
            ||||||||||||||||||||||||||||||||||||||||||||||||||||||||||||
Sbjct: 781  ggatggaagggaaaagaagcatacataccattaaagtttgataactgcgagaacatcacg 840


Query: 908  aataaagatattatagtattaaatccgattgcttgggtatgtcaggccttttatttctaa 967
            | |||||||||||||||||||||||||| ||||||||||||||||| | |||| ||||||
Sbjct: 841  attaaagatattatagtattaaatccgaatgcttgggtatgtcagg-catttaattctaa 899


Query: 968  aaacggaactataaatattataaaaattatatccccagggccaaatgggg 1017
            |||||| |||||| ||| |||||||||||||||| || ||||||||||||
Sbjct: 900  aaacggtactatagataatataaaaattatatcctcaaggccaaatgggg 949


#######

Query= HR0ADKBY82ZG01_3310C1P17_1.SCF   1363      0   1363  SCF

>cphy3310 reverse

Query: 65   gtgtaatggatagtgatcttaatggtgatggtgatgatgctcaaggataatattctttgt 124
            ||||||||||||||||||||||||||||||||||||||||||||||||||||||||||||
Sbjct: 1611 gtgtaatggatagtgatcttaatggtgatggtgatgatgctcaaggataatattctttgt 1552


Query: 125  agtcttttcatctacttcaaattttccttgttccaaattcgtgatcttctctcctaacac 184
            ||||||||||||||||||||||||||||||||||||||||||||||||||||||||||||
Sbjct: 1551 agtcttttcatctacttcaaattttccttgttccaaattcgtgatcttctctcctaacac 1492


Query: 185  attgatatttttaaaataacaccatcaatgttatgctctgcatcaaagcctttaattct 244
            ||||||||||||||||||||||||||||||||||||||||||||||||||||||||||||
Sbjct: 1491 attgatatttttaaaataacaccatcaatgttatgctctgcatcaaagcctttaattct 1432


Query: 245  tgaagagcagtttttaccaccaagcacatctactccgtcaattacgacatttttgatagt 304
            ||||||||||||||||||||||||||||||||||||||||||||||||||||||||||||
Sbjct: 1431 tgaagagcagtttttaccaccaagcacatctactccgtcaattacgacatttttgatagt 1372


Query: 305  tcctcggtctctagttgaggaccaattagaactcccggcaatattaatgtcaatgaggta 364
            ||||||||||||||||||||||||||||||||||||||||||||||||||||||||||||
Sbjct: 1371 tcctcggtctctagttgaggaccaattagaactcccggcaatattaatgtcaatgaggta 1312


Query: 365  tggcatctcgtcaccatcgccacttcccatctgagcattttcaactacaatatttttgaa 424
            ||||||||||||||||||||||||||||||||||||||||||||||||||||||||||||
```

```
Sbjct: 1311 tggcatctcgtcaccatcgccacttcccatctgagcattttcaactacaatatttttgaa 1252


Query: 425  agtaattccacttatcgctgcatcatctgcattatgaactgatattacaggcttatggaa 484
            ||||||||||||||||||||||||||||||||||||||||||||||||||||||||||||
Sbjct: 1251 agtaattccacttatcgctgcatcatctgcattatgaactgatattacaggcttatggaa 1192


Query: 485  gttatgaagcaccgtaatattctcaaatgtgatatcagagataaaagcattctctcgttt 544
            ||||||||||||||||||||||||||||||||||||||||||||||||||||||||||||
Sbjct: 1191 gttatgaagcaccgtaatattctcaaatgtgatatcagagataaaagcattctctcgttt 1132


Query: 545  gcctttattcgtttcatatccaacctccatagactgcgcaaaatccgtccatagctgcat 604
            ||||||||||||||||||||||||||||||||||||||||||||||||||||||||||||
Sbjct: 1131 gcctttattcgtttcatatccaacctccatagactgcgcaaaatccgtccatagctgcat 1072


Query: 605  attttcaaactttatatttttgagttatcatcataattttaattaccaaagaatcatc 664
            ||||||||||||||||||||||||||||||||||||||||||||||||||||||||||||
Sbjct: 1071 attttcaaactttatatttttgagttatcatcataattttaattaccaaagaatcatc 1012


Query: 665  ccagcttcgaacaaaaccatttttaacggtgtaatcttggcaggactgtaaagtgatacc 724
            ||||||||||||||||||||||||||||||||||||||||||||||||||||||||||||
Sbjct: 1011 ccagcttcgaacaaaaccatttttaacggtgtaatcttggcaggactgtaaagtgatacc 952


Query: 725  atccccatttggccttgaggatataatttttatattatctatagtaccgtttttagaatt 784
            ||||||||||||||||||||||||||||||||||||||||||||||||||||||||||||
Sbjct: 951  atccccatttggccttgaggatataatttttatattatctatagtaccgtttttagaatt 892


Query: 785  aaatgcctgacatacccaagcattcggatttaatactataatatctttaatcgtgatgtt 844
            ||||||||||||||||||||||||||||||||||||||||||||||||||||||||||||
Sbjct: 891  aaatgcctgacatacccaagcattcggatttaatactataatatctttaatcgtgatgtt 832


Query: 845  ctcgcagttatcaaactttaatggtatgtatgcttcttttcccttccatccattaaatta 904
            ||||||||||||||||||||||||||||||||||||||||||||||||||||||||||| 
Sbjct: 831  ctcgcagttatcaaactttaatggtatgtatgcttcttttcccttccatccattaaattt 772


Query: 905  aaaaccatcgataattcctcttccgcgaacggggaaattcttagcaaaattaacatttac 964
            | |||||||||||||||||||||||||||||| || |||||||||||||||| |||||||
Sbjct: 771  agaaccatcgataattcctcttccgcgaacggtgatattcttagcaaaattagcatttac 712


Query: 965  tatgccatgaatttacagnnnnnnnngaaaaatatagggtttgattatccccaaaaataa 1024
            ||||||||||| ||||||        || | |||||| |||||||||||| ||| |||||
Sbjct: 711  tatgccatgaa-ttacagcaccacccgagatatatagtgtttgattatcctcaagaataa 653


Query: 1025 aggtttcaaaattccatt 1042
             | |||||| ||||||||
Sbjct: 652  tgttttcaatattccatt 635

#######
```

```
Query= HR0ADKBY82ZG08_3368C3P16_1.SCF   1396      0   1396  SCF


>Cphy3368 forward

Query: 68   aaagaaggagataggatcATGggtgaaactgagcaagctttagcggcggcagcaacaagg 127
            ||||||||||||||||||||||||||||||||||||||||||||||||||||||||||||
Sbjct: 1    aaagaaggagataggatcatgggtgaaactgagcaagctttagcggcggcagcaacaagg 60


Query: 128  ggaacctatgagcaacgttttatggacttatggtcggatattaaaaacccaaagaacggt 187
            ||||||||||||||||||||||||||||||||||||||||||||||||||||||||||||
Sbjct: 61   ggaacctatgagcaacgttttatggacttatggtcggatattaaaaacccaaagaacggt 120


Query: 188  tattttagtcctcagggaattccgtatcattctattgaaacaatgattgtagaagctcct 247
            ||||||||||||||||||||||||||||||||||||||||||||||||||||||||||||
Sbjct: 121  tattttagtcctcagggaattccgtatcattctattgaaacaatgattgtagaagctcct 180


Query: 248  gattatggtcatgtaactactagtgaggcaatgagttactatatgtggcttgaagctatg 307
            ||||||||||||||||||||||||||||||||||||||||||||||||||||||||||||
Sbjct: 181  gattatggtcatgtaactactagtgaggcaatgagttactatatgtggcttgaagctatg 240


Query: 308  tacggcaagtttacaggtgacttttctggatatgggaccgcttggaatgtagcagaaaaa 367
            ||||||||||||||||||||||||||||||||||||||||||||||||||||||||||||
Sbjct: 241  tacggcaagtttacaggtgacttttctggatatgggaccgcttggaatgtagcagaaaaa 300


Query: 368  tatatgattccaacggatgcagatcaaccaccaaccagtatgagtaagtatacaccgagt 427
            ||||||||||||||||||||||||||||||||||||||||||||||||||||||||||||
Sbjct: 301  tatatgattccaacggatgcagatcaaccaccaaccagtatgagtaagtatacaccgagt 360


Query: 428  aaacctgcaacttatgcacctgagtatcaggatcctagtcagtacccagcgaagctcgat 487
            ||||||||||||||||||||||||||||||||||||||||||||||||||||||||||||
Sbjct: 361  aaacctgcaacttatgcacctgagtatcaggatcctagtcagtacccagcgaagctcgat 420


Query: 488  tcgagtgctcctgttggtagtgacccaatttggtcacagcttgttgcagcttatggacgg 547
            ||||||||||||||||||||||||||||||||||||||||||||||||||||||||||||
Sbjct: 421  tcgagtgctcctgttggtagtgacccaatttggtcacagcttgttgcagcttatggacgg 480


Query: 548  aatacaatctatggtatgcactggttactagatgttgataactggtatggatttggttct 607
            ||||||||||||||||||||||||||||||||||||||||||||||||||||||||||||
Sbjct: 481  aatacaatctatggtatgcactggttactagatgttgataactggtatggatttggttct 540


Query: 608  aggggagacggaacctcaaaaccatcctatatcaacacattccaacgtggagaacaggaa 667
            ||||||||||||||||||||||||||||||||||||||||||||||||||||||||||||
Sbjct: 541  aggggagacggaacctcaaaaccatcctatatcaacacattccaacgtggagaacaggaa 600


Query: 668  tcaacatgggaaacaattcctcagccatgctgggatacaatgaaatatggtggaacgaat 727
```

```
            ||||||||||||||||||||||||||||||||||||||||||||||||||||||||||||
Sbjct: 601  tcaacatgggaaacaattcctcagccatgctgggatacaatgaaatatggtggaacgaat 660


Query: 728  ggtttcctcgacttattcactggcgatagttcctatgcacagcaatttaagtatacggat 787
            ||||||||||||||||||||||||||||||||||||||||||||||||||||||||||||
Sbjct: 661  ggtttcctcgacttattcactggcgatagttcctatgcacagcaatttaagtatacggat 720


Query: 788  gcaccagatgctgatgcaagagcaattcaggctgcttattgggcaagtgaatgggcgaaa 847
            ||||||||||||||||||||||||||||||||||||||||||||||||||||||||||||
Sbjct: 721  gcaccagatgctgatgcaagagcaattcaggctgcttattgggcaagtgaatgggcgaaa 780


Query: 848  gattatggtgtaaatgtcgatacttattcctcaaaacctacgatgatgggcgattatctc 907
            ||||||||||||||||||||||||||||| |||||| |||||||||||||||||||||| 
Sbjct: 781  gattatggtgtaaatgtcgatacttattcatcaaaagctacgatgatgggcgattatctt 840


Query: 908  cgttattcctgttttgataaatattttnnnnnnnnngg-aatttcacagttgctgggaaa 966
            |||||||||   |||||||||||||||         || ||||  ||||||||||| | |
Sbjct: 841  cgttattccatgtttgataaatattttagaaaaataggtaattctacagttgctggtaca 900


Query: 967  ggaaataatgcatcaaataatctggtatccgggtattatggcttggggcggtggaattac 1026
            ||| || |||||||| || ||||| ||||| |||||||| ||||||||||||||||||||
Sbjct: 901  ggatatgatgcatcacattatctgttatcctggtattat-gcttggggcggtggaattac 959


Query: 1027 agctgatggggcatggggttttgggatctagccctaaacccctttgggttatcaaaatcc 1086
            ||||||| |||||| ||||| | |||||||||| | |||| |||| ||||||||||||||
Sbjct: 960  agctgattgggcat-gggttattggatctagccat-aaccactt t-ggttatcaaaatcc 1016

#######

Query= HR0ADKBY82ZG08_3368C3P17_1.SCF   1390      0   1390  SCF

>Cphy3368 reverse

Query: 66   gtgtaatggatagtgatcttaatggtgatggtgatgatgtggttcgataccccaattaag 125
            ||||||||||||||||||||||||||||||||||||||||||||||||||||||||||||
Sbjct: 2718 gtgtaatggatagtgatcttaatggtgatggtgatgatgtggttcgataccccaattaag 2659


Query: 126  ttttccacttatataagcagttgccttgttccaatcaacataggagttaccgctgctatt 185
            ||||||||||||||||||||||||||||||||||||||||||||||||||||||||||||
Sbjct: 2658 ttttccacttatataagcagttgccttgttccaatcaacataggagttaccgctgctatt 2599


Query: 186  aaatgaataatccccagtctgggtataattagaccagtcagtttttgagaatctaccttg 245
            ||||||||||||||||||||||||||||||||||||||||||||||||||||||||||||
Sbjct: 2598 aaatgaataatccccagtctgggtataattagaccagtcagtttttgagaatctaccttg 2539


Query: 246  aacttcgattccctgtcctgcctttaaagaaccagcaccacttgtaaatccaatctcaag 305
            ||||||||||||||||||||||||||||||||||||||||||||||||||||||||||||
Sbjct: 2538 aacttcgattccctgtcctgcctttaaagaaccagcaccacttgtaaatccaatctcaag 2479
```

```
Query: 306  ataataatctgctccagtctttggagttgccatctttacaaaagttccggtaacattact 365
            ||||||||||||||||||||||||||||||||||||||||||||||||||||||||||||
Sbjct: 2478 ataataatctgctccagtctttggagttgccatctttacaaaagttccggtaacattact 2419


Query: 366  gcttccaatcgatgaccaatcacaccagaaactctggctcttctcaccatcaattgtata 425
            ||||||||||||||||||||||||||||||||||||||||||||||||||||||||||||
Sbjct: 2418 gcttccaatcgatgaccaatcacaccagaaactctggctcttctcaccatcaattgtata 2359


Query: 426  gtagtaacgaagctttacatcagaaagattaatactattagagcctgtatttacaagata 485
            ||||||||||||||||||||||||||||||||||||||||||||||||||||||||||||
Sbjct: 2358 gtagtaacgaagctttacatcagaaagattaatactattagagcctgtatttacaagata 2299


Query: 486  aaaacgaggtgcaattccattcgttgttgcactagtatttccattgaacatctgaacctt 545
            ||||||||||||||||||||||||||||||||||||||||||||||||||||||||||||
Sbjct: 2298 aaaacgaggtgcaattccattcgttgttgcactagtatttccattgaacatctgaacctt 2239


Query: 546  aacatctcctactaccacaactactgttgtatccacaacggttactacaaaaggttttgt 605
            ||||||||||||||||||||||||||||||||||||||||||||||||||||||||||||
Sbjct: 2238 aacatctcctactaccacaactactgttgtatccacaacggttactacaaaaggttttgt 2179


Query: 606  ataagaattgctaaaattaaaagtaattgtagtatcaccaagtggtaatgtagataggaa 665
            ||||||||||||||||||||||||||||||||||||||||||||||||||||||||||||
Sbjct: 2178 ataagaattgctaaaattaaaagtaattgtagtatcaccaagtggtaatgtagataggaa 2119


Query: 666  ttctttcttaaggactactgtagtaccattaacagtatagtctgtatttaaagtcagagg 725
            ||||||||||||||||||||||||||||||||||||||||||||||||||||||||||||
Sbjct: 2118 ttctttcttaaggactactgtagtaccattaacagtatagtctgtatttaaagtcagagg 2059


Query: 726  tgtagtacctttacttaagttaacaaaggtataaccttgcattgataatgttgtactaag 785
            ||||||||||||||||||||||||||||||||||||||||||||||||||||||||||||
Sbjct: 2058 tgtagtacctttacttaagttaacaaaggtataaccttgcattgataatgttgtactaag 1999


Query: 786  atctgcttgttttcctacagctttatcaaaggaaccatttgtaggattaatattagcata 845
            ||||||||||||||||||||||||||||||||||||||||||||||||||||||||||||
Sbjct: 1998 atctgcttgttttcctacagctttatcaaaggaaccatttgtaggattaatattagcata 1939


Query: 846  atcagtattgccagaaccattctcataaaggagtgcatataccaccattggcaagcgcta 905
            ||||||| ||||||||||||||||||||||||||||||||| ||||||| ||||||||||
Sbjct: 1938 atcagtagtgccagaaccattctcataaaggagtgcatata-caccatttgcaagcgcta 1880


Query: 906  catcacactgaccccaaaatctaagaaaaataactcctggtgacttattactc 958
            ||||||||||| |||| |||||| || | ||||   |||||||||||||||||
Sbjct: 1879 catcacactgagcccagaatctatgatatataaactctggtgacttattactc 1827
```

#######

```
Query= HR0ADKBY82ZH05_3854C5P16_1.SCF   1382      0   1382  SCF

>cphy3854 forward



Query: 70  aaagaaggagataggatcATGcaatacggacattttgatcaagnnnnnnnngagtatgtc 129
            |||||||||||||||||||||||||||||||||||||||||||        |||||||||
Sbjct: 1   aaagaaggagataggatcatgcaatacggacattttgatcaagaaaaaaaagagtatgtc 60


Query: 130 atcgaccgcgttgaccttccaacctcatggaccaattatctaggggttaaagacacttgt 189
            ||||||||||||||||||||||||||||||||||||||||||||||||||||||||||||
Sbjct: 61  atcgaccgcgttgaccttccaacctcatggaccaattatctaggggttaaagacacttgt 120


Query: 190 gtggttgtaaatcaaaccgcaggtggctacatgttttataaatcaccagaataccaccgt 249
            ||||||||||||||||||||||||||||||||||||||||||||||||||||||||||||
Sbjct: 121 gtggttgtaaatcaaaccgcaggtggctacatgttttataaatcaccagaataccaccgt 180


Query: 250 gttacaagatttcgcggaaatagtgtaccaatggatcgccctgggcattatgtatacctt 309
            ||||||||||||||||||||||||||||||||||||||||||||||||||||||||||||
Sbjct: 181 gttacaagatttcgcggaaatagtgtaccaatggatcgccctgggcattatgtatacctt 240


Query: 310 agggacaacgagacgaaggattactggagtgtttcatggcaaccagtaggtaagccatta 369
            ||||||||||||||||||||||||||||||||||||||||||||||||||||||||||||
Sbjct: 241 agggacaacgagacgaaggattactggagtgtttcatggcaaccagtaggtaagccatta 300


Query: 370 gatgaagctaagtatacctgccgtcatggaatgtcctattccgtctacgaatgtgattat 429
            ||||||||||||||||||||||||||||||||||||||||||||||||||||||||||||
Sbjct: 301 gatgaagctaagtatacctgccgtcatggaatgtcctattccgtctacgaatgtgattat 360


Query: 430 cagaaattaaaagcgacacaaacccttgttgtaccaatcgatgaagatgtggaactttgg 489
            ||||||||||||||||||||||||||||||||||||||||||||||||||||||||||||
Sbjct: 361 cagaaattaaaagcgacacaaacccttgttgtaccaatcgatgaagatgtggaactttgg 420


Query: 490 gatgtaacagttacgaataacgattccaaaccaagaaatattagcctatttacttattgt 549
            ||||||||||||||||||||||||||||||||||||||||||||||||||||||||||||
Sbjct: 421 gatgtaacagttacgaataacgattccaaaccaagaaatattagcctatttacttattgt 480


Query: 550 gaattttcttttcatcatatcatgatagataatcaaaacttccagatgagcctttactgt 609
            ||||||||||||||||||||||||||||||||||||||||||||||||||||||||||||
Sbjct: 481 gaattttcttttcatcatatcatgatagataatcaaaacttccagatgagcctttactgt 540


Query: 610 gctggttcttcctatgaagatggtattaccctgcatgatttattctatgaagaatttggt 669
            ||||||||||||||||||||||||||||||||||||||||||||||||||||||||||||
Sbjct: 541 gctggttcttcctatgaagatggtattaccctgcatgatttattctatgaagaatttggt 600


Query: 670 tatcaatactttacctcaacccaaacaccagatggttatgactgtctccgcgataaattc 729
```

```
           ||||||||||||||||||||||||||||||||||||||||||||||||||||||||||||
Sbjct: 601 tatcaatactttacctcaacccaaacaccagatggttatgactgtctccgcgataaattc 660


Query: 730 cttggcctttatcatacagaatctgatccaattggagttattaatggggaattaagtggt 789
           ||||||||||||||||||||||||||||||||||||||||||||||||||||||||||||
Sbjct: 661 cttggcctttatcatacagaatctgatccaattggagttattaatggggaattaagtggt 720


Query: 790 agtacagaacttggaaataatcactgtggttctcctccacataattttgtgattcaccca 849
           |||||||||||||||||||||||||||||||||| || |||||||||||||||||| |||
Sbjct: 721 agtacagaacttggaaataatcactgtggttctcttcaacataattttgtgattcagcca 780


Query: 850 accaaaccaattcaaaattgttatatgttaggcaaaaggaatca 893
           | | || |||||| || |  ||||||||||||| || |||||||
Sbjct: 781 aacgaaacaattcgaattgtttatatgttaggcgaagggaatca 824

#######

Query= HR0ADKBY82ZH05_3854C5P17_1.SCF   1337      0   1337  SCF

>cphy3854 reverse


Query: 66   gtgtaatggatagtgatcttaatggtgatggtgatgatgcactcccataacaatgcgaac 125
            ||||||||||||||||||||||||||||||||||||||||||||||||||||||||||||
Sbjct: 2448 gtgtaatggatagtgatcttaatggtgatggtgatgatgcactcccataacaatgcgaac 2389


Query: 126  atggtgagtggtatgtggctcctgaactggaagtctttccaccaattttccatcaagata 185
            ||||||||||||||||||||||||||||||||||||||||||||||||||||||||||||
Sbjct: 2388 atggtgagtggtatgtggctcctgaactggaagtctttccaccaattttccatcaagata 2329


Query: 186  gatttctttgacgcccttcataactccatctggattctcaacagcaatctcatagactgc 245
            ||||||||||||||||||||||||||||||||||||||||||||||||||||||||||||
Sbjct: 2328 gatttctttgacgcccttcataactccatctggattctcaacagcaatctcatagactgc 2269


Query: 246  acctctccattctctagttacttggaagctatcccatgagccaggaatgcaaggattaat 305
            ||||||||||||||||||||||||||||||||||||||||||||||||||||||||||||
Sbjct: 2268 acctctccattctctagttacttggaagctatcccatgagccaggaatgcaaggattaat 2209


Query: 306  ttctaaataatcaaattgaggtttaataccgagcatatatctagtggcactaaaatatgc 365
            ||||||||||||||||||||||||||||||||||||||||||||||||||||||||||||
Sbjct: 2208 ttctaaataatcaaattgaggtttaataccgagcatatatctagtggcactaaaatatgc 2149


Query: 366  ccaaccgccagtaccagtcataaatgggtgtcttgcacgaccaaaggcagtgtgatctct 425
            ||||||||||||||||||||||||||||||||||||||||||||||||||||||||||||
Sbjct: 2148 ccaaccgccagtaccagtcataaatgggtgtcttgcacgaccaaaggcagtgtgatctct 2089


Query: 426  acccataataaactggcaataggaatatggctctgcttctcgaatttcaattttatcatt 485
            ||||||||||||||||||||||||||||||||||||||||||||||||||||||||||||
Sbjct: 2088 acccataataaactggcaataggaatatggctctgcttctcgaatttcaattttatcatt 2029
```

```
Query: 486  ttgataatatgggcaaagtgcattataaaattccattgctctgtcaccacggccaagttc 545
            ||||||||||||||||||||||||||||||||||||||||||||||||||||||||||||
Sbjct: 2028 ttgataatatgggcaaagtgcattataaaattccattgctctgtcaccacggccaagttc 1969


Query: 546  acattcagcagcccatgcccatggatttgggtgactaaaaattgctccattttcttttaa 605
            ||||||||||||||||||||||||||||||||||||||||||||||||||||||||||||
Sbjct: 1968 acattcagcagcccatgcccatggatttgggtgactaaaaattgctccattttcttttaa 1909


Query: 606  tccaggataaacacgggtaacaaatccaatgtcatcatcaggcactgtataggaaggagc 665
            ||||||||||||||||||||||||||||||||||||||||||||||||||||||||||||
Sbjct: 1908 tccaggataaacacgggtaacaaatccaatgtcatcatcaggcactgtataggaaggagc 1849


Query: 666  attcagtaaaattccatatggagtatataaatgctcatatacactatccatagctttgag 725
            ||||||||||||||||||||||||||||||||||||||||||||||||||||||||||||
Sbjct: 1848 attcagtaaaattccatatggagtatataaatgctcatatacactatccatagctttgag 1789


Query: 726  acctttttcttcggtagctgcaccggataatacagcccaagagttagactctaagtgaat 785
            ||||||||||||||||||||||||||||||||||||||||||||||||||||||||||||
Sbjct: 1788 acctttttcttcggtagctgcaccggataatacagcccaagagttagactctaagtgaat 1729


Query: 786  cttaccttctttatcatttattgtaccaannnnnnnaccgtttttcgtaattccacggat 845
            |||||||||||||||||||||||||||||       ||||||||||||||||||||||||
Sbjct: 1728 cttaccttctttatcatttattgtaccaatttttttaccgtttttcgtaattccacggat 1669


Query: 846  aaaccattcctgatcccataattctttattacatacttcttttacatgatcggctaaagc 905
            ||||||||||||||||||||||||||||||||||||||||||||||||||||||||||||
Sbjct: 1668 aaaccattcctgatcccataattctttattacatacttcttttacatgatcggctaaagc 1609


Query: 906  tgtgtatttctgtacatcctcttcacgatttaaaaacttcgcaagagaaataaaattatt 965
            |||||||||||||||||||||||||||||||||||||||||||||||| |||||||||||
Sbjct: 1608 tgtgtatttctgtacatcctcttcacgatttaaaaacttcgcaagagagataaaattatt 1549


Query: 966  aagaacccagtaatgca 982
            |||| ||||||| ||||
Sbjct: 1548 aagagcccagtagtgca 1532


########

Query= HR0ADKBY82ZH06_3862C1P16_1.SCF   1378      0   1378  SCF

>Cphy3862 forward

Query: 70   aaagaaggagataggatcATGtcaacaacagcatattccatggcgtctgatagcgacatc 129
            ||||||||||||||||||||||||||||||||||||||||||||||||||||||||||||
Sbjct: 1    aaagaaggagataggatcatgtcaacaacagcatattccatggcgtctgatagcgacatc 60
```

```
Query: 130  cagagtaaaacaatcggtaccacattcgatggcaccacatggtttcagaaagccggcggc 189
            ||||||||||||||||||||||||||||||||||||||||||||||||||||||||||||
Sbjct: 61   cagagtaaaacaatcggtaccacattcgatggcaccacatggtttcagaaagccggcggc 120


Query: 190  ccgacgcttactgttgttgacaacagtggagccaaagccattagtgttacagggcggact 249
            ||||||||||||||||||||||||||||||||||||||||||||||||||||||||||||
Sbjct: 121  ccgacgcttactgttgttgacaacagtggagccaaagccattagtgttacagggcggact 180


Query: 250  gcagactggaatagcgttgatttgaaaaatttagtttccttgcctgatgggtttgagtac 309
            ||||||||||||||||||||||||||||||||||||||||||||||||||||||||||||
Sbjct: 181  gcagactggaatagcgttgatttgaaaaatttagtttccttgcctgatgggtttgagtac 240


Query: 310  actatcatggtgaccggccgtacttctgagggctccaagatgaagctatcacagacggca 369
            ||||||||||||||||||||||||||||||||||||||||||||||||||||||||||||
Sbjct: 241  actatcatggtgaccggccgtacttctgagggctccaagatgaagctatcacagacggca 300


Query: 370  agcccgtatggaacacatgcttctttagttgtgggcacagatggtattttctctcttgag 429
            ||||||||||||||||||||||||||||||||||||||||||||||||||||||||||||
Sbjct: 301  agcccgtatggaacacatgcttctttagttgtgggcacagatggtattttctctcttgag 360


Query: 430  aagaccttcacgtatacccagttacagactgagaagaccgttcgtatccagtcggaagga 489
            ||||||||||||||||||||||||||||||||||||||||||||||||||||||||||||
Sbjct: 361  aagaccttcacgtatacccagttacagactgagaagaccgttcgtatccagtcggaagga 420


Query: 490  acaaccaacgactttacaattaacagcatcataatcacccagacagcagtctcagatggc 549
            ||||||||||||||||||||||||||||||||||||||||||||||||||||||||||||
Sbjct: 421  acaaccaacgactttacaattaacagcatcataatcacccagacagcagtctcagatggc 480


Query: 550  gtaacatctacacctacacctaccccgatttctgatggtgcaactactagcgacatctct 609
            ||||||||||||||||||||||||||||||||||||||||||||||||||||||||||||
Sbjct: 481  gtaacatctacacctacacctaccccgatttctgatggtgcaactactagcgacatctct 540


Query: 610  atttccttcaataccaccgacaataccaagtggagcgaagcttttagcgtgtccaacaca 669
            ||||||||||||||||||||||||||||||||||||||||||||||||||||||||||||
Sbjct: 541  atttccttcaataccaccgacaataccaagtggagcgaagcttttagcgtgtccaacaca 600


Query: 670  gataatgccgcaattgaatgggtttcggacttcggtaatggcgatacttttgcacttaag 729
            ||||||||||||||||||||||||||||||||||||||||||||||||||||||||||||
Sbjct: 601  gataatgccgcaattgaatgggtttcggacttcggtaatggcgatacttttgcacttaag 660


Query: 730  ggtatccacctatctacctcgactgactataccggcgctaacaacgccatccgtctgaca 789
            ||||||||||||||||||||||||||||||||||||||||||||||||||||||||||||
Sbjct: 661  ggtatccacctatctacctcgactgactataccggcgctaacaacgccatccgtctgaca 720


Query: 790  tttcctgagccattggctaagaacgccgtttacactatttcgtatagcgtctttgtccca 849
            ||||||||||||||||||||||||||||||||||||||||||||||||||||||||||||
Sbjct: 721  tttcctgagccattggctaagaacgccgtttacactatttcgtatagcgtctttgtccca 780
```

```
Query: 850  gccgtaggcaacgagggtaaagatacacttgtaggaccgggcatcgtcctgagtggcgat 909
            ||||||||||||||||||||||||||||||||||||||||||||||||||||||||||||
Sbjct: 781  gccgtaggcaacgagggtaaagatacacttgtaggaccgggcatcgtcctgagtggcgat 840


Query: 910  tatacaggctccacaggcgtgaccaaattcccggccaacttccgcacaattagtaacaag 969
            ||||||||||| |||||||||||||||||||||||| ||||| ||||||||||||   ||
Sbjct: 841  tatacaggctcaacaggcgtgaccaaattcccggccgacttcggcacaattagtacagag 900


Query: 970  acatggaaagatgttagtataacccaccccggaagggtgttctgaacgaaaacctaaaga 1029
            |||||||||||||||||||||| |||| |||||| |||| ||||||||| |  |||||||
Sbjct: 901  acatggaaagatgttagtataa-ccacaccggaa-ggtggtctgaacgagacactaaaga 958


Query: 1030 ccatccaccttccgtttccttgtaaa 1055
             |||| | |||||||||| |||||||
Sbjct: 959  gcatcga-cttccgtttcgttgtaaa 983

########


Query= HR0ADKBY82ZH06_3862C1P17_1.SCF   1415      0   1415  SCF

>cphy3862 reverse

Query: 66   gtgtaatggatagtgatcttaatggtgatggtgatgatgctcttcaataataactttaat 125
            ||||||||||||||||||||||||||||||||||||||||||||||||||||||||||||
Sbjct: 7329 gtgtaatggatagtgatcttaatggtgatggtgatgatgctcttcaataataactttaat 7270


Query: 126  tgacttcttcataccatctacatttacataaatccaatctgtttttgcagaaacggcctt 185
            ||||||||||||||||||||||||||||||||||||||||||||||||||||||||||||
Sbjct: 7269 tgacttcttcataccatctacatttacataaatccaatctgtttttgcagaaacggcctt 7210


Query: 186  aactgttgcagttgtacttcctggattcttcttcacaactactccatcctttttactaga 245
            ||||||||||||||||||||||||||||||||||||||||||||||||||||||||||||
Sbjct: 7209 aactgttgcagttgtacttcctggattcttcttcacaactactccatcctttttactaga 7150


Query: 246  catccagaggatgctatcttcatctaaaccatttacttttatttaaatactgcttcttc 305
            ||||||||||||||||||||||||||||||||||||||||||||||||||||||||||||
Sbjct: 7149 catccagaggatgctatcttcatctaaaccatttacttttatttaaatactgcttcttc 7090


Query: 306  tcccactttcatatgcacagcactttccacaaattcaatggtcgctttttttaacagataa 365
            ||||||||||||||||||||||||||||||||||||||||||||||||||||||||||||
Sbjct: 7089 tcccactttcatatgcacagcactttccacaaattcaatggtcgctttttttaacagataa 7030


Query: 366  ttttctagtataaaccttctgtgttccatcctgcattgtaacagtagccgtaataactat 425
            ||||||||||||||||||||||||||||||||||||||||||||||||||||||||||||
Sbjct: 7029 ttttctagtataaaccttctgtgttccatcctgcattgtaacagtagccgtaataactat 6970
```

```
Query: 426  aataccagattcttttgcaatcaaacgtccattctgttttacttctacaatacctggatt 485
            ||||||||||||||||||||||||||||||||||||||||||||||||||||||||||||
Sbjct: 6969 aataccagattcttttgcaatcaaacgtccattctgttttacttctacaatacctggatt 6910


Query: 486  atttgacttataagtaatcgtaattggaactattccctcatcaagcttacttaatgtctt 545
            ||||||||||||||||||||||||||||||||||||||||||||||||||||||||||||
Sbjct: 6909 atttgacttataagtaatcgtaattggaactattccctcatcaagcttacttaatgtctt 6850


Query: 546  ttcatcaaagttagctactttaacaacgttatttggaagctttaatccaaaccatcctaa 605
            ||||||||||||||||||||||||||||||||||||||||||||||||||||||||||||
Sbjct: 6849 ttcatcaaagttagctactttaacaacgttatttggaagctttaatccaaaccatcctaa 6790


Query: 606  gttaaaactcttacctccaacatatagagtaccagtgcgttttggtgttaagtgctctag 665
            ||||||||||||||||||||||||||||||||||||||||||||||||||||||||||||
Sbjct: 6789 gttaaaactcttacctccaacatatagagtaccagtgcgttttggtgttaagtgctctag 6730


Query: 666  aatctcttctgttgttaactctggtgtaggagtaggggttgcagtcggggcacccgtaac 725
            ||||||||||||||||||||||||||||||||||||||||||||||||||||||||||||
Sbjct: 6729 aatctcttctgttgttaactctggtgtaggagtaggggttgcagtcggggcacccgtaac 6670


Query: 726  cgttggaacctccgtaaccgttggggttggcgtaatcgtaatagggccttcggaggcagg 785
            ||||||||||||||||||||||||||||||||||||||||||||||||||||||||||||
Sbjct: 6669 cgttggaacctccgtaaccgttggggttggcgtaatcgtaatagggccttcggaggcagg 6610


Query: 786  cttacgagtgaacgaatggtggattggagctgcaatcatattaccagcggcatcacttaa 845
            ||||||||||||||||||||||||||||||||||||||||||||||||||||||||||||
Sbjct: 6609 cttacgagtgaacgaatggtggattggagctgcaatcatattaccagcggcatcacttaa 6550


Query: 846  gcgcatagtataaatattgcaaacttcacctttcccttttgttacattgttaaatgtcac 905
            ||||||||||||||||||||||||||||||||||||||||||||||||||||||||||||
Sbjct: 6549 gcgcatagtataaatattgcaaacttcacctttcccttttgttacattgttaaatgtcac 6490


Query: 906  ttctacccatttgttatcatcctgcccatgtaaataaaaagtcatcggaagaaatcaaat 965
            ||||||| |||||||||||| |||||||||||| |||||||||||||| |||||||||||
Sbjct: 6489 ttctaccgatttgttatcat-ctgcccatgtaagtaaaaagtcatcgg-agaaatcaaat 6432

#######

> cphy1799 forward

Query  69   AAAGAAGGAGATAGGATCATGGTTAAGGCAGCTTATCCAACCTGGCAAGCAGGTACTGCA  128
            ||||||||||||||||||||||||||||||||||||||||||||||||||||||||||||
Sbjct  1    AAAGAAGGAGATAGGATCATGGTTAAGGCAGCTTATCCAACCTGGCAAGCAGGTACTGCA  60

Query  129  TATAAAGCTGGTGATATTGTATCCTATGGAAACAACAACTACCAATGTATCCAATCACAC  188
            ||||||||||||||||||||||||||||||||||||||||||||||||||||||||||||
Sbjct  61   TATAAAGCTGGTGATATTGTATCCTATGGAAACAACAACTACCAATGTATCCAATCACAC  120

Query  189  ACAGCACTTACAGGTTGGGAACCTCCAGTAGTTCCTGCACTATGGTCATTATATAGCGGT  248
            ||||||||||||||||||||||||||||||||||||||||||||||||||||||||||||
```

```
Sbjct  121  ACAGCACTTACAGGTTGGGAACCTCCAGTAGTTCCTGCACTATGGTCATTATATAGCGGT  180

Query  249  GGGACTACGCAAACCGTAGCTACACCTGCATTTAACCCGGCGGGTGGAACCTATACTGCC  308
            ||||||||||||||||||||||||||||||||||||||||||||||||||||||||||||
Sbjct  181  GGGACTACGCAAACCGTAGCTACACCTGCATTTAACCCGGCGGGTGGAACCTATACTGCC  240

Query  309  TCGCAAAGCGTAACAATTACCTGTTCGACTTCCGGTAGTACTATCCGCTATACCTTGAAT  368
            ||||||||||||||||||||||||||||||||||||||||||||||||||||||||||||
Sbjct  241  TCGCAAAGCGTAACAATTACCTGTTCGACTTCCGGTAGTACTATCCGCTATACCTTGAAT  300

Query  369  GGCAGTGAGCCTACCTCTTCTTCTACTGTCTATAGCGGAGCTATCTTGGTTGCATCCACA  428
            ||||||||||||||||||||||||||||||||||||||||||||||||||||||||||||
Sbjct  301  GGCAGTGAGCCTACCTCTTCTTCTACTGTCTATAGCGGAGCTATCTTGGTTGCATCCACA  360

Query  429  ACCACAGTAAAAGCAAAGGCTTTCCTCTCTGGCATGAATGATTCTCAAACAGCTACTTCC  488
            ||||||||||||||||||||||||||||||||||||||||||||||||||||||||||||
Sbjct  361  ACCACAGTAAAAGCAAAGGCTTTCCTCTCTGGCATGAATGATTCTCAAACAGCTACTTCC  420

Query  489  ACTTATACCATAAATAAAAACGATACTGTAGCAACTCCTGTATTTTCTGTAGCGGAAGGA  548
            ||||||||||||||||||||||||||||||||||||||||||||||||||||||||||||
Sbjct  421  ACTTATACCATAAATAAAAACGATACTGTAGCAACTCCTGTATTTTCTGTAGCGGAAGGA  480

Query  549  ACCTATTATGGAAACATTGATGTTGTCCTTACCTGTGCAACAAGTAATGCAACAATTCAA  608
            |||||||||||||||||||||||||||||||||||||||||||||||||||||||||| |
Sbjct  481  ACCTATTATGGAAACATTGATGTTGTCCTTACCTGTGCAACAAGTAATGCAACAATTCGA  540

Query  609  TATACTACAGATGGCACCACTCCTGTATCAACTTCAAAGGTATATACCGGTGCGATAGCA  668
            ||||||||||||||||||||||||||||||||||||||||||||||||||||||||||||
Sbjct  541  TATACTACAGATGGCACCACTCCTGTATCAACTTCAAAGGTATATACCGGTGCGATAGCA  600

Query  669  GTTACAGCAACTACTACAATTAAAACCTATGCAACAGCTTCCAATCTGAAAGATTCCGCT  728
            ||||||||||||||||||||||||||||||||||||||||||||||||||||||||||||
Sbjct  601  GTTACAGCAACTACTACAATTAAAACCTATGCAACAGCTTCCAATCTGAAAGATTCCGCT  660

Query  729  GTCGCAAGTGCAACATATACAATATCCAAAGAACCAAACAGGTCTTCCTACTCACATTCC  788
            |||||||||||||||||||||||||| |||||||||||||||||||||||||||||| ||
Sbjct  661  GTCGCAAGTGCAACATATACAATATC-AAAGAACCAAACAGGTCTTCCTACTCACAT-CC  718

Query  789  TGACTGGTTACTGGCAAAATTTTAAAAAACGGCCCCAAATTGTTAAAAAATCATTGATGT  848
            |||||||||||||||| |||||| | || |||| | || | ||| |||||||| ||||||
Sbjct  719  TGACTGGTTACTGGCAGAATTTTGATAA-CGGCGCTAAGT-GTTTAAAAATCAGTGATGT  776

Query  849  TCCCACAAAAC-ACCATATAAATTTCAAAAACTTTTTGCTAAACCCACAACCACCGCCCG  907
            ||| ||||| | || |||||| || ||| | ||||| ||| || | |||||||| ||| |
Sbjct  777  TCC-ACAAAGCTACAATATAA-TTGCAATAGCTTTT-GCTGAAGCAACAACCACTGCC-G  832

Query  908  GGGAAGTTACTTTTAAACTTGATATCTAATTCTTGTTTCAAAGTTTAGGCGGGCTAAACC  967
            | ||||||||||||||||| ||| |||||| |||| ||| ||||| |||||| ||| |||
Sbjct  833  GTGAAGTTACTTTTAAACTGGAT-TCTAAT-CTTGCTTCTAAGTT-AGGCGG-CTATACC  888

Query  968  CAAAAAAGCAAATTTATACAATG  990
            || |  |||| ||||| |||||
Sbjct  889  GAACA--GCAA-TTTAT-CAATG  907

> cphy1799 reverse


Query  67    GTGTAATGGATAGTGATCTTAATGGTGATGGTGATGATGCGGAAGAGTCTTTAATTTTGC  126
```

```
                ||||||||||||||||||||||||||||||||||||||||||||||||||||||||||||
Sbjct  1632   GTGTAATGGATAGTGATCTTAATGGTGATGGTGATGATGCGGAAGAGTCTTTAATTTTGC  1573

Query  127    ACCAACTGTATTGGAAAAATCATAATTATTGCTGGCATCCCAATTGATGGACCAGGTCAT  186
              ||||||||||||||||||||||||||||||||||||||||||||||||||||||||||||
Sbjct  1572   ACCAACTGTATTGGAAAAATCATAATTATTGCTGGCATCCCAATTGATGGACCAGGTCAT  1513

Query  187    TGCACCACGTAGTGTAGGGTATGTCTTAGGTGGTTTATAAGAACCACCGTTGGTACCTTT  246
              ||||||||||||||||||||||||||||||||||||||||||||||||||||||||||||
Sbjct  1512   TGCACCACGTAGTGTAGGGTATGTCTTAGGTGGTTTATAAGAACCACCGTTGGTACCTTT  1453

Query  247    TGTAAGGCAATCAAGAGCTGCATTTACAACACTTGGTGCAACATAGCCGCTACCAGCTCC  306
              ||||||||||||||||||||||||||||||||||||||||||||||||||||||||||||
Sbjct  1452   TGTAAGGCAATCAAGAGCTGCATTTACAACACTTGGTGCAACATAGCCGCTACCAGCTCC  1393

Query  307    CTTTGTGGAAGCAGGCAATCCAAGACCAACCTGATCGGGACGTAGTCCATTTTCTAGTTG  366
              ||||||||||||||||||||||||||||||||||||||||||||||||||||||||||||
Sbjct  1392   CTTTGTGGAAGCAGGCAATCCAAGACCAACCTGATCGGGACGTAGTCCATTTTCTAGTTG  1333

Query  367    AATAGCTGCCAAAGCAGTTATGAAATCCACAGAGCCTTGGGAGTATACCTTGCCATCCTG  426
              ||||||||||||||||||||||||||||||||||||||||||||||||||||||||||||
Sbjct  1332   AATAGCTGCCAAAGCAGTTATGAAATCCACAGAGCCTTGGGAGTATACCTTGCCATCCTG  1273

Query  427    ACCTAACATACTACCGGAGTTATAGTACTGAGTATTCACAACAGTTAAAATATCCTTGAT  486
              ||||||||||||||||||||||||||||||||||||||||||||||||||||||||||||
Sbjct  1272   ACCTAACATACTACCGGAGTTATAGTACTGAGTATTCACAACAGTTAAAATATCCTTGAT  1213

Query  487    ATTCAATGCTACCTGGAAATAACCTGAGTTTACAGACTGCATGTCAAGAGTCTGAGGGGC  546
              ||||||||||||||||||||||||||||||||||||||||||||||||||||||||||||
Sbjct  1212   ATTCAATGCTACCTGGAAATAACCTGAGTTTACAGACTGCATGTCAAGAGTCTGAGGGGC  1153

Query  547    CATTGTAATAATTAGTCCAGAACCAGCTTTTGTGGATAGTTGGCGTAATGCAGAAGTCAT  606
              ||||||||||||||||||||||||||||||||||||||||||||||||||||||||||||
Sbjct  1152   CATTGTAATAATTAGTCCAGAACCAGCTTTTGTGGATAGTTGGCGTAATGCAGAAGTCAT  1093

Query  607    GTAGGTTGCATTGATTCCGTTTTCTAAGTCAATATCTACACCGTCAAATCCGTATTTTAC  666
              ||||||||||||||||||||||||||||||||||||||||||||||||||||||||||||
Sbjct  1092   GTAGGTTGCATTGATTCCGTTTTCTAAGTCAATATCTACACCGTCAAATCCGTATTTTAC  1033

Query  667    CATGAGTGCATATACACTGTTAGCAAAATTAGTAGCTGAGGTCGTATTATTTACACTTAC  726
              ||||||||||||||||||||||||||||||||||||||||||||||||||||||||||||
Sbjct  1032   CATGAGTGCATATACACTGTTAGCAAAATTAGTAGCTGAGGTCGTATTATTTACACTTAC  973

Query  727    AGTACCGGTTTCACCACCTACAGAAATGATTACCTTCTGGCCCTTTGCCTTAGCTGCTGC  786
              ||||||||||||||||||||||||||||||||||||||||||||||||||||||||||||
Sbjct  972    AGTACCGGTTTCACCACCTACAGAAATGATTACCTTCTGGCCCTTTGCCTTAGCTGCTGC  913

Query  787    TATGTCATTGATAAATTGCTGTTCGGTATAGCCGCCTAACTTAGAAGCAAGATTAGAATC  846
              ||||||||||||||||||||||||||||||||||||||||||||||||||||||||||||
Sbjct  912    TATGTCATTGATAAATTGCTGTTCGGTATAGCCGCCTAACTTAGAAGCAAGATTAGAATC  853

Query  847    CAGTTTAAAAGTAACTTCACCGGCAGTGGTTGTTGCTTCACCAAAAGCTATTGCAATTAT  906
              |||||||||||||||||||||||||||||||||||||||| |||||||||||||||||||
Sbjct  852    CAGTTTAAAAGTAACTTCACCGGCAGTGGTTGTTGCTTCAGCAAAAGCTATTGCAATTAT  793

Query  907    ATTGTAACTTTGTGAAACATCACTGAATTTAAAACACTTACCGCCCTTATCAAAATTCTG  966
              |||||| ||||||| ||||||||||| ||| ||||||||| |||| ||||||||||||||
Sbjct  792    ATTGTAGCTTTGTGGAACATCACTGATTTTTAAACACTTAGCGCCGTTATCAAAATTCTG  733
```

```
Query  967   GCCATAAACCAGTCAGGATAGTGAGTAAGAAAAACCGGTTTGGGTTCTTTTGAAATTG  1024
              |||  ||||||||||||| ||||||| ||| | || |||||| |||||| || ||||
Sbjct  732   -CCAGTAACCAGTCAGGAT-GTGAGTAGGAAGA-CCTGTTTGG-TTCTTT-GATATTG  680
```

#######

>Cphy1800 forward

```
Query  68    AAAGAAGGAGATAGGATCATGATCGTTGCTTACTTCCCGAACTGGGGAATCTATAATAGT  127
             ||||||||||||||||||||||||||||||||||||||||||||||||||||||||||||
Sbjct  1     AAAGAAGGAGATAGGATCATGATCGTTGCTTACTTCCCGAACTGGGGAATCTATAATAGT  60

Query  128   GCCCATCGCACTATGACCGTTGGTATGATTCCATGGGACAAGGTTACAGTAATAAACCAT  187
             ||||||||||||||||||||||||||||||||||||||||||||||||||||||||||||
Sbjct  61    GCCCATCGCACTATGACCGTTGGTATGATTCCATGGGACAAGGTTACAGTAATAAACCAT  120

Query  188   GCCTTTTTGAGGTAGATTCTTCCTTTAAACTGGCATCAACAGATTCCTTCGCTGACTTT  247
             ||||||||||||||||||||||||||||||||||||||||||||||||||||||||||||
Sbjct  121   GCCTTTTTGAGGTAGATTCTTCCTTTAAACTGGCATCAACAGATTCCTTCGCTGACTTT  180

Query  248   GATAAGATGATGGATCATTCCGAAGGGTGGGATACTAATCAATTAAGAGGACATTTCGGA  307
             ||||||||||||||||||||||||||||||||||||||||||||||||||||||||||||
Sbjct  181   GATAAGATGATGGATCATTCCGAAGGGTGGGATACTAATCAATTAAGAGGACATTTCGGA  240

Query  308   GAGTATAAATATTATAAGAATCTCTATCCGAATGTAAAAGTAATCGTATCCGTTGGTGGT  367
             ||||||||||||||||||||||||||||||||||||||||||||||||||||||||||||
Sbjct  241   GAGTATAAATATTATAAGAATCTCTATCCGAATGTAAAAGTAATCGTATCCGTTGGTGGT  300

Query  368   TGGACAAGAGGTCAAAATTTCCATGCAATGGCTGCTACTACTTCCACCAGAGCGGTTTTT  427
             ||||||||||||||||||||||||||||||||||||||||||||||||||||||||||||
Sbjct  301   TGGACAAGAGGTCAAAATTTCCATGCAATGGCTGCTACTACTTCCACCAGAGCGGTTTTT  360

Query  428   ATCCAGAGTGTTATTGATCTTCTGAGAAAATATCCGTTTATCGATGGCGTTGATCTTGAT  487
             ||||||||||||||||||||||||||||||||||||||||||||||||||||||||||||
Sbjct  361   ATCCAGAGTGTTATTGATCTTCTGAGAAAATATCCGTTTATCGATGGCGTTGATCTTGAT  420

Query  488   TGGGAATATCCTGGCATTAATAGGGCACCAGATCCTAACGATTCCTATGACAGAGGCTGC  547
             ||||||||||||||||||||||||||||||||||||||||||||||||||||||||||||
Sbjct  421   TGGGAATATCCTGGCATTAATAGGGCACCAGATCCTAACGATTCCTATGACAGAGGCTGC  480

Query  548   CCAGGAGGACCGGAGGATAAGCAGAATTTCACCTCTTTACTACGTGAAATACGTCAGGCA  607
             ||||||||||||||||||||||||||||||||||||||||||||||||||||||||||||
Sbjct  481   CCAGGAGGACCGGAGGATAAGCAGAATTTCACCTCTTTACTACGTGAAATACGTCAGGCA  540

Query  608   TATAATAACAATGGCTTAAACGGTAAGTTGTTAACGATAGCTGCTCCTTCCGGTTATGAC  667
             ||||||||||||||||||||||||||||||||||||||||||||||||||||||||||||
Sbjct  541   TATAATAACAATGGCTTAAACGGTAAGTTGTTAACGATAGCTGCTCCTTCCGGTTATGAC  600

Query  668   AAGCTTGCCCTGCAGGAACCAGATATCTATGCCCAGTATCTGGACTTCATAAATGTTATG  727
             ||||||||||||||||||||||||||||||||||||||||||||||||||||||||||||
Sbjct  601   AAGCTTGCCCTGCAGGAACCAGATATCTATGCCCAGTATCTGGACTTCATAAATGTTATG  660

Query  728   ACCTACGATATGCACGGGGCATGGGAAAATACAACAAATCATCAGTCTCCACTCTATGCA  787
             ||||||||||||||||||||||||||||||||||||||||||||||||||||||||||||
Sbjct  661   ACCTACGATATGCACGGGGCATGGGAAAATACAACAAATCATCAGTCTCCACTCTATGCA  720
```

```
Query  788   AATCCCAATGACCCTTGTCCTACTTCACCTGTTGACATTAAAAACAGGTATAATACGGGA  847
             ||||| ||||||||||||||||||||||||||||||||||| |||||||||||||||| |
Sbjct  721   AATCCTAATGACCCTTGTCCTACTTCACCTGTTGACATTAAGAACAGGTATAATACGG-A  779

Query  848   CTCCGCTATGAAAACCTTACAGCAGGTATATAAAATTCCTGCCGAGAAACTGTTGGGTAG  907
             |||||||||||| |||||||||||||||||||| |||||||||||||||||||||| |||
Sbjct  780   CTCCGCTATGAAGACCTTACAGCAGGTATATAAGATTCCTGCCGAGAAACTGTTGG-TAG  838

Query  908   GTCCCCCTTATTTATTCCAAAAGAAGGAAAGGGTGTTACTgggggggAATCAACGGGGAT  967
             || ||||||||| |||||| | || ||||||| ||||||||| || |  |||||||| ||
Sbjct  839   GTTCCCCTTATT-ATTCCAGAGGATGGAAAGG-TGTTACTGGTGGAG--TCAACGGG-AT  893

Query  968   GGTAGGCAAAATGCAACTTGGAACCACCAACCGGGTACCCTGGGAAAAATCCCCCAATCC  1027
             | || ||||| ||||||| ||| | | ||||||| |||| ||||| || ||| | ||||
Sbjct  894   G-TATGCAAA-TGCAACT-GGAGC-AGCAACCGG-TACC-TGGGATAA-TCCAC-AATCA  945

Query  1028  CCCAgggggggCAA  1041
             || ||| ||| |||
Sbjct  946   CC-AGGTGGG-CAA  957

> cphy1800 reverse


Query  68    GTGTAATGGATAGTGATCCTAATGGTGATGGTGATGATGTTGTACTAAACCCCACAATGC  127
             ||||||||||||||||||||||||||||||||||||||||||||||||||||||||||||
Sbjct  1911  GTGTAATGGATAGTGATCCTAATGGTGATGGTGATGATGTTGTACTAAACCCCACAATGC  1852

Query  128   TGGTACCACATCAGGGGTCCAGCCAGCCAAAGCTGTATGTGGCTGGATACATTTATAGGT  187
             ||||||||||||||||||||||||||||||||||||||||||||||||||||||||||||
Sbjct  1851  TGGTACCACATCAGGGGTCCAGCCAGCCAAAGCTGTATGTGGCTGGATACATTTATAGGT  1792

Query  188   CTTTCCGCTATAGGTTACCAGATCACCTGCTTTATAAGCTGTTCCAATTGTCCAAGCAGT  247
             ||||||||||||||||||||||||||||||||||||||||||||||||||||||||||||
Sbjct  1791  CTTTCCGCTATAGGTTACCAGATCACCTGCTTTATAAGCTGTTCCAATTGTCCAAGCAGT  1732

Query  248   TATTTGTGTAGAGCTGATTGTATAGGTTTGTGTGACCGTAGAGGAGCTAGTCATACCGGT  307
             ||||||||||||||||||||||||||||||||||||||||||||||||||||||||||||
Sbjct  1731  TATTTGTGTAGAGCTGATTGTATAGGTTTGTGTGACCGTAGAGGAGCTAGTCATACCGGT  1672

Query  308   CATAAATGCCTTAGCCTTTATGGTTGTAGTGGAACTTACCGTAATAGGAGAAGAGTATAA  367
             ||||||||||||||||||||||||||||||||||||||||||||||||||||||||||||
Sbjct  1671  CATAAATGCCTTAGCCTTTATGGTTGTAGTGGAACTTACCGTAATAGGAGAAGAGTATAA  1612

Query  368   AGCAGAGGTAGCTGTAGGTTCCGTACCATCTGTAGTATAACGAATTTCCGCTCCTTGGGT  427
             ||||||||||||||||||||||||||||||||||||||||||||||||||||||||||||
Sbjct  1611  AGCAGAGGTAGCTGTAGGTTCCGTACCATCTGTAGTATAACGAATTTCCGCTCCTTGGGT  1552

Query  428   TGCACAGGTAATGGTTACCGTCTGAGCACTTGAATAAGTTCCTCCCGCTGGGCTAAATAC  487
             ||||||||||||||||||||||||||||||||||||||||||||||||||||||||||||
Sbjct  1551  TGCACAGGTAATGGTTACCGTCTGAGCACTTGAATAAGTTCCTCCCGCTGGGCTAAATAC  1492

Query  488   CGGTGTTGCTACTGTCTGGGTATTACCTATTGTATAAACAACCGTTGTTGTTAACGAGCT  547
             ||||||||||||||||||||||||||||||||||||||||||||||||||||||||||||
Sbjct  1491  CGGTGTTGCTACTGTCTGGGTATTACCTATTGTATAAACAACCGTTGTTGTTAACGAGCT  1432

Query  548   GTTCATACCACTCTTAAATGCTTTTGCTTTTAAGGTTGTTGTAGTATTTACTGTAAGAGC  607
             ||||||||||||||||||||||||||||||||||||||||||||||||||||||||||||
Sbjct  1431  GTTCATACCACTCTTAAATGCTTTTGCTTTTAAGGTTGTTGTAGTATTTACTGTAAGAGC  1372
```

```
Query  608   AGAAGAGTAAAGAGCAGAAGAAGCCGTAGGTTCGGTACCGTCTTTAGTATAACGAATTTC  667
             ||||||||||||||||||||||||||||||||||||||||||||||||||||||||||||
Sbjct  1371  AGAAGAGTAAAGAGCAGAAGAAGCCGTAGGTTCGGTACCGTCTTTAGTATAACGAATTTC  1312

Query  668   AGCTCCGGAAGTTGCACAGGTAATAGTCACTGTCTGAGCATTAGTGTAAGTACCACCTGC  727
             ||||||||||||||||||||||||||||||||||||||||||||||||||||||||||||
Sbjct  1311  AGCTCCGGAAGTTGCACAGGTAATAGTCACTGTCTGAGCATTAGTGTAAGTACCACCTGC  1252

Query  728   CGGACTAAATACTGGTGTTGCTACTGTCTGTGTTGTACTACCAATTAATTTCTGGAAAGC  787
             ||||||||||||||||||||||||||||||||||||||||||||||||||||||||||||
Sbjct  1251  CGGACTAAATACTGGTGTTGCTACTGTCTGTGTTGTACTACCAATTAATTTCTGGAAAGC  1192

Query  788   AAGTGTTGTTAATTCAAAATTACTGGTATCCCCTGAGATTTCCCATACAATTAAACCGCC  847
             ||||||||||||||||||||||||||||||||||||||||||||||||||||||||||||
Sbjct  1191  AAGTGTTGTTAATTCAAAATTACTGGTATCCCCTGAGATTTCCCATACAATTAAACCGCC  1132

Query  848   ATATCCGTTACTGTTAATATAATCACATCTTGCACTTAAGGAAGTGGTATCTTCATAGCT  907
             ||||||||||||||||||||||||||||||||||||||||||||||||||||||||||||
Sbjct  1131  ATATCCGTTACTGTTAATATAATCACATCTTGCACTTAAGGAAGTGGTATCTTCATAGCT  1072

Query  908   AAATACAATTTCCCTGAGATGCGTTATATAACCCAAGGCGTCCTTTTGC-TAGGTGTCGT  966
             || ||||||| |||||||||||||||||||||| |||||||| ||| || ||||||||||
Sbjct  1071  AAGTACAATT-CCCTGAGATGCGTTATATAACC-AAGGCGTC-TTT-GCATAGGTGTCGT  1016

Query  967   CCCCAATATTTCACAAAATCCTTTTTGGTTTCTCCATGGTCCTTTAAGGGGAAATAAAGG  1026
             | | | ||||||||||| ||| |||||||| |||||||||| ||||||| ||||||| ||
Sbjct  1015  CGCGA-TATTTCACAAA-TCCATTTTGGTT-CTCCATGGTC-TTTAAGGTGAAATAA-GG  961

Query  1027  AAAATTGCCCACCCTGGTGAATTGTGGGAATATCCCAGG  1065
             | | ||||||||| |||||| |||||| | |||||||||
Sbjct  960   ATA-TTGCCCACC-TGGTGA-TTGTGG-ATTATCCCAGG  926
```

###

cphy0601 forward

Query= B01_601C2P16_1.SCF

>cphy_0601

```
Query: 71  aaagaaggagataggatcATGcaatttgagattgataaagacaaattggagaaatggtgt 130
           ||||||||||||||||||||||||||||||||||||||||||||||||||||||||||||
Sbjct: 1   aaagaaggagataggatcatgcaatttgagattgataaagacaaattggagaaatggtgt 60


Query: 131 aaaactggaattttagtactttccatcatcttagttatagcggtaggagtttattttgtt 190
           ||||||||||||||||||||||||||||||||||||||||||||||||||||||||||||
Sbjct: 61  aaaactggaattttagtactttccatcatcttagttatagcggtaggagtttattttgtt 120


Query: 191 ccaaagattattgcgaaagcagcaggagagaaacgagaactaccgatttattgtgtacag 250
           ||||||||||||||||||||||||||||||||||||||||||||||||||||||||||||
Sbjct: 121 ccaaagattattgcgaaagcagcaggagagaaacgagaactaccgatttattgtgtacag 180


Query: 251 acagagaaaaaacaagttgcattatcctttgatgcagcctgggggaatgaagatactgca 310
```

```
          ||||||||||||||||||||||||||||||||||||||||||||||||||||||||||||
Sbjct: 181 acagagaaaaaacaagttgcattatcctttgatgcagcctgggggaatgaagatactgca 240


Query: 311 cggatactagaaatattaaagaaacaaaatgttaatgtcacatttttatgaccggtggc 370
           ||||||||||||||||||||||||||||||||||||||||||||||||||||||||||||
Sbjct: 241 cggatactagaaatattaaagaaacaaaatgttaatgtcacatttttatgaccggtggc 300


Query: 371 tgggttgaaaaataccctgatgatgtaaaagcaatagcggcagccggtcatgatttggga 430
           ||||||||||||||||||||||||||||||||||||||||||||||||||||||||||||
Sbjct: 301 tgggttgaaaaataccctgatgatgtaaaagcaatagcggcagccggtcatgatttggga 360


Query: 431 aaccatagtgaaaaccataagcagatgtcaaagctttcaaaagagcagtcggtaaaggaa 490
           ||||||||||||||||||||||||||||||||||||||||||||||||||||||||||||
Sbjct: 361 aaccatagtgaaaaccataagcagatgtcaaagctttcaaaagagcagtcggtaaaggaa 420


Query: 491 ataatgtcaccgcatgataaggttaaagcacttactggtaaggatatgcaactttttaga 550
           ||||||||||||||||||||||||||||||||||||||||||||||||||||||||||||
Sbjct: 421 ataatgtcaccgcatgataaggttaaagcacttactggtaaggatatgcaactttttaga 480


Query: 551 ccacctttcggtgactataataataccttgattcaagcaacaaaagaatgtgggtattac 610
           ||||||||||||||||||||||||||||||||||||||||||||||||||||||||||||
Sbjct: 481 ccacctttcggtgactataataataccttgattcaagcaacaaaagaatgtgggtattac 540


Query: 611 tgtattcaatgggatgtggattccctagattggaaagactacggaaaagatagcattctt 670
           ||||||||||||||||||||||||||||||||||||||||||||||||||||||||||||
Sbjct: 541 tgtattcaatgggatgtggattccctagattggaaagactacggaaaagatagcattctt 600


Query: 671 aaacaagtcttaaataataaacatctaggaaatggttccatttatactttgccataatgg 730
           |||||||||||||||||||||||||||||||||||||||| |||||||||||||||||||
Sbjct: 601 aaacaagtcttaaataataaacatctaggaaatggttcca-ttatactttgccataatgg 659


Query: 731 tgcctaatttaccgcagacccattggaagaattaattgtagggttaaaagataaaggtta 790
           |||  |||||||||||||| ||||||||||||||||||||||||||||||||||||||||
Sbjct: 660 tgctaaatttaccgcagacgcattggaagaattaattgtagggttaaaagataaaggtta 719


Query: 791 tgaaatcgtgcctatttctaaattgatctacacagagaattttgaaatggattcttgagg 850
           ||||||||||||||||||||| ||||||||||||||||||||||| ||||| || ||| |
Sbjct: 720 tgaaatcgtgcctatttctaagttgatctacacagagaattttgagatgga-tcatga-g 777


Query: 851 ggaagaaaggtacaaaagcttcatccccatca-cattaagaattactatccatt 903
           |||||| ||||||| |||| ||||| |||||| ||||||| || ||||||||||
Sbjct: 778 ggaagacaggtacagaagcatcatcaccatcaccattaag-atcactatccatt 830

=================================================================

Cphy0601 reverse

Query= HR0ADKBY98ZB01_601C2P17_1.SCF
```

```
>cphy_0601

Query: 68  gtgtaatggatagtgatcTTAatggtgatggtgatgatgcttctgtacctgtcttccctc 127
           ||||||||||||||||||||||||||||||||||||||||||||||||||||||||||||
Sbjct: 834 gtgtaatggatagtgatcttaatggtgatggtgatgatgcttctgtacctgtcttccctc 775

Query: 128 atgatccatctcaaaattctctgtgtagatcaacttagaaataggcacgatttcataacc 187
           ||||||||||||||||||||||||||||||||||||||||||||||||||||||||||||
Sbjct: 774 atgatccatctcaaaattctctgtgtagatcaacttagaaataggcacgatttcataacc 715

Query: 188 tttatcttttaaccctacaattaattcttccaatgcgtctgcggtaaatttagcaccatt 247
           ||||||||||||||||||||||||||||||||||||||||||||||||||||||||||||
Sbjct: 714 tttatcttttaaccctacaattaattcttccaatgcgtctgcggtaaatttagcaccatt 655

Query: 248 atggcaaagtataatggaaccatttcctagatgtttattatttaagacttgtttaagaat 307
           ||||||||||||||||||||||||||||||||||||||||||||||||||||||||||||
Sbjct: 654 atggcaaagtataatggaaccatttcctagatgtttattatttaagacttgtttaagaat 595

Query: 308 gctatcttttccgtagtctttccaatctagggaatccacatcccattgaatacagtaata 367
           ||||||||||||||||||||||||||||||||||||||||||||||||||||||||||||
Sbjct: 594 gctatcttttccgtagtctttccaatctagggaatccacatcccattgaatacagtaata 535

Query: 368 cccacattcttttgttgcttgaatcaaggtattattatagtcaccgaaaggtggtctaaa 427
           ||||||||||||||||||||||||||||||||||||||||||||||||||||||||||||
Sbjct: 534 cccacattcttttgttgcttgaatcaaggtattattatagtcaccgaaaggtggtctaaa 475

Query: 428 aagttgcatatccttaccagtaagtgctttaaccttatcatgcggtgacattatttcctt 487
           ||||||||||||||||||||||||||||||||||||||||||||||||||||||||||||
Sbjct: 474 aagttgcatatccttaccagtaagtgctttaaccttatcatgcggtgacattatttcctt 415

Query: 488 taccgactgctcttttgaaagctttgacatctgcttatggttttcactatggtttcccaa 547
           ||||||||||||||||||||||||||||||||||||||||||||||||||||||||||||
Sbjct: 414 taccgactgctcttttgaaagctttgacatctgcttatggttttcactatggtttcccaa 355

Query: 548 atcatgaccggctgccgctattgcttttacatcatcagggtatttttcaacccagccacc 607
           ||||||||||||||||||||||||||||||||||||||||||||||||||||||||||||
Sbjct: 354 atcatgaccggctgccgctattgcttttacatcatcagggtatttttcaacccagccacc 295

Query: 608 ggtcataaaaaatgtgacattaacattttgtttctttaatatttctagtatccgtgcagt 667
           ||||||||||||||||||||||||||||||||||||||||||||||||||||||||||||
Sbjct: 294 ggtcataaaaaatgtgacattaacattttgtttctttaatatttctagtatccgtgcagt 235

Query: 668 atcttcattcccccaggctgcatcaaaggataatgcaactttgtttttctctgtctgta 727
           ||||||||||||||||||||||||||||||||||||||| ||||||||||||||||||||
Sbjct: 234 atcttcattcccccaggctgcatcaaaggataatgcaac-ttgtttttctctgtctgta 176
```

```
Query: 728 cacaataaatcggtagttctcgttttctctcctgctgctttcgcaataatctttggnnnn 787
           |||||||||||||||||||||| |||||||||||||||||||||||||||||||||
Sbjct: 175 cacaataaatcggtagttctcg-tttctctcctgctgctttcgcaataatctttggaaca 117


Query: 788 nnntaaactcctaccgctataactaaaatgatggaaagtactaaaatttccagttttaca 847
              ||||||||||||||||||||||| ||||||||||||||||||| |||||||||||||
Sbjct: 116 aaataaactcctaccgctataactaagatgatggaaagtactaaaa-ttccagttttaca 58


Query: 848 ccttttctccaatttgtctttaacaatcccaaattgc 885
            || ||||||||||||||||||| ||||| ||||||||
Sbjct: 57  -ccatttctccaatttgtctttatcaatctcaaattgc 21
```

==

Cphy0666 forward

Query= HR0ADKBY98ZE01_666C2P16_1.SCF   1

>cphy_0666

```
Query: 70  aaagaaggagataggatcATGaaatcatttataaaagaattaattgaacagaaggaaggt 129
           ||||||||||||||||||||||||||||||||||||||||||||||||||||||||||||
Sbjct: 1   aaagaaggagataggatcatgaaatcatttataaaagaattaattgaacagaaggaaggt 60


Query: 130 aattatattttacctttttctggcagcatggagagagtgaagggaagcttcgggagtat 189
           ||||||||||||||||||||||||||||||||||||||||||||||||||||||||||||
Sbjct: 61  aattatattttacctttttctggcagcatggagagagtgaagggaagcttcgggagtat 120


Query: 190 atggaggcaatccatagctgcggtattcgtgcagtctgtgttgaggccagaccccatccg 249
           ||||||||||||||||||||||||||||||||||||||||||||||||||||||||||||
Sbjct: 121 atggaggcaatccatagctgcggtattcgtgcagtctgtgttgaggccagaccccatccg 180


Query: 250 gattatcttggaccgggctggtggcatgatttggatatcattattgatgaagctaagaaa 309
           ||||||||||||||||||||||||||||||||||||||||||||||||||||||||||||
Sbjct: 181 gattatcttggaccgggctggtggcatgatttggatatcattattgatgaagctaagaaa 240


Query: 310 agggatatgaagttatggattctggatgatgcacattttcctaccggctatgccgctggg 369
           ||||||||||||||||||||||||||||||||||||||||||||||||||||||||||||
Sbjct: 241 agggatatgaagttatggattctggatgatgcacattttcctaccggctatgccgctggg 300


Query: 370 aaaatcgccgagtcagaagataaatactgtaagcagtatattagccggaagtttatcgat 429
           ||||||||||||||||||||||||||||||||||||||||||||||||||||||||||||
Sbjct: 301 aaaatcgccgagtcagaagataaatactgtaagcagtatattagccggaagtttatcgat 360


Query: 430 gtcagcggtcctacacctgaggttgaggttaatcttgccgctatgatgatgccgatcatg 489
           ||||||||||||||||||||||||||||||||||||||||||||||||||||||||||||
Sbjct: 361 gtcagcggtcctacacctgaggttgaggttaatcttgccgctatgatgatgccgatcatg 420
```

```
Query: 490 ctaccctccggattaaatcttgaaatgtttggtatgagctcggagaagaagcgctatttt 549
           ||||||||||||||||||||||||||||||||||||||||||||||||||||||||||||
Sbjct: 421 ctaccctccggattaaatcttgaaatgtttggtatgagctcggagaagaagcgctatttt 480


Query: 550 acagatgacagaatcttgtccatccgtgcatatcagatggataaggactcctccctgata 609
           ||||||||||||||||||||||||||||||||||||||||||||||||||||||||||||
Sbjct: 481 acagatgacagaatcttgtccatccgtgcatatcagatggataaggactcctccctgata 540


Query: 610 ggagaaggaatcgatctttaccacgaaaattgtgaatggaaaattaatatgggacgtttc 669
           |||||||||||||||| |||||||||| |||||||||||||||||||||||||||| |||
Sbjct: 541 ggagaaggaatcgatc-ttaccacgaacattgtgaatggaaaattaatatgggacg-ttc 598


Query: 670 ccaagggtacctggaaaattaatgctctctttattactagaaatggtgaggggcgctgcg 729
           ||||||||||||||| |||||||| |||||||||||||||||||||||||||||||||||
Sbjct: 599 ccaagggtacctggagaattaatgttctctttattactagaaatggtgaggggcgctgcg 658


Query: 730 actaaatcataaggggggataaggattccggtaaaacccgaatagatgcggtgtatgaac 789
           |||| ||||  | || ||||||||||||| ||| || ||  |||||||||||||||||||
Sbjct: 659 actatatcaatatggtggataaggattcctgtagaatccagatagatgcggtgtatgaac 718


Query: 790 ctcattatgagcttttacaaagaagaattttggaaaaatgattgcaggcttccttttcgg 849
           ||||||||||||  ||||||||||||| ||||| |||| |||||||||||| ||||||||
Sbjct: 719 ctcattatgagc-attacaaagaagaa-tttgggaaaacgattgcaggctt-cttttcgg 775


Query: 850 a 850
           |
Sbjct: 776 a 776
```

==

Cphy0666 reverse

Query= HR0ADKBY98ZE01_666C2P17_1.SCF

>cphy_0666

```
Query: 66   gtgtaatggatagtgatcTTAatggtgatggtgatgatgattccagatttccacactacc 125
            ||||||||||||||||||||||||||||||||||||||||||||||||||||||||||||
Sbjct: 2748 gtgtaatggatagtgatcttaatggtgatggtgatgatgattccagatttccacactacc 2689


Query: 126  tacgatacccgtcggctctgttatctttgaatagaacatcagggaacgcatatcctgggg 185
            ||||||||||||||||||||||||||||||||||||||||||||||||||||||||||||
Sbjct: 2688 tacgatacccgtcggctctgttatctttgaatagaacatcagggaacgcatatcctgggg 2629


Query: 186  aagggattttaccttccggtccagggttgtggcaacctcaatccttatatggttctctcc 245
            ||||||||||||||||||||||||||||||||||||||||||||||||||||||||||||
Sbjct: 2628 aagggattttaccttccggtccagggttgtggcaacctcaatccttatatggttctctcc 2569
```

```
Query: 246  ggctcttaatgcttctcccaaaggaaactgatatggcggacagattttcattccaagaca 305
            ||||||||||||||||||||||||||||||||||||||||||||||||||||||||||||
Sbjct: 2568 ggctcttaatgcttctcccaaaggaaactgatatggcggacagattttcattccaagaca 2509


Query: 306  gtggccattcagccaaacttcagcgccctcatagacatctgtaatattaagtagctccct 365
            ||||||||||||||||||||||||||||||||||||||||||||||||||||||||||||
Sbjct: 2508 gtggccattcagccaaacttcagcgccctcatagacatctgtaatattaagtagctccct 2449


Query: 366  gccggttggcattctatcatagacaaactcactttcataacgtaaatagccggaaaaatc 425
            ||||||||||||||||||||||||||||||||||||||||||||||||||||||||||||
Sbjct: 2448 gccggttggcattctatcatagacaaactcactttcataacgtaaatagccggaaaaatc 2389


Query: 426  aggtaatacccggcccatgcttttaactccgtcgtaacttctacaggttgaaactcagg 485
            ||||||||||||||||||||||||||||||||||||||||||||||||||||||||||||
Sbjct: 2388 aggtaatacccggcccatgcttttaactccgtcgtaacttctacaggttgaaactcagg 2329


Query: 486  atatttactgacttcggcactggagatgcgccatgctgcgtccagtctgtatccaggctt 545
            ||||||||||||||||||||||||||||||||||||||||||||||||||||||||||||
Sbjct: 2328 atatttactgacttcggcactggagatgcgccatgctgcgtccagtctgtatccaggctt 2269


Query: 546  agcctcgaaattgccttggtcttccacctccttcagatcctcaaacagagcaaccacact 605
            ||||||||||||||||||||||||||||||||||||||||||||||||||||||||||||
Sbjct: 2268 agcctcgaaattgccttggtcttccacctccttcagatcctcaaacagagcaaccacact 2209


Query: 606  ttgatagggtgttaatctaatatgtatgcttgttccgtcttcggtatttctatattctgc 665
            ||||||||||||||||||||||||||||||||||||||||||||||||||||||||||||
Sbjct: 2208 ttgatagggtgttaatctaatatgtatgcttgttccgtcttcggtatttctatattctgc 2149


Query: 666  cggaattatcctgttgtccctagcatcatataagctaacctcaccttttgatttcccggt 725
            ||||||||||||||||||| ||||||||||||||||||||||||||||||||||| ||||
Sbjct: 2148 cggaattatcctgttgtccatagcatcatataagctaacctcaccttttgatttcacggt 2089


Query: 726  tatccagccattgtattccttaccgaaactttcattggaaaacatatatagctcctggtc 785
            |||||||||||||||||||||||||| |||||||||||| ||||||||||||||   |||
Sbjct: 2088 tatccagccattgtattccttaccgatactttcattggagaacatatatagctc--tgtc 2031


Query: 786  tgctccttacggtaatggtaataacccaactccatcaaaatggattgtcactgcctgatt 845
            ||||||||||||||||||||||||| || | ||||| |||| ||||||||||| |||| |
Sbjct: 2030 tgctccttacggtaatggtaataacgcagc-ccatc-aaattgattgtcactg-ctga-t 1975


Query: 846  ttcaaataatttg 858
            |||||||||||||
Sbjct: 1974 ttcaaataatttg 1962
```

###

Cphy0769 forward

Query= G01_769C1P16_1.SCF

>cphy_0769

```
Query: 72  aaagaaggagataggatcATGattaaaattacgtttatgggtgcgggaagtaccatattt 131
           ||||||||||||||||||||||||||||||||||||||||||||||||||||||||||||
Sbjct: 1   aaagaaggagataggatcatgattaaaattacgtttatgggtgcgggaagtaccatattt 60


Query: 132 gcaaggaatgtattaggagattgtatgtgcaccgaagcgctttgtgaaagcgagatagcc 191
           ||||||||||||||||||||||||||||||||||||||||||||||||||||||||||||
Sbjct: 61  gcaaggaatgtattaggagattgtatgtgcaccgaagcgctttgtgaaagcgagatagcc 120


Query: 192 ttatatgacattgatgctgaacggctttcagattctaaaataatattggacgcaatcaac 251
           ||||||||||||||||||||||||||||||||||||||||||||||||||||||||||||
Sbjct: 121 ttatatgacattgatgctgaacggctttcagattctaaaataatattggacgcaatcaac 180


Query: 252 aaaaatgtaaacgagggaaaggctgttataaaaacttatttaggagtggaaaatagaaag 311
           ||||||||||||||||||||||||||||||||||||||||||||||||||||||||||||
Sbjct: 181 aaaaatgtaaacgagggaaaggctgttataaaaacttatttaggagtggaaaatagaaag 240


Query: 312 gctgctttaaagggtgcaagctttgtggtcaatgccatccaggtaggcggttacgatcct 371
           ||||||||||||||||||||||||||||||||||||||||||||||||||||||||||||
Sbjct: 241 gctgctttaaagggtgcaagctttgtggtcaatgccatccaggtaggcggttacgatcct 300


Query: 372 agtactattatagattttgagataccgaagaaattcggtctacgccagaccattgctgac 431
           ||||||||||||||||||||||||||||||||||||||||||||||||||||||||||||
Sbjct: 301 agtactattatagattttgagataccgaagaaattcggtctacgccagaccattgctgac 360


Query: 432 acactaggcatcggcggcattatgagaggactacgcacaattccagtcattcaggaattt 491
           ||||||||||||||||||||||||||||||||||||||||||||||||||||||||||||
Sbjct: 361 acactaggcatcggcggcattatgagaggactacgcacaattccagtcattcaggaattt 420


Query: 492 gctgcagatatggaggagatgtgcccagacgccctattcctcaattatacgaatcccatg 551
           ||||||||||||||||||||||||||||||||||||||||||||||||||||||||||||
Sbjct: 421 gctgcagatatggaggagatgtgcccagacgccctattcctcaattatacgaatcccatg 480


Query: 552 gccattctgactggctatatgcagcgttataccaatatccgtacggtgggcctctgccac 611
           ||||||||||||||||||||||||||||||||||||||||||||||||||||||||||||
Sbjct: 481 gccattctgactggctatatgcagcgttataccaatatccgtacggtgggcctctgccac 540


Query: 612 agtgtccaggtgtgttcagagcatctcttaaaggacccttggcatggatgataaagctgg 671
           ||||||||||||||||||||||||||||||||||| |||||||||||||||| |||||||
Sbjct: 541 agtgtccaggtgtgttcagagcatctcttaaagga-ccttggcatggatgat-aagctgg 598


Query: 672 agggccgtaaggagctgattgctggaatttaaccatattgggatggttggtaaaagatac 731
```

```
           ||||||||||||||||||||||||||| ||||||||| |||||||||| || ||||||||
Sbjct: 599 agggccgtaaggagctgattgctggaa-ttaaccata-tgggatggtt-gttaaagatac 655


Query: 732 agggaaagatatggtaatgacctgtatccctgaaatttaataaaagagctgttgtcaag 791
           | ||| ||||||||||||||||||||| |||||||  |||| ||||||||||||||||||
Sbjct: 656 a-ggatagatatggtaatgacctgtat-cctgaaa--ttaaaaaaagagctgttgtcaag 711


Query: 792 aactctaactttccccacataaaagaattggttaaggtatgaatacatcaacccagtttg 851
           |||||||   |||||||||| |||||  ||||| ||||||||||||||||| ||||||||
Sbjct: 712 aactcta--cttccccacat-aaagatatggtt-aggtatgaatacatcaa-ccagtttg 766


Query: 852 gatatt 857
           ||||||
Sbjct: 767 gatatt 772
```

===

Cphy0769 reverse

Query= G07_769C1P17_1.SCF

>cphy_0769

```
Query: 67   gtgtaatggatagtgatcTTAatggtgatggtgatgatgttggaattccgccatgtaatc 126
             ||||||||||||||||||||||||||||||||||||||||||||||||||||||||||||
Sbjct: 1377 gtgtaatggatagtgatcttaatggtgatggtgatgatgttggaattccgccatgtaatc 1318


Query: 127  cccatgagctgcaattagctcatcacacatacagcggatatcagagatattcaattctgc 186
             ||||||||||||||||||||||||||||||||||||||||||||||||||||||||||||
Sbjct: 1317 cccatgagctgcaattagctcatcacacatacagcggatatcagagatattcaattctgc 1258


Query: 187  agctgtgtgaggatctagcatggcagcttgataaatatggctacgatctttggttactgc 246
             ||||||||||||||||||||||||||||||||||||||||||||||||||||||||||||
Sbjct: 1257 agctgtgtgaggatctagcatggcagcttgataaatatggctacgatctttggttactgc 1198


Query: 247  tgcttctatagtcaaaagctggacattaatattggtcatgttcatagcagccagttgaac 306
             ||||||||||||||||||||||||||||||||||||||||||||||||||||||||||||
Sbjct: 1197 tgcttctatagtcaaaagctggacattaatattggtcatgttcatagcagccagttgaac 1138


Query: 307  aggtaggcgacccacatggcatggcgtgattccattaccatccacaagacaaggaacctc 366
             ||||||||||||||||||||||||||||||||||||||||||||||||||||||||||||
Sbjct: 1137 aggtaggcgacccacatggcatggcgtgattccattaccatccacaagacaaggaacctc 1078


Query: 367  cacgcaggcatctgagggaagattgtcaattaaaccgttgtttaacacattgcctccgat 426
             ||||||||||||||||||||||||||||||||||||||||||||||||||||||||||||
Sbjct: 1077 cacgcaggcatctgagggaagattgtcaattaaaccgttgtttaacacattgcctccgat 1018


Query: 427  gacataaggtttgttggtgacaattgcttccatgatataggaggcatattcagcagatcg 486
```

```
            ||||||||||||||||||||||||||||||||||||||||||||||||||||||||||||
Sbjct: 1017 gacataaggtttgttggtgacaattgcttccatgatataggaggcatattcagcagatcg 958


Query: 487  tgtatgggtaatctgaccattttgcaggattccgaccttttctgcctcccattctgcaat 546
            ||||||||||||||||||||||||||||||||||||||||| ||||||||||||||||||
Sbjct: 957  tgtatgggtaatctgaccattttgcaggattccgaccttttcagcctcccattctgcaat 898


Query: 547  ctgcttgatgcagcgacgaggatattcatccaggggaatgccatacccgtcaatcatctc 606
            ||||||||||||||||||||||||||||||||||||||||||||||||||||||||||||
Sbjct: 897  ctgcttgatgcagcgacgaggatattcatccaggggaatgccatacccgtcaatcatctc 838


Query: 607  aggatatctgtttttgataaaaagcggattatactccgcattgtgttcgcttgattcggt 666
            ||||||||||||||||||||||||||||||||||||||||||||||||||||||||||||
Sbjct: 837  aggatatctgtttttgataaaaagcggattatactccgcattgtgttcgcttgattcggt 778


Query: 667  gcaattatatccaaactgggatggagtatttcatacctaaccatatcttgatgtggggaa 726
            ||| | ||||||||||| || ||  ||| |||||||||||||||||||| ||||||||||
Sbjct: 777  gcagtaatatccaaact-ggttgatgta-ttcatacctaaccatatctttatgtggggaa 720


Query: 727  gtaaagttcttgacaacagctc 748
            ||| ||||||||||||||||||
Sbjct: 719  gtagagttcttgacaacagctc 698


===========================================================================

Cphy0776 forward

Query= D02_776C3P16_1.SCF

>cphy_0776


Query: 70  aaagaaggagataggatcATGaaggaagcagaattaattcagaaaatagaagcaaaaatg 129
           ||||||||||||||||||||||||||||||||||||||||||||||||||||||||||||
Sbjct: 1   aaagaaggagataggatcatgaaggaagcagaattaattcagaaaatagaagcaaaaatg 60


Query: 130 gtttttatgctggaacatttgggagactgtgtgccatggtatggtgaggacggcgtatac 189
           ||||||||||||||||||||||||||||||||||||||||||||||||||||||||||||
Sbjct: 61  gtttttatgctggaacatttgggagactgtgtgccatggtatggtgaggacggcgtatac 120


Query: 190 catggatggcaagaggattcctgggtcagtggcttttggccagggatgctgtggattttg 249
           ||||||||||||||||||||||||||||||||||||||||||||||||||||||||||||
Sbjct: 121 catggatggcaagaggattcctgggtcagtggcttttggccagggatgctgtggattttg 180


Query: 250 tatgacttaacaggaagggagagttataagcaagcggcctgggagtgggatggcaggatc 309
           ||||||||||||||||||||||||||||||||||||||||||||||||||||||||||||
Sbjct: 181 tatgacttaacaggaagggagagttataagcaagcggcctgggagtgggatggcaggatc 240
```

```
Query: 310 gagcagtgctatttaagaaattctaaccttcatcacgatgtggggttccagtttctacct 369
           ||||||||||||||||||||||||||||||||||||||||||||||||||||||||||||
Sbjct: 241 gagcagtgctatttaagaaattctaaccttcatcacgatgtggggttccagtttctacct 300


Query: 370 acggcggtgatgaagtataagctgaccggagatgaggatggaagacgcaggggactttttt 429
           ||||||||||||||||||||||||||||||||||||||||||||||||||||||||||||
Sbjct: 301 acggcggtgatgaagtataagctgaccggagatgaggatggaagacgcaggggactttttt 360


Query: 430 gcggccaattttctggcaggtcgctttaacctagcaggaagttttatccgggcgtggaat 489
           ||||||||||||||||||||||||||||||||||||||||||||||||||||||||||||
Sbjct: 361 gcggccaattttctggcaggtcgctttaacctagcaggaagttttatccgggcgtggaat 420


Query: 490 ttagataagattggatggtctatcattgattctaccatgaatttatcccttctattctgg 549
           ||||||||||||||||||||||||||||||||||||||||||||||||||||||||||||
Sbjct: 421 ttagataagattggatggtctatcattgattctaccatgaatttatcccttctattctgg 480


Query: 550 gcgtccaatgagacaaaagatcctaggttccgacaaattggcgtggcacaagcggagaca 609
           ||||||||||||||||||||||||||||||||||||||||||||||||||||||||||||
Sbjct: 481 gcgtccaatgagacaaaagatcctaggttccgacaaattggcgtggcacaagcggagaca 540


Query: 610 gttttaaagcactttatcaggccggatggatctgtttgtcacatactttcttttgacccc 669
           ||||||||||||||||||||||||||||||||||||||||||||||||||||||||||||
Sbjct: 541 gttttaaagcactttatcaggccggatggatctgtttgtcacatactttcttttgacccc 600


Query: 670 gagacaggagcattccttgagccattgggaggccagggatacgataaagattcttcatgg 729
           ||||||||||||||||||||||||||||||||||||||||||||||||||||||||||||
Sbjct: 601 gagacaggagcattccttgagccattgggaggccagggatacgataaagattcttcatgg 660


Query: 730 agccgggggcaggcttgggcaatatatgggatgggctatgtataccgttatacaggggat 789
           ||||||||||||||||||||||||||||||||||   |||||||||||||||||||||||
Sbjct: 661 agccgggggcaggcttgggcaatatatgggatggctaatgtataccgttatacaggggat 720


Query: 790 agacgattttttagatgccgcgaagcgagtcgcccattattttctgggcaaatctaaaag 849
           |||||| |||||||||||||||||||||||||||||||||||||| ||||||||| | ||
Sbjct: 721 agacga-tttttagatgccgcgaagcgagtcgcccattattttct-ggcaaatct-agag 777


Query: 850 gaaacgggaattccttgttgggattttcgtgttccac 886
           |||||||||||||||||||||||||||||||||||||
Sbjct: 778 gaaacgggaattccttgttgggattttcgtgttccac 814



========================================================================

Cphy0776 reverse


Query= D08_776C3P17_1.SCF
```

>cphy_0776

```
Query: 69   gtgtaatggatagtgatcTTAatggtgatggtgatgatgaaatactctgtggttccagcc 128
            ||||||||||||||||||||||||||||||||||||||||||||||||||||||||||||
Sbjct: 1149 gtgtaatggatagtgatcttaatggtgatggtgatgatgaaatactctgtggttccagcc 1090


Query: 129  gtgaagcttagctagtgcctccaaataaaaatagtctccatatattagtgacacatccac 188
            ||||||||||||||||||||||||||||||||||||||||||||||||||||||||||||
Sbjct: 1089 gtgaagcttagctagtgcctccaaataaaaatagtctccatatattagtgacacatccac 1030


Query: 189  ttgtgctcccttgggtttctctccggttccatgctttaaaaggccctggtactctacttc 248
            ||||||||||||||||||||||||||||||||||||||||||||||||||||||||||||
Sbjct: 1029 ttgtgctcccttgggtttctctccggttccatgctttaaaaggccctggtactctacttc 970


Query: 249  ttctaaagcaccgcaatgctctgtaaggctttcaagcaaacgcatagctgcctttcggta 308
            ||||||||||||||||||||||||||||||||||||||||||||||||||||||||||||
Sbjct: 969  ttctaaagcaccgcaatgctctgtaaggctttcaagcaaacgcatagctgcctttcggta 910


Query: 309  aaagctcccatcccactcgtctacttccgcaagttccaaaaggcctgaggcagcaatggc 368
            ||||||||||||||||||||||||||||||||||||||||||||||||||||||||||||
Sbjct: 909  aaagctcccatcccactcgtctacttccgcaagttccaaaaggcctgaggcagcaatggc 850


Query: 369  tcccgccgaggaatcccaaggttctttttctctgtgtggaacacgaaaatcccaacaagg 428
            ||||||||||||||||||||||||||||||||||||||||||||||||||||||||||||
Sbjct: 849  tcccgccgaggaatcccaaggttctttttctctgtgtggaacacgaaaatcccaacaagg 790


Query: 429  aattcccgtttcctctagatttgccagaaaataatgggcgactcgcttcgcggcatctaa 488
            ||||||||||||||||||||||||||||||||||||||||||||||||||||||||||||
Sbjct: 789  aattcccgtttcctctagatttgccagaaaataatgggcgactcgcttcgcggcatctaa 730


Query: 489  aaatcgtctatcccctgtataacggtatacattagccatcccatatattgcccaagcctg 548
            ||||||||||||||||||||||||||||||||||||||||||||||||||||||||||||
Sbjct: 729  aaatcgtctatcccctgtataacggtatacattagccatcccatatattgcccaagcctg 670


Query: 549  cccccggctccatgaagaatctttatcgtatccctggcctcccaatggctcaaggaatgc 608
            ||||||||||||||||||||||||||||||||||||||||||||||||||||||||||||
Sbjct: 669  cccccggctccatgaagaatctttatcgtatccctggcctcccaatggctcaaggaatgc 610


Query: 609  tcctgtctcggggtcaaaagaaagtatgtgacaaacagatccatccggcctgataaagtg 668
            ||||||||||||||||||||||||||||||||||||||||||||||||||||||||||||
Sbjct: 609  tcctgtctcggggtcaaaagaaagtatgtgacaaacagatccatccggcctgataaagtg 550


Query: 669  ctttaaaactgtctccgcttgtgccacgccaatttgtcggaacctaggatcttttgtctc 728
            ||||||||||||||||||||||||||||||||||||||||||||||||||||||||||||
Sbjct: 549  ctttaaaactgtctccgcttgtgccacgccaatttgtcggaacctaggatcttttgtctc 490
```

```
Query: 729  attggacgcccagaatagaagggataaattcatggtaaaatcaatgatagaccatccaat 788
            ||||||||||||||||||||||||||||||||||||| ||||||||||||||||||||||
Sbjct: 489  attggacgcccagaatagaagggataaattcatggtagaatcaatgatagaccatccaat 430


Query: 789  cttatctaaattccacgcccggataaaacttcctgctaagttaaagcgaactgccagaaa 848
            |||||||||||||||||||||||||||||||||||||| |||||||||| ||||||||||
Sbjct: 429  cttatctaaattccacgcccggataaaacttcctgctaggttaaagcgacctgccagaaa 370


Query: 849  aatggccgcaaaaaatcccctgcgtcttccatcctcatctccggtcagcttatacttcat 908
            | |||||||||||| |||||||||||||||||||||||||||||||||||||||||||||
Sbjct: 369  attggccgcaaaaagtcccctgcgtcttccatcctcatctccggtcagcttatacttcat 310


Query: 909  cacc 912
            ||||
Sbjct: 309  cacc 306
```

==

Cphy0777 forward

Query= F02_777C2P16_1.SCF

>cphy_0777

```
Query: 73  aaagaaggagataggatcATGggtgaaatcaagtggatgcaggaccgggtctatggtttg 132
           ||||||||||||||||||||||||||||||||||||||||||||||||||||||||||||
Sbjct: 1   aaagaaggagataggatcatgggtgaaatcaagtggatgcaggaccgggtctatggtttg 60


Query: 133 atggtacattatctaagccacataatgccgaaagaaggagagaaaaggcaaagttgggat 192
           ||||||||||||||||||||||||||||||||||||||||||||||||||||||||||||
Sbjct: 61  atggtacattatctaagccacataatgccgaaagaaggagagaaaaggcaaagttgggat 120


Query: 193 gagatgactgctacatttccgatacaacaattctgtgatgcggtggcggactcaggagcc 252
           ||||||||||||||||||||||||||||||||||||||||||||||||||||||||||||
Sbjct: 121 gagatgactgctacatttccgatacaacaattctgtgatgcggtggcggactcaggagcc 180


Query: 253 ggctgggtgatctttcccttgggtcagaatacgggatattattgttctgaaaatgccgtg 312
           ||||||||||||||||||||||||||||||||||||||||||||||||||||||||||||
Sbjct: 181 ggctgggtgatctttcccttgggtcagaatacgggatattattgttctgaaaatgccgtg 240


Query: 313 ctggagcagtactggccaggaaaatgctcccaaagagacctgatgctagaactagctgag 372
           ||||||||||||||||||||||||||||||||||||||||||||||||||||||||||||
Sbjct: 241 ctggagcagtactggccaggaaaatgctcccaaagagacctgatgctagaactagctgag 300


Query: 373 gcactttcagagcgggatattaagttaatagtctatctgccgtctgaggtggacgcagct 432
           ||||||||||||||||||||||||||||||||||||||||||||||||||||||||||||
Sbjct: 301 gcactttcagagcgggatattaagttaatagtctatctgccgtctgaggtggacgcagct 360
```

```
Query: 433 ccagaagaagaacgtaaagcgctgggctgggaacaggattcccatgataaatcagtattt 492
           ||||||||||||||||||||||||||||||||||||||||||||||||||||||||||||
Sbjct: 361 ccagaagaagaacgtaaagcgctgggctgggaacaggattcccatgataaatcagtattt 420


Query: 493 atggaacgctatatgggaattatccggtactggtcacaaaaattcgggaagctggtttct 552
           ||||||||||||||||||||||||||||||||||||||||||||||||||||||||||||
Sbjct: 421 atggaacgctatatgggaattatccggtactggtcacaaaaattcgggaagctggtttct 480


Query: 553 ggatggtggtttgatggttgctattacgcatgggagaagacctttacaagaactcatagc 612
           |||||||||||||||||||||||| ||||| |||||||||||||||||||||||||||||
Sbjct: 481 ggatggtggtttgatggttgctataacgcaggggagaagacctttacaagaactcatagc 540


Query: 613 tgggataacacccgattcgattatttgcgctgggcatatggcagcccgggatgggcaatc 672
           ||||||||||||||||||||||||||||||| |||| ||||||||||||||| |||||||
Sbjct: 541 tgggataacacccgattcgattatttgcgct-ggcagatggcagcccgggat-ggcaatc 598


Query: 673 ctgatgccttgattgctaatgtgtccaggagcggaagacatgaaatatgttatttcggga 732
           |||||||| |||||||| ||||||||||||||||| ||||||| ||||| ||||||||||
Sbjct: 599 ctgatgccgtgattgct-atgtgtccaggagcggaggacatgagatatg-tatttcggga 656


Query: 733 tcaggatttacctggccgagaaaggcaattactctgaa 770
           |||||| ||||||||||| || |||||| |||||||||
Sbjct: 657 tcagga-ttacctggccg-gagaggcaaatactctgaa 692

================================================================================

Cphy0777 reverse

Query= F08_777C2P17_1.SCF

>cphy_0777


Query: 68   gtgtaatggatagtgatcTTAatggtgatggtgatgatgtcctgtaattctagaattctc 127
            ||||||||||||||||||||||||||||||||||||||||||||||||||||||||||||
Sbjct: 1002 gtgtaatggatagtgatcttaatggtgatggtgatgatgtcctgtaattctagaattctc 943


Query: 128  tatgtttttcttcacttccctaatctgttccaatgtttttcagccataactccatcttc 187
            ||||||||||||||||||||||||||||||||||||||||||||||||||||||||||||
Sbjct: 942  tatgtttttcttcacttccctaatctgttccaatgtttttcagccataactccatcttc 883


Query: 188  atagattcccacattccaggtgatagcgcctttttgctgctggcactggagtgcgaaatc 247
            ||||||||||||||||||||||||||||||||||||||||||||||||||||||||||||
Sbjct: 882  atagattcccacattccaggtgatagcgcctttttgctgctggcactggagtgcgaaatc 823


Query: 248  cgtcacctcttggtcagaaaacctaggcggcacgatctctcctggctccttaccgtgcat 307
            ||||||||||||||||||||||||||||||||||||||||||||||||||||||||||||
Sbjct: 822  cgtcacctcttggtcagaaaacctaggcggcacgatctctcctggctccttaccgtgcat 763
```

```
Query: 308  ccagaaacagtccagccataccagacagtgccactgcatgccgtcgatcagcggtccttc 367
            ||||||||||||||||||||||||||||||||||||||||||||||||||||||||||||
Sbjct: 762  ccagaaacagtccagccataccagacagtgccactgcatgccgtcgatcagcggtccttc 703


Query: 368  atttaaatgattcagagtatttgcctctccggccaggtaatcctgatcccgaaatacata 427
            ||||||||||||||||||||||||||||||||||||||||||||||||||||||||||||
Sbjct: 702  atttaaatgattcagagtatttgcctctccggccaggtaatcctgatcccgaaatacata 643


Query: 428  tctcatgtcctccgctcctggacacatagcaatcacggcatcaggattgccatcccgggc 487
            ||||||||||||||||||||||||||||||||||||||||||||||||||||||||||||
Sbjct: 642  tctcatgtcctccgctcctggacacatagcaatcacggcatcaggattgccatcccgggc 583


Query: 488  tgccatctgccagcgcaaataatcgaatcgggtgttatcccagctatgagttcttgtaaa 547
            ||||||||||||||||||||||||||||||||||||||||||||||||||||||||| ||
Sbjct: 582  tgccatctgccagcgcaaataatcgaatcgggtgttatcccagctatgagttcttgt-aa 524


Query: 548  aggtcttctcccctgcgttataacaaccatcaaaccaccatccagaaaccagcttcccga 607
            |||||||||||||||||||||| |||||||||||||||||||||||||||||||||||||
Sbjct: 523  aggtcttctcccctgcgttatagcaaccatcaaaccaccatccagaaaccagcttcccga 464


Query: 608  atttttgtgacctgtaccggataattcccatatagcgttcccataaatactgatttatc 667
            | |||||||||| |||||||||||||||||||||||||| ||||||||||||||||||||
Sbjct: 463  a-ttttgtgaccagtaccggataattcccatatagcgtt-ccataaatactgatttatc 406


Query: 668  atgggaatcctgtttccagcccatcgctttaaatttcttcttctggaactgcgttcacct 727
            |||||||||||||| |||||||| |||||| |  ||||||||||||| |||||| |||||
Sbjct: 405  atgggaatcctgttcccagcccagcgcttt-acgttcttcttctggagctgcgtccacct 347


Query: 728  caaacggcaaatatactattaac 750
            || |||||| ||| |||||||||
Sbjct: 346  cagacggcagatagactattaac 324
```

===
=

Cphy1169 forward

Query= A03_1169C2P16_1.SCF

>cphy_1169

```
Query: 72  aaagaaggagataggatcATGaaagcaaaacttactttatcaaagattagtaagatagga 131
           ||||||||||||||||||||||||||||||||||||||||||||||||||||||||||||
Sbjct: 1   aaagaaggagataggatcatgaaagcaaaacttactttatcaaagattagtaagatagga 60


Query: 132 accatagacaaaagaatttatggctcctttattgagcatctaggaagagcagtatacggt 191
           ||||||||||||||||||||||||||||||||||||||||||||||||||||||||||||
Sbjct: 61  accatagacaaaagaatttatggctcctttattgagcatctaggaagagcagtatacggt 120
```

```
Query: 192 ggtatttatgaaccaggtcatgaaacttcagatgacatgggatttcgtcaggatgtaatg 251
           ||||||||||||||||||||||||||||||||||||||||||||||||||||||||||||
Sbjct: 121 ggtatttatgaaccaggtcatgaaacttcagatgacatgggatttcgtcaggatgtaatg 180


Query: 252 gaattgattaaagaattaaatattccaatcgttcgttatccaggtggtaattttgtatca 311
           ||||||||||||||||||||||||||||||||||||||||||||||||||||||||||||
Sbjct: 181 gaattgattaaagaattaaatattccaatcgttcgttatccaggtggtaattttgtatca 240


Query: 312 ggctatcgttgggaagatggtattggagataatgcgaagagaccaaggcgtcttgacctt 371
           |||||||||||||||||||||||||||||||| |||||||||||||||||||||||||||
Sbjct: 241 ggctatcgttgggaagatggtattggagataaggcgaagagaccaaggcgtcttgacctt 300


Query: 372 gcttggaagagcattgagacaaatgaggttggtgtagatgagtttcaagaatgggcgaaa 431
           ||||||||||||||||||||||||||||||||||||||||||||||| ||||||||||||
Sbjct: 301 gcttggaagagcattgagacaaatgaggttggtgtagatgagtttcaggaatgggcgaaa 360


Query: 432 cgtgccaatagtgatgttatgatggctgttaatctaggtaccagaggaccacaagaagcg 491
           ||||||||||||||||||||||||||||||||||||||||||||||||||||||||||||
Sbjct: 361 cgtgccaatagtgatgttatgatggctgttaatctaggtaccagaggaccacaagaagcg 420


Query: 492 catgatttaatttgagtattgtaattttcaaggaggaacttattactgttgacttatgaa 551
           || ||||||| ||||||||||||||||||||||||||||||||||| | ||||||| |||
Sbjct: 421 caggatttaa-ttgagtattgtaattttcaaggaggaacttattacag-tgacttaagaa 478


Query: 552 gaaagaacggatttgagaagccatttgatatttcagtttggtgcttatgttatgagatgg 611
           ||||||||||||||||||||||||||||||||  ||||||||||||| || |||||||||
Sbjct: 479 gaaagaacggatttgagaagccatttgatattaaagtttggtgcttaggtaatgagatgg 538


Query: 612 atggactctgtctaacctgtgctaaaactgtataagaatatgggagaattg 662
           ||||||  || | |||||||||||| || | | ||||||| ||||||||||
Sbjct: 539 atggaccatggcaaacctgtgctaagacagcagaagaatacgggagaattg 589
```

===

Cphy1169 reverse

Query= A09_1169C2P17_1.SCF

>cphy_1169

```
Query: 66   gtgtaatggatagtgatcTTAatggtgatggtgatgatgcaatgaaaaacgaattacatt 125
            ||||||||||||||||||||||||||||||||||||||||||||||||||||||||||||
Sbjct: 1554 gtgtaatggatagtgatcttaatggtgatggtgatgatgcaatgaaaaacgaattacatt 1495


Query: 126  ccaagaataaggctttaacgtagctattgcggtattttcctctatttttgtaactccagt 185
            ||||||||||||||||||||||||||||||||||||||||||||||||||||||||||||
Sbjct: 1494 ccaagaataaggctttaacgtagctattgcggtattttcctctatttttgtaactccagt 1435
```

```
Query: 186  tgcactaggaactacattcttaggattgcttgcagtattcgtagcttttaaatcagcatg 245
            ||||||||||||||||||||||||||||||||||||||||||||||||||||||||||||
Sbjct: 1434 tgcactaggaactacattcttaggattgcttgcagtattcgtagcttttaaatcagcatg 1375


Query: 246  aatcatctcagtatgctccaccaattttgcattctgaaactctgttaaatcaattgaaaa 305
            ||||||||||||||||||||||||||||||||||||||||||||||||||||||||||||
Sbjct: 1374 aatcatctcagtatgctccaccaattttgcattctgaaactctgttaaatcaattgaaaa 1315


Query: 306  atcaatggtttgatctgcggagcgatttacagcaaacactacaacctcattcttttcttc 365
            ||||||||||||||||||||||||||||||||||||||||||||||||||||||||||||
Sbjct: 1314 atcaatggtttgatctgcggagcgatttacagcaaacactacaacctcattcttttcttc 1255


Query: 366  attatgtactgcaactgtttctacataaggtactttatcactgattttattctcataagt 425
            ||||||||||||||||||||||||||||||||||||||||||||||||||||||||||||
Sbjct: 1254 attatgtactgcaactgtttctacataaggtactttatcactgattttattctcataagt 1195


Query: 426  ctcgcactctaagattggttttaaggcggtacccctaccataatttgatacgtgcataaa 485
            ||||||||||||||||||||||||||||||||||||||||||||||||||||||||||||
Sbjct: 1194 ctcgcactctaagattggttttaaggcggtacccctaccataatttgatacgtgcataaa 1135


Query: 486  tggatagaaaattgtttgagcccaagcagctccaccattctctgtcataataggagcaat 545
            ||||||||||||||||||||||||||||||||||||||||||||||||||||||||||||
Sbjct: 1134 tggatagaaaattgtttgagcccaagcagctccaccattctctgtcataataggagcaat 1075


Query: 546  aacatttactaattgagcaagacacgccatctttaacgcggtcacaatgtttaaccagtg 605
            |||||||||||||||||||||||||||||||||| |||||||||||||||||||||||||
Sbjct: 1074 aacatttactaattgagcaagacacgccatcttt-acgcggtcacaatgtttaaccagtg 1016


Query: 606  cgattagcaaacatcccactaacaaagcatcttccaaagtgtagacatcctctaaataaa 665
            |||||||||||||||||||||||||||||||||| |||||||||||||||||| ||||||
Sbjct: 1015 cgattagcaaacatcccactaacaaagcatcttcaaaagtgtagacatcctct-aataaa 957


Query: 666  ctaggagctttgccccaaggctctatcctcttatctttgaccatgactaatggaaccata 725
            ||||||||||||||||||||||||||||||||||| |||||||||||| |||||||||||
Sbjct: 956  ctaggagctttgccccaaggctctatcctcttatc-ttgaccatgact-atggaaccata 899


Query: 726  cattccactcatcaaaggaaagattgatggtctttttaaaatgctttttccctttcaaag 785
            |||||||||||||||||||||||||||||||||||||| ||||||||||| |||||| ||
Sbjct: 898  cattccactcatcaaaggaaagattgatggtctttttagaatgctttttcgctttcacag 839


Query: 786  aatcacaaattgctactaccgattcaaataaaggtttccattcttctaatgatttaagct 845
            ||||||||||||||||||||||||| |||||||||||||||   ||||||||| | ||||
Sbjct: 838  aatcacaaattgctactaccgattc-aataaaggtttccat--ctctaatgatct-agct 783


Query: 846  nnnnnnncatctggattcattttcataatttattatagtactgggttgcaggggaaatat 905
```

```
                   |||||||| |||||||||||| |||||||||||||||  |||| |||||||||
Sbjct: 782  aagaaaacatctgga-tcattttcataa-ttattatagtactgg--tgca-gggaaatat 728


Query: 906  aatcaacttaaccaatagg-atgttctaatacccgcacgtccccaatcctccaaaggtag 964
            ||||||| ||| | ||||| ||||||||||| ||| |||| |||||| ||||||||||||
Sbjct: 727  aatcaacataatc-ataggtatgttctaata-ccgtacgttcccaat-ctccaaaggtag 671


Query: 965  gctttgttaaaccaaaactaaccacaagc 993
            || ||||||||||| |||| |||||||||
Sbjct: 670  gcattgttaaaccagaact-accacaagc 643

=================================================================================

 Cphy1612 forward

Query= HR0ADKBY98ZC02_1612C2P16_1.SCF

>cphy_1612



Query: 66  aaagaaggagataggatcATGagtactaacattgacctaatcgaaagtagtggatggtta 125
           ||||||||||||||||||||||||||||||||||||||||||||||||||||||||||||
Sbjct: 1   aaagaaggagataggatcatgagtactaacattgacctaatcgaaagtagtggatggtta 60


Query: 126 gaatccgcatatgtgaagtggatgcctatggacggggtagacggatatgtagcgtatgtt 185
           ||||||||||||||||||||||||||||||||||||||||||||||||||||||||||||
Sbjct: 61  gaatccgcatatgtgaagtggatgcctatggacggggtagacggatatgtagcgtatgtt 120


Query: 186 aagggtgcttatgaagaggataccgcttatgttaagttagataatgaattaataagaaaa 245
           ||||||||||||||||||||||||||||||||||||||||||||||||||||||||||||
Sbjct: 121 aagggtgcttatgaagaggataccgcttatgttaagttagataatgaattaataagaaaa 180


Query: 246 tatccaacttactatagggcggatgccgtagggcttgcagcaggtatttatgtcattaaa 305
           ||||||||||||||||||||||||||||||||||||||||||||||||||||||||||||
Sbjct: 181 tatccaacttactatagggcggatgccgtagggcttgcagcaggtatttatgtcattaaa 240


Query: 306 gttgtccctgttatatctgggatagaagttgaggagagtgcagaggtaactggtacttta 365
           ||||||||||||||||||||||||||||||||||||||||||||||||||||||||||||
Sbjct: 241 gttgtccctgttatatctgggatagaagttgaggagagtgcagaggtaactggtacttta 300


Query: 366 tttgtatcagcacatacaagagaaggttttgcattttcagagttttcaacgtacaaaaca 425
           ||||||||||||||||||||||||||||||||||||||||||||||||||||||||||||
Sbjct: 301 tttgtatcagcacatacaagagaaggttttgcattttcagagttttcaacgtacaaaaca 360


Query: 426 ggatccggtggttataataacgatggaactgttagagatggtgcacagattgtttatcta 485
           ||||||||||||||||||||||||||||||||||||||||||||||||||||||||||||
Sbjct: 361 ggatccggtggttataataacgatggaactgttagagatggtgcacagattgtttatcta 420
```

```
Query: 486 accaatgaaaataaggatactgttaccttgggcgtaaacacaggtagcaaggaggaaact 545
           ||||||||||||||||||||||||||||||||||||||||||||||||||||||||||||
Sbjct: 421 accaatgaaaataaggatactgttaccttgggcgtaaacacaggtagcaaggaggaaact 480


Query: 546 tgtaccggtattgttgacattttgtcaaagagagaaaagggaaaagatttaaggccactt 605
           ||||||||||||||||||||||||||||||||||||||||||||||||||||||||||||
Sbjct: 481 tgtaccggtattgttgacattttgtcaaagagagaaaagggaaaagatttaaggccactt 540


Query: 606 atcatacgtatcattggaaaagtagaaactcctgctggagtgagtggttctttgttaaat 665
           ||||||||||||||||||||||||||||||||||||||||||||||||||||||||||||
Sbjct: 541 atcatacgtatcattggaaaagtagaaactcctgctggagtgagtggttctttgttaaat 600


Query: 666 ataaagaaaacttccaacgtaacattagaaggtatcggagacgatgcaacagtctatggc 725
           ||||||||||||||||||||||||||||||||||||||||||||||||||||||||||||
Sbjct: 601 ataaagaaaacttccaacgtaacattagaaggtatcggagacgatgcaacagtctatggc 660


Query: 726 tggggtattctagttcgaaaaggtaaaaacattgaaattagaaacttagggatcatgtgg 785
           |||||||||||||||||| |||||||||||||||| |||||||||||||| |||||||||
Sbjct: 661 tggggtattctagttcgagaaggtaaaaacattgagattagaaacttaggaatcatgtgg 720


Query: 786 tttggtgatgatgcagtttcattggacacctaaaaaatgagaaatatttgggttcttaac 845
           |||||||||||||||||||||||||||||   ||||||||| ||||| ||||||| ||||
Sbjct: 721 tttggtgatgatgcagtttcattggacac--taaaaatgag-aatatatgggttcataac 777


Query: 846 aatgattttattttatgggaaaaaccaggggagcgattct 885
           ||||| ||||||||||||  ||||||| ||||||||||||
Sbjct: 778 aatga-tttattttatgg--aaaacca-gggagcgattct 813

==============================================================================================

Cphy1612 reverse



Query= HR0ADKBY98ZC02_1612C2P17_1.SCF

>cphy_1612


Query: 65   gtgtaatggatagtgatcTTAatggtgatggtgatgatgtttataaacttgcactttggt 124
            ||||||||||||||||||||||||||||||||||||||||||||||||||||||||||||
Sbjct: 4182 gtgtaatggatagtgatcttaatggtgatggtgatgatgtttataaacttgcactttggt 4123


Query: 125  tttgatagaaagtaatttgccatttttatctttcacactaacaacgacataatcttcccc 184
            ||||||||||||||||||||||||||||||||||||||||||||||||||||||||||||
Sbjct: 4122 tttgatagaaagtaatttgccatttttatctttcacactaacaacgacataatcttcccc 4063


Query: 185  tacagattttgctgatactacagcggttaatttaccattatttttgccaacgatagcaat 244
            ||||||||||||||||||||||||||||||||||||||||||||||||||||||||||||
Sbjct: 4062 tacagattttgctgatactacagcggttaatttaccattatttttgccaacgatagcaat 4003
```

```
Query: 245  atctcgctttgtagttttccatacaatttcttcatttctatagccattgacttccaatgt 304
            ||||||||||||||||||||||||||||||||||||||||||||||||||||||||||||
Sbjct: 4002 atctcgctttgtagttttccatacaatttcttcatttctatagccattgacttccaatgt 3943


Query: 305  aaatgagtttttatcacctatcaccatggatgaagctgccctcttaaacgtaatgctcgc 364
            ||||||||||||||||||||||||||||||||||||||||||||||||||||||||||||
Sbjct: 3942 aaatgagtttttatcacctatcaccatggatgaagctgccctcttaaacgtaatgctcgc 3883


Query: 365  ttcatttactgtgacttgttgttgtaattgctttgttttaccgtctgacaaagtaactgt 424
            ||||||||||||||||||||||||||||||||||||||||||||||||||||||||||||
Sbjct: 3882 ttcatttactgtgacttgttgttgtaattgctttgttttaccgtctgacaaagtaactgt 3823


Query: 425  aatataaattatggtttttcctttactcttaccaacaatctttccagtttgacttactat 484
            ||||||||||||||||||||||||||||||||||||||||||||||||||||||||||||
Sbjct: 3822 aatataaattatggtttttcctttactcttaccaacaatctttccagtttgacttactat 3763


Query: 485  agcaatcttggtattaccagacttataactaagcatcatttcaccttcggaaatattagt 544
            ||||||||||||||||||||||||||||||||||||||||||||||||||||||||||||
Sbjct: 3762 agcaatcttggtattaccagacttataactaagcatcatttcaccttcggaaatattagt 3703


Query: 545  attggagcttgtaaagtcctttacaacttttagtgtacttgggtaacgtaagcctaccca 604
            ||||||||||||||||||||||||||||||||||||||||||||||||||||||||||||
Sbjct: 3702 attggagcttgtaaagtcctttacaacttttagtgtacttgggtaacgtaagcctaccca 3643


Query: 605  ttcccaatccattgttcctccaatataaatannnnnnncggaatttacattaatctgatc 664
            |||||||||||||||||||||||||||||||       ||||||||||||||||||||||
Sbjct: 3642 ttcccaatccattgttcctccaatataaatatttttttcggaatttacattaatctgatc 3583


Query: 665  aattaacgccactttcggtgttggttcaggtgttggacttggttttacgtctggtactgg 724
            ||||||||||||||||||||||||||||||||||||||||||||||||||||||||||||
Sbjct: 3582 aattaacgccactttcggtgttggttcaggtgttggacttggttttacgtctggtactgg 3523


Query: 725  atctggtttcccccctggtgttggacttggctttacctctggtgttggtttttggtgtta 784
            |||||||||||||||||||||||||||||||||||||||||||||||| |||||||||||
Sbjct: 3522 atctggtttcccccctggtgttggacttggctttacctctggtgttgg-ttttggtgtta 3464


Query: 785  cttcaggggttggcgttggttcaggggtaggctctacaataacctcatctttaaaaactg 844
            ||||||||||||||||||||||||||||||||||||||||||||||||||||||||||||
Sbjct: 3463 cttcaggggttggcgttggttcaggggtaggctctacaataacctcatctttaaaaactg 3404


Query: 845  caccaataaaaccgccattcgttgagaaagttgcctatttactaattctccttaattttg 904
            |||||||||||||||||||||||||||| ||||| |||||||| ||||||||||| ||||
Sbjct: 3403 caccaataaaaccgccattcgttgagaatgttgcatatttact-attctccttaa-tttg 3346


Query: 905  ca-aaaaccatgagtattaatggaacc 930
```

```
              || ||||||||||||||||||||||||
Sbjct: 3345 cagaaaaccatgagtattaatggaacc 3319

===============================================================================

Cphy1640 forward

Query= HR0ADKBY98ZA02_1640C3P16_1.SCF

>cphy_1640


Query: 68  aaagaaggagataggatcATGacttggtcatcagctgaccaatgggctacatggagtaat 127
            ||||||||||||||||||||||||||||||||||||||||||||||||||||||||||||
Sbjct: 1   aaagaaggagataggatcatgacttggtcatcagctgaccaatgggctacatggagtaat 60


Query: 128 ggaggttatacgatttataataatgtatggggcagcggtgcaggataccaatcaatctgg 187
            ||||||||||||||||||||||||||||||||||||||||||||||||||||||||||||
Sbjct: 61  ggaggttatacgatttataataatgtatggggcagcggtgcaggataccaatcaatctgg 120


Query: 188 gctaatagctacagcaattggggtgtatgggcgcagcatcctaatacaggaggtataaaa 247
            ||||||||||||||||||||||||||||||||||||||||||||||||||||||||||||
Sbjct: 121 gctaatagctacagcaattggggtgtatgggcgcagcatcctaatacaggaggtataaaa 180


Query: 248 tcttatccaaatgtaactaagtcgattggcaaaacattatcttcgataaaaaccttacaa 307
            ||||||||||||||||||||||||||||||||||||||||||||||||||||||||||||
Sbjct: 181 tcttatccaaatgtaactaagtcgattggcaaaacattatcttcgataaaaaccttacaa 240


Query: 308 agtagttttaatgttaccaggccaagtagcggggcttatgaaagtgcttatgatatttgg 367
            ||||||||||||||||||||||||||||||||||||||||||||||||||||||||||||
Sbjct: 241 agtagttttaatgttaccaggccaagtagcggggcttatgaaagtgcttatgatatttgg 300


Query: 368 tcgaatggctcgtcttatgaaattatgttatggatgaattcatatggaagtgttgctccg 427
            ||||||||||||||||||||||||||||||||||||||||||||||||||||||||||||
Sbjct: 301 tcgaatggctcgtcttatgaaattatgttatggatgaattcatatggaagtgttgctccg 360


Query: 428 atatcttataattgggatgcttccggtaagcctgtaccagtctatacgaatcttagtcta 487
            ||||||||||||||||||||||||||||||||||||||||||||||||||||||||||||
Sbjct: 361 atatcttataattgggatgcttccggtaagcctgtaccagtctatacgaatcttagtcta 420


Query: 488 ggcggtcattcatggaatgtgtatcagggtagcaatggatcgaatccggtatattcattc 547
            ||||||||||||||||||||||||||||||||||||||||||||||||||||||||||||
Sbjct: 421 ggcggtcattcatggaatgtgtatcagggtagcaatggatcgaatccggtatattcattc 480


Query: 548 gtaagaacaagtagtacgaattccggtacggtcgatatattagctatacttaattggatt 607
            ||||||||||||||||||||||||||||||||||||||||||||||||||||||||||||
Sbjct: 481 gtaagaacaagtagtacgaattccggtacggtcgatatattagctatacttaattggatt 540
```

```
Query: 608 aatggtaaaggctggtttagtggaactcaaacagttaatttagttcaatttgggtttgag 667
           ||||||||||||||||||||||||||||||||||||||||||||||||||||||||||||
Sbjct: 541 aatggtaaaggctggtttagtggaactcaaacagttaatttagttcaatttgggtttgag 600


Query: 668 attacttcatcaagtgccggatcagactttatctgcaatagttattctgtaaccagtaat 727
           ||||||||||||||||||||||||||||||||||||||||||||||||||||||||||||
Sbjct: 601 attacttcatcaagtgccggatcagactttatctgcaatagttattctgtaaccagtaat 660


Query: 728 catcatcaccatcaccattaagatcactatccattacac 766
           |||||||||||||||||||||||||||||||||||||||
Sbjct: 661 catcatcaccatcaccattaagatcactatccattacac 699

================================================================================

 Cphy1640 reverse

Query= HR0ADKBY98ZA02_1640C3P17_1.SCF

>cphy_1640


Query: 66  gtgtaatggatagtgatcTTAatggtgatggtgatgatgattactggttacagaataact 125
           ||||||||||||||||||||||||||||||||||||||||||||||||||||||||||||
Sbjct: 699 gtgtaatggatagtgatcttaatggtgatggtgatgatgattactggttacagaataact 640


Query: 126 attgcagataaagtctgatccggcacttgatgaagtaatctcaaacccaaattgaactaa 185
           ||||||||||||||||||||||||||||||||||||||||||||||||||||||||||||
Sbjct: 639 attgcagataaagtctgatccggcacttgatgaagtaatctcaaacccaaattgaactaa 580


Query: 186 attaactgtttgagttccactaaaccagcctttaccattaatccaattaagtatagctaa 245
           ||||||||||||||||||||||||||||||||||||||||||||||||||||||||||||
Sbjct: 579 attaactgtttgagttccactaaaccagcctttaccattaatccaattaagtatagctaa 520


Query: 246 tatatcgaccgtaccggaattcgtactacttgttcttacgaatgaatataccggattcga 305
           ||||||||||||||||||||||||||||||||||||||||||||||||||||||||||||
Sbjct: 519 tatatcgaccgtaccggaattcgtactacttgttcttacgaatgaatataccggattcga 460


Query: 306 tccattgctaccctgatacacattccatgaatgaccgcctagactaagattcgtatagac 365
           ||||||||||||||||||||||||||||||||||||||||||||||||||||||||||||
Sbjct: 459 tccattgctaccctgatacacattccatgaatgaccgcctagactaagattcgtatagac 400


Query: 366 tggtacaggcttaccggaagcatcccaattataagatatcggagcaacacttctatatga 425
           |||||||||||||||||||||||||||||||||||||||||||||||||||| ||||||
Sbjct: 399 tggtacaggcttaccggaagcatcccaattataagatatcggagcaacacttccatatga 340


Query: 426 attcatccataacataatttcataagacgagccattcgaccaaatatcataagcactttc 485
           ||||||||||||||||||||||||||||||||||||||||||||||||||||||||||||
Sbjct: 339 attcatccataacataatttcataagacgagccattcgaccaaatatcataagcactttc 280
```

```
Query: 486 ataagccccgctacttggcctggtaacattaaaactactttgtaaggtttttatcgaaga 545
           ||||||||||||||||||||||||||||||||||||||||||||||||||||||||||||
Sbjct: 279 ataagccccgctacttggcctggtaacattaaaactactttgtaaggtttttatcgaaga 220


Query: 546 taatgttttgccaatcgacttagttacatttggataagattttatacctcctgtattagg 605
           ||||||||||||||||||||||||||||||||||||||||||||||||||||||||||||
Sbjct: 219 taatgttttgccaatcgacttagttacatttggataagattttatacctcctgtattagg 160


Query: 606 atgctgcgcccatacaccccaattgctgtagctattagcccagattgattggtatcctgc 665
           ||||||||||||||||||||||||||||||||||||||||||||||||||||||||||||
Sbjct: 159 atgctgcgcccatacaccccaattgctgtagctattagcccagattgattggtatcctgc 100


Query: 666 accgctgccccatacattattataaatcgtataacctccattactccatgtagcccattg 725
           ||||||||||||||||||||||||||||||||||||||||||||||||||||||||||||
Sbjct: 99  accgctgccccatacattattataaatcgtataacctccattactccatgtagcccattg 40


Query: 726 gtcagctgatgaccaagtcatgatcctatctccttcttt 764
           |||||||||||||||||||||||||||||||||||||||
Sbjct: 39  gtcagctgatgaccaagtcatgatcctatctccttcttt 1
```

==

Cphy2028 forward

Query= C04_2028C3P16_1.SCF

>cphy_2028

```
Query: 69  aaagaaggagataggatcATGaattatcaaaatcctattattaggggattttatccggat 128
           ||||||||||||||||||||||||||||||||||||||||||||||||||||||||||||
Sbjct: 1   aaagaaggagataggatcatgaattatcaaaatcctattattaggggattttatccggat 60


Query: 129 ccaagtgtatgctttgcaaatggtaaatattatttagtctgcagttccatgcagtatttt 188
           ||||||||||||||||||||||||||||||||||||||||||||||||||||||||||||
Sbjct: 61  ccaagtgtatgctttgcaaatggtaaatattatttagtctgcagttccatgcagtatttt 120


Query: 189 cctggtgtacccttatttgaaagcgaggacttggtaaattggactcagattggccactgt 248
           ||||||||||||||||||||||||||||||||||||||||||||||||||||||||||||
Sbjct: 121 cctggtgtacccttatttgaaagcgaggacttggtaaattggactcagattggccactgt 180


Query: 249 ctaacaagacctgatcagattgcattacaaggggtcagcagttcaggcggggtttttgct 308
           ||||||||||||||||||||||||||||||||||||||||||||||||||||||||||||
Sbjct: 181 ctaacaagacctgatcagattgcattacaaggggtcagcagttcaggcggggtttttgct 240


Query: 309 ccttccatacgatattttgaaggtcgattctacatgaccaccacgaactctaccactcag 368
           ||||||||||||||||||||||||||||||||||||||||||||||||||||||||||||
Sbjct: 241 ccttccatacgatattttgaaggtcgattctacatgaccaccacgaactctaccactcag 300
```

```
Query: 369 cagaannnnnnngtttggacggatgatatctatggtgaatggtccgaccatattttcatt 428
           |||||       ||||||||||||||||||||||||||||||||||||||||||||||||
Sbjct: 301 cagaatttttttgtttggacggatgatatctatggtgaatggtccgaccatattttcatt 360


Query: 429 gatcaggacggaatctatccatccctatttttcgaaaacggaaaaagttattttatgagc 488
           ||||||||||||||| ||||||||||||||||||||||||||||||||||||||||||||
Sbjct: 361 gatcaggacggaatcgatccatccctatttttcgaaaacggaaaaagttattttatgagc 420


Query: 489 aacggatctgatgcagagggaatatcaggtatcgtgcaatgtgaaatcgctatcgacaca 548
           ||||||||||||||||||||||||||||||||||||||||||||||||||||||||||||
Sbjct: 421 aacggatctgatgcagagggaatatcaggtatcgtgcaatgtgaaatcgctatcgacaca 480


Query: 549 tgtaaaaagctttccccaagtaaaactatctggcagggttcaggtggtcgttattttaga 608
            |||||||||||||||||||||||||||||||||||||||||||||||||||| ||||||
Sbjct: 481 ggtaaaaagctttccccaagtaaaactatctggcagggttcaggtggtcgtta-tttaga 539


Query: 609 aggaccccatttatataaaatcaaagattattattatctggtagctgcaaagggaggta 668
           ||||||||| |||||||||||||||||||||||||||||||||||||||| |||||||||
Sbjct: 540 aggacccca-tttatataaaatcaaagattattattatctggtagctgcagagggaggta 598


Query: 669 ctgaatatggtcatatggtcacctatggcagaagtacttctccttacggtcccctttaaa 728
           ||||||||||||||||||||||||||| |||||||||||||||||||||| |||||| ||
Sbjct: 599 ctgaatatggtcatatggtcacctatgccagaagtacttctccttacggt-ccctttgaa 657


Query: 729 tcctaccctaccaatcccggtccttactaa-aataacctaggtgggttttcaattcaagg 787
            ||||||||| |||| |||||||||||||| |  ||||||||||| |||  |||||||||
Sbjct: 658 gcctaccctagcaat-ccggtccttactaacagaaacctaggtggttttcaaattcaagg 716


Query: 788 tgtccggcatggtgaattgattgaaactcc 817
           |||| |||||||||| ||||||||| ||||
```

==

Cphy2028 reverse

Query= C10_2028C3P17_1.SCF

>cphy_2028

```
Query: 69   gtgtaatggatagtgatcTTAatggtgatggtgatgatgttcatgcaagtattcacattt 128
             ||||||||||||||||||||||||||||||||||||||||||||||||||||||||||||
Sbjct: 1551 gtgtaatggatagtgatcttaatggtgatggtgatgatgttcatgcaagtattcacattt 1492


Query: 129  aaaatccttgaatttagcagtatttatggagttatgatcaagagcatataaaccaatgat 188
             ||||||||||||||||||||||||||||||||||||||||||||||||||||||||||||
Sbjct: 1491 aaaatccttgaatttagcagtatttatggagttatgatcaagagcatataaaccaatgat 1432
```

```
Query: 189  aactccggtaaatccaccagcaacttcggaggaaagataacgggtcaacccggtaccaag 248
            ||||||||||||||||||||||||||||||||||||||||||||||||||||||||||||
Sbjct: 1431 aactccggtaaatccaccagcaacttcggaggaaagataacgggtcaacccggtaccaag 1372


Query: 249  atggatgatttcattttctgatttgacataaaaactgtactgttgactgttagaatcaat 308
            ||||||||||||||||||||||||||||||||||||||||||||||||||||||||||||
Sbjct: 1371 atggatgatttcattttctgatttgacataaaaactgtactgttgactgttagaatcaat 1312


Query: 309  tatgaaggaagcacgattgctatcacctaaagaatattcagcttcaatggattttatatt 368
            ||||||||||||||||||||||||||||||||||||||||||||||||||||||||||||
Sbjct: 1311 tatgaaggaagcacgattgctatcacctaaagaatattcagcttcaatggattttatatt 1252


Query: 369  accaatacataaccttacgattactcggaagccttctggatatttcgatattgcaagatc 428
            ||||||||||||||||||||||||||||||||||||||||||||||||||||||||||||
Sbjct: 1251 accaatacataaccttacgattactcggaagccttctggatatttcgatattgcaagatc 1192


Query: 429  ataatggtggttttcgtccatgtaaaaagttatgcctgcttcaccacggtcaatttcaat 488
            ||||||||||||||||||||||||||||||||||||||||||||||||||||||||||||
Sbjct: 1191 ataatggtggttttcgtccatgtaaaaagttatgcctgcttcaccacggtcaatttcaat 1132


Query: 489  atcacaatttatgcgacagcagaattctttctgccttaatccgataaaagtcggagaatc 548
            ||||||||||||||||||||||||||||||||||||||||||||||||||||||||||||
Sbjct: 1131 atcacaatttatgcgacagcagaattctttctgccttaatccgataaaagtcggagaatc 1072


Query: 549  aacatcatcaagtgagatactgtttccatttaatataatttgactttgggataatgaata 608
            ||||||||||||||||||||||||||||||||||||||||||||||||||||||||||||
Sbjct: 1071 aacatcatcaagtgagatactgtttccatttaatataatttgactttgggataatgaata 1012


Query: 609  gttttccctagaaggatgcctttggtagcaccagtccttctcaaagcctgtattacaaaa 668
            |||||||||||||||||||||||||||||||||||||||||||||| |||||||||||||
Sbjct: 1011 gttttccctagaaggatgcctttggtagcaccagtccttctcaaagtctgtattacaaaa 952


Query: 669  agtatattcggttttgaactcctgcactttatatgtaaaaggtaccttcaacatatttct 728
            |||||||||||||||||||||||||||||||||||||||||||||| |||||||| ||||
Sbjct: 951  agtatattcggttttgaactcctgcactttatatgtaaaaggtacc-tcaacata-ttct 894


Query: 729  gaagtaattccgtctgttcccaacagtaaaccctccatcatcatggaaactcaccggtga 788
            |||||| ||||||||||| ||||||||||||| |||||||||||||||||||||||||||
Sbjct: 893  gaagtagttccgtctgtt-ccaacagtaaaccatccatcatcatggaaactcaccggtga 835


Query: 789  cagggaatacttcccttcctaattgatgggaaggaagtcaacccaacctgtccgaacgaa 848
            || ||||||||||||||||||| |||| |||||| |||| | || ||||||| | |||||
Sbjct: 834  ca-ggaatacttcccttcctaaatgat-ggaaggtagtcca-cctacctgtctg-acgaa 779


Query: 849  aacccaggtgaagcatccaaaaaacttcccgtcaggaaccttcatccaatccccatgccc 908
            |||||||||||||||||| |||||||| ||||||||| | |  ||| |||| ||||| ||
```

```
Sbjct: 778  aacccaggtgaagcatcc-aaaaactt-ccgtcaggagcttcaatcaaatcaccatg-cc 722


Query: 909  ccaaaccttgaaatttgaaacccacctaggttt 941
            | | |||||| ||||||||| ||||||||||||
Sbjct: 721  cgacaccttg-aatttgaaaaccacctaggttt 690
```

==

Cphy2058 forward

Query= D04_2058C1P16_1.SCF

>cphy_2058

```
Query: 69   aaagaaggagataggatcATGacagtgaatttgaattcgcagaatacagacaatcaaaat 128
            ||||||||||||||||||||||||||||||||||||||||||||||||||||||||||||
Sbjct: 1    aaagaaggagataggatcatgacagtgaatttgaattcgcagaatacagacaatcaaaat 60


Query: 129  caagatgcaaataataaagactcggataatcaaggcgcaaataatcaagagtcaaataat 188
            ||||||||||||||||||||||||||||||||||||||||||||||||||||||||||||
Sbjct: 61   caagatgcaaataataaagactcggataatcaaggcgcaaataatcaagagtcaaataat 120


Query: 189  caagaatcacaaaaccctgaattaaaaaatacaccaacacctcaaatcatagtactacca 248
            ||||||||||||||||||||||||||||||||||||||||||||||||||||||||||||
Sbjct: 121  caagaatcacaaaaccctgaattaaaaaatacaccaacacctcaaatcatagtactacca 180


Query: 249  ggtgacagcacaattgaagcaaacgaaattgtagccccaaccatcacatttgaacaaaaa 308
            ||||||||||||||||||||||||||||||||||||||||||||||||||||||||||||
Sbjct: 181  ggtgacagcacaattgaagcaaacgaaattgtagccccaaccatcacatttgaacaaaaa 240


Query: 309  gaaataccatcgaatccagcaataacttttgtacataatatgaaaataggatggaacctt 368
            ||||||||||||||||||||||||||||||||||||||||||||||||||||||||||||
Sbjct: 241  gaaataccatcgaatccagcaataacttttgtacataatatgaaaataggatggaacctt 300


Query: 369  ggaaatacatttgacgcagtgagtgattccaatctaatggatgaacttaattatgaaagc 428
            ||||||||||||||||||||||||||||||||||||||||||||||||||||||||||||
Sbjct: 301  ggaaatacatttgacgcagtgagtgattccaatctaatggatgaacttaattatgaaagc 360


Query: 429  tcatggtgtggtgtaaaaacaacagaagagatgatgaaagcaattaaagatgctgggttt 488
            ||||||||||||||||||||||||||||||||||||||||||||||||||||||||||||
Sbjct: 361  tcatggtgtggtgtaaaaacaacagaagagatgatgaaagcaattaaagatgctgggttt 420


Query: 489  cagtcgattagaataccagtatcgtggcacaatcatgtttctggtgatgattttattata 548
            ||||||||||||||||||||||||||||||||||||||||||||||||||||||||||||
Sbjct: 421  cagtcgattagaataccagtatcgtggcacaatcatgtttctggtgatgattttattata 480


Query: 549  agcgaagtatggcttaaccgagtacaagaagtggtcgattatgctatcaataatgatatg 608
            ||||||||||||||||||||||||||||||||||||||||||||||||||||||||||||
Sbjct: 481  agcgaagtatggcttaaccgagtacaagaagtggtcgattatgctatcaataatgatatg 540
```

```
Query: 609  tatgtgatattaaatactcaccatgatgtaagtaaaaatttttattatccaagtaatgaa 668
            ||||||||||||||||||||||||||||||||||||||||||||||||||||||||||||
Sbjct: 541  tatgtgatattaaatactcaccatgatgtaagtaaaaatttttattatccaagtaatgaa 600


Query: 669  aatttagaatcttctaaaaaatatatcaacgcagtatggacacaagtaagtgaacgattt 728
            ||||||||||||||||||||||||||||||||||||||||||||||||||||||||||||
Sbjct: 601  aatttagaatcttctaaaaaatatatcaacgcagtatggacacaagtaagtgaacgattt 660


Query: 729  tcttcctatggagaaaagttattatttgaagggatgaacgaaccaatgcttgcaggttct 788
            |||||||||||||||||||||||||||||||||||||||||||||| |||||||||||||
Sbjct: 661  tcttcctatggagaaaagttattatttgaagggatgaacgaaccaaggcttgcaggttct 720


Query: 789  aatttacgaatggtggttagatttatcaaagcctgagtgtaaagaagcaatcgaaagtat 848
            || |||||||||||||||||||||||||||||||||||||||||||||||||||| ||||
Sbjct: 721  aa-ttacgaatggtggttagatttatcaaagcctgagtgtaaagaagcaatcgaatgtat 779


Query: 849  taatcaattaaatcaagaatttgttgataccgttcgcaaatcggggaggagagaatactt 908
            ||||||||||||||| |||||||||||||||||||||||||| |||||||||||||||||
Sbjct: 780  taatcaattaaatcaggaatttgttgataccgttcgcaaatc-gggaggagagaatactt 838


Query: 909  ccaggtaacttctgataccagggtatgatggatcctctcaatatgcacttattaatgatt 968
            | ||||| |||||||||||||||||||||| ||| |||||||||||||||||||||||||
Sbjct: 839  ctaggtatcttctgataccagggtatgatgcatcgtctcaatatgcacttattaatgatt 898


Query: 969  ataaattaccnnnnnnnnntataaatggatcgtttaattgtatcaatacctggcaaatct 1028
            |||| |||||         ||||||| |||||||||||||||||| ||| | ||| ||||
Sbjct: 899  ataagttaccaaaagataatataaat-gatcgtttaattgtatcagtacat-gcatatct 956


Query: 1029 acctattgactttgccctaaaaagtccaaaaggaaaatggcag 1071
            |||   ||||||||||||||||||||| |||||||| ||||||
Sbjct: 957  accatatgactttgccctaaaaagtcc-aaaggaaagtggcag 998

===============================================================================

Cphy2058 reverse

Query= D10_2058C1P17_1.SCF

>cphy_2058


Query: 69   gtgtaatggatagtgatcTTAatggtgatggtgatgatgttctgcgtatttcattaaacc 128
            ||||||||||||||||||||||||||||||||||||||||||||||||||||||||||||
Sbjct: 1359 gtgtaatggatagtgatcttaatggtgatggtgatgatgttctgcgtatttcattaaacc 1300


Query: 129  ttgcaatatctcaggatattttatagtacaactctttctatcaaaaagaccgaagttttc 188
            ||||||||||||||||||||||||||||||||||||||||||||||||||||||||||||
Sbjct: 1299 ttgcaatatctcaggatattttatagtacaactctttctatcaaaaagaccgaagttttc 1240
```

```
Query: 189  cccgtcccctttaaatgcatgattatcccaccagaagcatgtgattccattcgcttttgc 248
            ||||||||||||||||||||||||||||||||||||||||||||||||||||||||||||
Sbjct: 1239 cccgtcccctttaaatgcatgattatcccaccagaagcatgtgattccattcgcttttgc 1180


Query: 249  agcacctatataataagtagcatactctacccgagattctaaattattttctttatctct 308
            ||||||||||||||||||||||||||||||||||||||||||||||||||||||||||||
Sbjct: 1179 agcacctatataataagtagcatactctacccgagattctaaattattttctttatctct 1120


Query: 309  ggcaccaaattcaccgataattacgggaactccatttttataaacttcatatataaact 368
            ||||||||||||||||||||||||||||||||||||||||||||||||||||||||||||
Sbjct: 1119 ggcaccaaattcaccgataattacgggaactccatttttataaacttcatatataaact 1060


Query: 369  atttaaaaaagaatctatctccttagtacatccagctatttttgaattccattctgatat 428
            ||||||||||||||||||||||||||||||||||||||||||||||||||||||||||||
Sbjct: 1059 atttaaaaaagaatctatctccttagtacatccagctatttttgaattccattctgatat 1000


Query: 429  actgccactttcctttggactttttagggcaaagtcatatggtagatatgcatgtactga 488
            ||||||||||||||||||||||||||||||||||||||||||||||||||||||||||||
Sbjct: 999  actgccactttcctttggactttttagggcaaagtcatatggtagatatgcatgtactga 940


Query: 489  tacaattaaacgatcatttatattatcttttggtaacttataatcattaataagtgcata 548
            ||||||||||||||||||||||||||||||||||||||||||||||||||||||||||||
Sbjct: 939  tacaattaaacgatcatttatattatcttttggtaacttataatcattaataagtgcata 880


Query: 549  ttgagacgatgcatcataccctggtatcagaagatacctagaagtattctctcctcccga 608
            ||||||||||||||||||||||||||||||||||||||||||||||||||||||||||||
Sbjct: 879  ttgagacgatgcatcataccctggtatcagaagatacctagaagtattctctcctcccga 820


Query: 609  tttgcgaacggtatcaacaaattcctgatttaattgattaatacattcgattgcttcttt 668
            ||||||||||||||||||||||||||||||||||||||||||||||||||||||||||||
Sbjct: 819  tttgcgaacggtatcaacaaattcctgatttaattgattaatacattcgattgcttcttt 760


Query: 669  acactcaggcttttgataaaatctaaccaccattcgtaattagaaacctgcaagccttgg 728
            |||||||||| |||||| ||||||||||||||||||||||||| ||||||||||||||||
Sbjct: 759  acactcaggc-tttgat-aaatctaaccaccattcgtaattag-aacctgcaagccttgg 703


Query: 729  ttccgttcatcccttcaaataataacttttctccattaggaagaaaatcgttcactttac 788
            || ||||||||||||||||||||||||||||||| ||||||||||||||||||| ||||
Sbjct: 702  tt-cgttcatcccttcaaataataacttttctcca-taggaagaaaatcgttcac-ttac 646


Query: 789  tttgtgtccatactgcgttg 808
             |||||||||||||||||||
Sbjct: 645  -ttgtgtccatactgcgttg 627

================================================================================
```

Cphy2304 forward

Query= H04_2304C2P16_1.SCF

>cphy_2304

```
Query: 70  aaagaaggagataggatcATGacagataccattacgattcattatcatagggatgatggc 129
           ||||||||||||||||||||||||||||||||||||||||||||||||||||||||||||
Sbjct: 1   aaagaaggagataggatcatgacagataccattacgattcattatcatagggatgatggc 60


Query: 130 gattatgaaaaatggaatctgtggttatgggcagaaggaaaagatggcgcggcatattat 189
           ||||||||||||||||||||||||||||||||||||||||||||||||||||||||||||
Sbjct: 61  gattatgaaaaatggaatctgtggttatgggcagaaggaaaagatggcgcggcatattat 120


Query: 190 tttgatggagaagatgcatttgggccgtatgtttcggtttctctggataagagtgcagac 249
           ||||||||||||||||||||||||||||||||||||||||||||||||||||||||||||
Sbjct: 121 tttgatggagaagatgcatttgggccgtatgtttcggtttctctggataagagtgcagac 180


Query: 250 aggatagggtttattgtccgtacggattcttgggaaaaagatgtttcggaggaccggttt 309
           ||||||||||||||||||||||||||||||||||||||||||||||||||||||||||||
Sbjct: 181 aggatagggtttattgtccgtacggattcttgggaaaaagatgtttcggaggaccggttt 240


Query: 310 attgatacatcacttggggatgaaatctggatatccagtggagagagcacattttcttat 369
           ||||||||||||||||||||||||||||||||||||||||||||||||||||||||||||
Sbjct: 241 attgatacatcacttggggatgaaatctggatatccagtggagagagcacattttcttat 300


Query: 370 gaagcaccagaagggtatgaaaaagaggtatcaatagaatctttccagcttaagcttaat 429
           ||||||||||||||||||||||||||||||||||||||||||||||||||||||||||||
Sbjct: 301 gaagcaccagaagggtatgaaaaagaggtatcaatagaatctttccagcttaagcttaat 360


Query: 430 tacttaaggtatgatgaagagtatacggatatttcatttcgattaacctttgaggatggg 489
           ||||||||||||||||||||||||||||||||||||||||||||||||||||||||||||
Sbjct: 361 tacttaaggtatgatgaagagtatacggatatttcatttcgattaacctttgaggatggg 420


Query: 490 acgacagattttcttactaaagagcatatgcgtattgaaaatggtatattaaaagcagaa 549
           ||||||||||||||||||||||||||||||||||||||||||||||||||||||||||||
Sbjct: 421 acgacagattttcttactaaagagcatatgcgtattgaaaatggtatattaaaagcagaa 480


Query: 550 aaagaagtcaaatatggtaaaaagataacacttgatgtattaaaaaatgggttagaagag 609
           ||||||||||||||||||||||||||||||||||||||||||||||||||||||||||||
Sbjct: 481 aaagaagtcaaatatggtaaaaagataacacttgatgtattaaaaaatgggttagaagag 540


Query: 610 gattatcaaggtgtttctttttctacggccaaaattgatgaggaaagtaagctagagatg 669
           ||||||||||||||||||||||||||||||||||||||||||||||||||||||||||||
Sbjct: 541 gattatcaaggtgtttctttttctacggccaaaattgatgaggaaagtaagctagagatg 600


Query: 670 tattggatgcagggaacaggaactatttcaccgaaggctgactttatcaagagaagtaag 729
```

```
           ||||||||||||||||||||||||||||||||||||||||||||||||||||||||||||
Sbjct: 601 tattggatgcagggaacaggaactatttcaccgaaggctgactttatcaagagaagtaag 660


Query: 730 gaaattgaatcggcattgattacttccatgaaggaaataactatcaagcttttccgttcc 789
           |||||||||||||||||||||||||||||||||||||||||  |||||| ||||||||||
Sbjct: 661 gaaattgaatcggcattgattacttccatgaaggaaataacagtcaagc-tttccgttcc 719


Query: 790 ttgcagagtagatgatatcaagcaggatggaatttaaactttcgccgaaaattagctgtt 849
           |||||||||||||||||||||||||||||| |||||||||||||||| ||||||||||||
Sbjct: 720 ttgcagagtagatgatatcaagcaggatgg-atttaaactttcgccg-aaattagctgtt 777


Query: 850 tccaagggggaagcgaccaatacaagggacagtgaatac 888
           ||||||| ||||||||| | |||||||||||||||||||
Sbjct: 778 tccaaggtggaagcgacaagtacaagggacagtgaatac 816
```

==

Cphy2304 reverse

Query= H10_2304C2P17_1.SCF

>cphy_2304

```
Query: 68   gtgtaatggatagtgatcTTAatggtgatggtgatgatggcgttttctttttaaaatcca 127
             ||||||||||||||||||||||||||||||||||||||||||||||||||||||||||||
Sbjct: 3105 gtgtaatggatagtgatcttaatggtgatggtgatgatggcgttttctttttaaaatcca 3046


Query: 128  gaataggactcctcctgcaagaacacagactgtagctatgccgacccataaccatgcatt 187
             ||||||||||||||||||||||||||||||||||||||||||||||||||||||||||||
Sbjct: 3045 gaataggactcctcctgcaagaacacagactgtagctatgccgacccataaccatgcatt 2986


Query: 188  cccgctctctttttctgctacttcctgcatattcgtaggtagctcttggatttcgtctcc 247
             ||||||||||||||||||||||||||||||||||||||||||||||||||||||||||||
Sbjct: 2985 cccgctctctttttctgctacttcctgcatattcgtaggtagctcttggatttcgtctcc 2926


Query: 248  ctttactgcatctttatcgccttttattactgcatctttattctctagaagaactagtgt 307
             ||||||||||||||||||||||||||||||||||||||||||||||||||||||||||||
Sbjct: 2925 ctttactgcatctttatcgccttttattactgcatctttattctctagaagaactagtgt 2866


Query: 308  agattttgcgggcactttaattgttccacctgttatggtttctatgacatcggttccagc 367
             ||||||||||||||||||||||||||||||||||||||||||||||||||||||||||||
Sbjct: 2865 agattttgcgggcactttaattgttccacctgttatggtttctatgacatcggttccagc 2806


Query: 368  agtatcttggtttactaatacatcccatgcatttgcagaaagtactacttccttttcttc 427
             ||||||||||||||||||||||||||||||||||||||||||||||||||||||||||||
Sbjct: 2805 agtatcttggtttactaatacatcccatgcatttgcagaaagtactacttccttttcttc 2746


Query: 428  taatgttccattgaatattaatgcgatttctctaccatcttttaccgcatcatgaattcg 487
```

```
            ||||||||||||||||||||||||||||||||||||||||||||||||||||||||||||
Sbjct: 2745 taatgttccattgaatattaatgcgatttctctaccatcttttaccgcatcatgaattcg 2686


Query: 488  gtaagcaatcacggaagagtcagaatcgacaaattctaacttttgttgtatttcttctgc 547
            ||||||||||||||||||||||||||||||||||||||||||||||||||||||||||||
Sbjct: 2685 gtaagcaatcacggaagagtcagaatcgacaaattctaacttttgttgtatttcttctgc 2626


Query: 548  tgtctgcatacggaatgcatcgtaagtttttctaagagcgattaatcctttgtaatattc 607
            ||||||||||||||||||||||||||||||||||||||||||||||||||||||||||||
Sbjct: 2625 tgtctgcatacggaatgcatcgtaagtttttctaagagcgattaatcctttgtaatattc 2566


Query: 608  aaataaatccttgtattctgtcttattgtcccaattcaacatatttaatgctgtctggcg 667
            ||||||||||||||||||||||||||||||||||||||||||| ||||||||||||||||
Sbjct: 2565 aaataaatccttgtattctgtcttattgtcccaattcaacata-ttaatgctgtctggcg 2507


Query: 668  actgatatgaattatcattttcccttctttgccctagccatttcttccccctgcttgga 727
            ||||||| ||||||||| ||||||||||||| |||||||| |||||| ||||||||||||
Sbjct: 2506 actgataggaattatca-tttcccttctttgtcctagcca-tttctt-cccctgcttgga 2450


Query: 728  aaaacggaattccttgggaagtcaggacaatttgccgccgacaattt 774
            |||||||||||||||||||||| || |||| ||||||| ||||||||
Sbjct: 2449 aaaacggaattccttgggaagttagtacaa-ttgccgcagacaattt 2404

================================================================================

Cphy2344 forward

Query= B05_2344C1P16_1.SCF

>cphy_2344


Query: 69  aaagaaggagataggatcATGgtaaatcagaatccgtctgaattaaatcaggatgaaagt 128
           ||||||||||||||||||||||||||||||||||||||||||||||||||||||||||||
Sbjct: 1   aaagaaggagataggatcatggtaaatcagaatccgtctgaattaaatcaggatgaaagt 60


Query: 129 caaaaggaaaaggaagaaaacgatgatgaaggcacaccggaagtgtctcaggatgaaact 188
           ||||||||||||||||||||||||||||||||||||||||||||||||||||||||||||
Sbjct: 61  caaaaggaaaaggaagaaaacgatgatgaaggcacaccggaagtgtctcaggatgaaact 120


Query: 189 aaggcagttattccttatgactatgtacagaatttaaatataattgatgataactaccgt 248
           ||||||||||||||||||||||||||||||||||||||||||||||||||||||||||||
Sbjct: 121 aaggcagttattccttatgactatgtacagaatttaaatataattgatgataactaccgt 180


Query: 249 aatttttatgaaatattcgtttactctttttatgacagcaacggtgacggcattggagat 308
           ||||||||||||||||||||||||||||||||||||||||||||||||||||||||||||
Sbjct: 181 aatttttatgaaatattcgtttactctttttatgacagcaacggtgacggcattggagat 240


Query: 309 attaatggtgttatatcaaaattagactatattaatgatggcaatgatgcgaccgattca 368
```

```
           ||||||||||||||||||||||||||||||||||||||||||||||||||||||||||||
Sbjct: 241 attaatggtgttatatcaaaattagactatattaatgatggcaatgatgcgaccgattca 300


Query: 369 gatctaggctttaacgggatttggctaatgcctatcatgccatcaaccacatatcacaaa 428
           ||||||||||||||||||||||||||||||||||||||||||||||||||||||||||||
Sbjct: 301 gatctaggctttaacgggatttggctaatgcctatcatgccatcaaccacatatcacaaa 360


Query: 429 tatgacgtaacggactattacaatattgatccacagtacgggacattagaggattttaaa 488
           ||||||||||||||||||||||||||||||||||||||||||||||||||||||||||||
Sbjct: 361 tatgacgtaacggactattacaatattgatccacagtacgggacattagaggattttaaa 420


Query: 489 aatctggtttctgaatgtcataaacgtggaattcatttaattatcgactttgtatttcaa 548
           |||||||||||||||||||||||||||||||||||||||||||||||||||||| |||||
Sbjct: 421 aatctggtttctgaatgtcataaacgtggaattcatttaattatcgactttgta-ttcaa 479


Query: 549 tcacacctcggcaaaacatccttggtttttaaaagcggtttcctatctggaaatcttgaa 608
           ||||||||||||||||||||||||||||||| ||||||||||||||||||||| ||||||
Sbjct: 480 tcacacctcggcaaaacatccttggtttttagaagcggtttcctatctggaaagcttgaa 539


Query: 609 tgtaggagaaaagcctgattt 629
            |  |||||| ||||||||||
Sbjct: 540 ggagggagaagagcctgattt 560

================================================================================

Cphy2344 reverse

Query= B11_2344C1P17_1.SCF

>cphy_2344


Query: 67   gtgtaatggatagtgatcTTAatggtgatggtgatgatgttttaatataactatcgaata 126
            ||||||||||||||||||||||||||||||||||||||||||||||||||||||||||||
Sbjct: 1710 gtgtaatggatagtgatcttaatggtgatggtgatgatgttttaatataactatcgaata 1651


Query: 127  cttaggcattgaaacaacaccatctgacatggtaactgcatttccatccactgataagta 186
            ||||||||||||||||||||||||||||||||||||||||||||||||||||||||||||
Sbjct: 1650 cttaggcattgaaacaacaccatctgacatggtaactgcatttccatccactgataagta 1591


Query: 187  tcctctgatatttaactccgtaagacctgcatcttttaacaatatgttggctgattctgt 246
            ||||||||||||||||||||||||||||||||||||||||||||||||||||||||||||
Sbjct: 1590 tcctctgatatttaactccgtaagacctgcatcttttaacaatatgttggctgattctgt 1531


Query: 247  attaatattataaagaatcacaatttcactgccttggtataccttttttaattgcactgat 306
            ||||||||||||||||||||||||||||||||||||||||||||||||||||||||||||
Sbjct: 1530 attaatattataaagaatcacaatttcactgccttggtataccttttttaattgcactgat 1471


Query: 307  gtcctttgtgcataattcttcaattacactcatatctcctcttgcaatctctgggttctc 366
```

```
          ||||||||||||||||||||||||||||||||||||||||||||||||||||||||||||
Sbjct: 1470 gtcctttgtgcataattcttcaattacactcatatctcctcttgcaatctctgggttctc 1411


Query: 367  attacggattctaactgctctcttatagtagttataaagggatagaggatctttcatttg 426
            ||||||||||||||||||||||||||||||||||||||||||||||||||||||||||||
Sbjct: 1410 attacggattctaactgctctcttatagtagttataaagggatagaggatctttcatttg 1351


Query: 427  ctcatcgacaggagggaacttctgctctacagaatctgcattagctggtggtgtagtaat 486
            ||||||||||||||||||||||||||||||||||||||||||||||||||||||||||||
Sbjct: 1350 ctcatcgacaggagggaacttctgctctacagaatctgcattagctggtggtgtagtaat 1291


Query: 487  tcccgtagtatctgtagctgaccactgcataggcaatcttttattctcatccttggttcc 546
            ||||||||||||||||||||||||||||||||||||||||||||||||||||||||||||
Sbjct: 1290 tcccgtagtatctgtagctgaccactgcataggcaatcttttattctcatccttggttcc 1231


Query: 547  cttactattcataccaatctcttctccataatagacatatgggctgccattcatggtcaa 606
            ||||||||||||||||||||||||||||||||||||||||||||||||||||||||||||
Sbjct: 1230 cttactattcataccaatctcttctccataatagacatatgggctgccattcatggtcaa 1171


Query: 607  taacatacctgctgacattttcatttgatcttcatcattgacaccttgggcaaatattct 666
            |||||||||||||||||||||||||||||||||||||||||||| ||||||| |||||||
Sbjct: 1170 taacatacctgctgacattttcatttgatcttcatcattgacacattgggcagatattct 1111


Query: 667  tgtggtatcatgattaccaataaacgaatcatctatatatctaggattannnnnnnttat 726
            ||||||||||||||||| |||||||| | ||||||||||| ||||||||       ||||
Sbjct: 1110 tgtggtatcatgattactaataaacggagcatctatatatttaggatta-ccctccttat 1052


Query: 727  acttctcatctaaacgaattaaaattttaccaaaatttttaccataaaaactaccccaat 786
            |||||||||||||||||| |||| ||||| |||| ||||||||| || || |||||  | 
Sbjct: 1051 acttctcatctaaacgaagtaaagttttagcaaagtttttaccagaagaagtaccc--aa 994


Query: 787  ttttctggctgtgttt 802
            ||||||||||||||||
Sbjct: 993  ttttctggctgtgttt 978
```

Cphy2350 forward

Query= G05_2350C3P16_1.SCF

>cphy_2350

```
Query: 71   aaagaaggagataggatcATGaaatttgaagctatttaccacagaacctctgataattat 130
            ||||||||||||||||||||||||||||||||||||||||||||||||||||||||||||
Sbjct: 1    aaagaaggagataggatcatgaaatttgaagctatttaccacagaacctctgataattat 60


Query: 131  tgttatccgttaaatgaagaggatcttattatcaatatcaaaacgggccacgatatagag 190
```

```
            ||||||||||||||||||||||||||||||||||||||||||||||||||||||||||||
Sbjct: 61   tgttatccgttaaatgaagaggatcttattatcaatatcaaaacgggccacgatatagag 120


Query: 191  agggtttttatctattatggggatcctttcgaaggtggaattttaggcggtaattggact 250
            ||||||||||||||||||||||||||||||||||||||||||||||||||||||||||||
Sbjct: 121  agggtttttatctattatggggatcctttcgaaggtggaattttaggcggtaattggact 180


Query: 251  tggaatggtgtggaagaagagttgatttataaaaagaacctaacacatcatatttggtgg 310
            ||||||||||||||||||||||||||||||||||||||||||||||||||||||||||||
Sbjct: 181  tggaatggtgtggaagaagagttgatttataaaaagaacctaacacatcatatttggtgg 240


Query: 311  acaacaacagtaaagccaaagtttaaaaggtgtaaatattattttaaattagttgctaat 370
            ||||||||||||||||||||||||||||||||||||||||||||||||||||||||||||
Sbjct: 241  acaacaacagtaaagccaaagtttaaaaggtgtaaatattattttaaattagttgctaat 300


Query: 371  gatacttcttactattattttgaagatggattctatacagaagcagaaatgaatcatcaa 430
            ||||||||||||||||||||||||||||||||||||||||||||||||||||||||||||
Sbjct: 301  gatacttcttactattattttgaagatggattctatacagaagcagaaatgaatcatcaa 360


Query: 431  gacaaaaatttagtatatttcacattcccatggatgaatagtattgatattaacaaaact 490
            ||||||||||||||||||||||||||||||||||||||||||||||||||||||||||||
Sbjct: 361  gacaaaaatttagtatatttcacattcccatggatgaatagtattgatattaacaaaact 420


Query: 491  cctgactgggtaaatgataccgtatggtatcagatttttccggaacgttttaataacgga 550
            ||||||||||||||||||||||||||||||||||||||||||||||||||||||||||||
Sbjct: 421  cctgactgggtaaatgataccgtatggtatcagatttttccggaacgttttaataacgga 480


Query: 551  gataaagaaaatgacccgaagaatgtaaaagcttggggatttcatacagtgagcaatgat 610
            ||||||||||||||||||||||||||||||||||||||||||||||||||||||||||||
Sbjct: 481  gataaagaaaatgacccgaagaatgtaaaagcttggggatttcatacagtgagcaatgat 540


Query: 611  gagttttatggcggagatttacagggtattataaatcgattggattatttggcggataga 670
            |||||||||||||||||||||||||||||||||||||||||||||||||||||||||| |
Sbjct: 541  gagttttatggcggagatttacagggtattataaatcgattggattatttggcggatata 600


Query: 671  ggcataagcggaatatatctgacacctatctttgaagcaaaatccagtcataaatacgat 730
            |||||||||||||||||||||||||||||||||||||||||  |||||||||||||||||
Sbjct: 601  ggcataagcggaatatatctgacacctatctttgaagcaaataccagtcataaatacgat 660


Query: 731  accaaaaac-atatgaagatggat-cacatttgggtggatgaaaaattattttaaaaacc 788
            |||||| || |||||||||| ||| ||||||| ||| ||||| ||| |||||  |||| |
Sbjct: 661  accaaagactatatgaagattgatccacattttggt-gatgagaaagtattt--aaaaac 717


Query: 789  ttatttgaaccagctcatgaaaaaggcattcgtatttatgcttgatggtgtatttaatca 848
            ||| ||||  ||||||||||||| |||||||||| |||||||||||||||||||||||||
Sbjct: 718  ttagttgatacagctcatgaaaagggcattcgta-ttatgcttgatggtgtatttaatca 776
```

```
Query: 849  ttgcggtaatctattgtgctccatggctaagtgttttaaagaatggacttgtactccata 908
            ||||||||||| | | |||||||||||||  |||||| |||||||||| || ||| || |
Sbjct: 777  ttgcggtaatc-agtttgctccatggctagatgttttgaagaatggacctg-act-caaa 833


Query: 909  gttttttaactgggttttgattaaaaattggcctttttacaaagaaaaatcaaaatacaa 968
            || |||||||||| || ||||||| || ||||||||| |||||| || |||| |||||||
Sbjct: 834  gtattttaactggtttatgattaacaaatggccttttaacaaag-aagatcataatacaa 892


Query: 969  aggaatgggtcattttatctcttttgccttttacttcca 1007
            | | |||||||||||||| || |||| ||||||||||||
Sbjct: 893  atg-atgggtcattttat-tcctttg-cttttacttcca 928
```

===

Cphy2350 reverse

Query= G11_2350C3P17_1.SCF

>cphy_2350

```
Query: 67   gtgtaatggatagtgatcTTAatggtgatggtgatgatgtatctgctgaataaaaacac 125
            |||||||||||||||||||||||||||||||||||||||||||||||||||||||||||
Sbjct: 1807 gtgtaatggatagtgatcttaatggtgatggtgatgatgtatctgctgaataaaaacac 1748


Query: 126  cttttggttttagaatagtatcttcatttaataggctaaataattcattacctttccttt 185
            ||||||||||||||||||||||||||||||||||||||||||||||||||||||||||||
Sbjct: 1747 cttttggttttagaatagtatcttcatttaataggctaaataattcattacctttccttt 1688


Query: 186  gaactacgatactatccttagagcaatttaaaataacttcaacctgtttatgatcttcgt 245
            ||||||||||||||||||||||||||||||||||||||||||||||||||||||||||||
Sbjct: 1687 gaactacgatactatccttagagcaatttaaaataacttcaacctgtttatgatcttcgt 1628


Query: 246  ctgtttttatatagtgaattactctgttattattcttatcctcaataaaatgataatgtc 305
            ||||||||||||||||||||||||||||||||||||||||||||||||||||||||||||
Sbjct: 1627 ctgtttttatatagtgaattactctgttattattcttatcctcaataaaatgataatgtc 1568


Query: 306  tgcttttgaaagcattgtttgtttttcttaaatgaatcagtgcttttatgatttcaattc 365
            ||||||||||||||||||||||||||||||||||||||||||||||||||||||||||||
Sbjct: 1567 tgcttttgaaagcattgtttgtttttcttaaatgaatcagtgcttttatgatttcaattc 1508


Query: 366  tatctttaaacaaacctgcatcgatgtcttcccaaggcatacatctacgacagtccggat 425
            ||||||||||||||||||||||||||||||||||||||||||||||||||||||||||||
Sbjct: 1507 tatctttaaacaaacctgcatcgatgtcttcccaaggcatacatctacgacagtccggat 1448


Query: 426  catagcttccttccattgctatttcagttccataataaatacatggactacccggcatgg 485
            ||||||||||||||||||||||||||||||||||||||||||||||||||||||||||||
Sbjct: 1447 catagcttccttccattgctatttcagttccataataaatacatggactacccggcatgg 1388
```

```
Query: 486  taaatagaactgctaactgctggtaaaaaatatcaatatcttttactttatctatcaatc 545
            ||||||||||||||||||||||||||||||||||||||||||||||||||||||||||||
Sbjct: 1387 taaatagaactgctaactgctggtaaaaaatatcaatatcttttactttatctatcaatc 1328


Query: 546  tattggtatcatgggaatccaataaattgaataagacatcgtttgtctgctgcatataca 605
            ||||||||||||||||||||||||||||||||||||||||||||||||||||||||||||
Sbjct: 1327 tattggtatcatgggaatccaataaattgaataagacatcgtttgtctgctgcatataca 1268


Query: 606  tggtaaagttattgattaatggcacattcaaagtcatagttagttttctccggataaacc 665
            ||||||||||| ||||||||||||||||||||||||||||||||||||||||||||||||
Sbjct: 1267 tggtaaagtta-tgattaatggcacattcaaagtcatagttagttttctccggataaacc 1209


Query: 666  caataatctgctaaagaagtggctaatgggtaattcataactccatcaaattcattctcc 725
            |||||||||||||||||||||||||||||||||||||||||||||||||||||| ||| |
Sbjct: 1208 caataatctgctaaagaagtggctaatgggtaattcataactccatcaaattca-tct-c 1151


Query: 726  catggaaccaagtaattacatcatgccataactcacctaatatataaaaaatcgggttct 785
            |||| ||||||||||||  |||||||||||||||||||||||||| |||||||||||| |
Sbjct: 1150 catgtaaccaagtaatagcatcatgccataactcacctaatatat-aaaaatcgggtttt 1092


Query: 786  aactctttgggtcaacttttttaactccttac 817
            |||||||| |||||||||| |||| |||||||
Sbjct: 1091 aactcttt-ggtcaactttcttaaatccttac 1061

===============================================================================

 Cphy2567 forward

Query= HR0ADKBY98ZF02_2567C2P16_1.SCF

>cphy_2567


Query: 68  aaagaaggagataggatcATGcaaacatttactgttgaattaccggtttttccagataga 127
           ||||||||||||||||||||||||||||||||||||||||||||||||||||||||||||
Sbjct: 1   aaagaaggagataggatcatgcaaacatttactgttgaattaccggtttttccagataga 60


Query: 128 caggtgagtattacagattataaggcagtgtctgggggtattgaatcgaatacagctgca 187
           ||||||||||||||||||||||||||||||||||||||||||||||||||||||||||||
Sbjct: 61  caggtgagtattacagattataaggcagtgtctgggggtattgaatcgaatacagctgca 120


Query: 188 atcaatcaagcaattacggagttatcaaagctcggtggtggtacggttaacgttccagaa 247
           ||||||||||||||||||||||||||||||||||||||||||||||||||||||||||||
Sbjct: 121 atcaatcaagcaattacggagttatcaaagctcggtggtggtacggttaacgttccagaa 180


Query: 248 ggcatctggcttacaggtcctattactttaaagtcaaatattaaccttcatctagaaaag 307
           ||||||||||||||||||||||||||||||||||||||||||||||||||||||||||||
Sbjct: 181 ggcatctggcttacaggtcctattactttaaagtcaaatattaaccttcatctagaaaag 240
```

```
Query: 308 ggtgcattaattaccttcgataagaatccagaagaatacccaattattcttactaattat 367
           ||||||||||||||||||||||||||||||||||||||||||||||||||||||||||||
Sbjct: 241 ggtgcattaattaccttcgataagaatccagaagaatacccaattattcttactaattat 300


Query: 368 gagggacaaccaagacttcgtgcggtttctccaatccatgcatttgatgaagagaatatt 427
           ||||||||||||||||||||||||||||||||||||||||||||||||||||||||||||
Sbjct: 301 gagggacaaccaagacttcgtgcggtttctccaatccatgcatttgatgaagagaatatt 360


Query: 428 gcaattactggtgaaggtgttattgatggtaatggtcacgaatggcgtccattaaaagag 487
           ||||||||||||||||||||||||||||||||||||||||||||||||||||||||||||
Sbjct: 361 gcaattactggtgaaggtgttattgatggtaatggtcacgaatggcgtccattaaaagag 420


Query: 488 tttaaggtaacgaagaaacagtggcaagcacgtttaaagaagagtccttatgtaattgat 547
           ||||||||||||||||||||||||||||||||||||||||||||||||||||||||||||
Sbjct: 421 tttaaggtaacgaagaaacagtggcaagcacgtttaaagaagagtccttatgtaattgat 480


Query: 548 acgaaagaaggcggtatctggtatcctagtaaaacttcttatgaaggttgtttagaagga 607
           ||||||||||||||||||||||||||||||||||||||||||||||||||||||||||||
Sbjct: 481 acgaaagaaggcggtatctggtatcctagtaaaacttcttatgaaggttgtttagaagga 540


Query: 608 gaagtaagtgttgaagatccagatgcacttaagaaggcagctccaaactatgatctctac 667
           ||||||||||||||||||||||||||||||||||||||||||||||||||||||||||||
Sbjct: 541 gaagtaagtgttgaagatccagatgcacttaagaaggcagctccaaactatgatctctac 600


Query: 668 cgtccagttatgacgaacttagttcgctgtaataagatattaatagaaggagttacactt 727
           ||||||||||||||||||||||||||||||||||||||||||||||||||||||||||||
Sbjct: 601 cgtccagttatgacgaacttagttcgctgtaataagatattaatagaaggagttacactt 660


Query: 728 caaaattcacccgcttggaaccttcacccattactttgtacgaatctaacccttcgaaat 787
           ||||||||||||||||||||||||||||||||||||||||||||||||||||||||||||
Sbjct: 661 caaaattcacccgcttggaaccttcacccattactttgtacgaatctaacccttcgaaat 720


Query: 788 gcgtttattcgtaatgctaattttgctcaaaatggtgatggacttgacttagagtcttgt 847
           ||||||||||||||||||||||||||||||||||||||||||||||||||||||||||||
Sbjct: 721 gcgtttattcgtaatgctaattttgctcaaaatggtgatggacttgacttagagtcttgt 780


Query: 848 cgtttcgttgacatctacggagtgaaatttgatgttggtgatgatgctatttgcataaaa 907
           ||||||||||||||||||||||||||||||||||||||||||||||||||||||||||||
Sbjct: 781 cgtttcgttgacatctacggagtgaaatttgatgttggtgatgatgctatttgcataaaa 840


Query: 908 tccggtaaaaatgcaattggaagaaaaattaccgtgccaacaaaacacgttcgcatta 965
           |||||||||||||| ||||||||||||||||||||||||||| |||||||||||||||
Sbjct: 841 tccggtaaaaatgcgattggaagaaaaattaccgtgccaacagaacacgttcgcatta 898

=================================================================================
```

Cphy2567 reverse

Query= HR0ADKBY98ZF02_2567C2P17_1.SCF

>cphy_2567

```
Query: 65   gtgtaatggatagtgatcTTAatggtgatggtgatgatggaaattatgactacagtcaga 124
            ||||||||||||||||||||||||||||||||||||||||||||||||||||||||||||
Sbjct: 1479 gtgtaatggatagtgatcttaatggtgatggtgatgatggaaattatgactacagtcaga 1420


Query: 125  taaaacatcctctttaaatacaaaagattgatctaaattgtcctgattagttaccgaaac 184
            ||||||||||||||||||||||||||||||||||||||||||||||||||||||||||||
Sbjct: 1419 taaaacatcctctttaaatacaaaagattgatctaaattgtcctgattagttaccgaaac 1360


Query: 185  attcttaaagaaaatctgatttgcattcttaatctcgattgctttcttcgcttgaatcat 244
            ||||||||||||||||||||||||||||||||||||||||||||||||||||||||||||
Sbjct: 1359 attcttaaagaaaatctgatttgcattcttaatctcgattgctttcttcgcttgaatcat 1300


Query: 245  aacattttctaatatgatatcatggattggcatctgctctagcccatcaattttaattgc 304
            ||||||||||||||||||||||||||||||||||||||||||||||||||||||||||||
Sbjct: 1299 aacattttctaatatgatatcatggattggcatctgctctagcccatcaattttaattgc 1240


Query: 305  ttgtcctgctcgaagacagtttacatcacggatggttatattcttaaactctggtatatc 364
            ||||||||||||||||||||||||||||||||||||||||||||||||||||||||||||
Sbjct: 1239 ttgtcctgctcgaagacagtttacatcacggatggttatattcttaaactctggtatatc 1180


Query: 365  ctctttcgaaatcgtatctggttcatcagacgcttgatggtctaaacggaataaggtata 424
            ||||||||||||||||||||||||||||||||||||||||||||||||||||||||||||
Sbjct: 1179 ctctttcgaaatcgtatctggttcatcagacgcttgatggtctaaacggaataaggtata 1120


Query: 425  acccatggtaaatataattgcgtcttcttcaatatccgtcatctgaatgttacgaattgt 484
            ||||||||||||||||||||||||||||||||||||||||||||||||||||||||||||
Sbjct: 1119 acccatggtaaatataattgcgtcttcttcaatatccgtcatctgaatgttacgaattgt 1060


Query: 485  aatatcctccacaactccaccgcgtccaatcgcactcttaaaacgtattcctgtatcggt 544
            ||||||||||||||||||||||||||||||||||||||||||||||||||||||||||||
Sbjct: 1059 aatatcctccacaactccaccgcgtccaatcgcactcttaaaacgtattcctgtatcggt 1000


Query: 545  acctaaaaataagcaattttctattacaacatctcgtacgccacgggacatctcactacc 604
            ||||||||||||||||||||||||||||||||||||||||||||||||||||||||||||
Sbjct: 999  acctaaaaataagcaattttctattacaacatctcgtacgccacgggacatctcactacc 940


Query: 605  aattacaaatccaccatgtccatgatatacgacacaatctctaatgcgaacgtgttctgt 664
            ||||||||||||||||||||||||||||||||||||||||||||||||||||||||||||
Sbjct: 939  aattacaaatccaccatgtccatgatatacgacacaatctctaatgcgaacgtgttctgt 880


Query: 665  tggcacggtaatttttcttccaatcgcatttttaccggattttatgcaaatagcatcatc 724
            ||||||||||||||||||||||||||||||||||||||||||||||||||||||||||||
```

```
Sbjct: 879  tggcacggtaatttttcttccaatcgcatttttaccggattttatgcaaatagcatcatc 820


Query: 725  accaacatcaaatttcactccgtagatgtcaacgaaacgacaagactctaagtcaagtcc 784
            ||||||||||||||||||||||||||||||||||||||||||||||||||||||||||||
Sbjct: 819  accaacatcaaatttcactccgtagatgtcaacgaaacgacaagactctaagtcaagtcc 760


Query: 785  atcaccattttgagcaaaattagcattacgaataaacgcatttcgaagggttagattcgt 844
            ||||||||||||||||||||||||||||||||||||||||||||||||||||||||||||
Sbjct: 759  atcaccattttgagcaaaattagcattacgaataaacgcatttcgaagggttagattcgt 700


Query: 845  acaaagtaatgggtgaaggttccaagcgggtgaattttgaaatgtaacttccttctatta 904
            |||||||||||||||||||||||||||||||||||||||||| |||||| |||||||||||
Sbjct: 699  acaaagtaatgggtgaaggttccaagcgggtgaattttgaagtgtaac-tccttctatta 641


Query: 905  atatctttattaacgcgaactaaatttcgtcataactgg 943
            ||||| ||||||  ||||||| || ||||||||||||||
Sbjct: 640  atatc-ttattacagcgaact-aagttcgtcataactgg 604
```
